# Copper-catalyzed oxidative benzylic C-H cyclization via iminyl radical from intermolecular anion-radical redox relay

**DOI:** 10.1038/s41467-019-08849-z

**Published:** 2019-02-22

**Authors:** Xiang-Huan Shan, Hong-Xing Zheng, Bo Yang, Lin Tie, Jia-Le Fu, Jian-Ping Qu, Yan-Biao Kang

**Affiliations:** 1 Department of Chemistry, University of Science and Technology of China, 230026 Hefei, Anhui China; 2 Institute of Advanced Synthesis, School of Chemistry and Molecular Engineering, Jiangsu National Synergetic Innovation Center for Advanced Materials, Nanjing Tech University, 211816 Nanjing, China

## Abstract

Base-promoted C-H cleavage without transition metals opens a practical alternative for the one based on noble metals or radical initiators. The resulting carbanion can pass through radical addition to unsaturated bonds like C-N or C-C triple bonds, in which stoichiometric oxidants are needed. When in situ C-H cleavage meets catalytic carbanion-radical relay, it turns to be challenging but has not been accomplished yet. Here we report the combination of base-promoted benzylic C-H cleavage and copper-catalyzed carbanion-radical redox relay. Catalytic amount of naturally abundant and inexpensive copper salt, such as copper(II) sulfate, is used for anion-radical redox relay without any external oxidant. By avoiding using N-O/N-N homolysis or radical initiators to generate iminyl radicals, this strategy realizes modular synthesis of N-H indoles and analogs from abundant feedstocks, such as toluene and nitrile derivatives, and also enables rapid synthesis of large scale pharmaceuticals.

## Abstract (web summary)

The synthesis of high value heterocycles from cheap starting materials remains challenging. Here, the authors develop a new strategy to realize practical and modular synthesis of N-H indoles and analogs from toluene and nitrile derivatives using naturally abundant copper salt as catalyst.

## Introduction

**Fig. 1:**
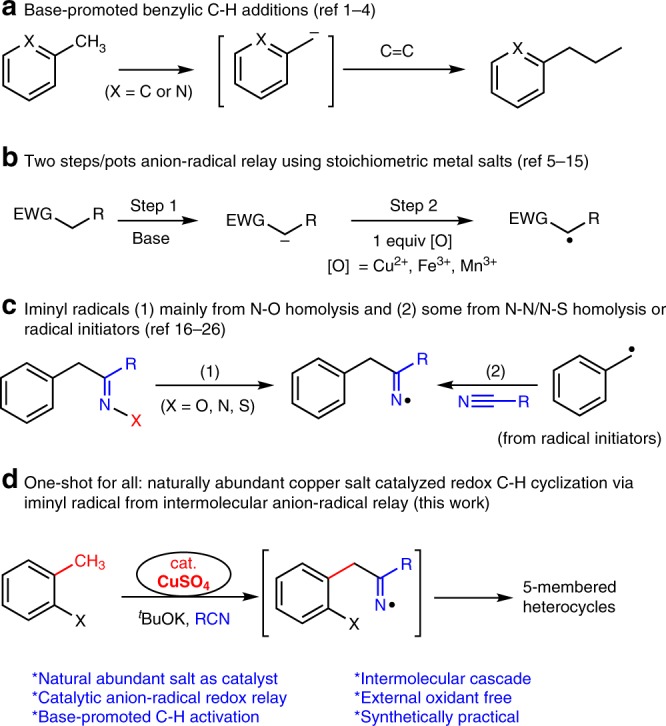
Formation of iminyl radical from anion/radical redox relay other than N-O cleavage or initiators. **a** Base-promoted benzylic C-H cleavage in the absence of transition metal catalysts followed by an addition to alkenes. **b** Two steps/pots anion-radical relay using metal oxidants. **c** Methods for the generation of iminyl radicals. **d** This work: naturally abundant copper salt catalyzed redox C-H cyclization via iminyl radical from intermolecular anion-radical relay

Base-promoted C-H cleavage in the absence of transition metal catalysts, especially, without noble metals, such as rhodium, palladium, and iridium, has emerged near recently (Fig. 1a)^1–4^. This approach opens a practical and cheap alternative for previously established C-H cleavage based on noble metals or radical initiators. It has been well known that carbanion can pass through radical addition to unsaturated bonds like C-N or C-C triple bonds using a metal oxidant such as Cu(II) (Fig. 1b)^5–15^. Stoichiometric oxidants are needed for high turnovers^5^. The catalytic copper-mediated anion-radical relay is not possible unless an extra oxidant is presented. Thus problem emerges when in situ C-H cleavage meets carbanion-radical relay without stoichiometric high oxidation state metals or oxidants. To the best of our knowledge, the example of anion-radical oxidative relay using catalytic amount of copper salt has not been accomplished yet^5^.

On the other hand, iminyl radical has been well-established as intermediates for the construction of *N*-containing 5- and 6-membered heterocycles including indoles and pyridines^16–21^. Iminyl radical is normally generated from N-O bond cleavage using either light or initiators (Fig. 1c), which has attracted high interests of chemists with the renaissance of radical reactions in organic synthesis^17,22–25^. It has been barely reported that intermolecular carbanion-radical relay can furnish iminyl radical by catalytic transition metals. Therefore a strategy needs to be established for aforementioned challenges.

As a typical 5-memebrered *N*-containing rings, indoles are among the most widely existing skeletons in natural products as well as in pharmaceuticals. Despite that the approaches for the synthesis indoles have been well established^26–31^, actually, most methods are based on the derivatives of anilines^26,32–64^, where the C-N bonds have been generally preinstalled. Therefore, we envision that a strategy through benzylic C-H addition and iminyl radical relay can enable the cyclization of toluenes and nitriles to indoles. Herein, we report our recent results (Fig. 1d).

**Fig. 2:**
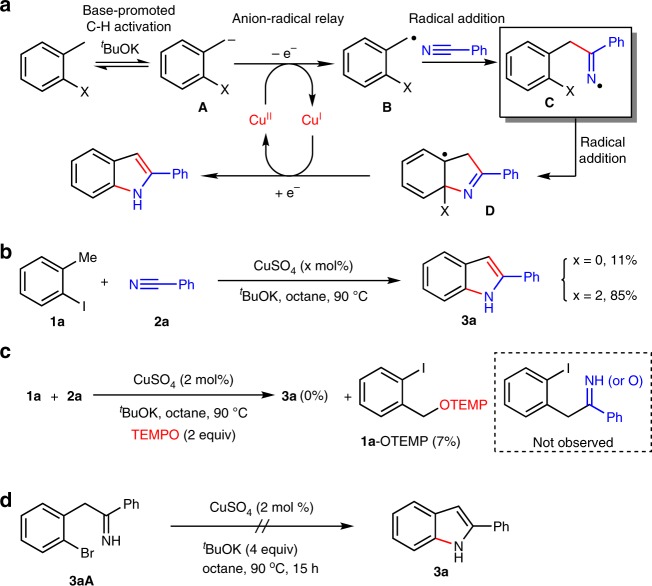
Iminyl radical from anion-radical redox relay. **a** Pathway for the copper-catalyzed anion-radical redox relay. **b** The anion-radical redox relay of **1a** and **2a** in the presence of 2 mol% CuSO_4_ affords 85% of **3a**. **c** TEMPO inhibits the anion-radical redox was confirmed by the observation of **1a**-OTEMP adduct. **d** No Ullmann-type intramolecular cyclization was observed

Herein, we report our recent results in the probe of the copper-catalyzed anion-radical redox relay, we initialize the reaction by a base-promoted benzylic C-H cleavage to generate the benzyl anion **A**, which passes through a Cu(II)-mediated oxidation to radical **B**^5^. The intermolecular radical addition of **B** to PhCN generates iminyl radial **C**, which is trapped by aryl ring to form **D**^65–67^. **D** is reduced by Cu(I) to indoles with the regeneration of Cu(II) (Fig. 2a). The reaction in the presence of 2 mol% CuSO_4_ affords 85% of **3a** (Fig. 2b), whereas the radical trapping experiment shows that TEMPO totally inhibits the reaction with the observation of **1a**-OTEMP adduct (Fig. 2c), suggesting the radical pathway should be rational. No Ullmann-type intramolecular cyclization further proves the radical pathway (Fig. 2d). Further investigation using palladium instead of copper salts proves no promotion effect. Therefore, CuSO_4_ herein plays a crucial role of redox catalyst to generate benzylic radical from benzylic anion and enables the efficient synthesis of N-H indoles and analogs from toluene and nitrile derivatives.

## Results

### Investigations of reaction conditions and scope

The reaction conditions were optimized. First, various copper salts were investigated as catalysts in the cyclization of toluene **1a** with nitrile **2a** (Table 1). CuO and Cu_2_O afford similar yields (entries 2 and 3), indicating that either Cu(II) or Cu(I) might be involved in this reaction. CuSO_4_ gives the best yield as 77% (entry 6). The reaction in octane is better than that in other solvents, such as dioxane, toluene, and DMF (entries 6, 9–11). Increasing the concentration results in the slight increase of yield to 81% (entries 6 and 12). Reducing the catalyst loading from 10 to 2 mol % also gives rise to the increase of yields (entries 12–13). The unreacted nitrile **2a** was recovered almost quantitatively (entry 13). Palladium catalysts do not promote this reaction (entries 16–18). The reaction condition in entry 13 was chosen for the standard reaction conditions where 2 mol % of CuSO_4_ was used as catalyst.

**Table 1:** Reaction conditions


Entry^a^	[cat](mol %)	Base	Solvent	3a(%)^c^
1	CuTC (10)	^*t*^BuOK	octane	48
2	Cu_2_O (10)	^*t*^BuOK	octane	62
3	CuO (10)	^*t*^BuOK	octane	68
4	CuCl (10)	^*t*^BuOK	octane	57
5	CuCl_2_ (10)	^*t*^BuOK	octane	63
6	CuSO_4_ (10)	^*t*^BuOK	octane	77
7	CuSO_4_ (10)	^*t*^BuONa	octane	0
8	CuSO_4_ (10)	KOH	octane	0
9	CuSO_4_ (10)	^*t*^BuOK	dioxane	70
10	CuSO_4_ (10)	^*t*^BuOK	toluene	45
11	CuSO_4_ (10)	^*t*^BuOK	DMF	22
12^b^	CuSO_4_ (10)	^*t*^BuOK	octane	81
13^b, d^	CuSO_4_ (2)	^*t*^BuOK	octane	85
14	Cu(300 mesh)	^*t*^BuOK	octane	54
15	none	^*t*^BuOK	octane	11
16	Pd_2_(dba)_3_ (5)	^*t*^BuOK	octane	0
17	Pd(PPh_3_)_4_ (10)	^*t*^BuOK	octane	17
18	Pd(OAc)_2_ (10)	^*t*^BuOK	octane	15

^a^Conditions: **1a** (1 mmol), **2a** (5 mmol), [cat] (x mol %), ^*t*^BuOK (4 mmol), solvent (1 mL), 15 h, 90 °C, argon

^b^Octane (0.75 mL)

^c^Determined by ^1^H NMR

^d^By column purification, 4.1 out of 5 equiv of nitriles **2a** was recovered

**Fig. 3:**
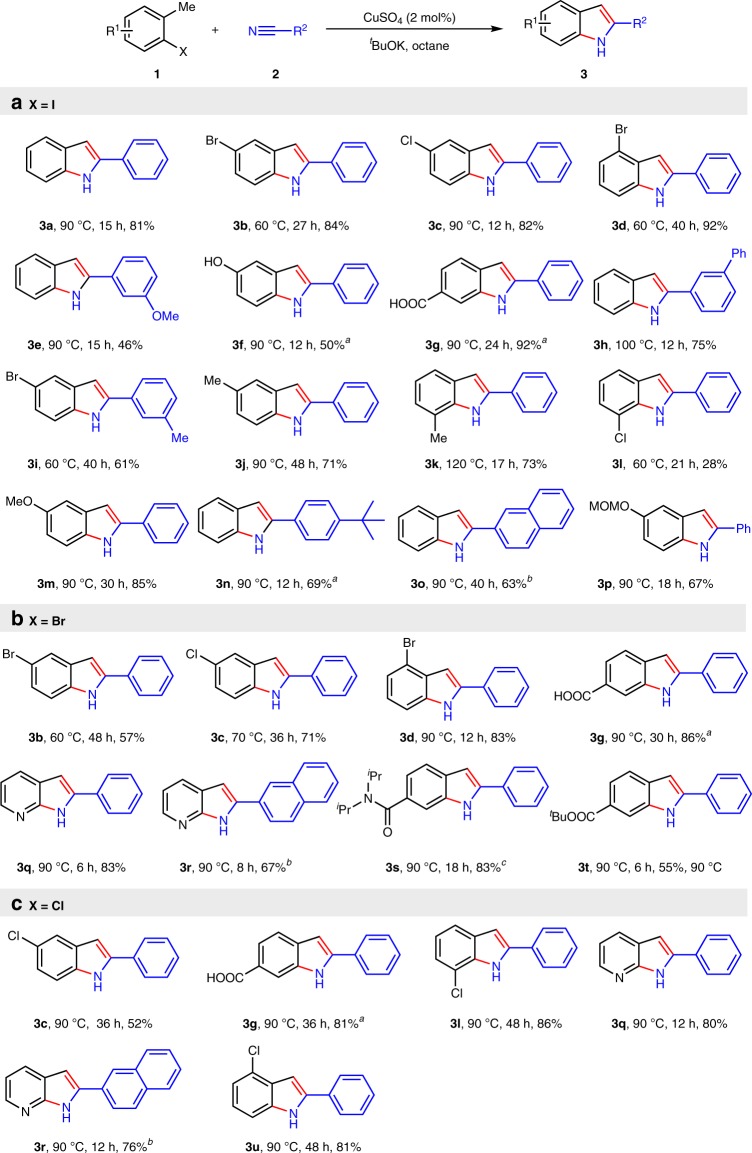
Scope of methyl (het)arenes and nitriles. Reaction conditions: CuSO_4_ (2 mol%), **1** (1 mmol), **2** (5 mmol), ^*t*^BuOK (5 mmol), octane (0.75 mL). Extra 1 mmol ^*t*^BuOK was used for **3f** and **3g**. ^a^**2** (1.5 mL) was used as solvent. ^b^Octane (3 mL). ^c^CuSO_4_ (20 mol %) was used. **a** 2-Iodomethyl arenes were subjected to the standard conditions. **b** 2-Bromomethyl arenes were subjected to the standard conditions. **c** 2-Chloromethyl arenes were subjected to the standard conditions

With the standard reaction conditions in hand, the scope of this method was investigated. Various 2-halotoluenes were subjected to the standard conditions and the corresponding indole products were obtained (Fig. 3). Halogen can survive under basic conditions (**3b**, **3c**, **3d**, **3i**, and **3l**). Such indoles are useful intermediates for further functionalization via cross coupling reactions. Starting materials with hydroxyl and carboxyl groups can directly undergo cyclization without protecting groups (**3f** and **3g**). 7-Azoindoles **3q** and **3r** could also be achieved by these reactions. Functional groups and protecting groups, such as halogen (**3b-d**, **3j**, **3l**, **3u**), (Ar)OH (**3f**), COOH (**3g**), MOM (**3p**), amide (**3l**), and ester (**3t**), are all tolerable in this reaction.

### Application in the synthesis of BACE1 inhibitor

**Fig. 4:**
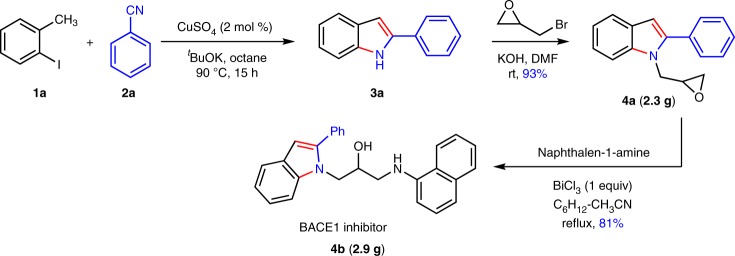
Gram-scale synthesis of pharmaceuticals**. a** BACE1 inhibitor is synthesized from 2-iodotoluene **1a** and benzonitrile **2a** in gram scale using anion-radical redox relay as the key step

This indoles synthesis from nitriles and toluenes is a synthetically practical and scalable method. By this method, a BACE1 inhibitor^68^ is synthesized from benzonitrile and 2-iodotoluene by our method in three steps in gram scale (Fig. 4).

### Investigations of C3-substituted indoles and applications

C3-substituted indoles can also be synthesized by this method. Reaction conditions were further optimized on the basis of Table 1 (Table 2). Despite of the fact that even 1.5 equiv of nitrile affords 85% of **6d** (entry 5), the conditions listed in entry 2 were chosen for the balance of yields and amounts of the base and nitriles.

**Table 2:** Reaction conditions for the synthesis of 6


Entry^a^	PhCN (equiv)	^*t*^BuOK (equiv)	*t* (h)	6d (%)
1^*b*^	5	4	11	93
2	2.5	2.5	24	92
3	2	2	36	75
4	2	3	24	91
5	1.5	3	24	85

^a^Conditions: **5d** (1 mmol), **2a** (1.5–5 mmol), CuSO_4_ (2 mol%), ^*t*^BuOK (2–5 mmol), dioxane (0.75 mL), argon. Yields were determined by ^1^H NMR

^b^At 60 °C

**Fig. 5:**
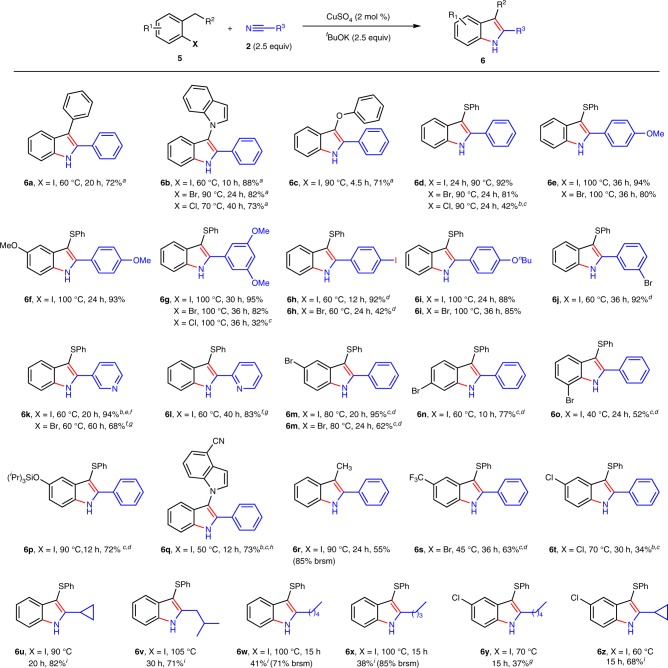
Scope of C3-functionalized indoles. Reaction conditions: **5** (1 mmol), **2** (2.5 mmol), CuSO_4_ (2 mol %), ^*t*^BuOK (2.5 mmol), dioxane (0.75 mL). ^a^**2** (5 mmol), ^*t*^BuOK (4 mmol), octane (0.75 mL) instead of dioxane. ^b^**2** (1.5 mL) was used as solvent. ^c^CuSO_4_ (10 mol %) was used. ^d^Dioxane (1.5 mL). ^e^**2** (15 mL) was used as solvent. ^f^CuSO_4_ (20 mol %) was used. ^g^Dioxane (20 mL). ^h*t*^BuOK (4 mmol). ^i*t*^BuOK (4 mmol), no extra solvent. Brsm yield refers to the yield based on recovered starting material

**Fig. 6:**
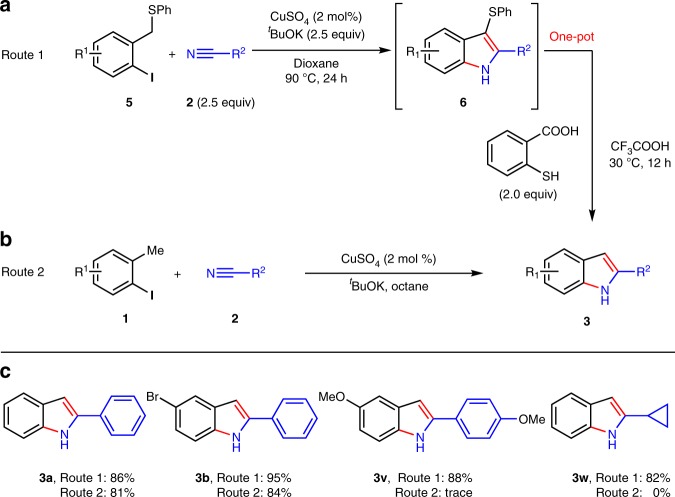
Comparison between the synthesis of **3** from **1** and that from **5**. **a** Synthesis of **3** using a one-pot cyclization-deprotection from **5**. **b** Synthesis of **3** from the cyclization of **1** and **2**. **c** Comparison between Route 1 and Route 2

Indoles bearing both C3-substituents, such as phenyl, heteroaryl, phenoxyl, and phenylthio, were readily obtained (Figs. 5, **6a–d**). These functionalized indoles are useful synthons or intermediates and are normally synthesized via C3-functionalization of indoles. For example, 2,3-diphenyl indole **6a** is a key intermediate of a BACE1 inhibitor^68^. What should be mentioned is that the method for the synthesis of 3-sulfenylindoles is still limited^69–73^. Current method provides a powerful route to 3-sulfenylindoles (**6d**-**6p**, **6s-6t**). Either aryl or alkyl substituted 3-sulfenylindoles could be achieved. C5-C7 bromo indoles are useful intermediates in organic synthesis, which were obtained in moderated to good yields (**6m**-**6o**). Plus the results in Fig. 4, most functional groups, such as halogen, OH, COOH, amide, ester, silyl protecting group, MOM, CF_3_, nitrile, pyridine, etc, have been tolerated in this reaction. Besides 2-halogen toluenes, 2-iodo ethyl benzene is also reactive to afford the desired 3-methyl indole **6r** in 55% yield. Alkyl substituted 3-sulfenylindoles are normally difficult to generate due to the instability of aliphatic nitriles under strong basic conditions. To our delight, C2-alkyl indoles could also be prepared in moderate to good yields (Fig. 6, **6u–6z**). The yields seem dependent on the steric hindrance of nitriles (**6u**, **6v** vs **6w**). Nevertheless, this reaction provides an efficient route to either aromatic or aliphatic substituted indoles.

C3-phenylthio can be easily removed in the presence of 2-mercaptobenzoic acid and CF_3_CO_2_H^74^. Thus PhS-group can be used as a leaving group in organic synthesis. In Fig. 6, a one-pot cyclization-deprotection synthesis of **3** either from **5** or **1** is demonstrated. Both indoles **3a** and **3b** were obtained in high yields via both routes, whereas indoles **3v** and **3w** could only be afforded by route 1 in high yields. The radical addition to nitrile is a nucleophilic process, thus the electron rich nitriles are normally less reactive. If the stability of radical intermediates are better, it gives more chance. Thio-groups are better stabilizer for adjacent carbon radicals. As a result, thio-substrate **5** is much better than **1** and some unavailable indoles from **1** and **2** can be achieved via **5**. Compound **3v** is a key intermediate for a potential anti-breast cancer medicine.

**Fig. 7:**
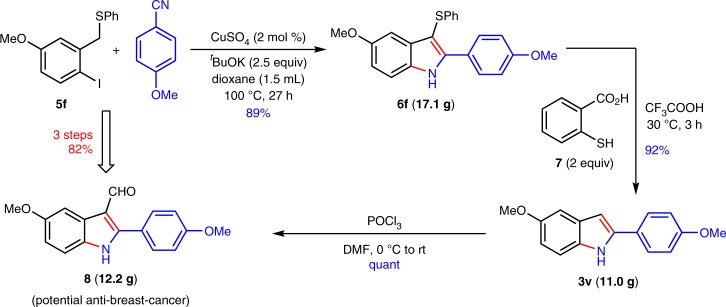
Large-scale synthesis of pharmaceutical. A potential anti-breast cancer reagent **8** is synthesized from **5f** in 10 gram-scale

A potential anti-breast cancer reagent **8** is synthesized through three steps from **5f** via the removal of the phenylthio in more than 10 gram-scale with overall 85% yield (Fig. 7)^75^.

### Preliminary investigations of reaction mechanism

**Fig. 8:**
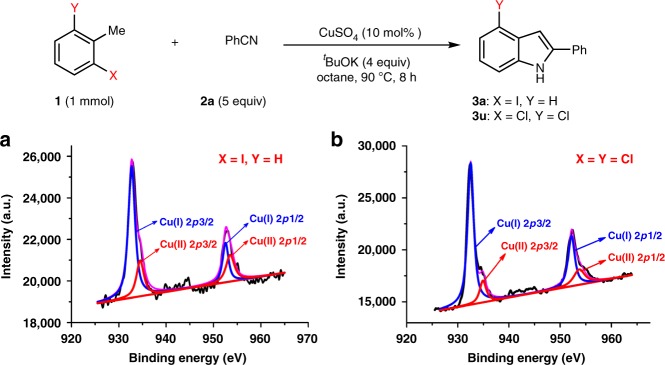
Detection of Cu(II) and Cu(I) by XPS experiments. **a** To **3a** when X = I and **b** to **3u** when X = Cl. XPS Instrument type: Thermo ESCALAB 250Xi; X-ray excitation source: monochromatic Al Ka (hv = 1486.6 eV), power 150 W, X-ray beam 500 μm; Energy analyzer fixed transmission energy: 30 eV

The reaction mechanism has been investigated with experimental evidence. In the reaction model in Fig. 2a, a Cu-catalyzed reaction cycle has been presented. The XPS experiments for the reaction using CuSO_4_ as catalyst demonstrated both Cu(II) and Cu(I), indicating that Cu-salts have been involved in the reaction (Fig. 8). The iodometric determination of Cu(II) in the reaction mixture was performed using a chloro-substrate to avoid the interruption of KI generated from the reaction. By the above titration, 65% Cu(II) was determined. Therefore the reaction could possibly pass through Fig. 1a via an iminyl radical addition to aryl rings.

## Discussion

In conclusion, we have developed a catalytic approach for the in situ generation of iminyl radicals via an intermolecular carbanion-radical redox relay using a naturally abundant copper salt as catalyst. This strategy is realized by combining a base-promoted C-H cleavage and CuSO_4_-catalyzed carbanion-radical redox relay, where CuSO_4_ is a cheap and naturally abundant inorganic salt and as low as only 2 mol% catalyst loading is needed. By avoiding using N-O/N-N homolysis or radical initiators to generate iminyl radicals, this reaction provides a practical and noble metal-free access to indoles. What should be mentioned is that this method directly affords *N*-H indoles without any *N*-protecting groups, avoiding the irremovable or hard removable protecting groups in organic synthesis. This method is also a synthetically practical method, which has been readily applied in the modification of the large-scale pharmaceutical synthesis from abundant feedstocks using cheap and “green” reagents (CuSO_4_ and ^*t*^BuOK).

## Methods

### General procedure

The Schlenk tube charged with ^*t*^BuOK (2.5–5 mmol) and CuSO_4_ (0.02 mmol, 3.2 mg) was dried under high vacuum for 15 min. Octane (0.75 mL), **1** or **5** (1 mmol), and **2** (2.5–5 mmol) were added under argon and stirred at 90 °C. The resulting reaction mixture was monitored by TLC. Upon completion of the starting materials, the reaction mixture was directly purified by silica gel column to give the desired product.

### Procedure for XPS experiments

A Schlenk tube charged with ^*t*^BuOK (4 mmol, 449 mg) and CuSO_4_ (0.1 mmol, 16 mg) was dried under high vacuum for 15 min. Octane (0.75 mL), **1** (1 mmol), and **2a** (5 mmol) were added under argon and stirred at 90 °C for 8 h. The mixture was concentrated under vacuum and the solid was measured by XPS tests.

### Iodometric determination of Cu(II) in the reaction mixture

A Schlenk tube charged with ^*t*^BuOK (4 mmol, 449 mg) and CuSO_4_ (0.1 mmol, 16 mg) was dried under high vacuum for 15 min. Octane (0.75 mL), **1u** (1 mmol) and **2a** (5 mmol) were added under argon and stirred at 90 °C. The reaction was stirred for 8 h and quenched by CH_2_Cl_2_ and extracted with H_2_O. The aqueous phase were combined. When the pH was adjusted to 7–8, KI (8 mmol) and 5 ml of 0.5 wt% starch solution were sequentially added. Sodium thiosulfate standard titration solution [c(Na_2_S_2_O_3_) = 0.05 mol/L] was used to titrate until the solution blue disappeared. Cu(II) was determined as 0.065 mmol (65% based on 10 mol% CuSO_4_).

## Supplementary information

Supplementary Information

Peer Review File

## Data Availability

The authors declare that the data supporting this study are available within the Article and its Supplementary Information files.
