# Supplementary material for: Copper-catalyzed oxidative benzylic C-H cyclization via iminyl radical from intermolecular anion-radical redox relay
Source: Nat Commun. 2019 Feb 22;10:908. doi: 10.1038/s41467-019-08849-z (PMC6385223; doi:10.1038/s41467-019-08849-z)
Supplement: Supplementary file 1 — Supplementary Information [file 41467_2019_8849_MOESM1_ESM.pdf]

Supplementary information

Copper-catalyzed oxidative benzylic C-H cyclization via iminyl radical from intermolecular anion-radical redox relay

Xiang-Huan Shan,[1] Hong-Xing Zheng,[1,3] Bo Yang,[1] Lin Tie,[1] Jia-Le Fu,[1] Jian-Ping Qu,[2,*] and Yan-Biao Kang[1,3*]

[1]Department of Chemistry, University of Science and Technology of China, Hefei, Anhui 230026, China

[2]Institute of Advanced Synthesis, School of Chemistry and Molecular Engineering, Jiangsu National Synergetic Innovation Center for Advanced Materials, Nanjing Tech University, Nanjing 211816, China

[3]State Key Laboratory of Organometallic Chemistry, Shanghai Institute of Organic Chemistry, CAS, 345 Lingling Road, Shanghai 200032, China

E-mail: *ybkang@ustc.edu.cn; ias_jpqu@njtech.edu.cn

Contents

Supplementary Methods 2

Supplementary Figures 26

Supplementary References 180

Supplementary Methods

General Information: Solvents were predried over activated 4 Å molecular sieves and further dried by refluxing and distilling over sodium (1,4-dioxane) or CaH_2 (toluene, octane, CH_3CN, CH_2Cl_2 and DMF) under argon atmosphere. ^{1}H, ^{13}C NMR spectra were recorded on a Bruker 400 spectrometer. Chemical shifts are reported in δ units relative to $CDCl_3$ [^{1}H δ = 7.26, ^{13}C δ = 77.16], DMSO-*d*$_6$ [^{1}H δ = 2.50, ^{13}C δ = 39.52].

Experiment Procedure for Table 1: To a Schlenk tube charged with $CuSO_4$ (0.02 mmol, 3.2 mg) and ^{t}BuOK (4 mmol, 449 mg) was added **1a** (1 mmol, 218 mg), **2a** (5 mmol, 0.51 mL) and solvent (0.75 mL) and the resulting reaction mixture was stirred at 90 ℃ (oil bath). The conversion was estimated by ^{1}H NMR spectrometer using dibenzyl ether (48 μL) as an internal standard.

General Procedure 1 for Synthesis of Indoles: The Schlenk tube charged with ^{t}BuOK (2.5-5 mmol) and $CuSO_4$ (0.02 mmol, 3.2 mg) was dried under high vacuum for 15 min. Octane (0.75 mL), **1** or **5** (1 mmol), and **2** (2.5-5 mmol) were added under argon and stirred at 90 ℃. The resulting reaction mixture was monitored by TLC. Upon completion of astarting materials, the reaction mixture was directly prified by silica gel column to give the desired product.

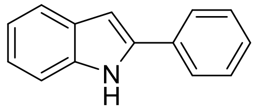

2-phenyl-1*H*-indole (3a)

This compound was prepared according to the general procedure, white solid, 156.5 mg, 81%. ^{1}H NMR (400 MHz, $CDCl_3$) δ 8.26 (s, 1H), 7.64–7.62 (m, 3H), 7.44–7.36 (m, 3H), 7.28–7.33 (m, 1H), 7.21–7.17 (m, 1H), 7.14–7.10 (m, 1H), 6.82 (dd, *J* = 2.0, 0.8 Hz, 1H). ^{13}C{^{1}H} NMR (100 MHz, $CDCl_3$) δ 138.0, 136.9, 132.5, 129.4, 129.1, 127.8, 125.3, 122.5, 120.8, 120.4, 111.0, 100.1.[10]

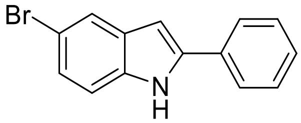

5-bromo-2-phenyl-1*H*-indole (3b)

This compound was prepared according to the general procedure, white solid, X = I, 228.4 mg, 84%. X = Br, 154.8 mg, 57%. ^{1}H NMR (400 MHz, $CDCl_3$) δ 8.34 (s, 1H), 7.74 (s, 1H), 7.63 (d, *J* = 7.2 Hz, 2H), 7.44 (t, *J* = 7.2 Hz, 2H), 7.34 (t, *J* = 7.2 Hz, 1H), 7.26 (s, 2H), 6.75 (s, 1H). ^{13}C{^{1}H} NMR (100 MHz, $CDCl_3$) δ 139.2, 135.5, 131.9, 131.1, 129.3, 128.3, 125.4, 125.2, 123.2, 113.5, 112.4, 99.6.[10]

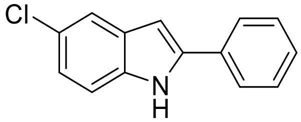

5-chloro-2-phenyl-1*H*-indole (3c)

This compound was prepared according to the general procedure, white solid, X = I, 186.8 mg, 82%. X = Br, 162.1 mg, 71%. X = Cl, 117.9 mg, 52%. ^{1}H NMR (400 MHz, $CDCl_3$) δ 8.32 (s, 1H), 7.64–7.62 (m, 2H), 7.58 (d, *J* = 2.0

Hz, 1H), 7.44 (t, *J* = 7.6 Hz, 2H), 7.32–7.34 (m, 1H), 7.29 (d, *J* = 8.8 Hz, 1H), 7.13 (dd, *J* = 8.4, 2.0 Hz, 1H), 6.75 (dd, *J* = 2.4, 0.8 Hz, 1H). $^{13}C\{^1H\}$ NMR (100 MHz, $CDCl_3$) δ 139.4, 135.2, 132.0, 130.4, 129.2, 128.3, 126.0, 125.4, 122.7, 120.1, 112.0, 99.7.[10]

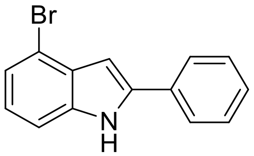

4-bromo-2-phenyl-1*H*-indole (3d)

This compound was prepared according to the general procedure, white solid, X = I, 250.6 mg, 92%. X = Br, 225.3 mg, 83%. ^1H NMR (400 MHz, $CDCl_3$) δ 8.35 (s, 1H), 7.61 (d, *J* = 8.8 Hz, 2H), 7.41 (t, *J* = 7.6 Hz, 2H), 7.34–7.26 (m, 3H), 7.01 (t, *J* = 8.0 Hz, 1H), 6.85 (d, *J* = 1.6 Hz, 1H). $^{13}C\{^1H\}$ NMR (100 MHz, $CDCl_3$) δ 138.6, 137.0, 131.7, 130.1, 129.2, 128.3, 125.3, 123.2, 114.6, 110.2, 100.2.[11]

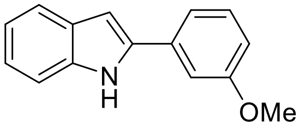

2-(3-methoxyphenyl)-1*H*-indole (3e)

This compound was prepared according to the general procedure, white solid, 102.7 mg, 46%. **^{1}H** NMR (400 MHz, $CDCl_3$) δ 8.27 (s, 1H), 7.62 (d, *J* = 8.4 Hz, 1H), 7.36–7.30 (m, 2H), 7.21–7.16 (m, 3H), 7.09–7.13 (m, 1H), 6.85 (ddd, *J* = 8.4, 2.4, 0.8 Hz, 1H), 6.80 (dd, *J* = 2.0, 0.8 Hz, 1H), 3.84 (s, 3H). $^{13}C\{^1H\}$ NMR (100 MHz, $CDCl_3$) δ 160.1, 137.9, 136.9, 133.8, 130.2, 129.3, 122.5, 120.8, 120.4, 117.8, 113.2, 111.1 (two peaks), 100.3, 55.5.[12]

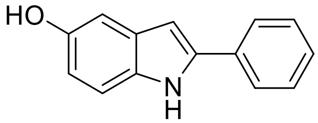

2-phenyl-1*H*-indol-5-ol (3f)

^{t}BuOK (1 mmol, 112 mg) and **1f** (1 mmol, 234 mg) were weighed directly into a Schlenk tube. Then toluene (2 mL) was added under argon and stirred. After stirring for 0.5 h at rt. Then concentrated under vacuum. Then ^{t}BuOK (4 mmol, 449 mg) and $CuSO_4$ (0.02 mmol, 3.2 mg) were weighed directly into a Schlenk tube and dried under high vacuum for 15 min. Then **2a** (1.5 mL) was added under argon and stirred at 90 ℃ and the reaction was monitored by TLC. The reaction was quenched by H_2O (10 mL)/1M HCl (4 mL) and extracted with EA. The combined organic extracts were washed with brine, dried over anhydrous Na_2SO_4, filtered, concentrated and purified on silica gel chromatography (EtOAc/petroleum ether as eluent) to obtain **3f** as a white solid, 104.5 mg, 50%. ^1H NMR (400 MHz, DMSO–d_6) δ 11.23 (s, 1H), 8.71 (s, 1H), 7.81 (d, *J* = 7.6 Hz, 2H), 7.43 (t, *J* = 7.6 Hz, 2H), 7.28 (t, *J* = 7.6 Hz, 1H), 7.20 (d, *J* = 8.8 Hz, 1H), 6.85 (d, *J* = 2.0 Hz, 1H), 6.72 (d, *J* = 1.2 Hz, 1H), 6.63 (dd, *J* = 8.8, 2.4 Hz, 1H). $^{13}C\{^1H\}$ NMR (100 MHz, DMSO–d_6) δ 150.9, 137.9, 132.5, 131.7, 129.4, 128.9, 127.2, 124.8, 112.0, 116.7, 111.7, 98.0.[13]

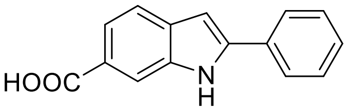

2-phenyl-1*H*-indole-6-carboxylicacid (3g)

^{t}BuOK (1 mmol, 112 mg) and **1g** (1 mmol, 262 mg) were weighed directly into a Schlenk tube. Then tolueue (2 mL) was added under argon and stirred. After stirring for 0.5 h at rt. Then concentrated under vacuum. Then ^{t}BuOK (4 mmol, 449 mg) and $CuSO_4$ (0.02 mmol, 3.2 mg) were weighed directly into a Schlenk tube and dried under high vacuum for 15 min. Then **2a** (1.5 mL) was added under argon and stirred at 90 ℃ and the reaction was monitored by TLC. The reaction was quenched by H_2O (10 mL)/1M HCl (4 mL) and extracted with EA. The combined organic extracts were washed with brine, dried over anhydrous Na_2SO_4, filtered, concentrated and purified on silica gel chromatography (EtOAc/petroleum ether as eluent) to obtain **3g** as a white solid, X = I, 218.3 mg, 92%. X = Br, 204.3 mg, 86%. X = Cl, 191.7 mg, 81%. ^{1}H NMR (400 MHz, DMSO–d_6) δ 12.62 (s, 1H), 11.94 (s, 1H), 8.12 (s, 1H), 7.93 (d, *J* = 7.2 Hz, 2H), 7.68 (d, *J* = 7.6 Hz, 1H), 7.62 (d, *J* = 8.4 Hz, 1H), 7.51 (t, *J* = 7.6 Hz, 2H), 7.39 (t, *J* = 7.6 Hz, 1H), 7.01(s, 1H). ^{13}C{^{1}H} NMR (100 MHz, DMSO–d_6) δ 168.4, 141.1, 136.5, 132.1, 131.6, 129.1, 128.2, 125.5, 123.6, 120.5, 119.7, 113.3, 99.1.[14]

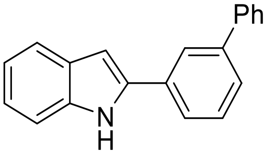

2-([1,1'-biphenyl]-3-yl)-1*H*-indole (3h)

This compound was prepared according to the general procedure, white solid, 202 mg, 75%. ^{1}H NMR (400 MHz, $CDCl_3$) δ 8.38 (s, 1H), 7.86 (s, 1H), 7.66–7.63 (m, 4H), 7.55–7.46 (m, 4H), 7.42–7.37 (m, 2H), 7.21 (t, *J* = 8.0 Hz, 1H), 7.13 (t, *J* = 7.2 Hz, 1H), 6.89 (d, *J* = 1.2 Hz, 1H). ^{13}C{^{1}H} NMR (100 MHz, $CDCl_3$) δ 142.2, 141.0, 137.9, 137.0, 133.0, 129.6, 129.4, 129.0, 127.8, 127.4, 126.7, 124.2, 122.6, 120.8, 120.5, 111.1, 100.4. HRMS (ESI–TOF) m/z: $[M+H]^+$ calcd for $C_{20}H_{16}N$ 270.1277; found 270.1275.

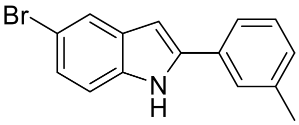

5-bromo-2-(m-tolyl)-1*H*-indole (3i)

This compound was prepared according to the general procedure, white solid, 174.6 mg, 61%. ^{1}H NMR (400 MHz, $CDCl_3$) δ 8.34 (s, 1H), 7.73 (s, 1H), 7.46–7.42 (m, 2H), 7.33 (t, *J* = 7.6 Hz, 1H), 7.25 (s, 2H), 7.16 (d, *J* = 7.6 Hz, 1H), 6.73 (d, *J* = 2.0 Hz, 1H), 2.42 (s, 3H). ^{13}C{^{1}H} NMR (100 MHz, $CDCl_3$) δ 139.4, 138.9, 135.4, 131.8, 131.1, 129.1 (two peaks), 126.1, 125.1, 123.1, 122.5, 113.5, 112.4, 99.4, 21.7.[15]

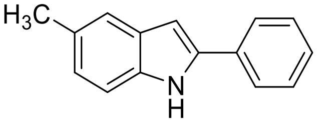

5-methyl-2-phenyl-1*H*-indole (3j)

This compound was prepared according to the general procedure, white solid, 147.2 mg, 71%. ^{1}H NMR (400 MHz, $CDCl_3$) δ 8.18 (s, 1H), 7.62 (d, *J* = 7.2 Hz, 2H), 7.43–7.40 (m, 3H), 7.31–7.22 (m, 2H), 7.01 (d, *J* = 8.4 Hz, 1H), 6.73

(s, 1H), 2.44 (s, 3H). $^{13}C\{^1H\}$ NMR (100 MHz, $CDCl_3$) δ 138.1, 135.3, 132.6, 129.7, 129.6, 129.1, 127.7, 125.2, 124.1, 120.4, 110.7, 99.7, 21.6.[10]

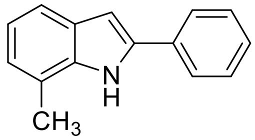

7-methyl-2-phenyl-1*H*-indole (3k)

This compound was prepared according to the general procedure, white solid, 151.3 mg, 73%. ^1H NMR (400 MHz, $CDCl_3$) δ 8.18 (s, 1H), 7.69–7.66 (m, 2H), 7.49–7.41 (m, 3H), 7.31 (t, J = 7.2 Hz, 1H), 7.06–6.98 (m, 2H), 6.82 (d, J = 2.0 Hz, 1H), 2.53 (s, 3H). $^{13}C\{^1H\}$ NMR (100 MHz, $CDCl_3$) δ 137.7, 136.5, 132.6, 129.1, 128.9, 127.8, 125.3, 123.1, 120.6, 120.2, 118.5, 100.7, 16.9.[10]

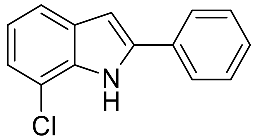

7-chloro-2-phenyl-1*H*-indole (3l)

This compound was prepared according to the general procedure, white solid, X = I, 63.8 mg, 28%. X = Cl, 195.6 mg, 86%. ^1H NMR (400 MHz, $CDCl_3$) δ 8.42 (s, 1H), 7.64–7.62 (m, 2H), 7.49 (d, J = 7.6 Hz, 1H), 7.41 (t, J = 7.6 Hz, 2H), 7.33–7.29 (m, 1H), 7.18–7.15 (m, 1H), 7.04–6.99 (m, 1H), 6.80 (d, J = 2.4 Hz, 1H). $^{13}C\{^1H\}$ NMR (100 MHz, $CDCl_3$) δ 138.7, 134.1, 131.8, 130.7, 129.2, 128.2, 125.4, 121.7, 121.1, 119.3, 116.4, 100.9.[11]

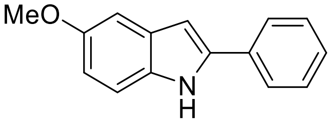

5-methoxy-2-phenyl-1*H*-indole (3m)

This compound was prepared according to the general procedure, white solid, 190.3 mg, 85%. ^1H NMR (400 MHz, $CDCl_3$) δ 8.24 (s, 1H), 7.63 (d, J = 7.2 Hz, 2H), 7.42 (t, J = 7.6 Hz, 2H), 7.32–7.24 (m, 2H), 7.09 (d, J = 2.4 Hz, 1H), 6.85 (dd, J = 8.8, 2.4 Hz, 1H), 6.75 (d, J = 1.6 Hz, 1H), 3.86 (s, 3H). $^{13}C\{^1H\}$ NMR (100 MHz, $CDCl_3$) δ 154.6, 138.7, 132.5, 132.1, 129.8, 129.1, 127.8, 125.2, 112.7, 111.8, 102.3, 99.9, 56.0.[11]

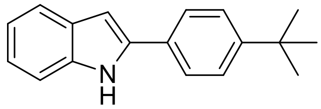

2-(4-(tert-butyl)phenyl)-1*H*-indole (3n)

This compound was prepared according to the general procedure, yellow solid, 172 mg, 69%. ^1H NMR (400 MHz, $CDCl_3$) δ 8.30 (s, 1H), 7.63–7.58 (m, 3H), 7.48–7.45 (m, 2H), 7.41–7.38 (m, 1H), 7.20–7.16 (m, 1H), 7.13–7.09 (m, 1H), 6.79 (dd, J = 2.0, 0.8 Hz, 1H), 1.36 (s, 9H). $^{13}C\{^1H\}$ NMR (100 MHz, $CDCl_3$) δ 151.0, 138.1, 136.8, 129.7, 129.5, 126.1, 125.0, 122.2, 120.7, 120.3, 111.0, 99.6, 34.8, 31.4.[11]

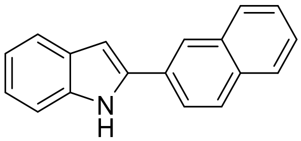

2-(naphthalene-2-yl)-1*H*-indole (3o)

This compound was prepared according to the general procedure, white solid, 153.3 mg, 63%. ^{1}H NMR (400 MHz, $CDCl_3$) δ 8.42 (s, 1H), 8.02 (s, 1H), 7.89–7.78 (m, 4H), 7.65 (d, *J* = 7.8 Hz, 1H), 7.52–7.45 (m, 2H), 7.41 (dd, *J* = 8.0, 0.8 Hz, 1H), 7.23–7.19 (m, 1H), 7.16–7.12 (m, 1H), 6.94 (dd, *J* = 2.0, 0.8 Hz, 1H). ^{13}C{^{1}H} NMR (100 MHz, $CDCl_3$) δ 138.0, 137.1, 133.7, 133.0, 129.8, 129.5, 128.9, 128.1, 127.9, 126.8, 126.3, 123.9, 123.1, 122.7, 120.9, 120.5, 111.1, 100.8.[11]

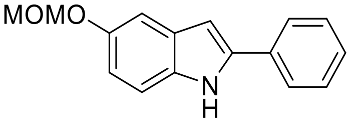

5-(methoxymethoxy)-2-phenyl-1*H*-indole (3p)

This compound was prepared according to the general procedure, white solid, 168.7 mg, 67%. ^{1}H NMR (400 MHz, $CDCl_3$) δ 8.26 (s, 1H), 7.63–7.61 (m, 2H), 7.44–7.39 (m, 2H), 7.33–7.24 (m, 3H), 6.93 (dd, *J* = 8.8, 2.4 Hz, 1H), 6.74 (dd, *J* = 2.0, 0.8 Hz, 1H), 5.21 (s, 2H), 3.53 (s, 3H). ^{13}C{^{1}H} NMR (100 MHz, $CDCl_3$) δ 152.0, 139.0, 133.0, 132.5, 129.9, 129.1, 127.8, 125.2, 114.0, 111.6, 106.9, 100.1, 95.9, 56.0. HRMS (ESI–TOF) m/z: $[M+H]^+$ calcd for $C_{16}H_{16}NO_2$ 254.1176; found 254.1173.

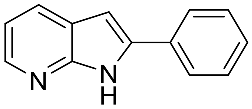

2-phenyl-1*H*-pyrrolo[2,3-*b*]pyridine (3q)

This compound was prepared according to the general procedure, white solid, X = Br, 161.8 mg, 83%. X = Cl, 155.3 mg, 80%. ^{1}H NMR (400 MHz, $CDCl_3$) δ 12.95 (s, 1H), 8.30 (d, *J* = 3.2 Hz, 1H), 7.98–7.91 (m, 3H), 7.53 (t, *J* = 7.2 Hz, 2H), 7.40 (t, *J* = 6.8 Hz, 1H), 7.11 (dd, *J*= 7.2, 4.8 Hz, 1H), 6.79 (s, 1H). ^{13}C{^{1}H} NMR (100 MHz, $CDCl_3$) δ 150.2, 142.2, 139.8, 132.6, 129.2, 128.9, 128.3, 126.1, 122.5, 116.2, 97.5.[17]

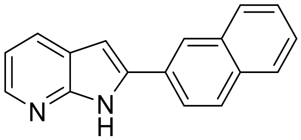

2-(naphthalen-2-yl)-1*H*-pyrrolo[2,3-*b*]pyridine (3r)

This compound was prepared according to the general procedure, white solid, X = Br, 163.6 mg, 67%. X = Cl, 186.1 mg, 76%. ^{1}H NMR (400 MHz, DMSO–d_6) δ 12.29 (s, 1H), 8.53 (d, *J* = 1.6 Hz, 1H), 8.25 (dd, *J* = 4.4, 1.2 Hz, 1H), 8.10 (dd, *J* = 8.4, 2.0 Hz, 1H), 8.01–7.93 (m, 4H), 7.59–7.51 (m, 2H), 7.10–7.07 (m, 2H). ^{13}C{^{1}H} NMR (100 MHz, DMSO–d_6) δ 149.8, 143.0, 138.2, 133.1, 132.5, 129.0, 128.4, 128.0, 127.9, 127.7, 126.7, 126.3, 123.7 (two peaks), 121.0, 116.1, 97.9. HRMS (ESI–TOF) m/z: $[M+H]^+$ calcd for $C_{17}H_{13}N_2$ 245.1073; found 245.1078.

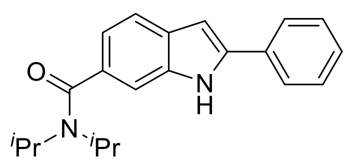

N,N-diisopropyl-2-phenyl-1H-indole-6-carboxamide (3s)

This compound was prepared according to the general procedure, yellow solid, 265.7 mg, 83%. ^1H NMR (400 MHz, $CDCl_3$) δ 10.03 (s, 1H), 7.70 (d, *J* = 7.2 Hz, 2H), 7.48 (s, 1H), 7.44 (d, *J* = 8.0 Hz, 1H), 7.37 (t, *J* = 7.2 Hz, 2H), 7.27 (t, *J* = 7.2 Hz, 1H), 6.91 (dd, *J* = 8.0, 1.2 Hz, 1H), 6.70 (d, *J* = 1.6 Hz, 1H), 3.97 (brs, 1H), 3.60 (brs, 1H), 1.54 (brs, 6H), 1.25 (brs, 6H). $^{13}C\{^1H\}$ NMR (100 MHz, $CDCl_3$) δ 172.9, 139.8, 136.8, 132.5, 132.0, 129.5, 128.9, 127.6, 125.6, 119.9, 117.2, 110.1, 99.2, 21.0. HRMS (ESI–TOF) m/z: $[M+H]^+$ calcd for $C_{21}H_{25}N_2O$ 321.1961; found 321.1959.

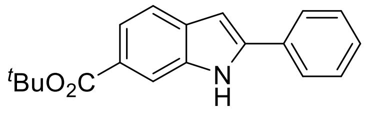

tert-butyl 2-phenyl-1H-indole-6-carboxylate (3t)

This compound was prepared according to the general procedure, yellow solid, 161.6 mg, 55%. ^1H NMR (400 MHz, $CDCl_3$) δ 8.84 (s, 1H), 8.16 (s, 1H), 7.78 (dd, *J* = 8.0, 1.6 Hz, 1H), 7.73-7.71 (m, 2H), 7.62 (d, *J* = 8.4 Hz, 1H), 7.46 (t, *J* = 7.6 Hz, 2H), 7.36 (tt, *J* = 7.2, 1.2 Hz, 1H), 6.86 (dd, *J* = 2.0, 0.8 Hz, 1H), 1.63 (s, 9H). $^{13}C\{^1H\}$ NMR (100 MHz, $CDCl_3$) δ 167.1, 141.1, 136.3, 132.7, 131.9, 129.2, 128.4, 125.8, 125.6, 121.4, 120.0, 113.2, 100.2, 80.7, 28.4. HRMS (ESI–TOF) m/z: $[M+H]^+$ calcd for $C_{19}H_{20}NO_2$ 294.1489; found 294.1488.

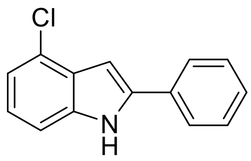

4-chloro-2-phenyl-1H-indole (3u)

This compound was prepared according to the general procedure, yellow solid, 183.8 mg, 81%. ^1H NMR (400 MHz, $CDCl_3$) δ 8.40 (s, 1H), 7.67–7.65 (m, 2H), 7.48–7.45 (m, 2H), 7.37 (tt, *J* = 7.2, 1.2 Hz, 1H), 7.28 (d, *J* = 7.6 Hz, 1H), 7.18–7.10 (m, 2H), 6.95 (dd, *J* = 2.4, 0.8 Hz, 1H). $^{13}C\{^1H\}$ NMR (100 MHz, $CDCl_3$) δ 138.6, 137.4, 131.8, 129.2, 128.3, 128.2, 125.9, 125.3, 122.9, 120.1, 109.6, 98.5.[18]

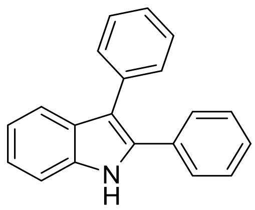

2,3-diphenyl-1*H*-indole (6a)

This compound was prepared according to the general procedure, colorless oil, 193.4 mg, 72%. ^1H NMR (400 MHz, $CDCl_3$) δ 8.13 (s, 1H), 7.68 (d, *J* = 8.0 Hz, 1H), 7.44–7.34 (m, 7H), 7.32–7.19 (m, 5H), 7.16–7.12 (m, 1H). $^{13}C\{^1H\}$ NMR (100 MHz, $CDCl_3$) δ 136.0, 135.2, 134.2, 132.8, 130.3, 128.9, 128.8, 128.7, 128.3, 127.8, 126.4, 122.8, 120.6, 119.8, 115.1, 111.0.[11]

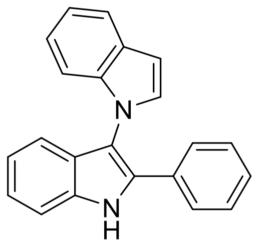

2'-phenyl-1'*H*-1,3'-biindole (6b)

This compound was prepared according to the general procedure, yellow oil, X = I, 271.0 mg, 88%. X = Br, 253.5 mg, 82%. X= Cl, 225.4 mg, 73%. ^{1}H NMR (400 MHz, $CDCl_3$) δ 8.37 (s, 1H), 7.72 (d, *J* = 7.6 Hz, 1H), 7.46 (d, *J* = 7.6 Hz, 1H), 7.30–7.22 (m, 5H), 7.18–7.08 (m, 7H), 6.70 (d, *J* = 3.2 Hz, 1H). ^{13}C{^{1}H} NMR (100 MHz, $CDCl_3$) δ 137.5, 134.3, 132.4, 130.5, 129.7, 129.1, 128.8, 128.3, 126.6, 126.3, 123.5, 122.1, 121.0, 120.8, 120.1, 118.7, 113.5, 111.4, 111.2, 103.3. HRMS (ESI–TOF) m/z: $[M+H]^+$ calcd for $C_{22}H_{17}N_2$ 309.1386; found 309.1387.

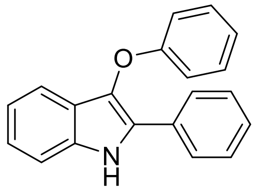

3-phenoxy-2-phenyl-1*H*-indole (6c)

This compound was prepared according to the general procedure, yellow solid, 201.5 mg, 71%. ^{1}H NMR (400 MHz, $CDCl_3$) δ 7.99 (s, 1H), 7.73 (d, *J* = 7.6 Hz, 2H), 7.38 (t, *J* = 8.0 Hz, 3H), 7.29–7.18 (m, 5H), 7.06–6.98 (m, 4H). ^{13}C{^{1}H} NMR (100 MHz, $CDCl_3$) δ 158.6, 134.0, 131.2, 130.8, 129.7, 129.1, 127.7, 126.2, 125.8, 123.3, 122.5, 122.1, 120.2, 118.6, 115.9, 111.5. HRMS (ESI–TOF) m/z: $[M+H]^+$ calcd for $C_{20}H_{16}NO$ 286.1226; found 286.1220.

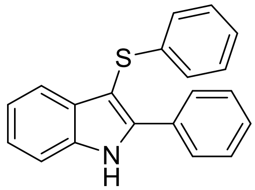

2-phenyl-3-(phenylthio)-1*H*-indole (6d)

This compound was prepared according to the general procedure, yellow oil, X = I, 276.8 mg, 92%. X = Br, 244.8 mg, 81%. X = Cl, 126.3 mg, 42%. ^{1}H NMR (400 MHz, $CDCl_3$) δ 8.47 (s, 1H), 7.72 (d, *J* = 7.2 Hz, 2H), 7.63 (d, *J* = 8.0 Hz, 1H), 7.42–7.34 (m, 4H), 7.26 (t, *J* = 7.2 Hz, 1H), 7.18–7.02 (m, 6H). ^{13}C{^{1}H} NMR (100 MHz, $CDCl_3$) δ 142.2, 139.3, 135.9, 131.5, 131.3, 128.9 (two peaks), 128.8, 128.2, 125.6, 124.7, 123.5, 121.3, 120.1, 111.3, 99.4.[19]

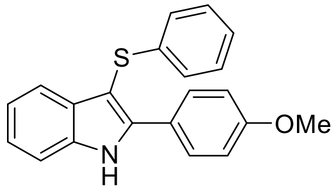

2-(4-methoxyphenyl)-3-(phenylthio)-1*H*-indole (6e)

This compound was prepared according to the general procedure, yellow oil, X = I, 310.5 mg, 94%. X = Br, 265.7 mg, 80%. ^{1}H NMR (400 MHz, $CDCl_3$) δ 8.44 (s, 1H), 7.67–7.64 (m, 2H), 7.61 (d, *J* = 8.0 Hz, 1H), 7.40 (d, *J* = 8.0 Hz, 1H), 7.26–7.22 (m, 1H), 7.17–7.13 (m, 3H), 7.10–7.07 (m, 2H), 7.05–7.02 (m, 1H), 6.95–6.91 (m, 2H), 3.80 (s,

3H). $^{13}C\{^1H\}$ NMR (100 MHz, $CDCl_3$) δ 160.1, 142.3, 139.5, 135.8, 131.4, 129.5, 128.9, 125.5, 124.7, 124.0, 123.2, 121.2, 119.8, 114.4, 111.2, 98.3, 55.5.[19]

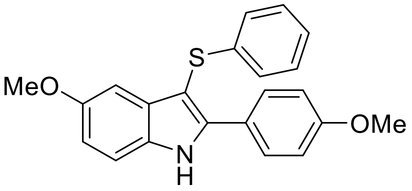

5-methoxy-2-(4-methoxyphenyl)-3-(phenylthio)-1*H*-indole (6f)

This compound was prepared according to the general procedure, yellow oil, 343.4 mg, 95%. ^1H NMR (400 MHz, $CDCl_3$) δ 8.40 (s, 1H), 7.64–7.62 (m, 2H), 7.28 (d, *J* = 8.4 Hz, 1H), 7.18–7.14 (m, 2H), 7.10–7.02 (m, 4H), 6.92–6.87 (m, 3H), 3.79 (s, 3H), 3.77 (s, 3H). $^{13}C\{^1H\}$ NMR (100 MHz, $CDCl_3$) δ 160.0, 155.3, 142.9, 139.6, 132.3, 130.6, 129.4, 128.9, 125.4, 124.6, 124.1, 114.3, 113.4, 112.0, 101.1, 97.9, 55.9, 55.4. HRMS (ESI–TOF) m/z: $[M+Na]^+$ calcd for $C_{22}H_{19}NNaO_2S$ 384.1029; found 384.1034.

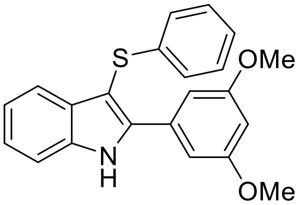

2-(3,5-dimethoxyphenyl)-3-(phenylthio)-1*H*-indole (6g)

This compound was prepared according to the general procedure, yellow oil, X = I, 342.7 mg, 95%. X = Br, 295.7 mg, 82%. X = Cl, 114.9 mg, 32%. ^1H NMR (400 MHz, $CDCl_3$) δ 8.57 (s, 1H), 7.67 (d, *J* = 8.0 Hz, 1H), 7.40 (d, *J* = 8.0 Hz, 1H), 7.26 (td, *J* = 8.0, 1.2 Hz, 1H), 7.19–7.07 (m, 5H), 7.04–7.00 (m, 1H), 6.86 (d, *J* = 2.4 Hz, 2H), 6.45 (t, *J* = 2.0 Hz, 1H), 3.66 (s, 6H). $^{13}C\{^1H\}$ NMR (100 MHz, $CDCl_3$) δ 161.0, 142.0, 139.7, 135.7, 133.1, 131.6, 128.9, 125.6, 124.7, 123.6, 121.3, 120.0, 111.3, 106.2, 101.2, 99.5, 55.4. HRMS (ESI–TOF) m/z: $[M+H]^+$ calcd for $C_{22}H_{20}NO_2S$ 362.1209; found 362.1209.

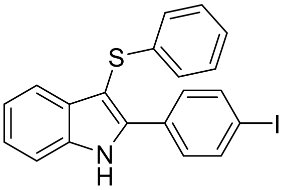

2-(4-iodophenyl)-3-(phenylthio)-1*H*-indole (6h)

This compound was prepared according to the general procedure, yellow solid, X = I, 392.2 mg, 92%. X = Br, 180.7 mg, 42%. ^1H NMR (400 MHz, $CDCl_3$) δ 8.44 (s, 1H), 7.71 (d, *J* = 8.4 Hz, 2H), 7.62(d, *J* = 8.0 Hz, 1H), 7.43 (d, *J* = 8.4 Hz, 2H), 7.40 (d, *J* = 8.0 Hz, 1H), 7.26 (t, *J* = 7.6 Hz, 1H), 7.17–7.12 (m, 3H), 7.06–7.02 (m, 3H). $^{13}C\{^1H\}$ NMR (100 MHz, $CDCl_3$) δ 140.9, 138.9, 138.0, 136.0, 131.2, 130.8, 129.7, 129.0, 125.7, 124.9, 123.8, 121.5, 120.2, 111.4, 100.2, 94.9. HRMS (ESI–TOF) m/z: $[M+H]^+$ calcd for $C_{20}H_{15}INS$ 427.9964; found 427.9952.

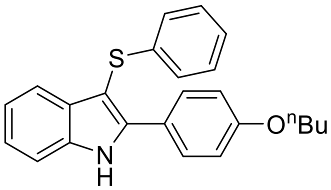

2-(4-butoxyphenyl)-3-(phenylthio)-1*H*-indole (6i)

This compound was prepared according to the general procedure, yellow oil, X = I, 328.7 mg, 88%. X = Br, 317.2 mg, 85%. ^{1}H NMR (400 MHz, $CDCl_3$) δ 8.41 (s, 1H), 7.64–7.59 (m, 3H), 7.37 (dt, *J* = 8.0, 0.8 Hz, 1H), 7.24–7.20 (m, 1H), 7.16–7.07 (m, 5H), 7.04–7.00 (m, 1H), 6.92–6.88 (m, 2H), 3.94 (t, *J* = 6.4 Hz, 2H), 1.78–1.71 (m, 2H), 1.52–1.42 (m, 2H), 0.96 (t, *J* = 7.2 Hz, 3H). ^{13}C{^{1}H} NMR (100 MHz, $CDCl_3$) δ 159.7, 142.4, 139.6, 135.8, 131.4, 129.5, 128.9, 125.6, 124.7, 123.7, 123.1, 121.2, 119.8, 114.9, 111.1, 98.3, 67.9, 31.4, 19.3, 14.0. HRMS (ESI–TOF) m/z: $[M+H]^+$ calcd for $C_{24}H_{24}NOS$ 374.1573; found 374.1563.

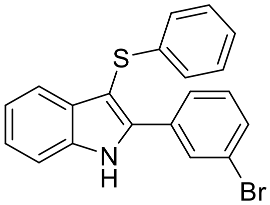

2-(3-bromophenyl)-3-(phenylthio)-1*H*-indole (6j)

This compound was prepared according to the general procedure, yellow oil, 350.6 mg, 92%. ^{1}H NMR (400 MHz, $CDCl_3$) δ 8.47 (s, 1H), 7.85 (t, *J* = 1.6 Hz, 1H), 7.71–7.68 (m, 1H), 7.63 (d, *J* = 8.4 Hz, 1H), 7.48 (ddd, *J* = 8.0, 1.6, 1.2 Hz, 1H), 7.42 (d, *J* = 8.0 Hz, 1H), 7.30–7.24 (m, 2H), 7.19–7.13 (m, 3H), 7.09–7.02 (m, 3H). ^{13}C{^{1}H} NMR (100 MHz, $CDCl_3$) δ 140.2, 138.9, 136.0, 133.5, 131.7, 131.2, 130.9, 130.4, 129.0, 127.0, 125.9, 125.0, 123.9, 122.9, 121.5, 120.3, 111.4, 100.8. HRMS (ESI–TOF) m/z: $[M+H]^+$ calcd for $C_{20}H_{15}BrNS$ 380.0103; found 380.0092.

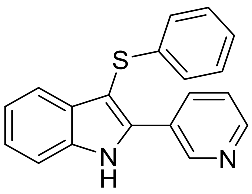

3-(phenylthio)-2-(pyridine-3-yl)-1*H*-indole (6k)

This compound was prepared according to the general procedure, white solid, X = I, 284.3 mg, 94%. X = Br, 205.1 mg, 68%. ^{1}H NMR (400 MHz, DMSO–d_6) δ 12.30 (s, 1H), 9.02 (d, *J* = 1.6 Hz, 1H), 8.60 (dd, *J* = 4.8, 1.6 Hz, 1H), 8.21 (dt, *J* = 8.0, 2.0 Hz, 1H), 7.57–7.52 (m, 2H), 7.48 (d, *J* = 8.0 Hz, 1H), 7.29–7.20 (m, 3H), 7.15–7.06 (m, 2H), 7.03–7.01(m, 2H). ^{13}C{^{1}H} NMR (100 MHz, DMSO–d_6) δ 149.2, 148.7, 139.2, 138.6, 136.5, 135.4, 130.3, 129.1, 127.2, 125.0, 123.6, 123.2, 120.8, 118.8, 112.2, 97.7. HRMS (ESI–TOF) m/z: $[M+H]^+$ calcd for $C_{19}H_{15}N_2S$ 303.0950; found 303.0948.

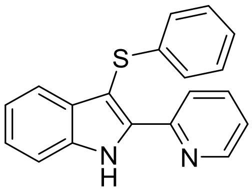

3-(phenylthio)-2-(pyridine-2-yl)-1*H*-indole (6l)

This compound was prepared according to the general procedure, white solid, 251.3 mg, 83%. ^{1}H NMR (400 MHz, $CDCl_3$) δ 10.53 (s, 1H), 8.62 (d, *J* = 8.0 Hz, 1H), 8.52 (dd, *J* = 4.8, 0.8 Hz, 1H), 7.60–7.56 (m, 2H), 7.28 (d, *J* = 8.0 Hz, 1H), 7.17–7.02 (m, 7H), 6.97–6.93 (m, 1H). ^{13}C{^{1}H} NMR (100 MHz, $CDCl_3$) δ 149.4, 149.0, 139.1, 138.4, 137.1, 135.6, 132.0, 129.0, 125.8, 125.0, 124.2, 123.0, 122.7, 121.1, 120.3, 111.7, 100.3. HRMS (ESI–TOF) m/z: $[M+H]^+$ calcd for $C_{19}H_{15}N_2S$ 303.0950; found 303.0958.

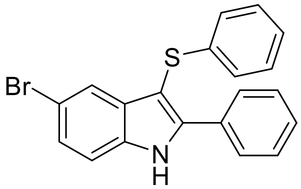

5-bromo-2-phenyl-3-(phenylthio)-1*H*-indole (6m)

This compound was prepared according to the general procedure, yellow oil, X = I, 361.3 mg, 95%. X = Br, 236.2 mg, 62%. ^{1}H NMR (400 MHz, $CDCl_3$) δ 8.58 (s, 1H), 7.76 (d, *J* = 1.6 Hz, 1H), 7.71 (dd, *J* = 8.0, 1.6 Hz, 2H), 7.43–7.36 (m, 3H), 7.35–7.27 (m, 2H), 7.17 (t, *J* = 7.6 Hz, 2H), 7.08–7.04 (m, 3H). ^{13}C{^{1}H} NMR (100 MHz, $CDCl_3$) δ 143.4, 138.8, 134.5, 133.2, 130.9, 129.2, 129.1, 129.0, 128.2, 126.4, 125.6, 125.0, 122.5, 114.7, 112.8, 99.1. HRMS (ESI–TOF) m/z: $[M+H]^+$ calcd for $C_{20}H_{15}BrNS$ 380.0103; found 380.0087

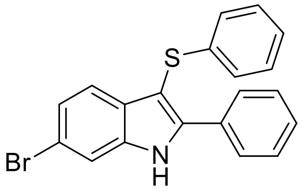

6-bromo-2-phenyl-3-(phenylthio)-1*H*-indole (6n)

This compound was prepared according to the general procedure, yellow oil, 292.9 mg, 77%. ^{1}H NMR (400 MHz, $CDCl_3$) δ 8.47 (s, 1H), 7.65–7.63 (m, 2H), 7.48 (d, *J* = 1.6 Hz, 1H), 7.39–7.27 (m, 4H), 7.16 (dd, *J* = 8.4, 1.6 Hz, 1H), 7.10–7.05 (m, 2H), 6.99–6.95 (m, 3H). ^{13}C{^{1}H} NMR (100 MHz, $CDCl_3$) δ 142.7, 138.8, 136.6, 131.0, 130.2, 129.1, 129.0 (two peaks), 128.2, 125.6, 124.9, 124.6, 121.3, 116.8, 114.2, 99.8. HRMS (ESI–TOF) m/z: $[M+H]^+$ calcd for $C_{20}H_{15}BrNS$ 380.0103; found 380.0081.

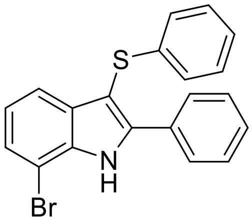

7-bromo-2-phenyl-3-(phenylthio)-1*H*-indole (6o)

This compound was prepared according to the general procedure, yellow oil, 197.8 mg, 52%. ^{1}H NMR (400 MHz, $CDCl_3$) δ 8.54 (s, 1H), 7.67–7.64 (m, 2H), 7.46 (d, J = 8.0 Hz, 1H), 7.35–7.25 (m, 4H), 7.10–7.03 (m, 2H), 6.99–6.90 (m, 4H). ^{13}C{^{1}H} NMR (100 MHz, $CDCl_3$) δ 142.9, 138.8, 134.6, 132.4, 130.9, 129.2, 129.0, 128.9, 128.4, 125.8, 125.7, 125.0, 122.4, 119.3, 104.7, 101.0. HRMS (ESI–TOF) m/z: $[M+H]^+$ calcd for $C_{20}H_{15}BrNS$ 380.0103; found 380.0094.

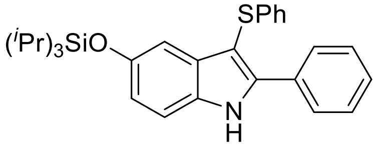

2-phenyl-3-(phenylthio)-5-((triisopropylsilyl)oxy)-1*H*-indole (6p)

This compound was prepared according to the general procedure, yellow oil, 340.2 mg, 72%. ^{1}H NMR (400 MHz, $CDCl_3$) δ 8.36 (s, 1H), 7.76–7.73 (m, 2H), 7.46–7.36 (m, 3H), 7.28 (d, J = 8.8 Hz, 1H), 7.18–7.11 (m, 4H), 7.22–7.18 (m, 2H), 7.08–7.03 (m, 2H), 6.88 (dd, J = 8.8, 2.4 Hz, 1H), 1.22–1.13 (m, 3H), 1.07–1.04 (m, 18H). ^{13}C{^{1}H} NMR (100 MHz, $CDCl_3$) δ 151.0, 142.4, 139.1, 132.0, 131.7, 131.2, 128.9, 128.8, 128.7, 128.2, 126.1, 124.8, 117.6, 111.6, 109.2, 99.4, 18.1, 12.7. HRMS (ESI–TOF) m/z: $[M+H]^+$ calcd for $C_{29}H_{36}NOSSi$ 474.2281; found 474.2273.

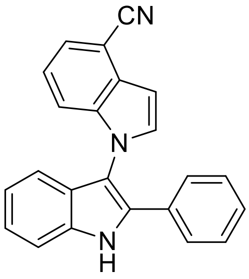

2'-phenyl-1'*H*-[1,3'-biindole]-4-carbonitrile (6q)

This compound was prepared according to the general procedure, yellow solid, 242.8 mg, 73%. ^{1}H NMR (400 MHz, $CDCl_3$) δ 8.58 (s, 1H), 7.54–7.50 (m, 2H), 7.35–7.21 (m, 7H), 7.16–7.11 (m, 4H), 6.92 (dd, J = 3.2, 0.8 Hz, 1H). ^{13}C{^{1}H} NMR (100 MHz, $CDCl_3$) δ 137.1, 134.3, 132.8, 132.5, 130.0, 129.3, 128.7, 126.3, 126.2, 125.7, 123.8, 121.9, 121.3, 119.0, 118.1, 116.0, 112.1, 111.6, 103.3, 102.2. HRMS (ESI–TOF) m/z: $[M+H]^+$ calcd for $C_{23}H_{16}N_3$ 334.1339; found 334.1333.

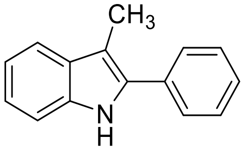

3-methyl-2-phenyl-1*H*-indole (6r)

This compound was prepared according to the general procedure, white solid, 113.7 mg, 55%. ^{1}H NMR (400 MHz, $CDCl_3$) δ 7.98 (s, 1H), 7.61–7.55 (m, 3H), 7.48–7.44 (m, 2H), 7.36–7.32 (m, 2H), 7.22–7.18 (m, 1H), 7.14 (td, J = 7.6, 1.2 Hz, 1H), 2.46 (s, 3H). ^{13}C{^{1}H} NMR (100 MHz, $CDCl_3$) δ 135.9, 134.2, 133.5, 130.1, 128.9, 127.9, 127.4, 122.4, 119.7, 119.1, 110.8, 108.8, 9.8.[16]

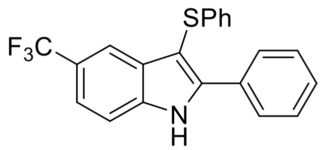

2-phenyl-3-(phenylthio)-5-(trifluoromethyl)-1*H*-indole (6s)

This compound was prepared according to the general procedure, yellow oil, 232.8 mg, 63%. ^{1}H NMR (400 MHz, $CDCl_3$) δ 8.73 (s, 1H), 7.97 (s, 1H), 7.78–7.75 (m, 2H), 7.52 (d, J = 1.2 Hz, 2H), 7.48–7.40 (m, 3H), 7.22–7.18 (m, 2H), 7.12–7.08 (m, 3H). ^{13}C{^{1}H} NMR (100 MHz, $CDCl_3$) δ 143.9, 138.7, 137.3, 131.0, 130.8, 129.4, 129.1, 129.0, 128.2, 126.5, 125.7, 125.1, 123.8 (q, J = 31.9 Hz), 120.3 (q, J = 3.4 Hz), 117.8 (q, J = 4.2 Hz), 111.7, 100.7.[20]

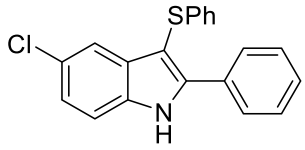

5-chloro-2-phenyl-3-(phenylthio)-1*H*-indole (6t)

This compound was prepared according to the general procedure, yellow oil, 113.8 mg, 34%. ^{1}H NMR (400 MHz, $CDCl_3$) δ 8.57 (s, 1H), 7.75–7.73 (m, 2H), 7.62 (d, J = 2.0 Hz, 1H), 7.48–7.40 (m, 3H), 7.36 (d, J = 8.8 Hz, 1H), 7.24 – 7.17 (m, 3H), 7.10 – 7.06 (m, 3H). ^{13}C{^{1}H} NMR (100 MHz, $CDCl_3$) δ 143.5, 138.9, 134.2, 132.6, 131.0, 129.2, 129.1, 129.0, 128.2, 127.2, 125.6, 125.0, 123.9, 119.5, 112.4, 99.2.[21]

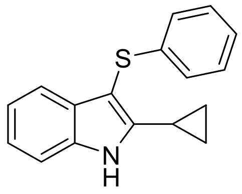

2-cyclopropyl-3-(phenylthio)-1*H*-indole (6u)

This compound was prepared according to the general procedure, yellow oil, 217.7 mg, 82%. ^{1}H NMR (400 MHz, $CDCl_3$) δ 7.82 (s, 1H), 7.52 (d, J = 7.6 Hz, 1H), 7.23 (d, J = 8.0 Hz, 1H), 7.16–7.05 (m, 6H), 7.01 (t, J = 7.2 Hz, 1H), 2.33–2.26 (m, 1H), 1.02–0.97 (m, 2H), 0.84–0.80 (m, 2H). ^{13}C{^{1}H} NMR (100 MHz, $CDCl_3$) δ 146.1, 139.5, 135.0, 130.8, 128.8, 125.5, 124.6, 122.2, 120.8, 118.7, 110.8, 99.1, 8.3, 8.0. HRMS (ESI–TOF) m/z: $[M+Na]^+$ calcd for $C_{17}H_{15}NNaS$ 288.0817; found 288.0804.

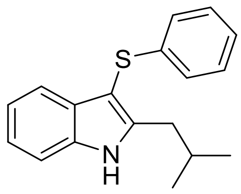

2-isobutyl-3-(phenylthio)-1*H*-indole (6v)

This compound was prepared according to the general procedure, yellow oil, 199.9 mg, 71%. ^{1}H NMR (400 MHz, $CDCl_3$) δ 8.17 (s, 1H), 7.53 (d, J = 8.0 Hz, 1H), 7.33 (dt, J = 8.0, 0.8 Hz, 1H), 7.21–7.17 (m, 1H), 7.14–7.09 (m, 3H), 7.04–6.99 (m, 3H), 2.76 (d, J = 7.6 Hz, 2H), 2.05–1.92 (m, 1H), 0.91 (d, J = 6.8 Hz, 6H). ^{13}C{^{1}H} NMR (100 MHz, $CDCl_3$) δ 144.7, 139.6, 135.6, 130.3, 128.7, 125.6, 124.5, 122.3, 120.8, 119.3, 110.9, 99.7, 35.7, 29.3, 22.6. HRMS (ESI–TOF) m/z: $[M+H]^+$ calcd for $C_{18}H_{20}NS$ 282.1311; found 282.1300.

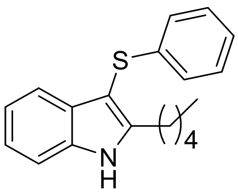

2-ethyl-3-(phenylthio)-1*H*-indole (6w)

This compound was prepared according to the general procedure, yellow oil, 121.4 mg, 41%. ^{1}H NMR (400 MHz, $CDCl_3$) δ 8.25 (s, 1H), 7.54 (d, *J* = 7.2 Hz, 1H), 7.36 (d, *J* = 8.0 Hz, 1H), 7.19 (td, *J* = 7.6, 1.2 Hz, 1H), 7.15–7.10 (m, 3H), 7.04–7.00 (m, 3H), 2.90 (t, *J* = 7.6 Hz, 2H), 1.70–1.62 (m, 2H), 1.31–1.25 (m, 4H), 0.85–0.81 (m, 3H). ^{13}C{^{1}H} NMR (100 MHz, $CDCl_3$) δ 145.7, 139.7, 135.6, 130.4, 128.7, 125.5, 124.5, 122.3, 120.8, 119.2, 110.8, 99.0, 31.5, 29.3, 26.5, 22.5, 14.1. HRMS (ESI–TOF) m/z: $[M+H]^+$ calcd for $C_{19}H_{22}NS$ 296.1467; found 296.1468.

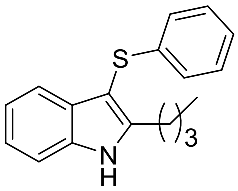

2-ethyl-3-(phenylthio)-1*H*-indole (6x)

This compound was prepared according to the general procedure, yellow oil, 106.9 mg, 38%. ^{1}H NMR (400 MHz, $CDCl_3$) δ 8.25 (s, 1H), 7.53 (d, *J* = 8.0 Hz, 1H), 7.33 (d, *J* = 8.0 Hz, 1H), 7.20–7.16 (m, 1H), 7.15–7.09 (m, 3H), 7.04–7.00 (m, 3H), 2.88 (t, *J* = 7.6 Hz, 2H), 1.65–1.58 (m, 2H), 1.38–1.26 (m, 2H), 0.87 (t, *J* = 7.2 Hz, 3H). ^{13}C{^{1}H} NMR (100 MHz, $CDCl_3$) δ 145.7, 139.6, 135.6, 130.3, 128.7, 125.5, 124.5, 122.2, 120.7, 119.1, 110.9, 98.8, 31.7, 26.2, 22.4, 13.9. HRMS (ESI–TOF) m/z: $[M+H]^+$ calcd for $C_{18}H_{20}NS$ 282.1311; found 282.1313.

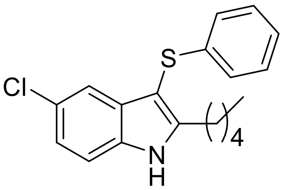

5-chloro-2-ethyl-3-(phenylthio)-1*H*-indole (6y)

This compound was prepared according to the general procedure, yellow oil, 122.1 mg, 37%. ^{1}H NMR (400 MHz, $CDCl_3$) δ 8.28 (s, 1H), 7.51 (d, *J* = 2.0 Hz, 1H), 7.24–7.22 (m, 1H), 7.17–7.11 (m, 3H), 7.06–6.99 (m, 3H), 2.86 (t, *J* = 7.6 Hz, 2H), 1.67–1.59 (m, 2H), 1.29–1.25 (m, 4H), 0.84–0.80 (m, 3H). ^{13}C{^{1}H} NMR (100 MHz, $CDCl_3$) δ 147.2, 139.2, 133.9, 131.8, 128.9, 126.7, 125.6, 124.8, 122.6, 118.7, 111.9, 99.0, 31.5, 29.2, 26.6, 22.5, 14.0. HRMS (ESI–TOF) m/z: $[M+H]^+$ calcd for $C_{19}H_{21}ClNS$ 330.1078; found 330.1065.

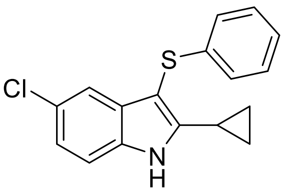

5-chloro-2-cyclopropyl-3-(phenylthio)-1*H*-indole (6z)

This compound was prepared according to the general procedure, yellow oil, 203.9 mg, 68%. ^{1}H NMR (400 MHz, $CDCl_3$) δ 7.95 (s, 1H), 7.49 (d, *J* = 2.0 Hz, 1H), 7.20–7.14 (m, 3H), 7.11–7.03 (m, 4H), 2.37–2.30 (m, 1H), 1.11–1.03 (m, 2H), 0.90–0.86 (m, 2H). $^{13}C\{^1H\}$ NMR (100 MHz, $CDCl_3$) δ 147.7, 139.1, 133.4, 132.2, 128.9, 126.8, 125.6, 124.8, 122.5, 118.3, 111.8, 99.3, 8.4, 8.3. HRMS (ESI–TOF) m/z: $[M+H]^+$ calcd for $C_{17}H_{15}ClNS$ 300.0608; found 300.0588.

Preparation of Starting Materials

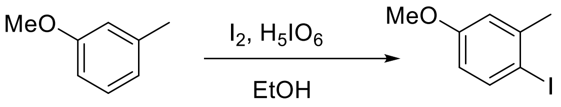

1m

H_5IO_6 (1.8 mmol, 410 mg) was weighed directly into a Schlenk tube and dried under vacuum for 15 min. Then 95% EtOH (10 mL) and I_2 (4.3 mmol, 1.09 g) were added. After 1-methoxy-3-methylbenzene (10 mmol, 1.26 mL) was added. The reaction mixtures were stirred at 80 ºC. The resulting reaction mixture was monitored by TLC. The reaction was quenched by H_2O and extracted with Et_2O. The combined organic extracts were washed with brine, dried over anhydrous Na_2SO_4, filtered, concentrated and purified on silica gel chromatography (EtOAc/petroleum ether as eluent) to obtain **1m** as a white solid, 1.54 g, 62%. ^{1}H NMR (400 MHz, $CDCl_3$) δ 7.63 (d, *J* = 8.8 Hz, 1H), 6.79 (d, *J* =3.2 Hz, 1H), 6.45 (dd, *J* = 8.8, 2.8 Hz, 1H), 3.74 (s, 3H), 2.37 (s, 3H). $^{13}C\{^1H\}$ NMR (100 MHz, $CDCl_3$) δ 159.9, 142.4, 139.4, 115.9, 113.4, 89.8, 55.4, 28.3.[1]

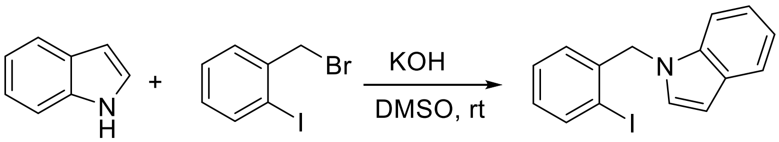

KOH (2 equiv) was weighed directly into a Schlenk tube and dried under vacuum for 15 min. Then DMSO (10 mL) was added and stirred for 5 minutes. Indole (5 mmol) was slowly added. After stirring for 30 min, 1-(bromomethyl)-2-iodobenzene (1.2 equiv) was added. The resulting reaction mixture was monitored by TLC. The reaction was quenched by H_2O and extracted with Et_2O. The combined organic extracts were washed with brine, dried over anhydrous Na_2SO_4, filtered, concentrated and purified on silica gel chromatography (EtOAc/petroleum ether as eluent) to obtain product.

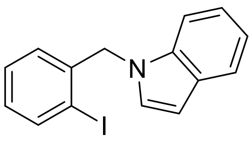

1-(2-iodobenzyl)-1*H*-indole (5bA)

This compound was prepared according to the general procedure, white solid, 1.23 g, 74%. ^{1}H NMR (400 MHz, $CDCl_3$) δ 7.87 (d, *J* = 8.0 Hz, 1H), 7.67 (d, *J* = 8.0 Hz, 1H), 7.22–7.10 (m, 5H), 6.94 (t, *J* = 7.6 Hz, 1H), 6.59 (d, *J* =

2.8 Hz, 1H), 6.45 (d, *J* = 8.0 Hz, 1H), 5.28 (s, 2H). $^{13}C\{^{1}H\}$ NMR (100 MHz, $CDCl_3$) δ 139.6, 139.5, 136.3, 129.4, 128.8 (two peaks), 128.4, 127.7, 122.0, 121.2, 119.9, 109.8, 102.2, 97.2, 55.2.[2]

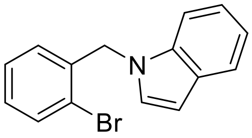

1-(2-bromobenzyl)-1*H*-indole (5bB)

This compound was prepared according to the general procedure, white solid, 1.17 g, 82%. ^{1}H NMR (400 MHz, $CDCl_3$) δ 7.67 (d, *J* = 7.6 Hz, 1H), 7.58 (t, *J* = 4.4 Hz, 1H), 7.23–7.06 (m, 6H), 6.58 (d, *J* = 2.8 Hz, 1H), 6.50 (t, *J* = 4.4 Hz, 1H), 5.36 (s, 2H). $^{13}C\{^{1}H\}$ NMR (100 MHz, $CDCl_3$) δ 136.8, 136.4, 132.8, 129.2, 128.8, 128.5, 128.1, 127.9, 122.3, 122.0, 121.2, 119.9, 109.8, 102.2, 50.3.[2]

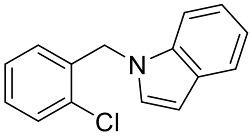

1-(2-chlorobenzyl)-1*H*-indole (5bC)

This compound was prepared according to the general procedure, white solid, 1.02 g, 85%. ^{1}H NMR (400 MHz, $CDCl_3$) δ 7.66 (d, *J* = 6.8 Hz, 1H), 7.39 (dd, *J* = 8.0, 1.2 Hz, 1H), 7.24–7.10 (m, 5H), 6.94 (tt, *J* = 8.0, 1.2 Hz, 1H), 6.58–6.55 (m, 2H), 5.40 (s, 2H). $^{13}C\{^{1}H\}$ NMR (100 MHz, $CDCl_3$) δ 136.4, 135.3, 132.5, 129.6, 128.9, 128.8, 128.5, 128.1, 127.3, 122.0, 121.2, 119.8, 109.7, 102.2, 47.8.[3]

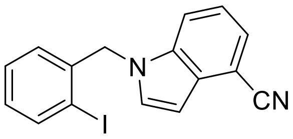

1-(2-iodobenzyl)-1*H*-indole-4-carbonitrile (5q)

This compound was prepared according to the general procedure, white solid, 1.41 g, 79%. ^{1}H NMR (400 MHz, $CDCl_3$) δ 7.90 (d, *J* = 8.0 Hz, 1H), 7.47 (dd, *J* = 11.6, 7.6 Hz, 2H), 7.28 (d, *J* = 3.2 Hz, 1H), 7.23-7.17 (m, 2H), 7.00 (t, *J* = 8.0 Hz, 1H), 6.80 (d, *J* = 3.2 Hz, 1H), 6.48 (d, *J* = 7.6 Hz, 1H), 5.34 (s, 2H). $^{13}C\{^{1}H\}$ NMR (100 MHz, $CDCl_3$) δ 139.7, 138.5, 136.0, 131.0, 129.9, 129.8, 128.9, 127.6, 125.3, 121.6, 118.7, 114.6, 103.5, 101.2, 97.4, 55.4. HRMS (ESI–TOF) m/z: $[M+H]^+$ calcd for $C_{15}H_{12}IN_2$ 359.0040, found 359.0045.

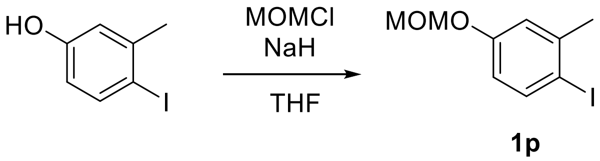

NaH (11 mmol) was added over 30 minutes to a solution of 4-iodo-3-methylphenol in THF at 0℃. MOMCl was added dropwise at room temperature to the yellow solution.

The resulting mixture was stirred for 16 h before the reaction was quenched with aqueous NaOH (1M, 20 mL). The aqueous phase was extracted with EtOAc. The combined organic layers were washed with brine, dried over anhydrous

Na_2SO4, filtered, concentrated and purified on silica gel chromatography (EtOAc/petroleum ether as eluent) to give the product **1p** as a colorless oil, 1.47g, 53%. ^{1}H NMR (400 MHz, $CDCl_3$) δ 7.67 (d, *J* = 8.8 Hz, 1H), 6.96 (d, *J* = 2.8 Hz, 1H), 6.62 (dd, *J* = 8.4, 2.8 Hz, 1H), 5.14 (s, 2H), 3.47 (s, 2H), 2.40 (s, 3H). ^{13}C{^{1}H} NMR (100 MHz, $CDCl_3$) δ 157.6, 142.6, 139.5, 118.1, 115.8, 94.5, 91.4, 56.2, 28.4. HRMS (ESI–TOF) m/z: $[M+H]^+$ calcd for $C_9H_{12}INO_2$ 278.9871, found 278.9858.

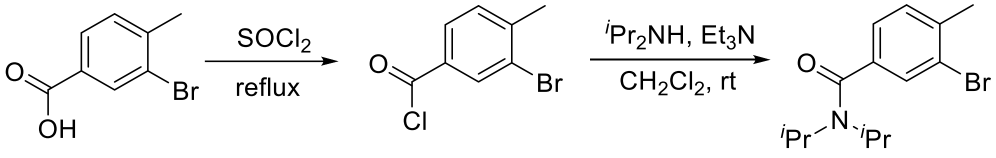

1s

The solution of 3-bromo-4-methylbenzoic acid (10 mmol) was refluxed in 10 mL $SOCl_2$ for 2 h. The mixture was cooled to rt. and the excess $SOCl_2$ was removed under vacuum to give 3-bromo-4-methylbenzoyl chloride. The acyl chloride was dissolved in 10 mL dry CH_2Cl_2 and added dropwise to a 20 mL solution of ^{i}Pr$_2$NH (15 mmol) and Et_3N (24 mmol) in dry CH_2Cl_2 at 0 °C. After stirring for 6 h at rt, the resulting mixture was washed with brine, and extracted with CH_2Cl_2, dried over anhydrous Na_2SO4, filtered, concentrated and purified on silica gel chromatography (EtOAc/petroleum ether as eluent) to give the product **1s** as a yellow solid, 2.38g, 80%. ^{1}H NMR (400 MHz, $CDCl_3$) δ 7.47 (s, 1H), 7.21 (d, *J* = 7.6 Hz, 1H), 7.12 (d, *J* = 7.6 Hz, 1H), 3.79 (brs, 1H), 3.51 (brs, 1H), 2.38 (s, 3H), 1.48 (brs, 6H), 1.46 (brs, 6H). ^{13}C{^{1}H} NMR (100 MHz, $CDCl_3$) δ 169.4, 138.5, 138.2, 130.8, 129.6, 125.0, 124.6, 50.9, 46.1, 22.9, 20.8. HRMS (ESI–TOF) m/z: $[M+Na]^+$ calcd for $C_{14}H_{20}BrNNaO$ 320.0620, found 320.0618.

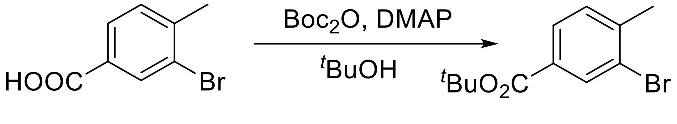

1t

To a solution of 3-bromo-4-methylbenzoic acid (4.3 g, 20 mmol) in t-BuOH (100 mL) was added Boc_2O (4.6 mL, 20 mmol) and DMAP (730 mg, 6 mmol), and the reaction mixture was stirred overnight at 40 ℃. The mixture was diluted with water and extracted with EtOAc (3 × 10 mL). The combined organic layers were washed with brine, dried over anhydrous Na_2SO_4, and concentrated by rotary evaporation. Purification by silica gel chromatography (EtOAc/petroleum ether as eluent) to obtain **1t** as a yellow oil, 2.98 g, 55%. ^{1}H NMR (400 MHz, $CDCl_3$) δ 8.12 (d, *J* = 1.6 Hz, 1H), 7.81 (dd, *J* = 8.0, 1.6 Hz, 1H), 7.27 (d, *J* = 8.0 Hz, 1H), 2.44 (s, 3H), 1.59 (s, 9H). ^{13}C{^{1}H} NMR (100 MHz, $CDCl_3$) δ 164.6, 142.8, 133.4, 131.5, 130.6, 128.4, 124.7, 81.6, 28.3, 23.3.[4]

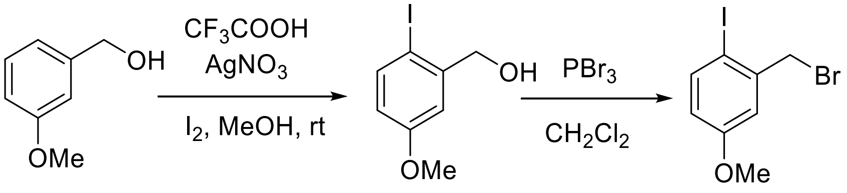

5f-1 **5f-2**

$AgNO_3$ (13 mmol) was weighed directly into a 100 mL round-bottom flask and dried under vacuum for 15 min. Then (3-methoxyphenyl)methanol (10 mmol) and CF_3COOH (13 mmol) were added. Then MeOH (15 mL) was added and cooled in an ice bath. A solution of I_2 (1.0 equiv) in MeOH (25 mL) was added dropwise over 30 min. The resulting reaction mixture was monitored by TLC. Then filtered and concentrated under vacuum. The residue **5f-1** was used in the next step without purification.

PBr_3 (1.0 mL, 16 mmol) was added over a solution of **5f-1** (8 mmol) in dry CH_2Cl_2 (100 mL), and the reaction mixture was stirred at rt for 5 h. Solvent was evaporated, and the resulting oil was treated with saturated $NaHCO_3$. The resulting aqueous phase was extracted with CH_2Cl_2. The combined organic extracts were dried over Na_2SO_4, filtered, concentrated and purified on silica gel chromatography (EtOAc/petroleum ether as eluent) to obtain **5f-2** as a white solid. 2.22g, 85%. ^{1}H NMR (400 MHz, $CDCl_3$) δ 7.69 (d, J = 8.4 Hz, 1H), 7.03 (d, J = 2.8 Hz, 1H), 6.59 (dd, J = 8.8, 3.2 Hz, 1H), 4.54 (s, 2H), 3.79 (s, 3H). ^{13}C{^{1}H} NMR (100 MHz, $CDCl_3$) δ 160.3, 141.1, 140.6, 116.5, 116.3, 88.5, 55.6, 38.9.[5]

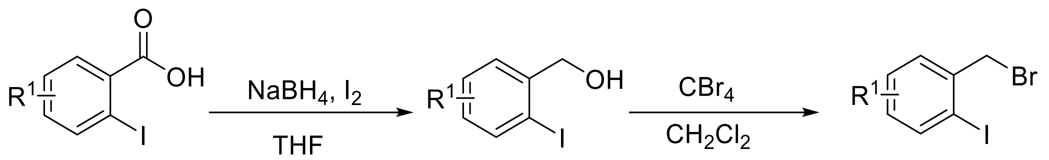

Benzoic acid (8 mmol) was weighed directly into a 100 mL round-bottom flask and dried under vacuum for 15 min. Then THF (20 mL) was added. $NaBH_4$ (2.5 equiv) was added and cooled in an ice bath. A solution of I_2 (1.0 equiv) in THF (10 mL) was added dropwise over 30 min with vigorous evolution of hydrogen. After the flask was heated to reflux for 12 h and then cooled to room temperature, diluted with methanol until the solid disappeared. After stirring 30 min, the solvent was removed by rotary evaporation leaving a white paste which was dissolved by addition of 20% aqueous KOH (20 mL). The solution was stirred for 4 h and extracted with ethyl acetate (30 mL × 2). The combined organic extracts were washed with brine, dried over anhydrous $MgSO_4$, filtered, and concentrated under vacuum. The residue benzyl alcohol was used in the next step without purification.

A solution of triphenylphosphine (6.6 mmol) in dry CH_2Cl_2 (8 mL) was added dropwise to a stirred solution of carbon tetrabromide (6.6 mmol) and benzyl alcohol (6 mmol) in dry CH_2Cl_2 (30 mL) at room temperature under N_2. The reaction was stirred for 4 h at room temperature and was concentrated in vacuo to give the crude product. This was purified by flash chromatography.

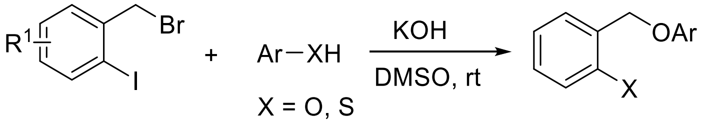

KOH (2 equiv) was weighed directly into a Schlenk tube and dried under vacuum for 15 min. Then DMSO (10 mL) was added and stirred for 5 minutes, phenol (5 mmol) was slowly added. After stirring for 30 min, 1-(bromomethyl)-2-iodobenzene (1.2 equiv) was added. The resulting reaction mixture was monitored by TLC. The reaction was quenched by H_2O and extracted with Et_2O. The combined organic extracts were washed with brine, dried over

anhydrous Na_2SO_4, filtered, concentrated and purified on silica gel chromatography (EtOAc/petroleum ether as eluent) to give the product.

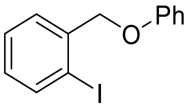

1-iodo-2-(phenoxymethyl)benzene (5c)

This compound was prepared according to the general procedure, white solid, 1.32 g, 85%. ^{1}H NMR (400 MHz, $CDCl_3$) δ 7.86 (d, J = 7.6 Hz, 1H), 7.52 (d, J = 7.6 Hz, 1H), 7.38–7.29 (m, 3H), 7.04–6.98 (m, 4H), 5.04 (s, 2H). ^{13}C{^{1}H} NMR (100 MHz, $CDCl_3$) δ 158.5, 139.4, 139.3, 129.7, 129.6, 128.8, 128.5, 121.4, 115.1, 97.3, 74.0.[6]

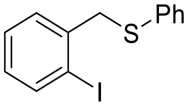

(2-iodobenzyl)(phenyl)sulfane (5dA)

This compound was prepared according to the general procedure, colorless oil, 1.32 g, 81%. ^{1}H NMR (400 MHz, $CDCl_3$) δ 7.83 (d, J = 7.6 Hz, 1H), 7.33–7.30 (m, 2H), 7.27–7.17 (m, 5H), 6.92–6.88 (m, 1H), 4.18 (s, 2H). ^{13}C{^{1}H} NMR (100 MHz, $CDCl_3$) δ 139.9, 139.8, 135.7, 130.9, 130.1, 129.0, 128.4, 126.9, 100.7, 44.8.[7]

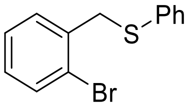

(2-bromobenzyl)(phenyl)sulfane (5dB)

This compound was prepared according to the general procedure, colorless oil, 1.32 g, 81%. ^{1}H NMR (400 MHz, $CDCl_3$) δ 7.53 (d, J = 8.0 Hz, 1H), 7.32–7.30 (m, 2H), 7.25–7.13 (m, 5H), 7.06 (tt, J = 7.6, 1.6 Hz, 1H), 4.19 (s, 2H). ^{13}C{^{1}H} NMR (100 MHz, $CDCl_3$) δ 136.8, 135.7, 133.0, 130.8, 130.7, 129.0, 128.9, 127.5, 126.8, 124.6, 39.7. [7]

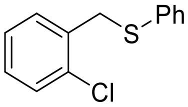

(2-chlorobenzyl)(phenyl)sulfane (5dC)

This compound was prepared according to the general procedure, colorless oil, 1.32 g, 81%. ^{1}H NMR (400 MHz, $CDCl_3$) δ 7.36 (dd, J = 7.6, 1.2 Hz, 1H), 7.33–7.31 (m, 2H), 7.28–7.11 (m, 6H), 4.21 (s, 2H). ^{13}C{^{1}H} NMR (100 MHz, $CDCl_3$) δ 135.8, 135.3, 134.2, 130.8 (two peaks), 129.8, 129.0, 128.7, 126.9 (two peaks), 37.1.

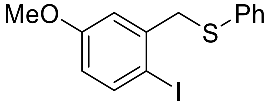

(2-iodo-5-methoxybenzyl)(phenyl)sulfane (5f)

This compound was prepared according to the general procedure, colorless oil, 1.28 g, 75%. **^{1}**H NMR (400 MHz, $CDCl_3$) δ 7.68 (d, J = 8.8 Hz, 1H), 7.35–7.32 (m, 2H), 7.28–7.19 (m, 3H), 6.78 (d, J = 3.2 Hz, 1H), 6.53 (dd, J = 8.8, 3.2 Hz, 1H), 4.14 (s, 2H), 3.66 (s, 3H). ^{13}C{^{1}H} NMR (100 MHz, $CDCl_3$) δ 159.9, 140.9, 140.1, 135.7, 131.0, 129.0, 127.0, 115.7, 115.5, 89.0, 55.4, 44.9. HRMS (ESI–TOF) m/z: $[M+H]^+$ calcd for $C_{14}H_{14}IOS$ 356.9805; found 356.9794.

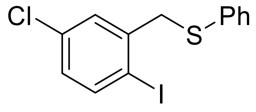

(5-chloro-2-iodobenzyl)(phenyl)sulfane (5y)

This compound was prepared according to the general procedure, colorless oil, 1.33 g, 74%. ^{1}H NMR (400 MHz, $CDCl_3$) δ 7.71 (d, J = 8.4 Hz, 1H), 7.33–7.21 (m, 5H), 7.17 (dd, J = 4.4, 2.4 Hz, 1H), 6.90 (dd, J = 8.4, 2.8 Hz, 1H), 4.11 (d, J = 2.4 Hz, 2H). ^{13}C{^{1}H} NMR (100 MHz, $CDCl_3$) δ 141.9, 140.6, 135.0, 134.5, 131.2, 130.0, 129.1, 129.0, 127.2, 97.7, 44.6. HRMS (ESI–TOF) m/z: $[M+H]^+$ calcd for $C_{13}H_{11}ClIS$ 360.9309; found 360.9327.

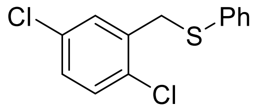

(2,5-dichlorobenzyl)(phenyl)sulfane (5t)

This compound was prepared according to the general procedure, colorless oil, 1.1 g, 81%. ^{1}H NMR (400 MHz, $CDCl_3$) δ 7.33–7.21 (m, 6H), 7.19 (d, J = 2.4 Hz, 1H), 7.15 (dd, J = 8.4, 1.6 Hz, 1H), 4.14 (s, 2H). ^{13}C{^{1}H} NMR (100 MHz, $CDCl_3$) δ 137.2, 135.1, 132.6, 132.4, 131.2, 130.8, 130.6, 129.1, 128.7, 127.3, 37.0. HRMS (ESI–TOF) m/z: $[M+H]^+$ calcd for $C_{13}H_{11}Cl_2S$ 268.9953; found 268.9935.

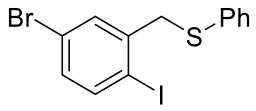

(5-bromo-2-iodobenzyl)(phenyl)sulfane (5mA)

This compound was prepared according to the general procedure, colorless oil, 1.34 g, 66%. ^{1}H NMR (400 MHz, $CDCl_3$) δ 7.66 (d, J = 8.4 Hz, 1H), 7.33–7.22 (m, 6H), 7.05 (dd, J = 8.4, 2.4 Hz, 1H), 4.11 (s, 2H). ^{13}C{^{1}H} NMR (100 MHz, $CDCl_3$) δ 142.2, 140.9, 134.9, 133.0, 132.0, 131.4, 129.1, 127.4, 122.6, 98.6, 44.7. HRMS (ESI–TOF) m/z: $[M+H]^+$ calcd for $C_{13}H_{11}BrIS$ 404.8804; found 404.8795.

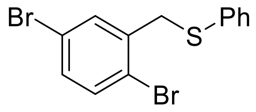

(2,5-dibromobenzyl)(phenyl)sulfane (5mB)

This compound was prepared according to the general procedure, colorless oil, 1.27 g, 71%. ^{1}H NMR (400 MHz, $CDCl_3$) δ 7.40 (d, J = 8.8 Hz, 1H), 7.33–7.23 (m, 6H), 7.21 (dd, J = 8.4, 2.0 Hz, 1H), 4.13 (s, 2H). ^{13}C{^{1}H} NMR (100 MHz, $CDCl_3$) δ 139.2, 135.0, 134.3, 133.6, 131.8, 131.3, 129.1, 127.3, 123.2, 121.2, 39.7. HRMS (ESI–TOF) m/z: $[M+H]^+$ calcd for $C_{13}H_{11}Br_2S$ 356.8943; found 404.8935.

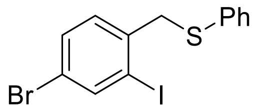

(4-bromo-2-iodobenzyl)(phenyl)sulfane (5n)

This compound was prepared according to the general procedure, colorless oil, 1.54 g, 76%. ^{1}H NMR (400 MHz, $CDCl_3$) δ 7.97 (d, J = 2.0 Hz, 1H), 7.33–7.20 (m, 6H), 7.04 (d, J = 8.4 Hz, 1H), 4.11 (s, 2H). ^{13}C{^{1}H} NMR (100

MHz, $CDCl_3$) δ 141.6, 139.2, 135.1, 131.4, 131.2, 130.9, 129.1, 127.2, 121.5, 101.0, 44.3. HRMS (ESI–TOF) m/z: $[M+Na]^+$ calcd for $C_{13}H_{10}BrINaS$ 426.8623; found 426.8629.

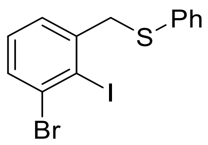

(3-bromo-2-iodobenzyl)(phenyl)sulfane (5o)

This compound was prepared according to the general procedure, white solid, 1.58 g, 78%. ^1H NMR (400 MHz, $CDCl_3$) δ 7.48 (dd, *J* = 7.2, 2.4 Hz, 1H), 7.32–7.19 (m, 5H), 7.08–7.02 (m, 2H), 4.27 (s, 2H). $^{13}C\{^1H\}$ NMR (100 MHz, $CDCl_3$) δ 143.5, 135.3, 131.6 (two peaks), 131.1, 129.2, 129.1, 128.1, 127.1, 108.1, 47.5. HRMS (ESI–TOF) m/z: $[M+Na]^+$ calcd for $C_{13}H_{10}BrINaS$ 426.8623; found 426.8643.

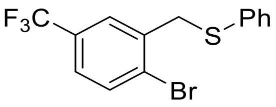

(2-bromo-5-(trifluoromethyl)benzyl)(phenyl)sulfane (5s)

This compound was prepared according to the general procedure, colorless oil, 1.15 g, 66%. ^1H NMR (400 MHz, $CDCl_3$) δ 7.67 (d, *J* = 9.2 Hz, 1H), 7.33–7.23 (m, 7H), 4.19 (s, 2H). $^{13}C\{^1H\}$ NMR (100 MHz, $CDCl_3$) δ 138.3, 134.5, 133.6, 132.0, 130.0, 129.7, 129.2, 128.4, 127.7, 127.5 (q, *J* = 3.7 Hz), 125.4 (q, *J* = 3.7 Hz), 125.0, 122.3, 40.2. HRMS (ESI–TOF) m/z: $[M+H]^+$ calcd for $C_{14}H_{11}BrF_3S$ 346.9711; found 346.9701.

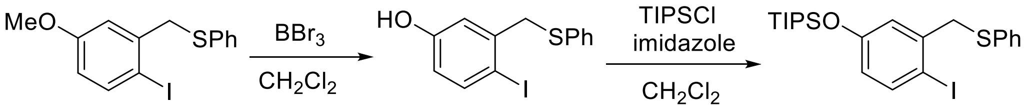

5p

5f (10 mmol)was dissolved in 50 mL of DCM. BBr_3 (25 mmol) was added to the mixture and the mixture stirred at room temperature until no starting material remained by TLC. The mixture was diluted with water and extracted with EtOAc (3 × 30 mL). The combined organic layers were washed with brine, dried over anhydrous Na_2SO_4, and concentrated by rotary evaporation. Purification by silica gel chromatography (EtOAc/petroleum ether as eluent) to obtain **1u** as a yellow oil.

1u (5 mmol) and imidazole (12.5 mmol) were dissolved in 30 mL of CH_2Cl_2. Triisopropylsilyl chloride (6 mmol) was added to the mixture and the mixture stirred at room temperature until no starting material remained by TLC. The reaction mixture was extracted using distilled H_2O (1 ×70 mL) and extracted with EtOAc (3 ×30 mL). The combined organic layers were washed with brine, dried over anhydrous Na_2SO_4 and concentrated to afford pure product **5p** as a colorless oil (2.2 g, 88%). ^1H NMR (400 MHz, $CDCl_3$) δ 7.63 (d, *J* = 8.8 Hz, 1H), 7.32–7.30 (m, 2H), 7.27–7.23 (m, 2H), 7.21–7.16 (m, 1H), 6.82 (d, *J* = 2.8 Hz, 1H), 6.50 (d, *J* = 8.4, 2.8 Hz, 1H), 4.12 (s, 2H), 1.16–1.08 (m, 3H), 1.04–1.00 (m, 18H). $^{13}C\{^1H\}$ NMR (100 MHz, $CDCl_3$) δ 156.6, 140.8, 140.1, 135.6, 130.3, 129.0, 126.6, 121.8, 121.1, 89.6, 44.1, 18.0, 12.6. HRMS (ESI–TOF) m/z: $[M+Na]^+$ calcd for $C_{22}H_{31}INaOSSi$ 521.0802; found 521.0795.

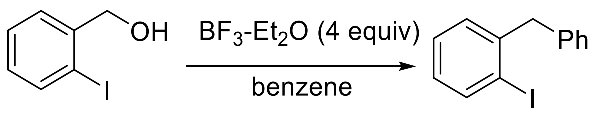

BF$_3$-Et$_2$O (4.9 mL, 40 mmol) and (2-iodophenyl) methanol (1.08 g, 10 mmol) were taken round-bottom flask. Then benzene (20 mL) was added. After stirring for 2 h at 80 ºC in argon atmosphere. Benzene was concentrated and the residue was purified on silica gel chromatography (EtOAc/petroleum ether as eluent) to obtain **5a** as a colorless oil (2.18g, 74%). ^{1}H NMR (400 MHz, CDCl$_3$) δ 7.84 (d, *J* = 7.6 Hz, 1H), 7.30–7.16 (m, 6H), 7.09 (d, *J* = 7.6 Hz, 1H), 6.91–6.87 (m, 1H), 4.10 (s, 2H). ^{13}C{^{1}H} NMR (100 MHz, CDCl$_3$) δ 143.7, 139.7, 130.5, 129.2, 128.6, 128.5, 128.1, 126.4, 101.5, 46.6.[8]

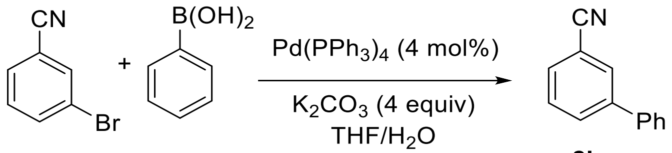

Aryl boronic acid (1.34 g, 11 mmol) and Pd(PPh$_3$)$_4$ (462 mg, 0.4 mmol) were added under argon atmosphere. Aryl bromide (1.82 g, 10 mmol) and K$_2$CO$_3$ (5.5 g, 40 mmol) were added. Then THF (60 mL) and H$_2$O (8 mL) were added under Argon and stirred at 70 ℃. The reaction was monitored by TLC until the starting material disappeared and cooled to room temperature, filtered through a thin silica gel pad and washed with EtOAc. The filtrate was concentrated and the residue was purified by chromatography on silica gel (PE/EA = 200/1 to PE/EA = 10/1) to afford the corresponding product **2h** as a white solid (1.72 g, 96%). ^{1}H NMR (400 MHz, CDCl$_3$) δ 7.82 (t, *J* = 1.6 Hz, 1H), 7.78 (dt, *J* = 7.6, 1.6 Hz, 1H), 7.60 (dt, *J* = 8.0, 1.6 Hz, 1H), 7.54–7.50 (m, 3H), 7.48–7.43 (m, 2H), 7.40 (tt, *J* = 7.2, 1.6 Hz, 1H). ^{13}C{^{1}H} NMR (100 MHz, CDCl$_3$) δ 142.4, 138.8, 131.5, 130.7 (two peaks), 129.6, 129.2, 128.4, 127.1, 118.9, 112.9.[9]

Synthesis of 8: ^{t}BuOK (150 mmol, 16.8 g) and CuSO$_4$ (1.2 mmol, 192 mg) were weighed directly into a 250 mL round bottom flask and dried under high vacuum for 15 min. Then dioxane (50 mL), **5f** (60 mmol, 21.4 g), and 4-methoxybenzonitrile (150 mmol, 20g) were added under argon and stirred at 100 ℃ and the reaction was monitored by TLC until the starting material disappeared and cooled to room temperature, filtered through a thin silica gel pad and washed with EtOAc. The filtrate was concentrated and the residue was purified by chromatography on silica gel (PE/EA = 200/1 to PE/EA = 10/1) to afford the corresponding product **6f** as a yellow oil (19.3 g, 89%).

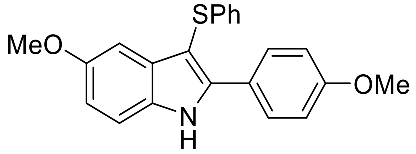

5-methoxy-2-(4-methoxyphenyl)-3-(phenylthio)-1*H*-indole (6f)

^{1}H NMR (400 MHz, CDCl$_3$) δ 8.40 (s, 1H), 7.64–7.62 (m, 2H), 7.28 (d, *J* = 8.4 Hz, 1H), 7.18–7.14 (m,

2H), 7.10–7.02 (m, 4H), 6.92–6.87 (m, 3H), 3.79 (s, 3H), 3.77 (s, 3H). $^{13}C\{^1H\}$ NMR (100 MHz, $CDCl_3$) δ 160.0, 155.3, 142.9, 139.6, 132.3, 130.6, 129.4, 128.9, 125.4, 124.6, 124.1, 114.3, 113.4, 112.0, 101.1, 97.9, 55.9, 55.4. HRMS (ESI–TOF) m/z: $[M+Na]^+$ calcd for $C_{22}H_{19}NNaO_2S$ 384.1029; found 384.1034.

2–Mercaptobenzoic acid **7** (94 mmol, 14.5 g,) was weighed directly into a 250 mL round bottom flask and dried under high vacuum for 15 min. Then **6f** (17.1 g, 47.3 mmol) and trifluoroacetic acid (94 mL) were added under Ar, and it was stirred at 30 ºC and the reaction was monitored by TLC until the starting material disappeared, filtered through a thin silica gel pad and washed with EtOAc. The filtrate was concentrated, after which it was added to 0.5 M aqueous NaOH. The solution was extracted with ethyl acetate. The combined organic extracts were washed with brine, dried over anhydrous $MgSO_4$, The filtrate was concentrated and the residue was purified by chromatography on silica gel (PE/EA = 200/1 to PE/EA = 10/1) to afford **3r** as a white solid (11 g, 92%)

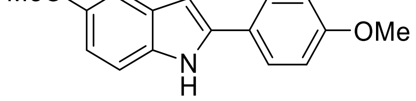

5-methoxy-2-(4-methoxyphenyl)-1*H*-indole (3v)

^1H NMR (400 MHz, DMSO–*d6*) δ 11.29 (s, 1H), 7.79 (d, J = 8.8 Hz, 2H), 7.29 (d, J = 8.4 Hz, 1H), 7.07–7.03 (m, 3H), 6.75 (d, J = 8.4 Hz, 1H), 6.71 (s, 1H), 3.84 (s, 3H), 3.79 (s, 3H). $^{13}C\{^1H\}$ NMR (100 MHz, DMSO–*d6*) δ 158.7, 153.5, 138.3, 132.0, 129.2, 126.2, 125.0, 114.3, 111.7, 111.1, 101.4, 97.3, 55.2 (two peaks).[22]

$POCl_3$ (8 g, 52.2 mmol) was added dropwise to stirred DMF (50 mL) at 0 ºC, after the addition was complete, the mixture was stirred for an additional 1 h. To the vigorously stirred solution was added a solution of **3r** (11 g, 43.5 mmol) in DMF (100 mL) dropwise at 0 °C over 30 min. The reaction within minutes a heavy precipitate was formed, then the reaction was warmed up to room temperature and the reaction mixture was stirred for 3 h. Addition of water dissolved the precipitate, after 30 min of stirring, the solution pH was adjusted to 6 by adding NaOH solution (40%), then within minutes a heavy precipitate formed, filtered, wash with H_2O, dried under infrared drying lamp, the corresponding product **8** was obtained without further purification, as a yellow solid (12.2 g, 100%).

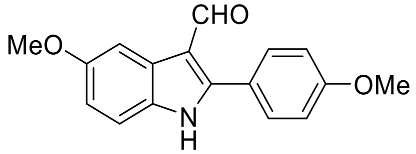

5-methoxy-2-(4-methoxyphenyl)-1*H*-indole-3-carbaldehyde (8)

^1H NMR (400 MHz, DMSO–*d6*) δ 12.19 (s, 1H), 9.94 (s, 1H), 7.73–7.70 (m, 3H), 7.39 (d, J = 8.8 Hz, 1H), 7.15 (d, J = 8.4 Hz, 2H), 6.91 (dd, J = 8.8, 1.6 Hz, 1H), 3.86 (s, 3H), 3.81 (s, 3H). $^{13}C\{^1H\}$ NMR (100 MHz, DMSO–*d6*) δ 185.3, 160.5, 155.8, 149.3, 131.2, 130.6, 126.8, 122.2, 114.5, 113.1 (two peaks), 112.7, 102.8, 55.4, 55.3. HRMS (ESI–TOF) m/z: $[M+Na]^+$ calcd for $C_{17}H_{15}NO_3Na$ 304.0950; found

304.0948.

Synthesis of BACE1 Inhibitor 4b

^{t}BuOK (52 mmol, 5.8 g) and $CuSO_4$ (0.26 mmol, 41.5 mg) were weighed directly into a 50 mL round bottom flask and dried under high vacuum for 15 min. Octane (10 mL), **1a** (13 mmol, 2.83 g), and **2a** (65 mmol, 6.7 g) were added under argon and stirred at 90 ℃ until the disappearance of **1a**. The resulting mixture was cooled to RT, filtered through a thin silica gel pad and washed with EtOAc. The filtrate was concentrated and purified by chromatography on silica gel (PE/EA = 200/1 to PE/EA = 10/1) to afford the corresponding product **3a** as a white solid (1.93 g, 77%). To **3a** (10 mmol, 1.93 g) in 20 mL DMF was added slowly KOH (12 mmol, 792 mg) in an ice bath. After stirring for 1 h, the bromo epoxide (25 mmol, 3.43 g) was added by syringe. The reaction mixture was stirred at RT for 18 h. The reaction mixture was concentrated and purified by silica gel flash chromatography using petroleum ether/EtOAc (10:1) to afford intermediate **4a** as a yellow oil (2.3 g, 93%).

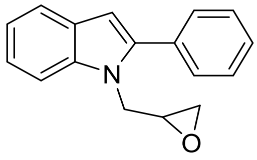

1-(oxiran-2-ylmethyl)-2-phenyl-1*H*-indole (4a)

^{1}H NMR ($CDCl_3$, 400 MHz) δ 7.63 (dt, *J* = 8.0, 1.2 Hz, 1H), 7.54–7.51 (m, 2H), 7.48–7.38 (m, 4H), 7.27–7.22 (m, 1H), 7.18–7.14 (m, 1H), 6.57 (d, *J* = 0.8 Hz, 1H), 4.36 (dd, *J* = 14.6, 3.6 Hz, 1H), 4.24 (dd, *J* = 14.6, 3.6 Hz, 1H), 3.21–3.17 (m, 1H), 2.71 (dd, *J* = 4.8, 4.0 Hz, 1H), 2.38 (dd, *J* = 4.8, 2.4 Hz, 1H). $^{13}C\{^1H\}$ NMR ($CDCl_3$, 100 MHz) δ 141.7, 138.2, 132.7, 129.9, 128.7, 128.3, 122.1, 120.7, 120.4, 110.4, 102.9, 51.0, 45.8, 45.6. HRMS (ESI–TOF) m/z: $[M+H]^+$ calcd for $C_{17}H_{16}NO$ 250.1226; found 250.1232.

To the 250 mL flask charged with **4a** (9 mmol, 2.24 g) was added 1–naphthylamine (18 mmol, 2.58 g), $BiCl_3$ (9 mmol, 2.84 g), 54 mL C_6H_{12} and 18 mL CH_3CN. Then mixture was reacted at reflux and monitored by TLC. The mixture was concentrated under vacuum and the residue was purified by silica gel flash chromatography using petroleum ether/EtOAc (15:1 to 4:1) to afford **4b** as a yellow oil (2.9 g, 81%).

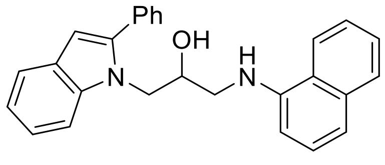

1-(naphthalen-1-ylamino)-3-(2-phenyl-1*H*-indol-1-yl)propan-2-ol (4b)

^{1}H NMR ($CDCl_3$, 400 MHz) δ 7.74 (d, *J* = 8.0 Hz, 1H), 7.63 (dd, *J* = 7.6, 0.8 Hz, 1H), 7.54–7.34 (m, 9H), 7.26 –7.21 (m, 3H), 7.16 (m, 1H), 6.56 (s, 1H), 6.32 (dd, *J* = 6.4, 2.4 Hz, 1H), 4.46–4.33 (m, 2H), 4.24 (s,

1H), 3.18–3.13 (m, 1H), 2.99–2.94 (m, 1H), 2.05 (s, 1H). $^{13}C\{^1H\}$ NMR ($CDCl_3$, 100 MHz) δ 143.0, 141.7, 138.0, 134.4, 133.0, 129.8, 128.9, 128.7, 128.5, 128.4, 126.5, 125.9, 124.9, 123.9, 122.2, 120.9, 120.5, 120.1, 118.3, 110.5, 105.0, 103.4, 69.3, 47.8, 47.2. HRMS (ESI–TOF) m/z: $[M+H]^+$ calcd for $C_{27}H_{25}N_2O$ 393.1961; found 393.1969.

Trapping 1a-OTEMP

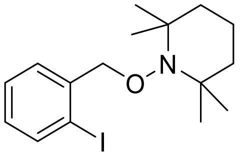

1-((2-iodobenzyl)oxy)-2,2,6,6-tetramethylpiperidine (1a-OTEMP)

^{t}BuOK (4 mmol, 449 mg) and $CuSO_4$ (0.02 mmol, 3.2 mg) were weighed directly into a Schlenk tube and dried under high vacuum for 15 min. Octane (0.75 mL), **1a** (1 mmol), TEMPO (2 mmol) and **2a** (5 mmol, 0.51 mL) were added under argon and stirred at 90 ℃ for 15 h. The mixture was concentrated and the residue was purified by silica gel flash chromatography using petroleum to afford **1a-OTEMP** as a white solid. ^1H NMR ($CDCl_3$, 400 MHz) δ 7.81 (dd, *J* = 8.0, 1.2 Hz, 1H), 7.55 (dt, *J* = 7.6, 0.8 Hz, 1H), 7.36 (td, *J* = 7.6, 1.2 Hz, 1H), 6.97 (td, *J* = 7.6, 1.6 Hz, 1H), 4.81 (s, 2H), 1.63–1.35 (m, 6H), 1.25 (s, 6H), 1.15 (s, 6H). $^{13}C\{^1H\}$ NMR ($CDCl_3$, 100 MHz) δ 141.3, 139.0, 128.7, 128.4, 128.1, 96.9, 82.3, 60.2, 39.3, 33.1, 20.6, 17.3. HRMS (ESI–TOF) m/z: $[M+H]^+$ calcd for $C_{16}H_{25}INO$ 374.0975; found 374.0979.

Supplementary Figures

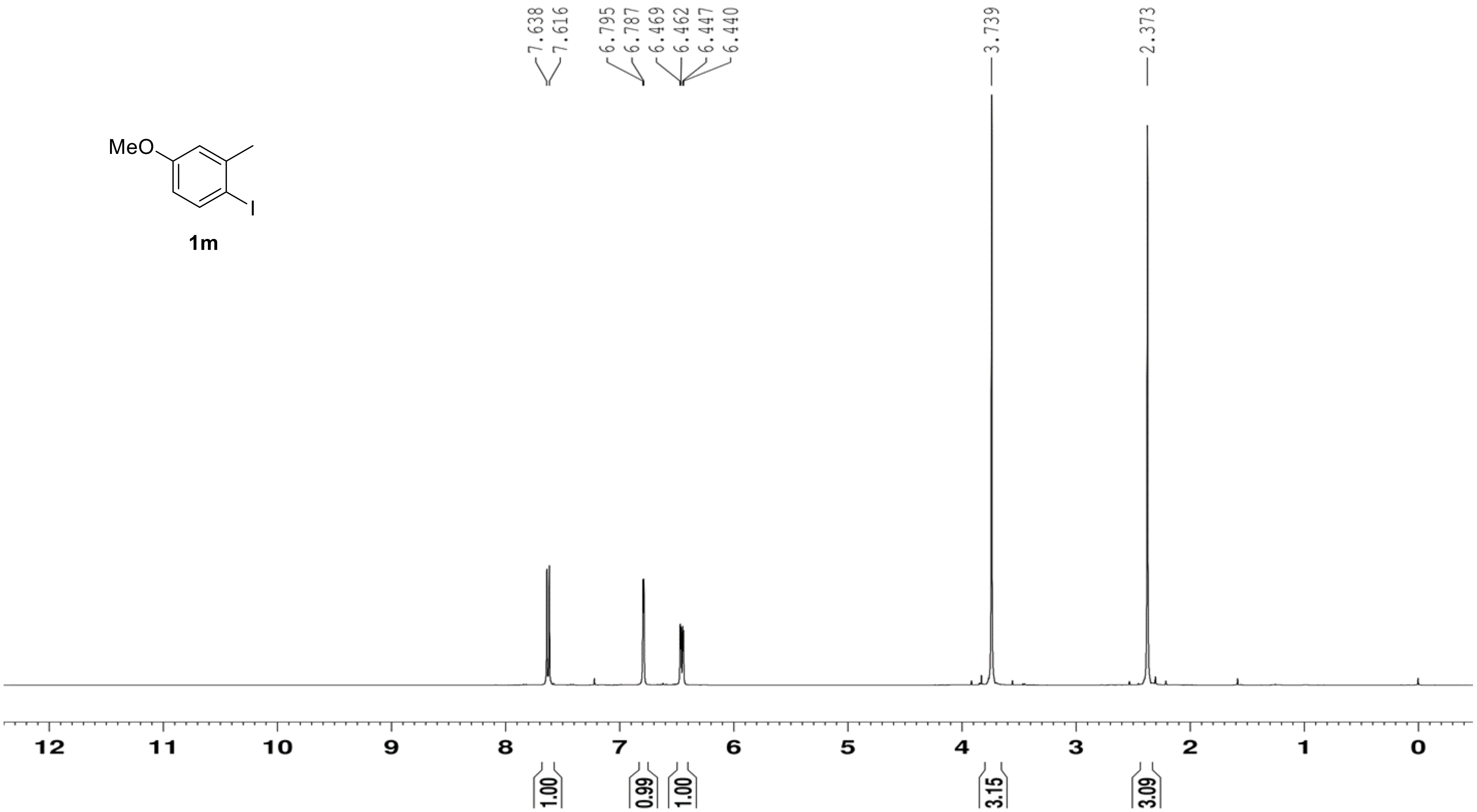

Supplementary Figure 1. ^{1}H NMR spectrum of **1m**.

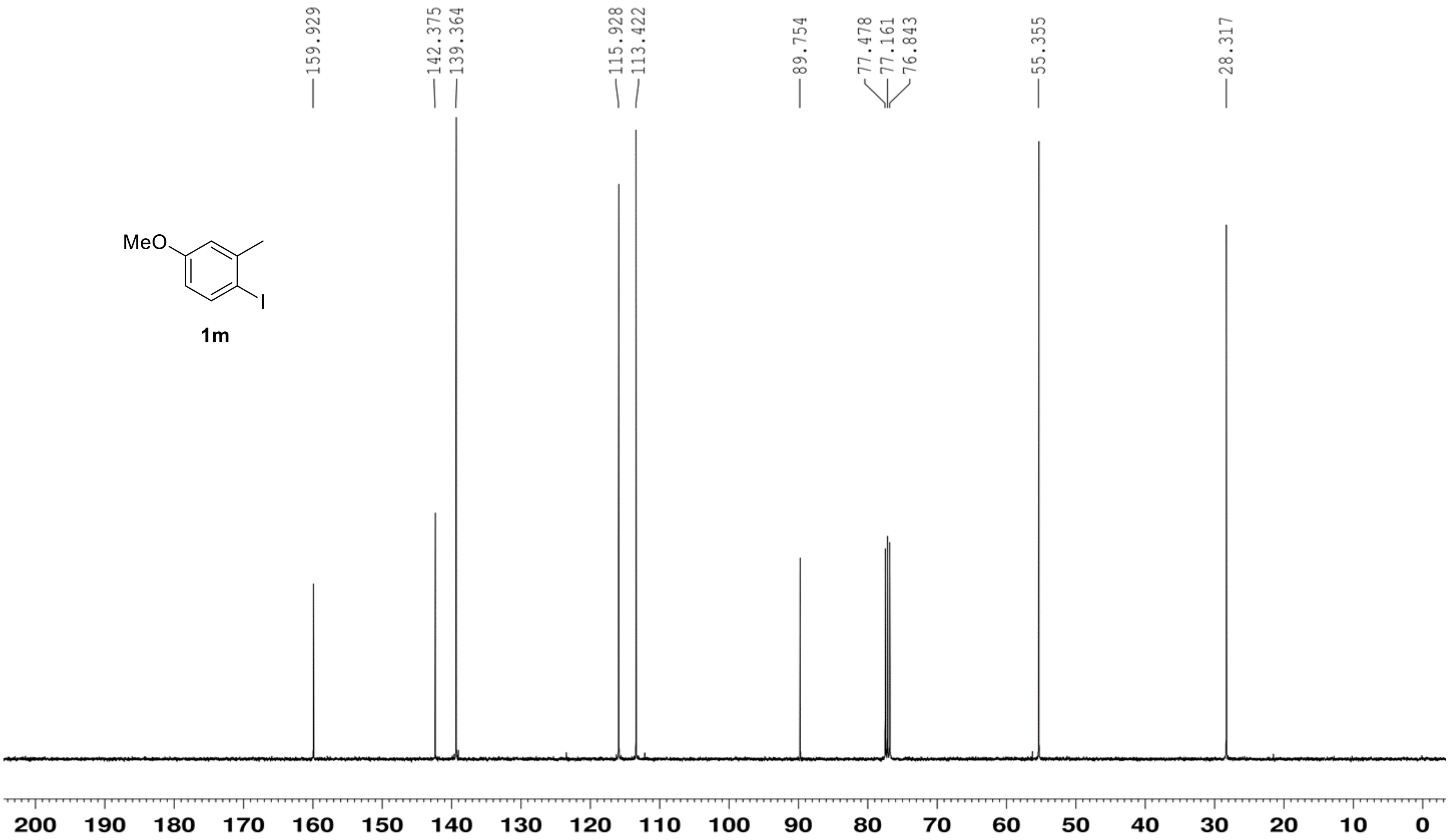

Supplementary Figure 2. ^{13}C NMR spectrum of **1m**.

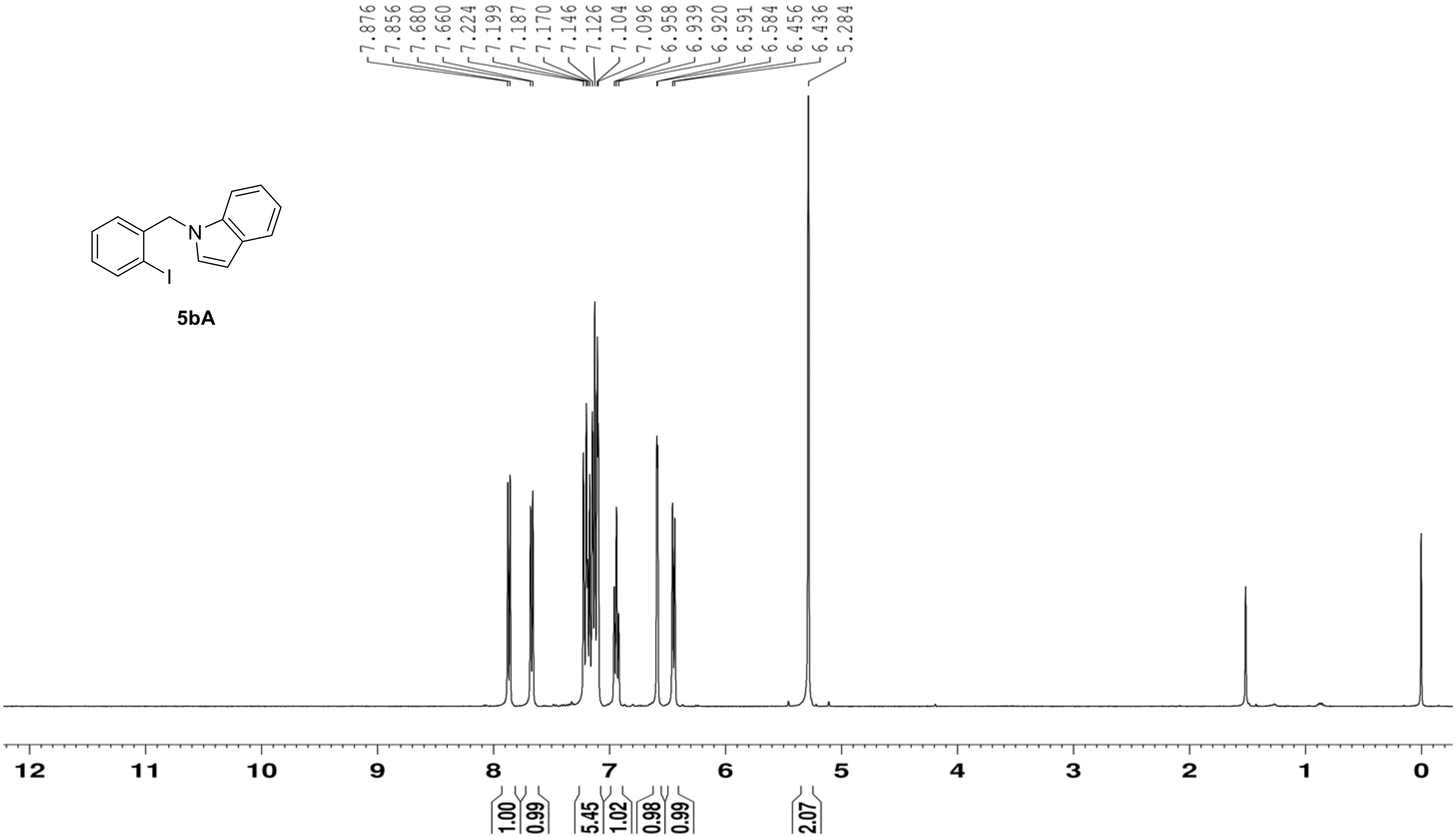

Supplementary Figure 3. ^{1}H NMR spectrum of **5bA**.

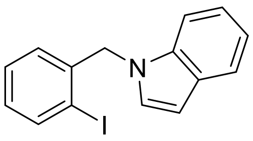

5bA

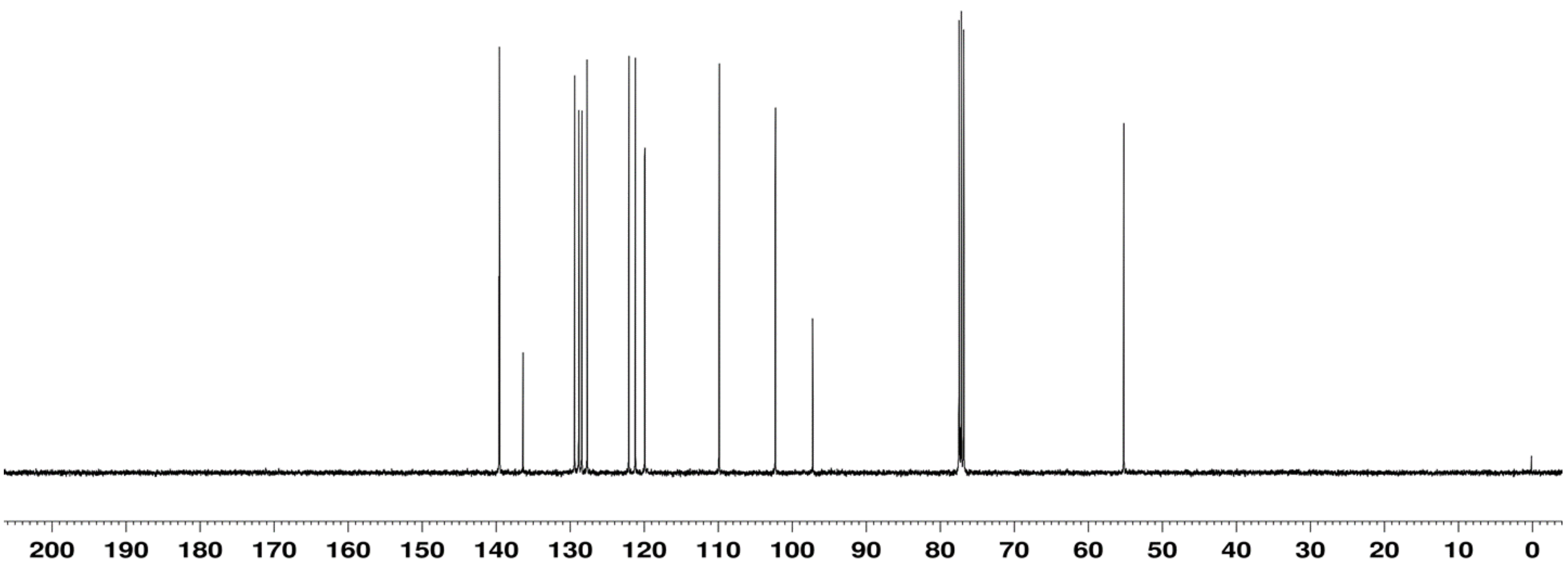

Supplementary Figure 4. ¹³C NMR spectrum of **5bA**.

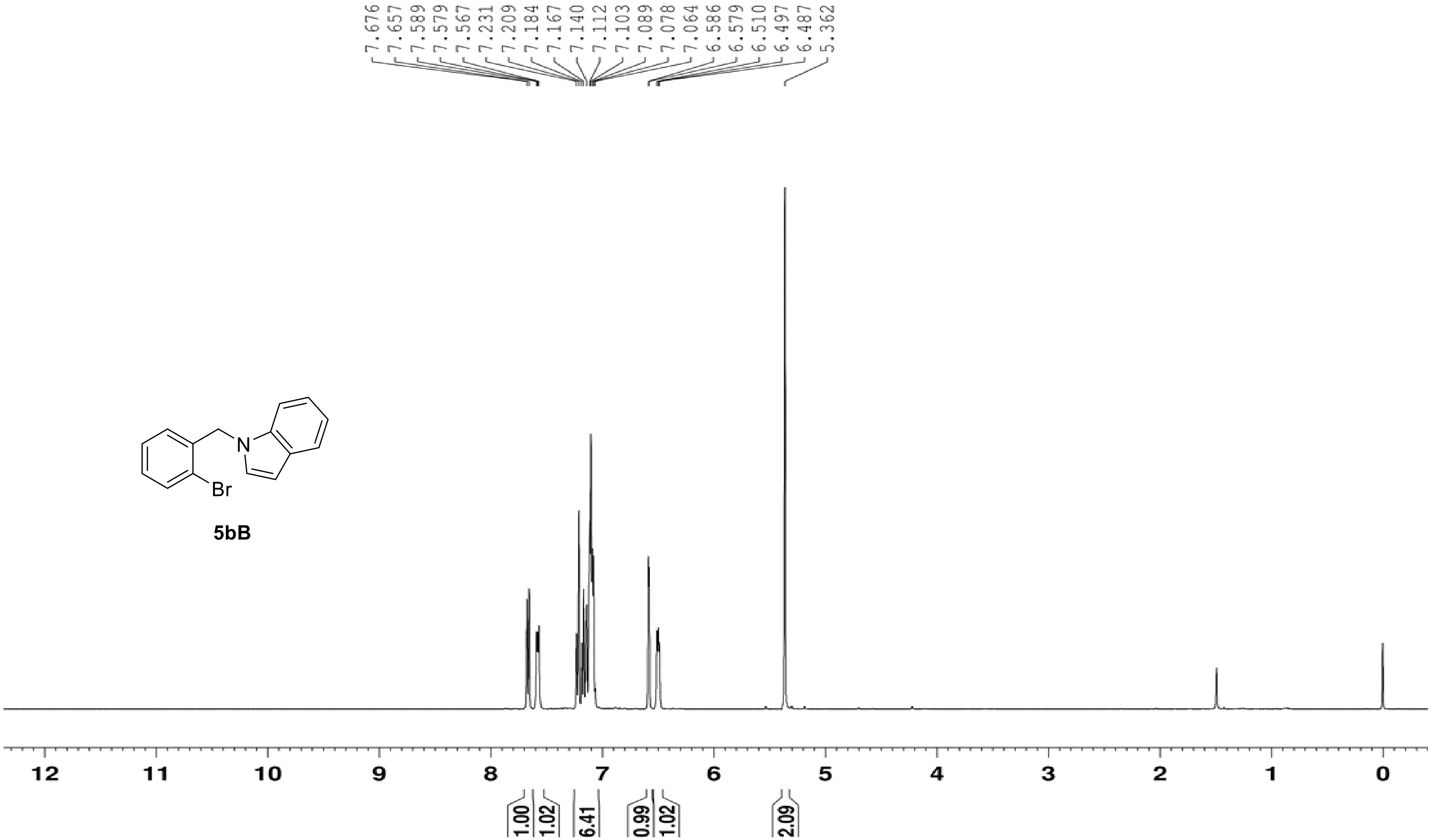

Supplementary Figure 5. ^{1}H NMR spectrum of **5bB**.

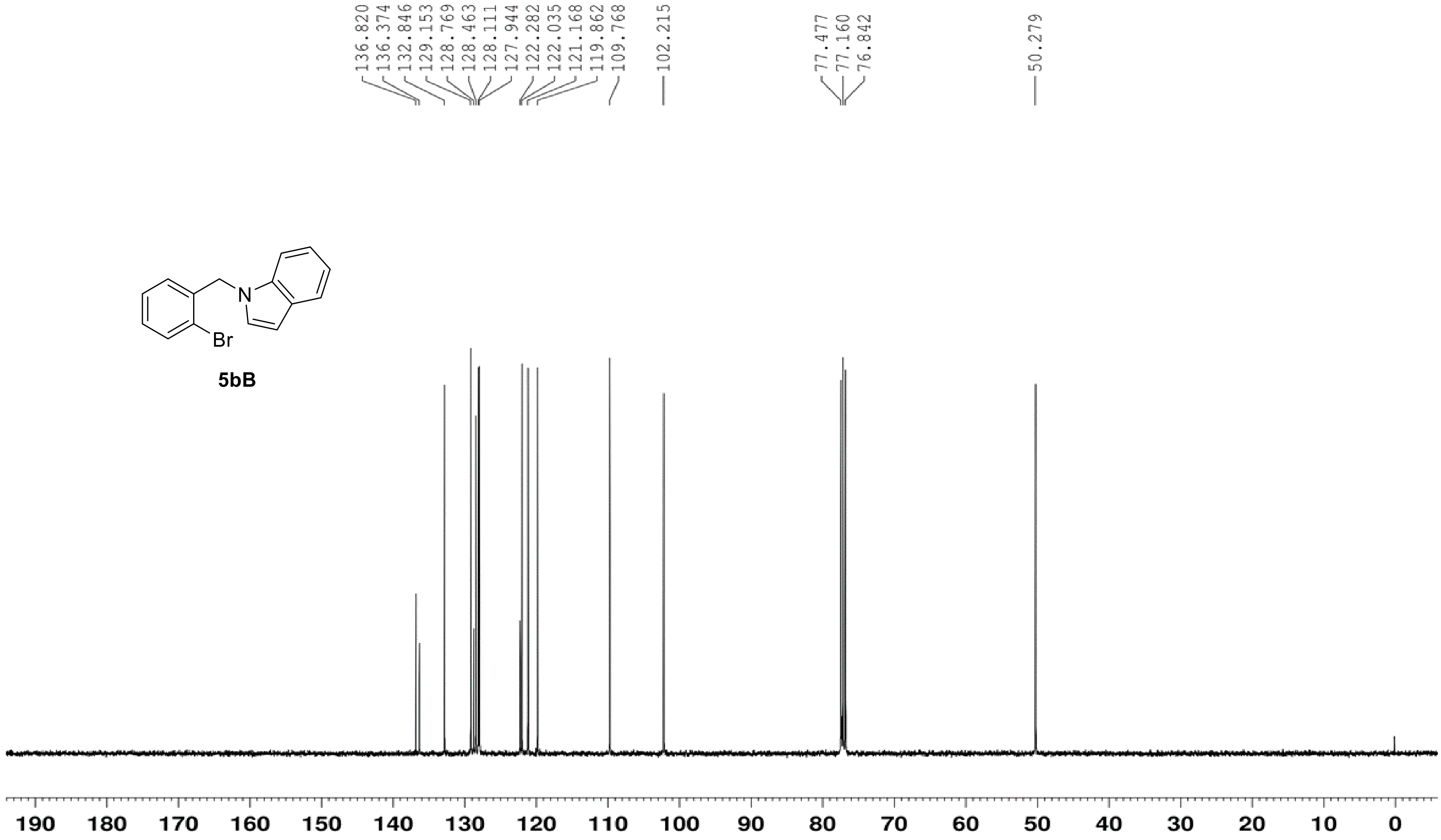

Supplementary Figure 6. ^{13}C NMR spectrum of **5bB**.

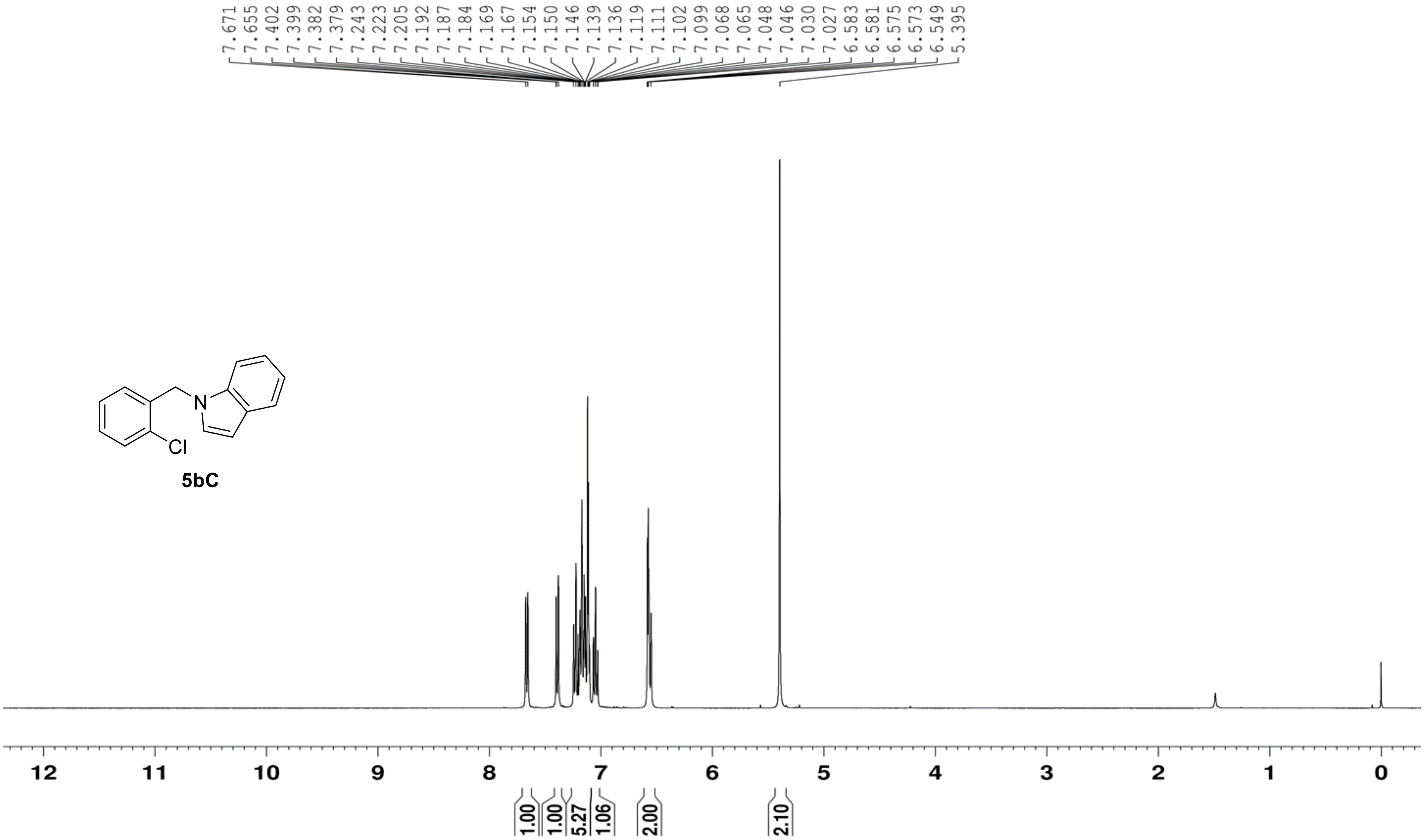

Supplementary Figure 7. ^{1}H NMR spectrum of **5bC**.

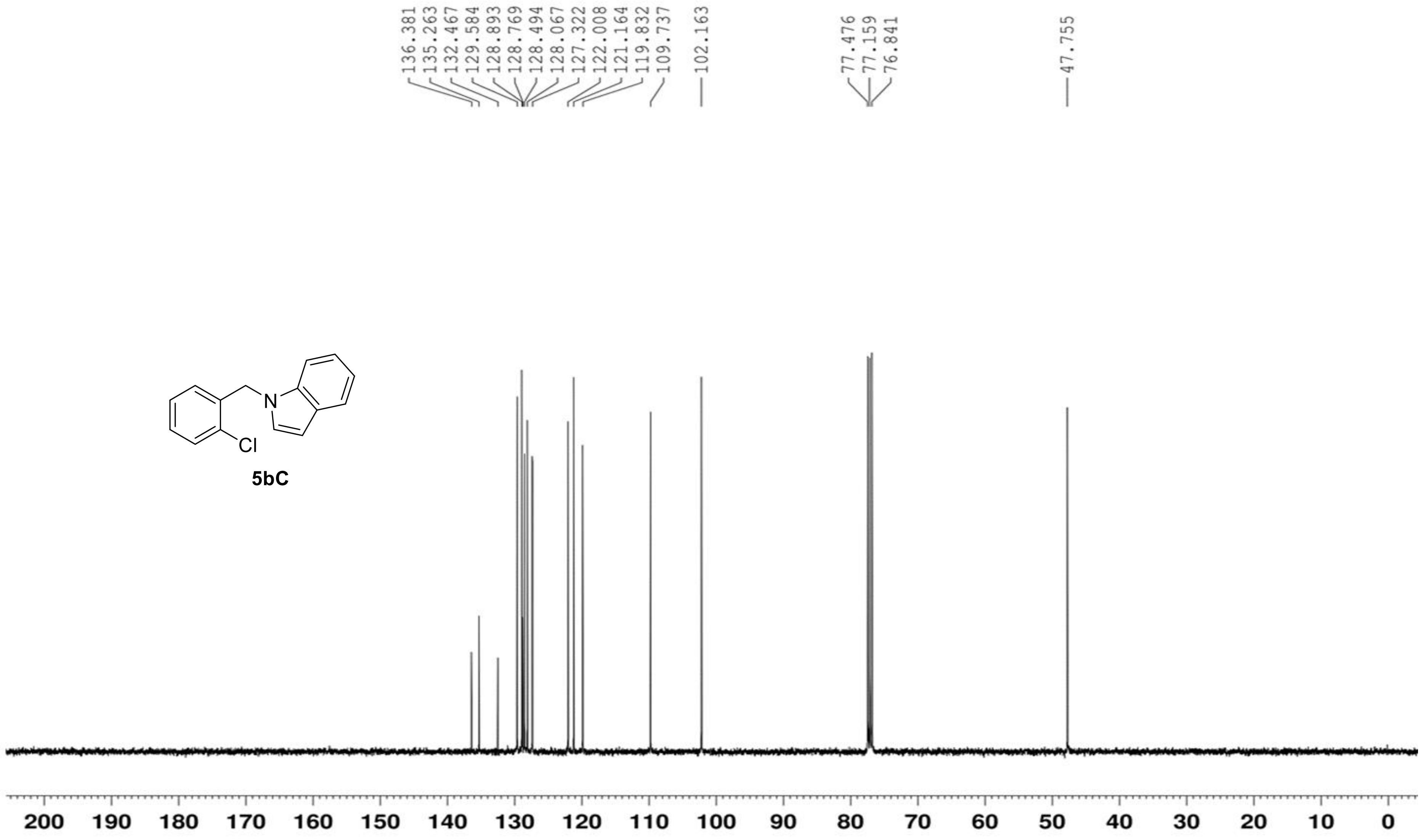

Supplementary Figure 8. ^{13}C NMR spectrum of **5bC**.

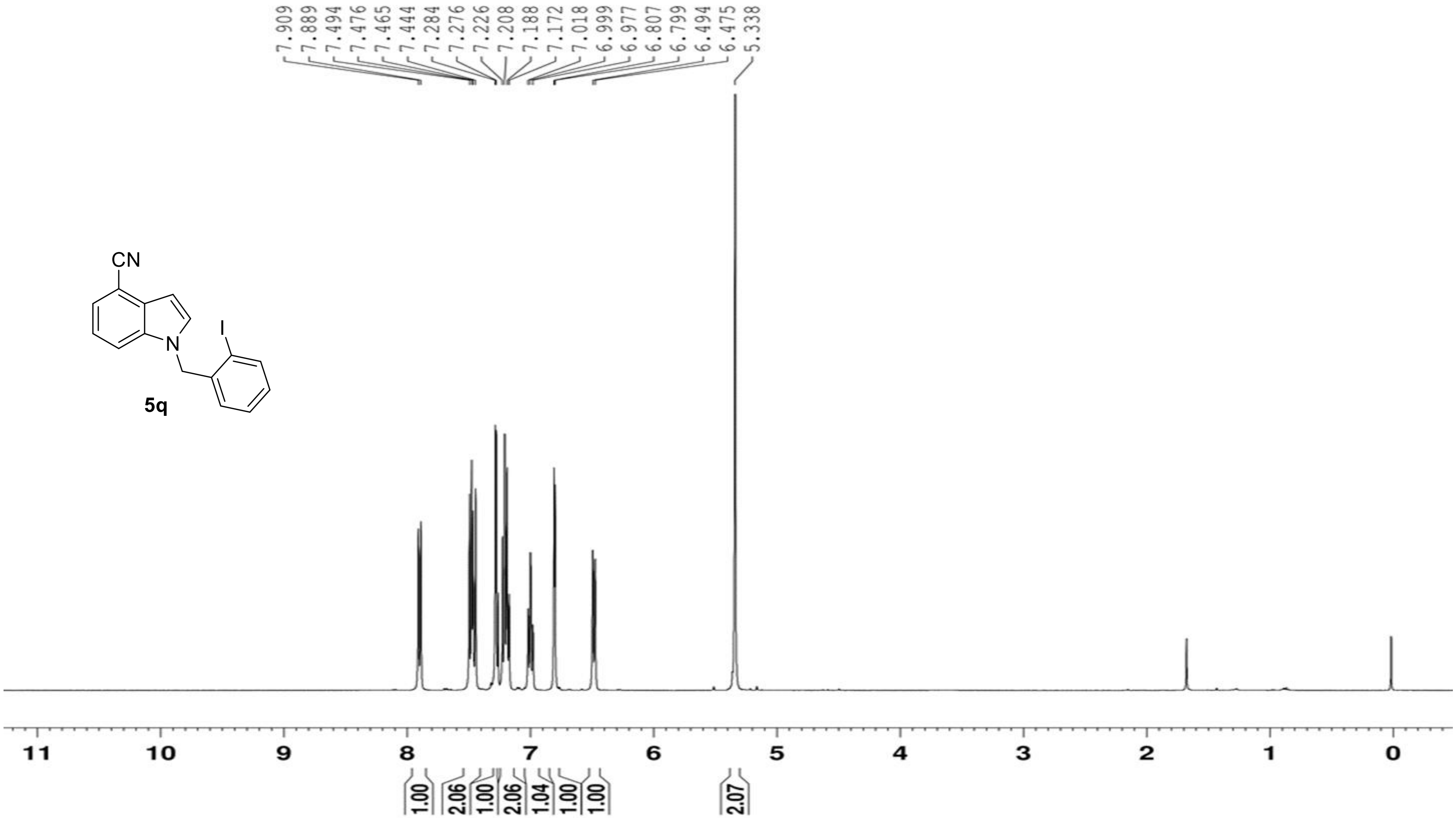

Supplementary Figure 9. ^{1}H NMR spectrum of **5q**.

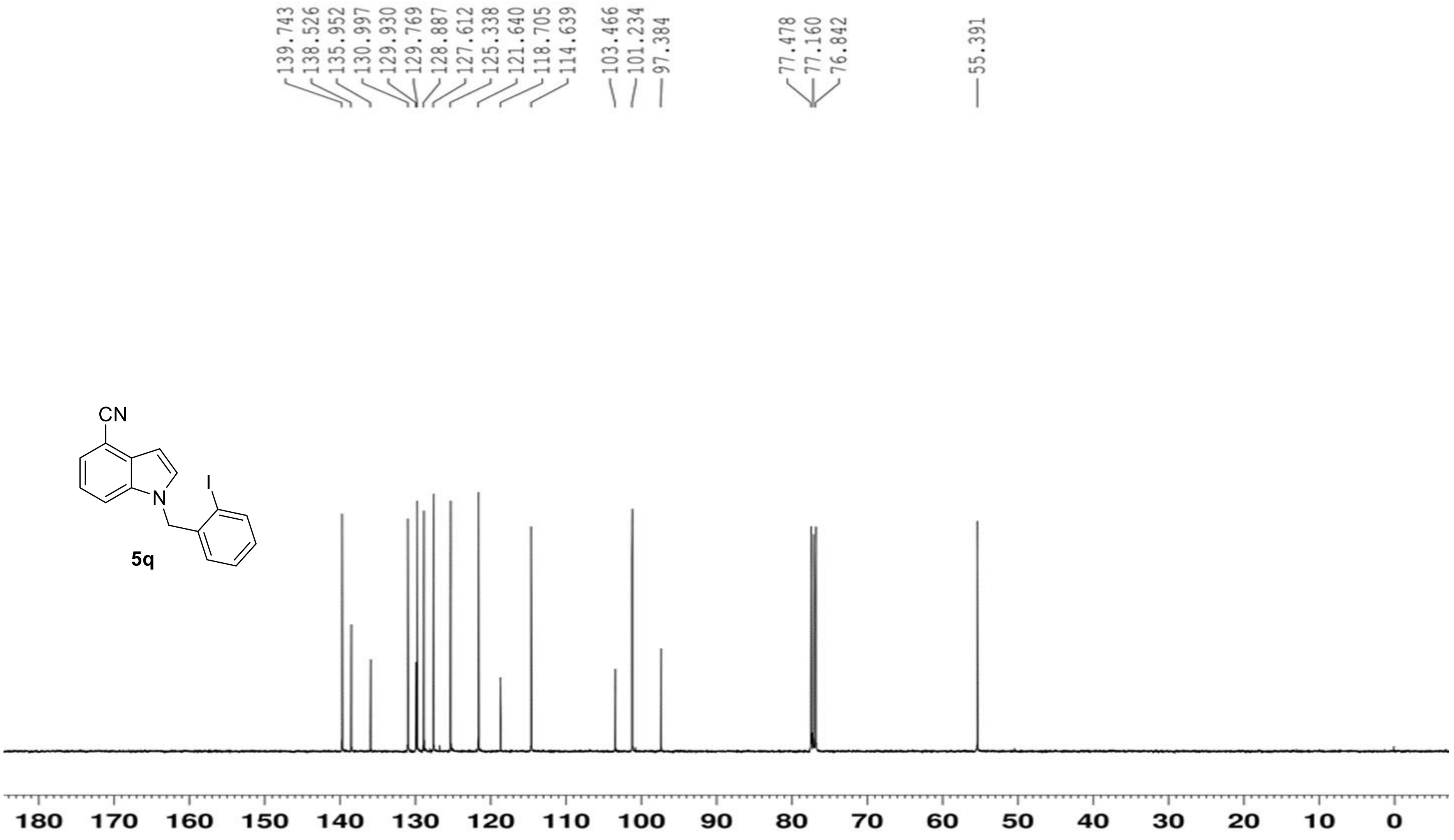

Supplementary Figure 10. ^{13}C NMR spectrum of **5q**.

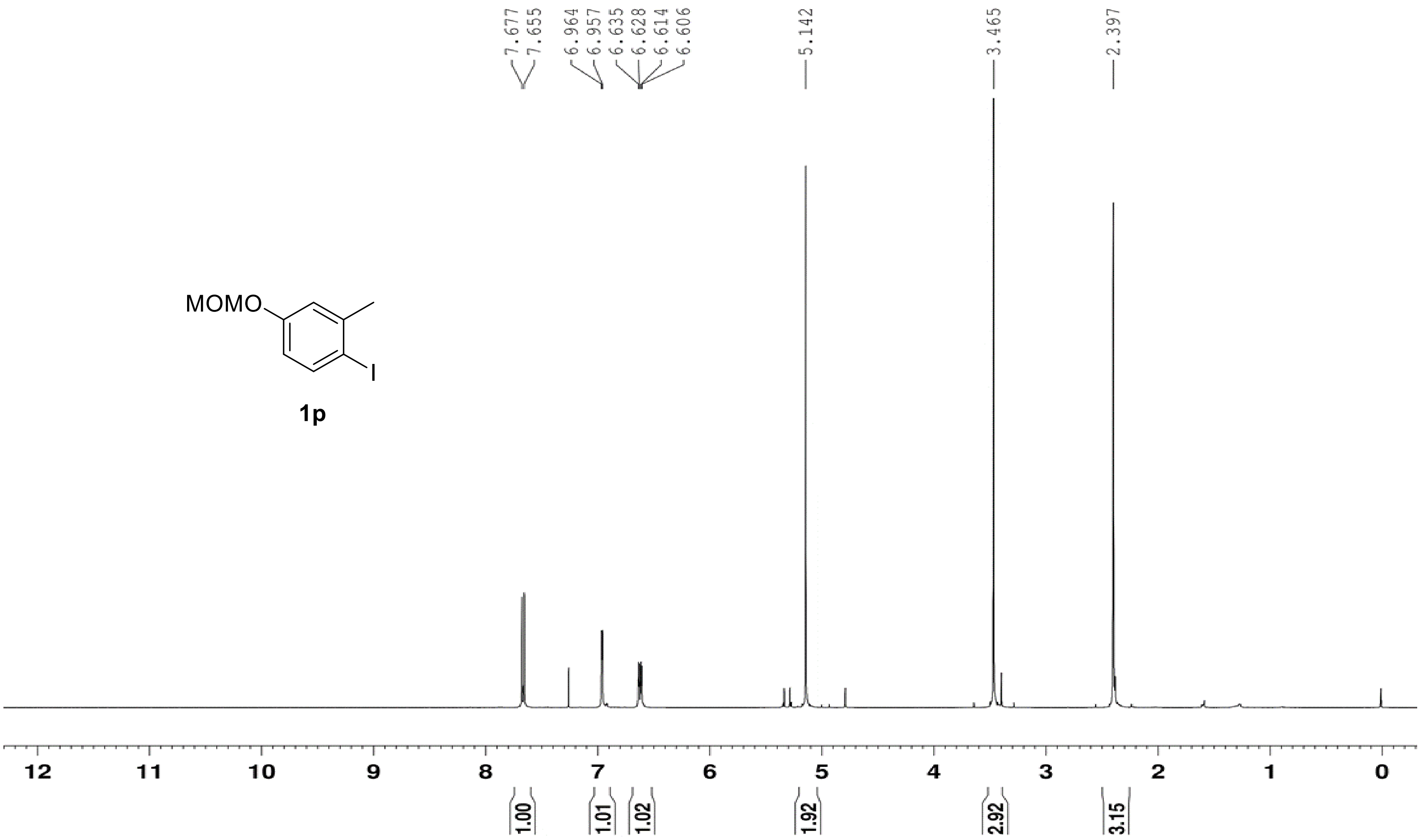

Supplementary Figure 11. ^{1}H NMR spectrum of **1p**.

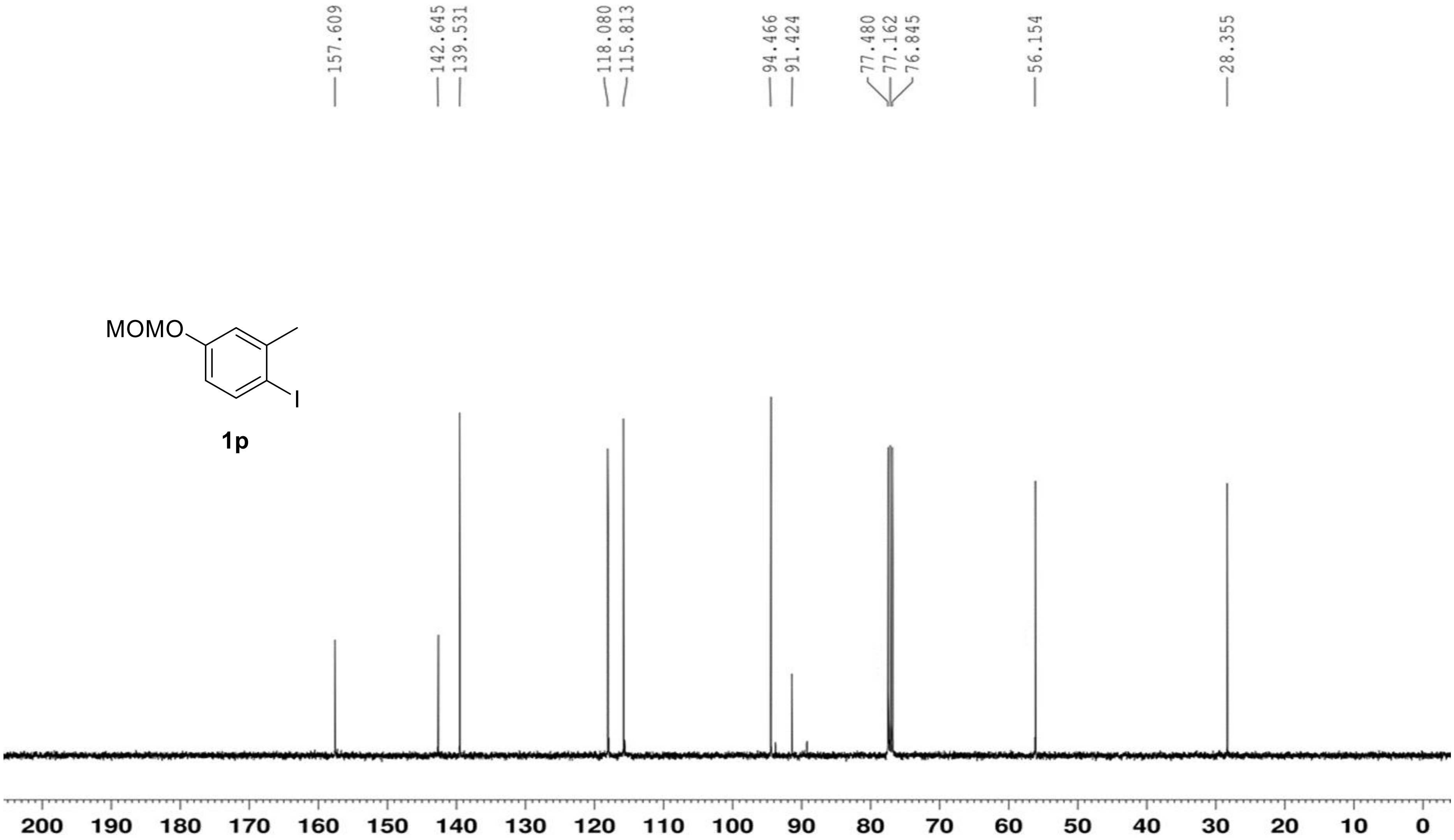

Supplementary Figure 12. ^{13}C NMR spectrum of **1p**.

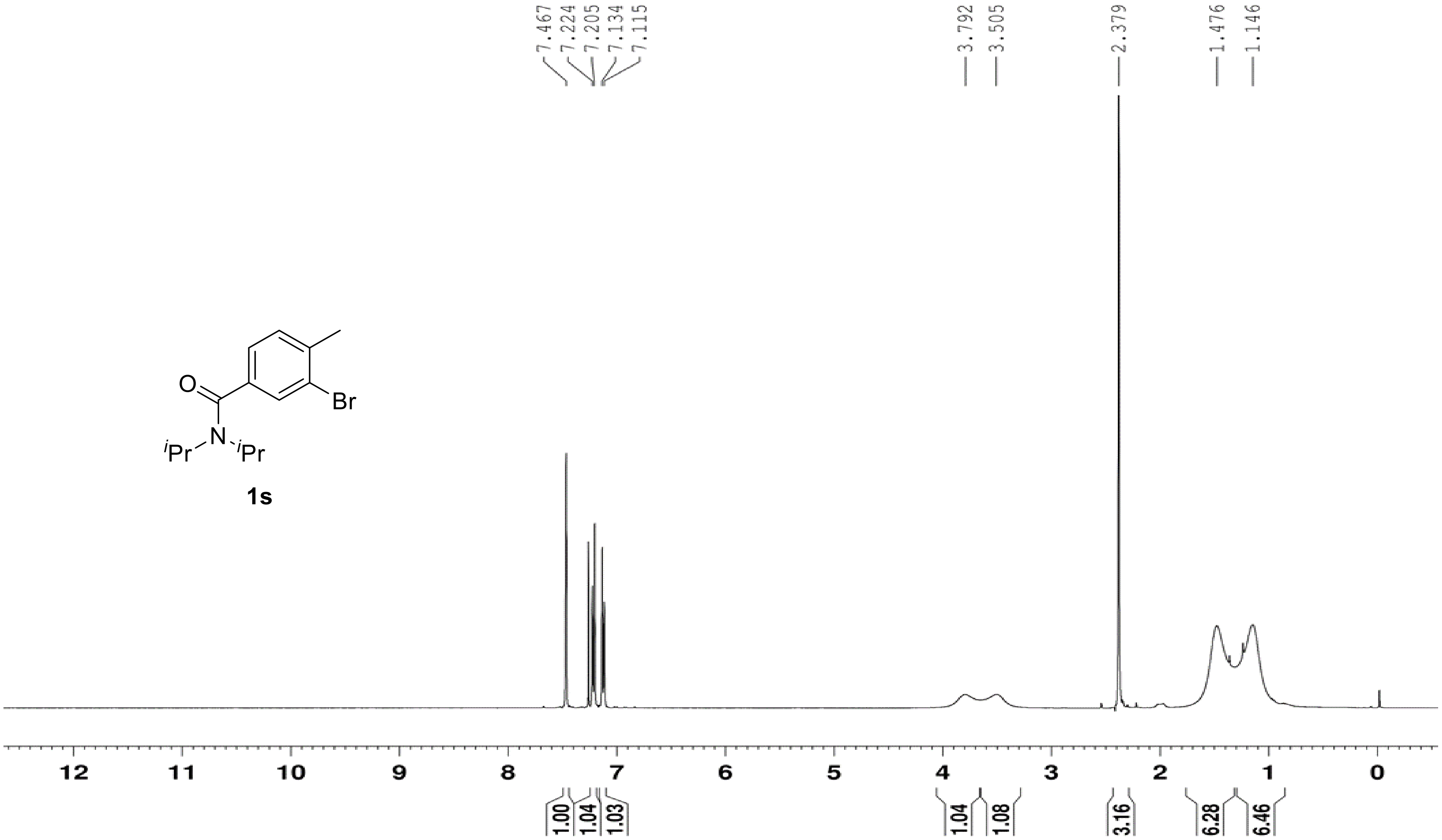

Supplementary Figure 13. ^{1}H NMR spectrum of **1s**.

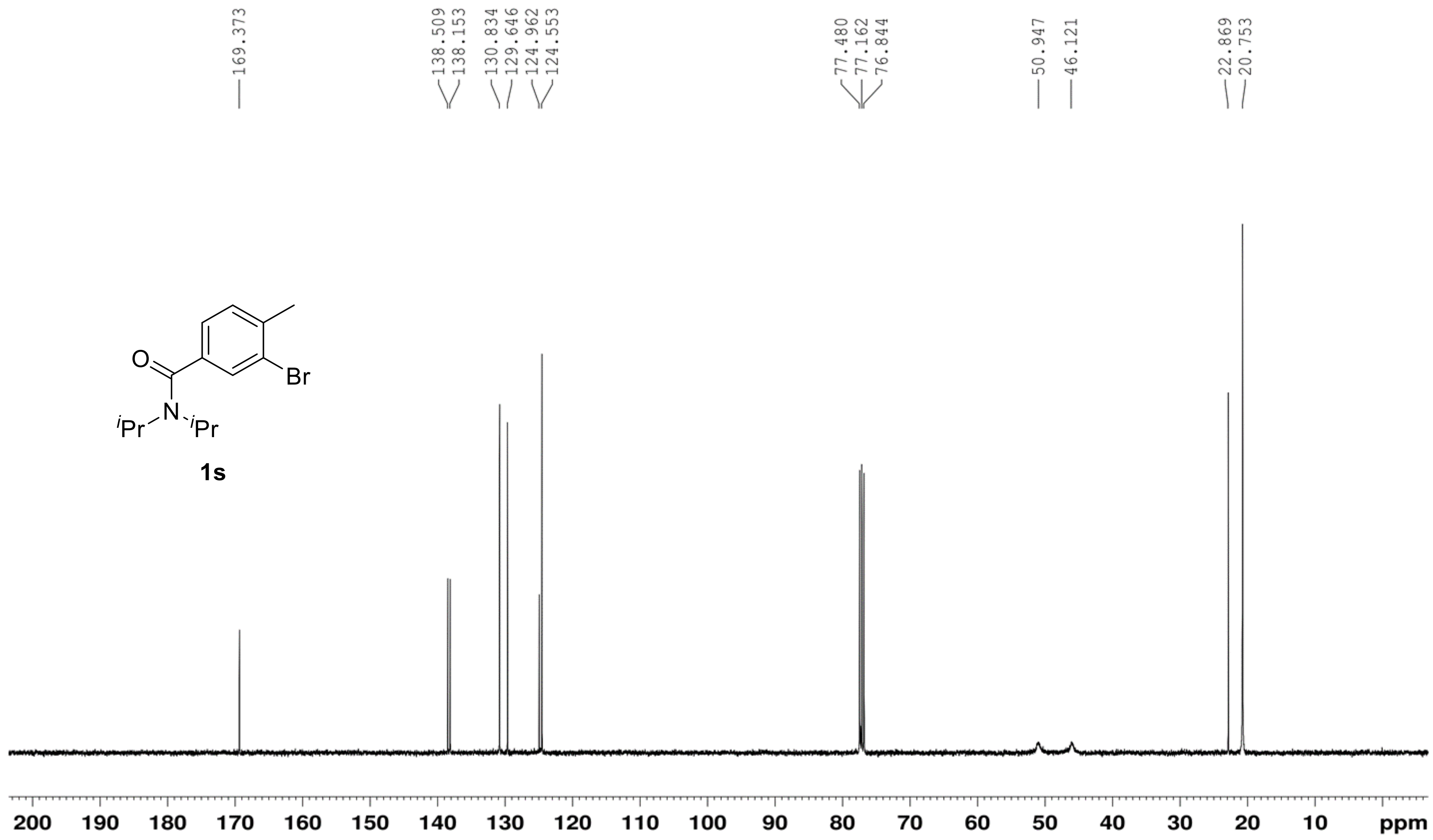

Supplementary Figure 14. ^{13}C NMR spectrum of **1s**.

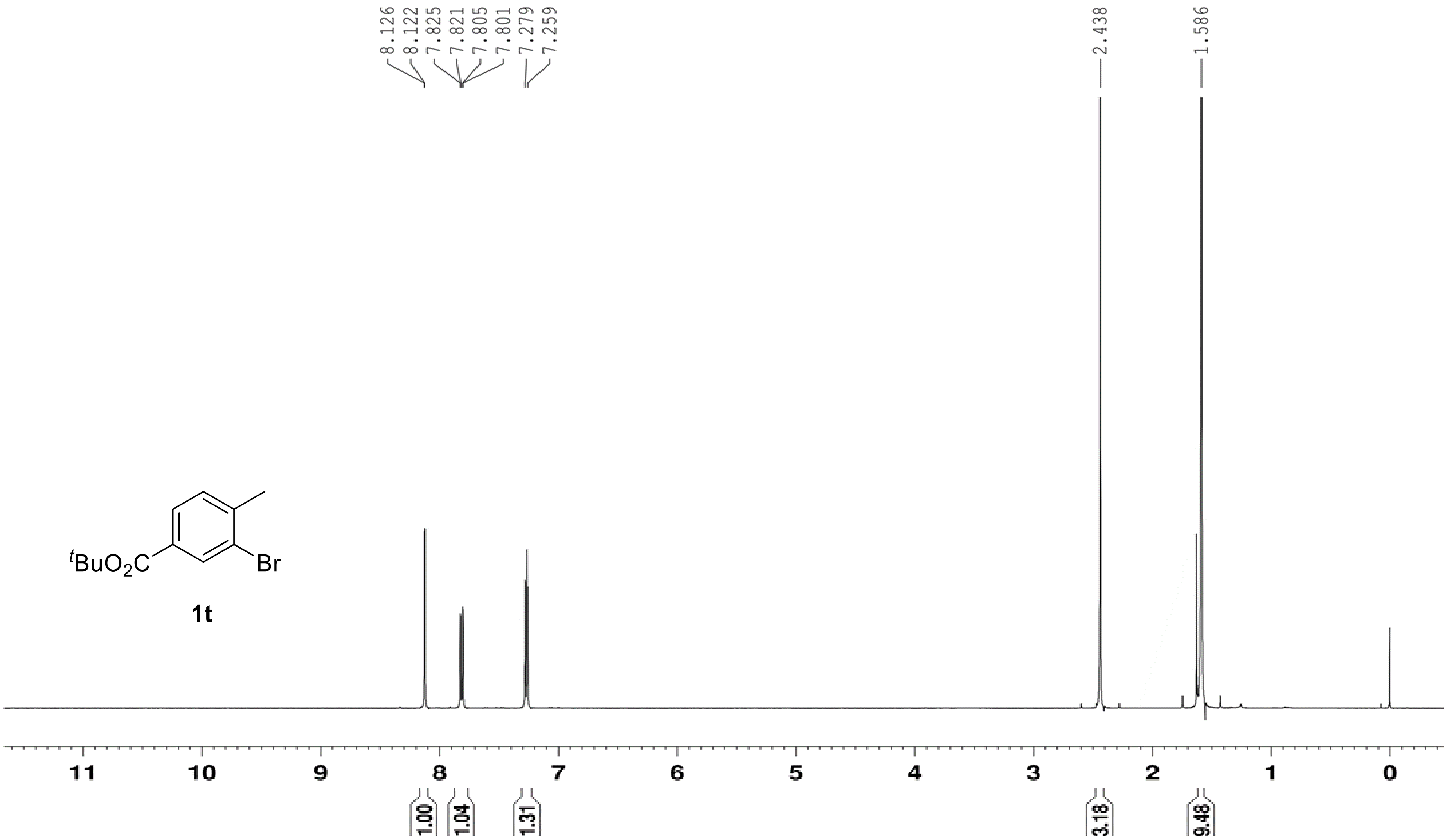

Supplementary Figure 15. ^{1}H NMR spectrum of **1t**.

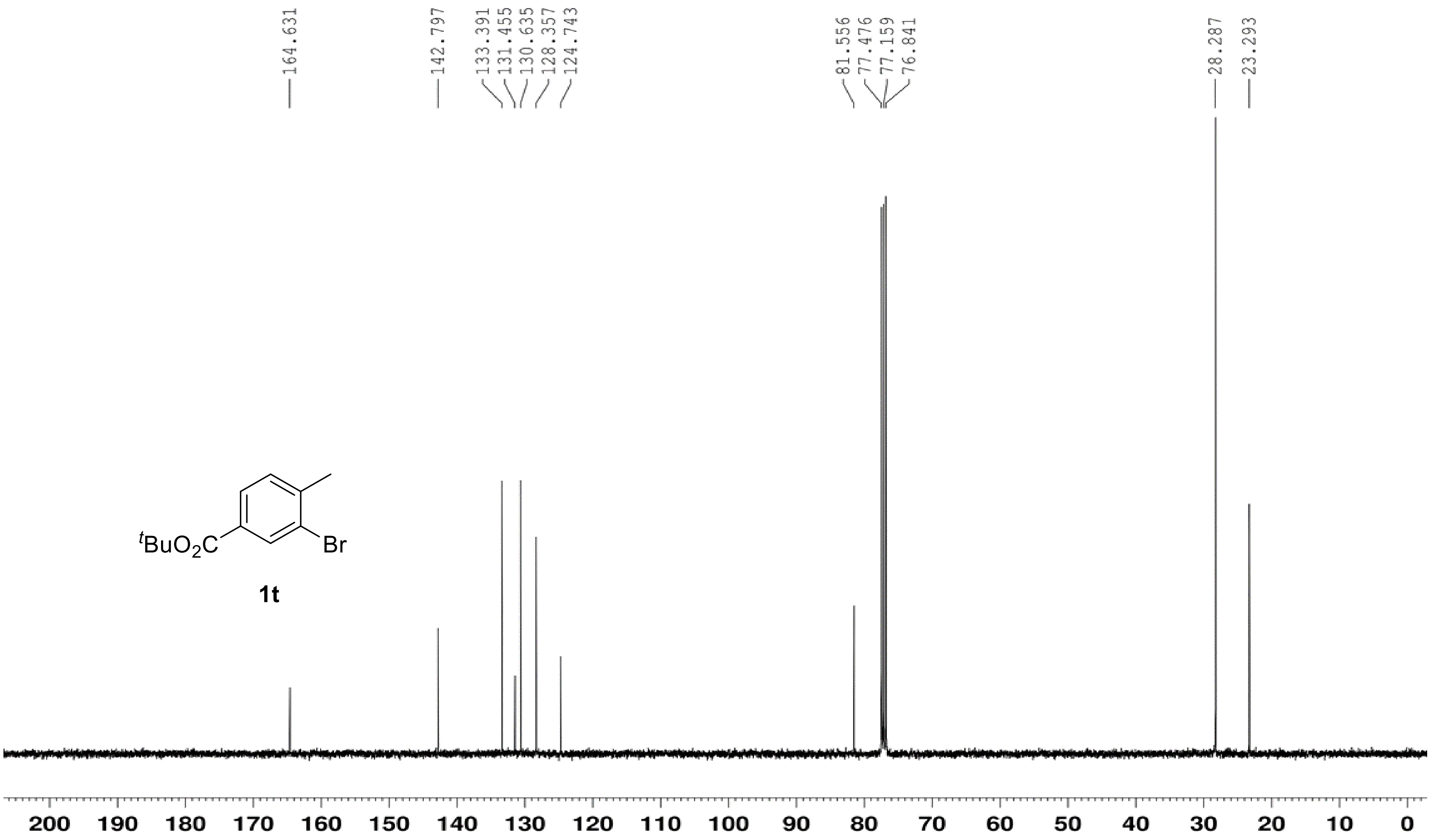

Supplementary Figure 16. ^{13}C NMR spectrum of **1t**.

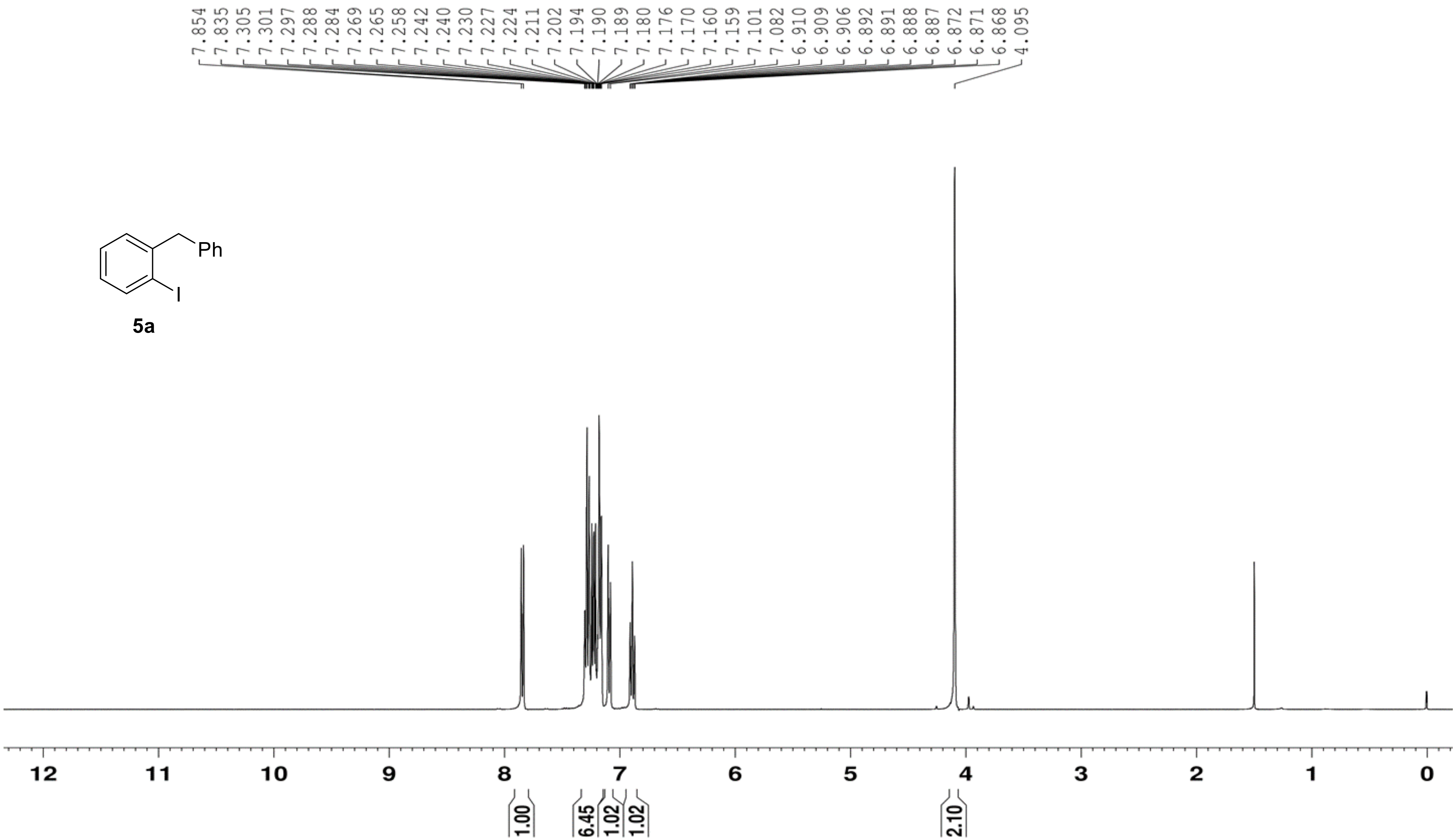

Supplementary Figure 17. ^{1}H NMR spectrum of **5a**.

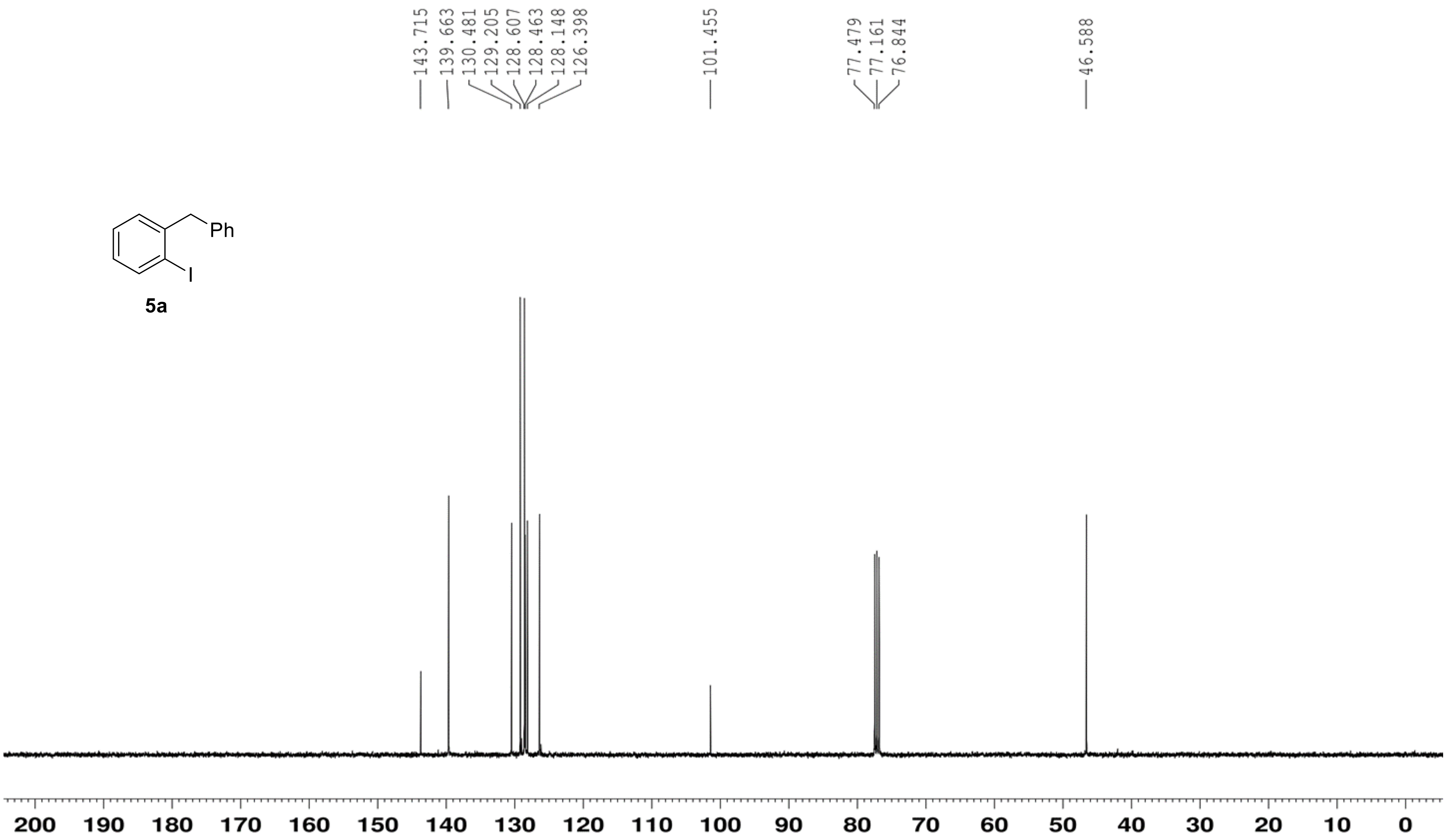

Supplementary Figure 18. ^{13}C NMR spectrum of **5a**.

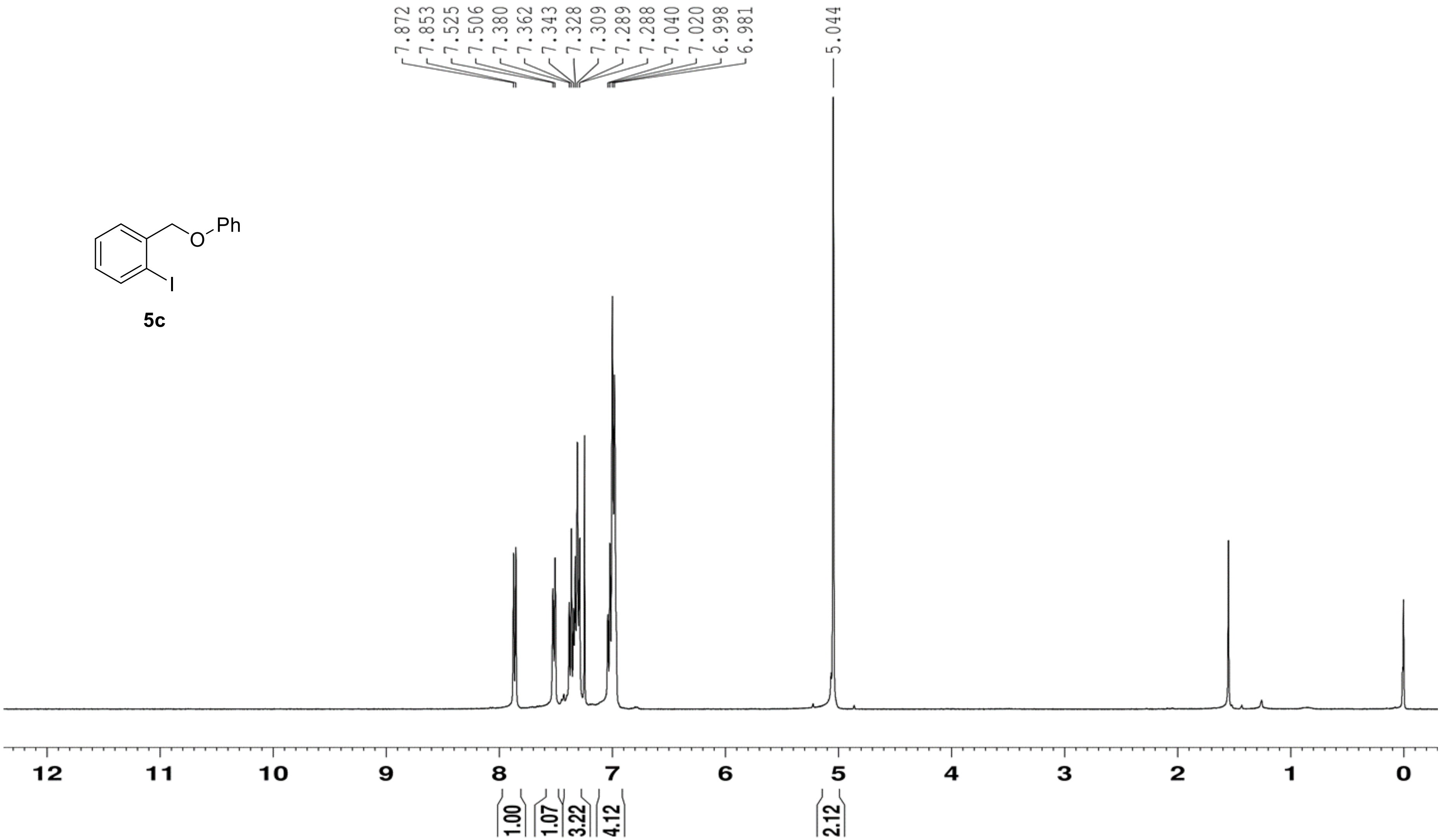

Supplementary Figure 19. ^{1}H NMR spectrum of **5c**.

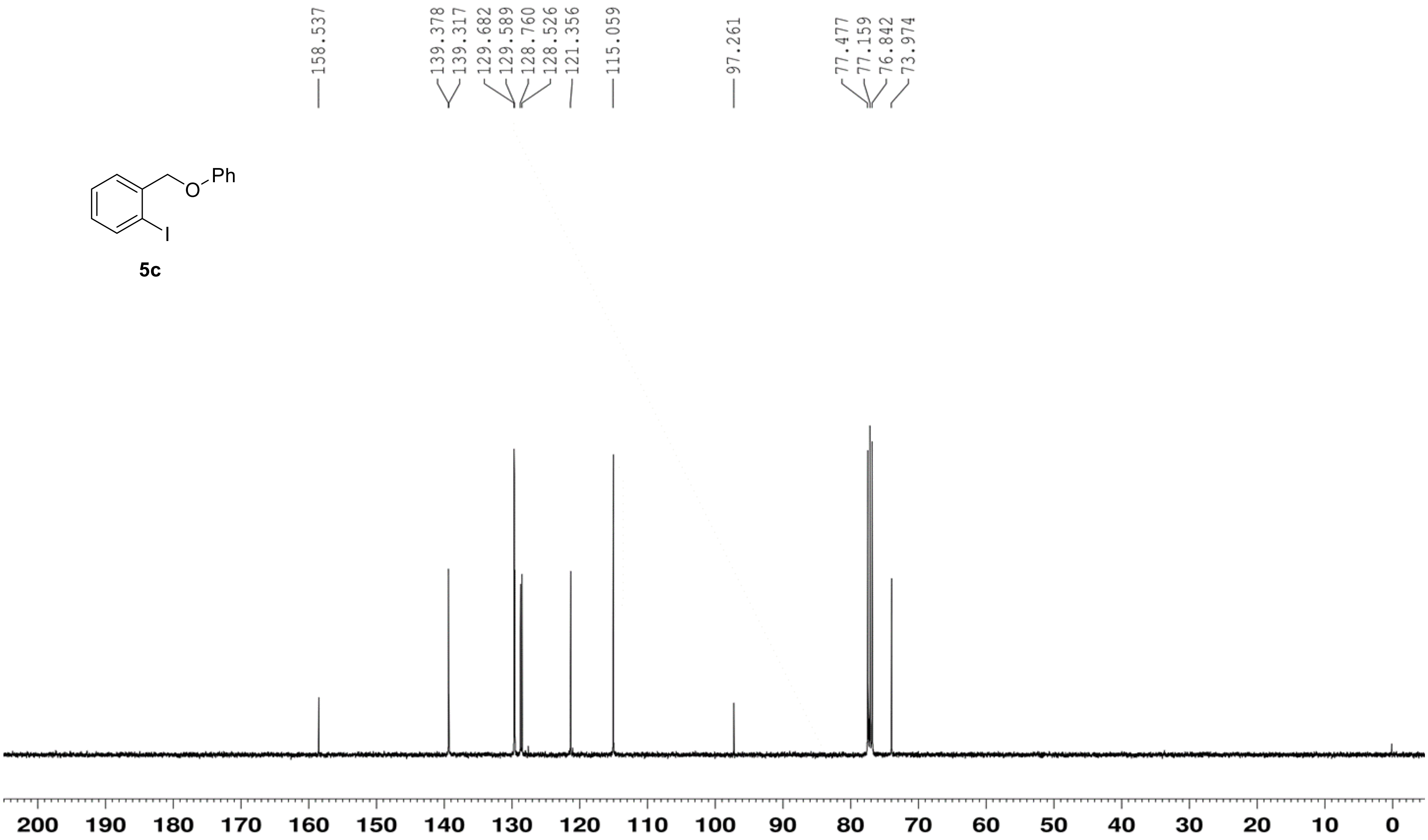

Supplementary Figure 20. ^{13}C NMR spectrum of **5c**.

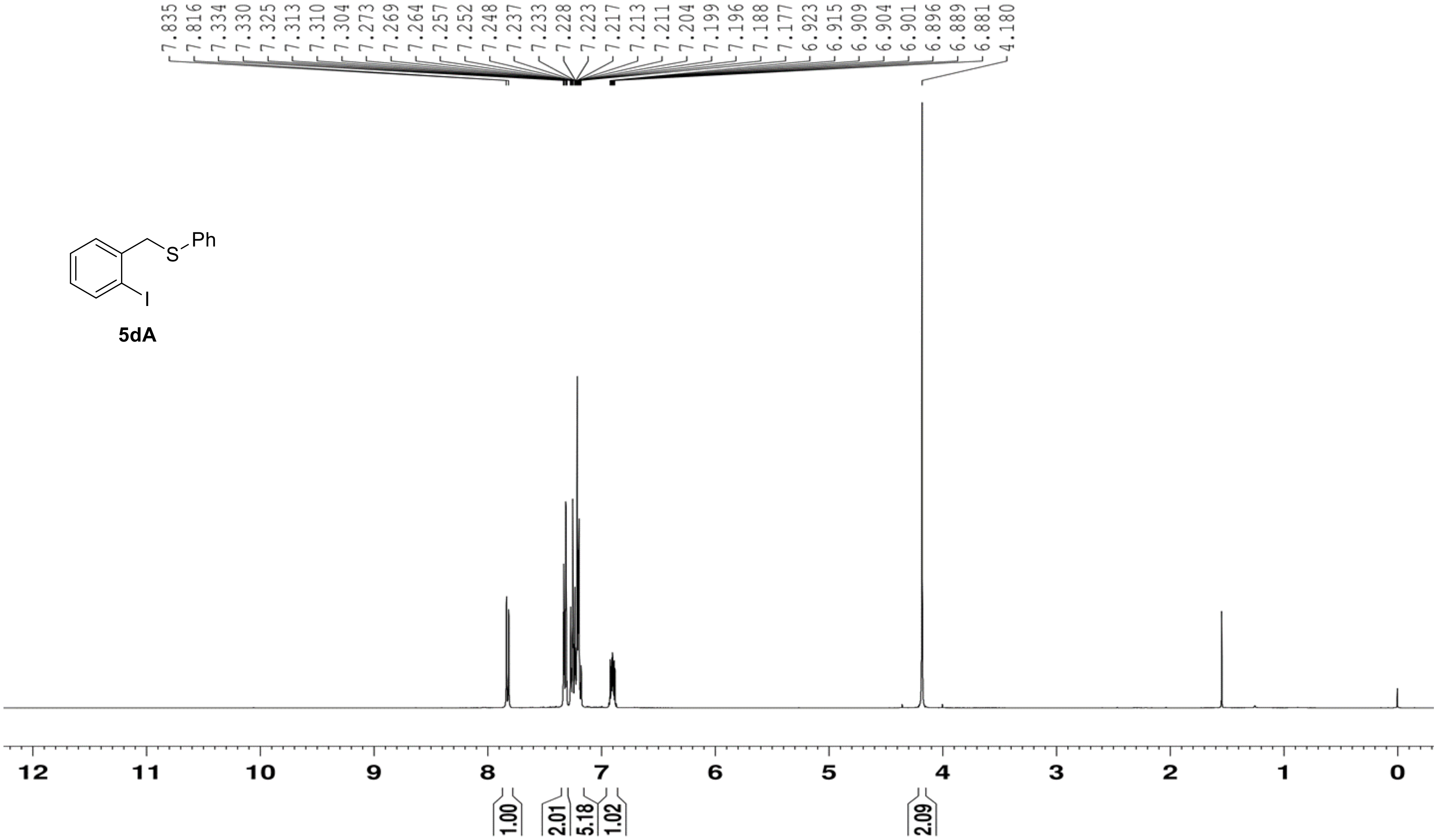

5dA

Supplementary Figure 21. ^{1}H NMR spectrum of **5dA**.

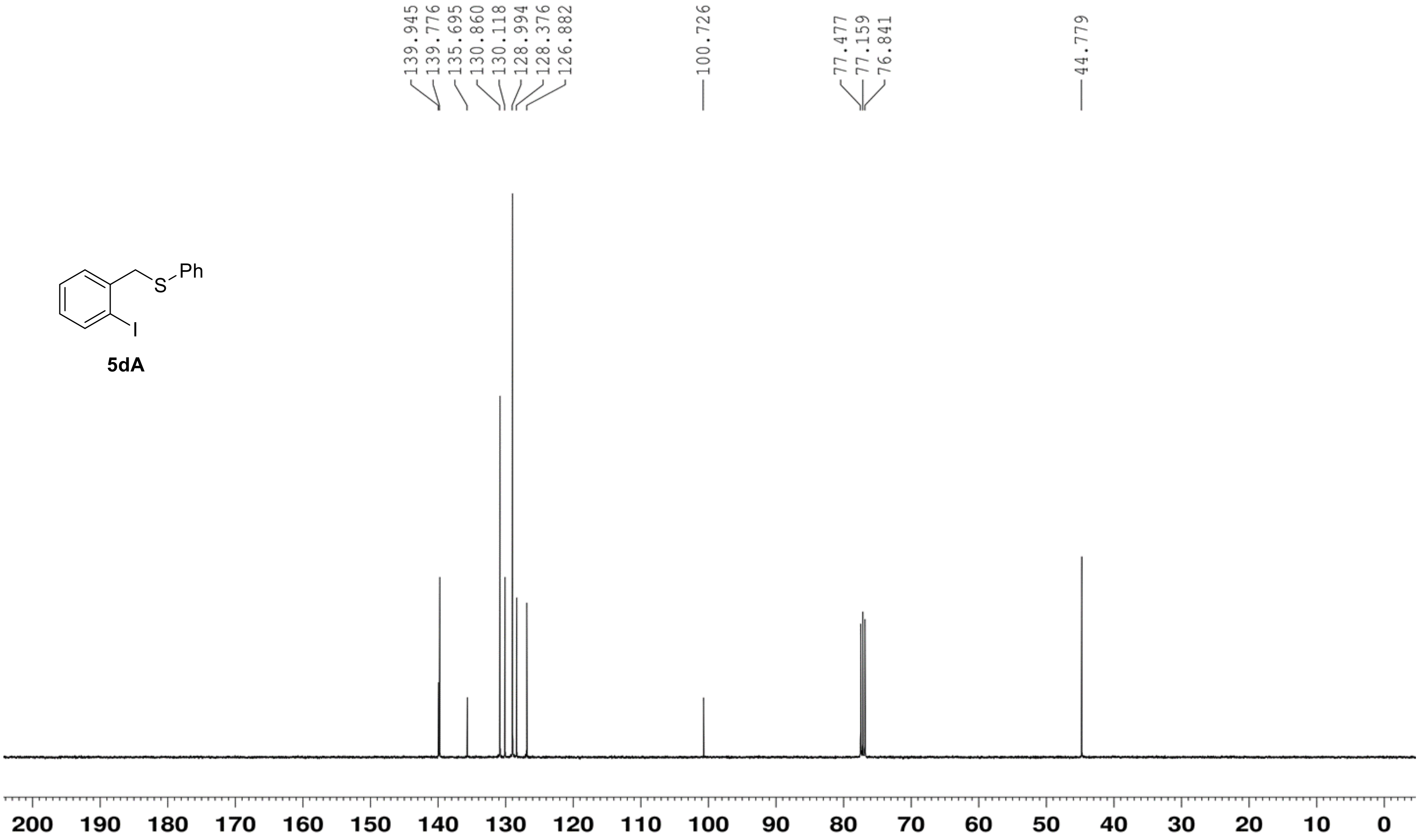

Supplementary Figure 22. ^{13}C NMR spectrum of **5dA**.

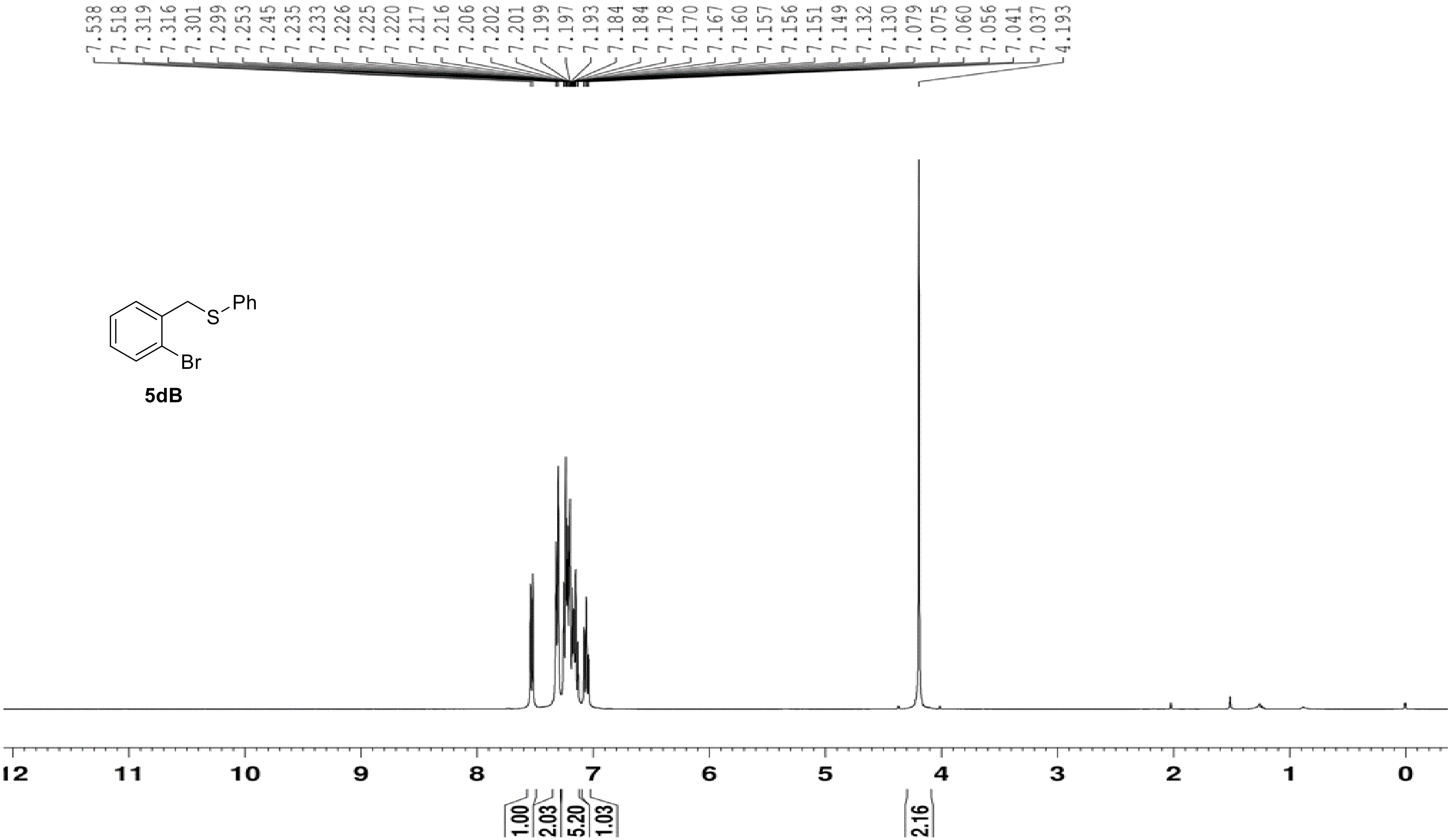

Supplementary Figure 23. ^{1}H NMR spectrum of **5dB**.

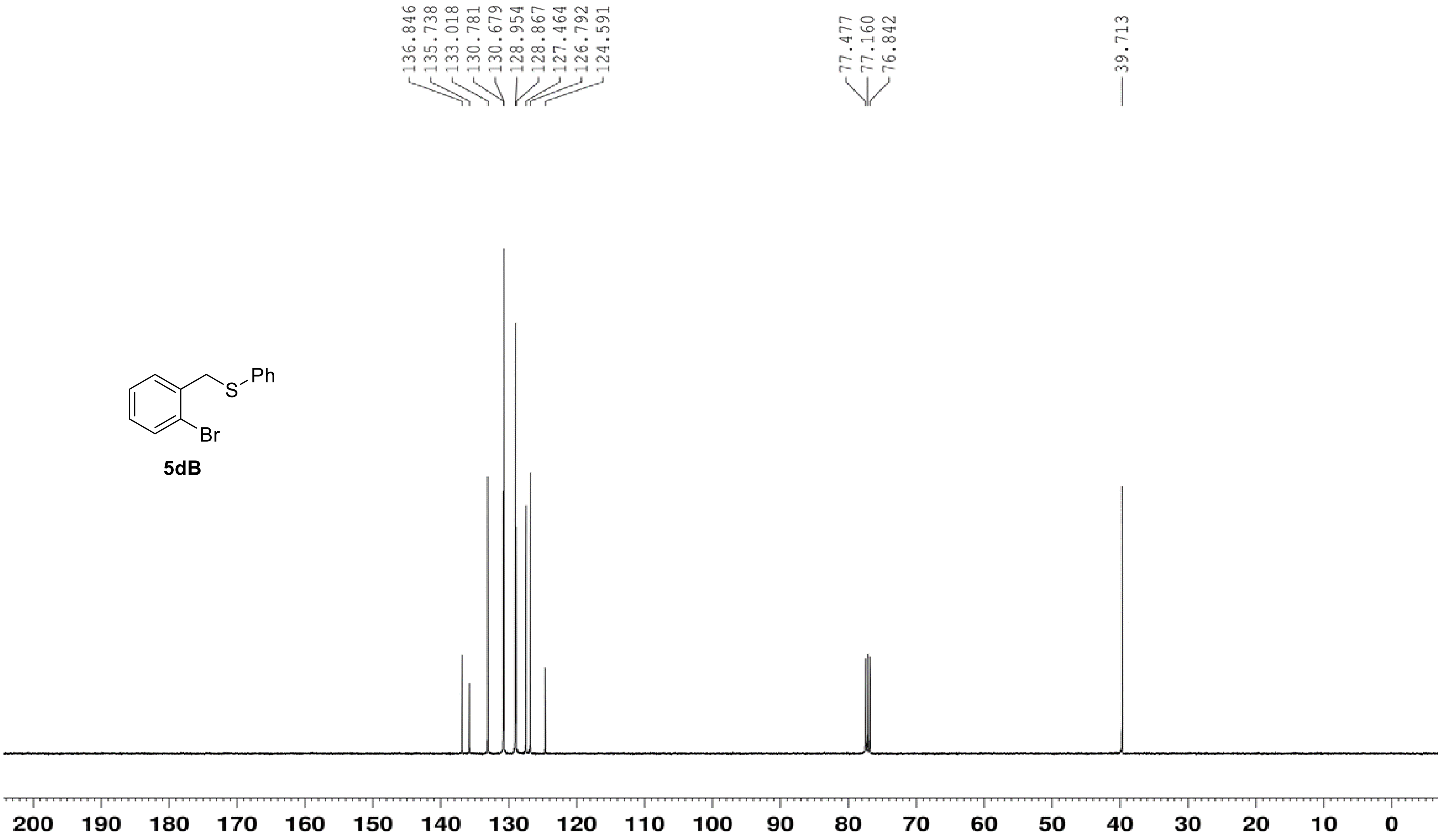

Supplementary Figure 24. ^{13}C NMR spectrum of **5dB**.

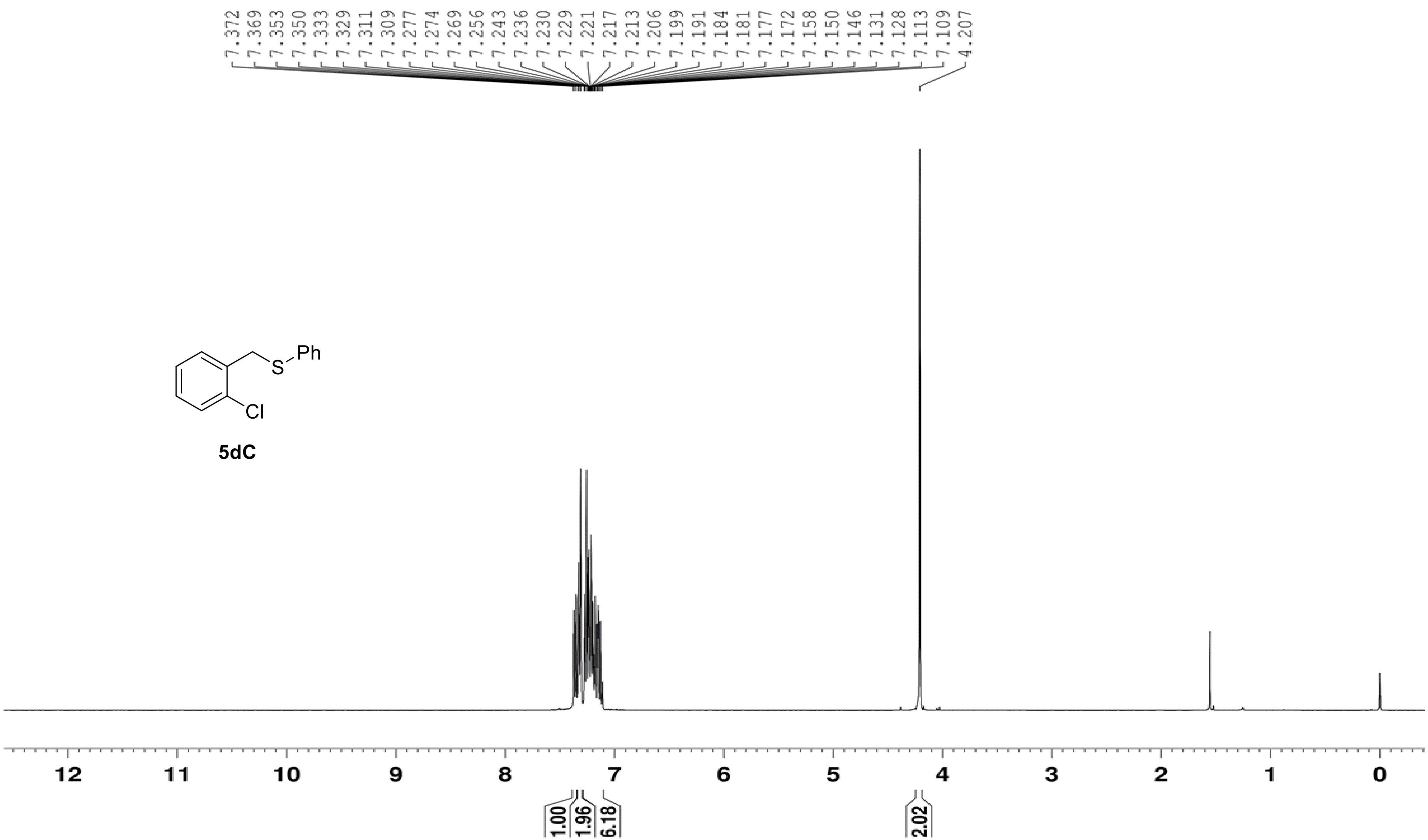

5dC

Supplementary Figure 25. ^{1}H NMR spectrum of **5dC**.

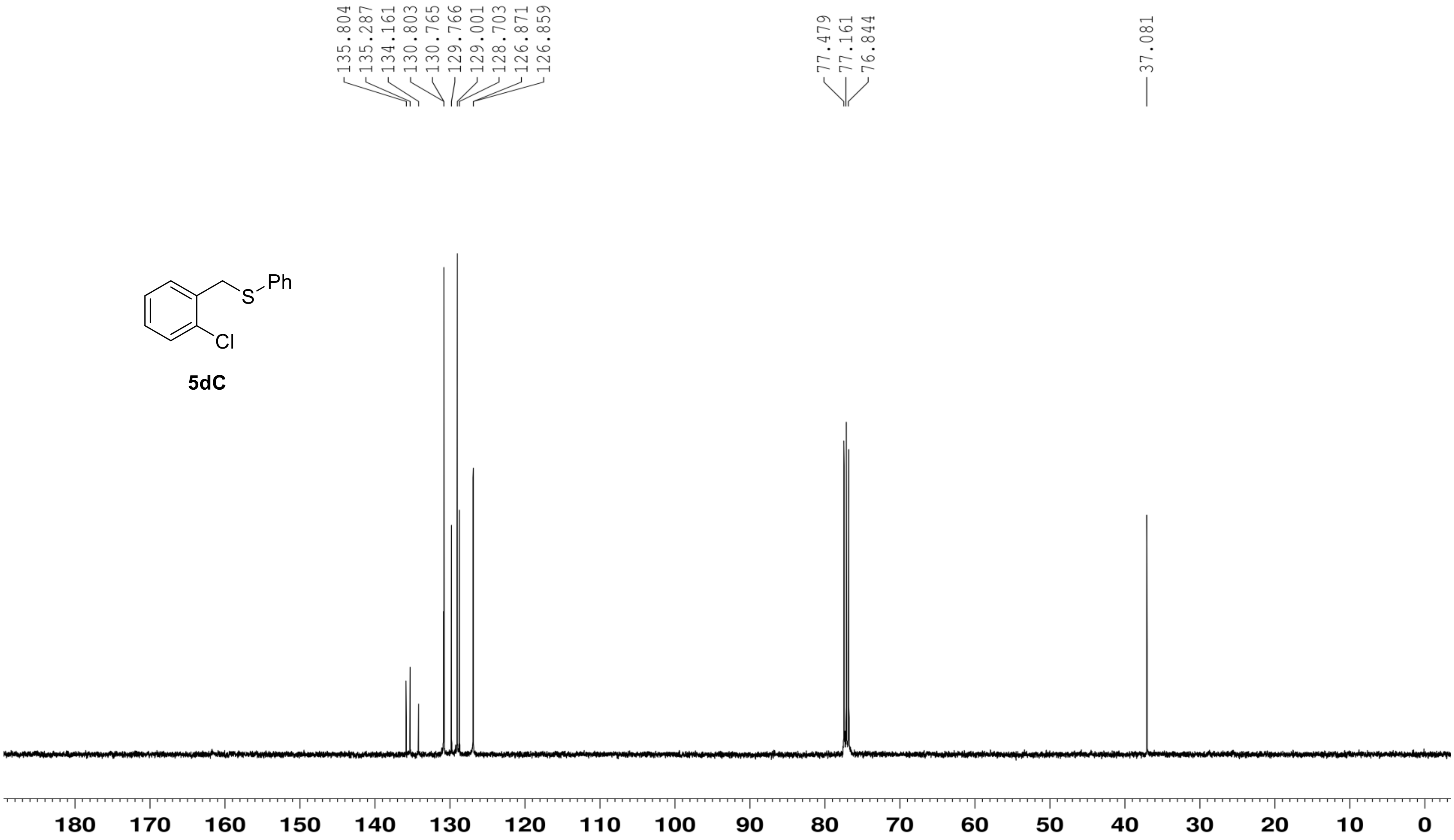

Supplementary Figure 26. ^{13}C NMR spectrum of **5dC**.

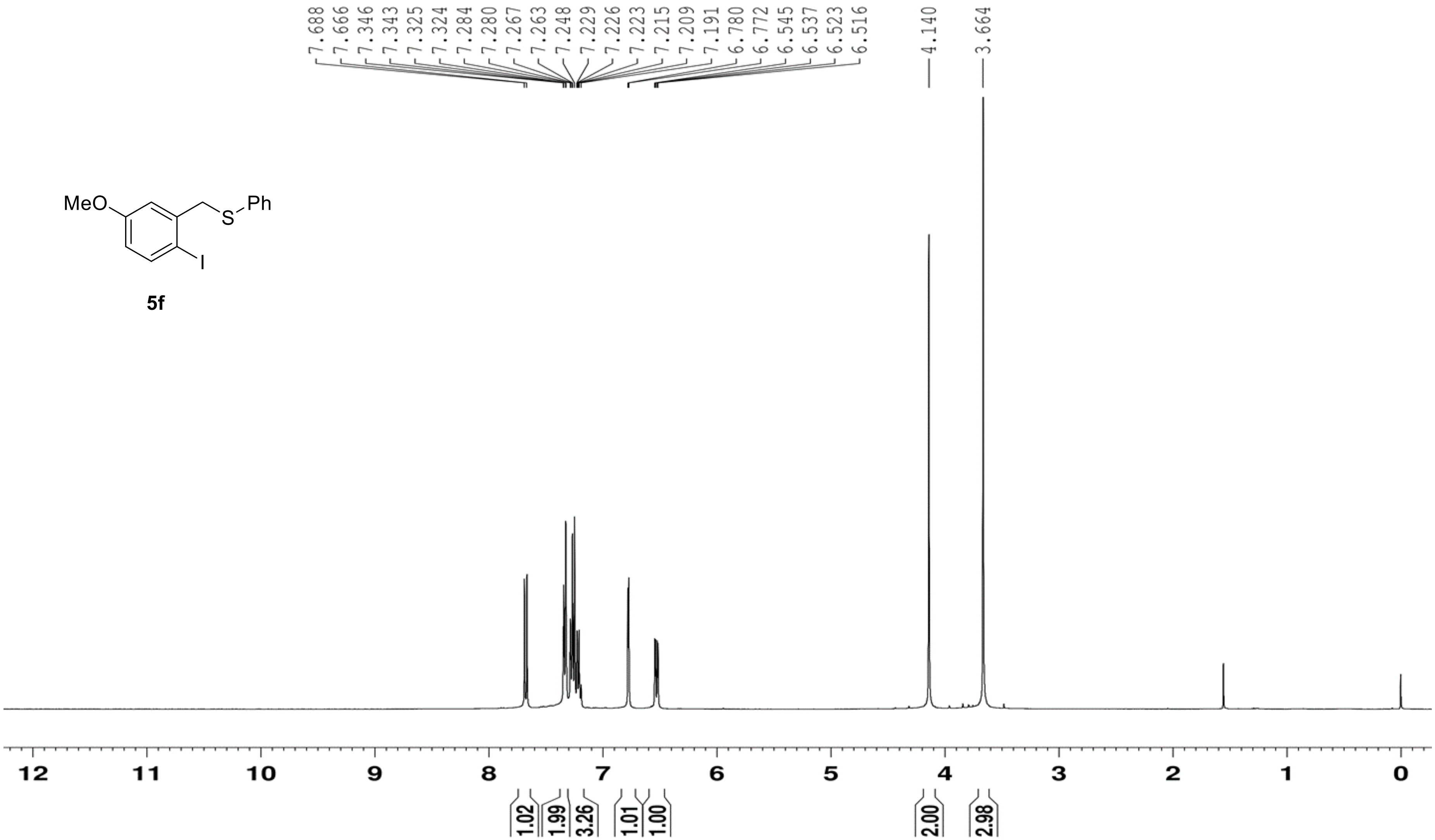

Supplementary Figure 27. ^{1}H NMR spectrum of **5f**.

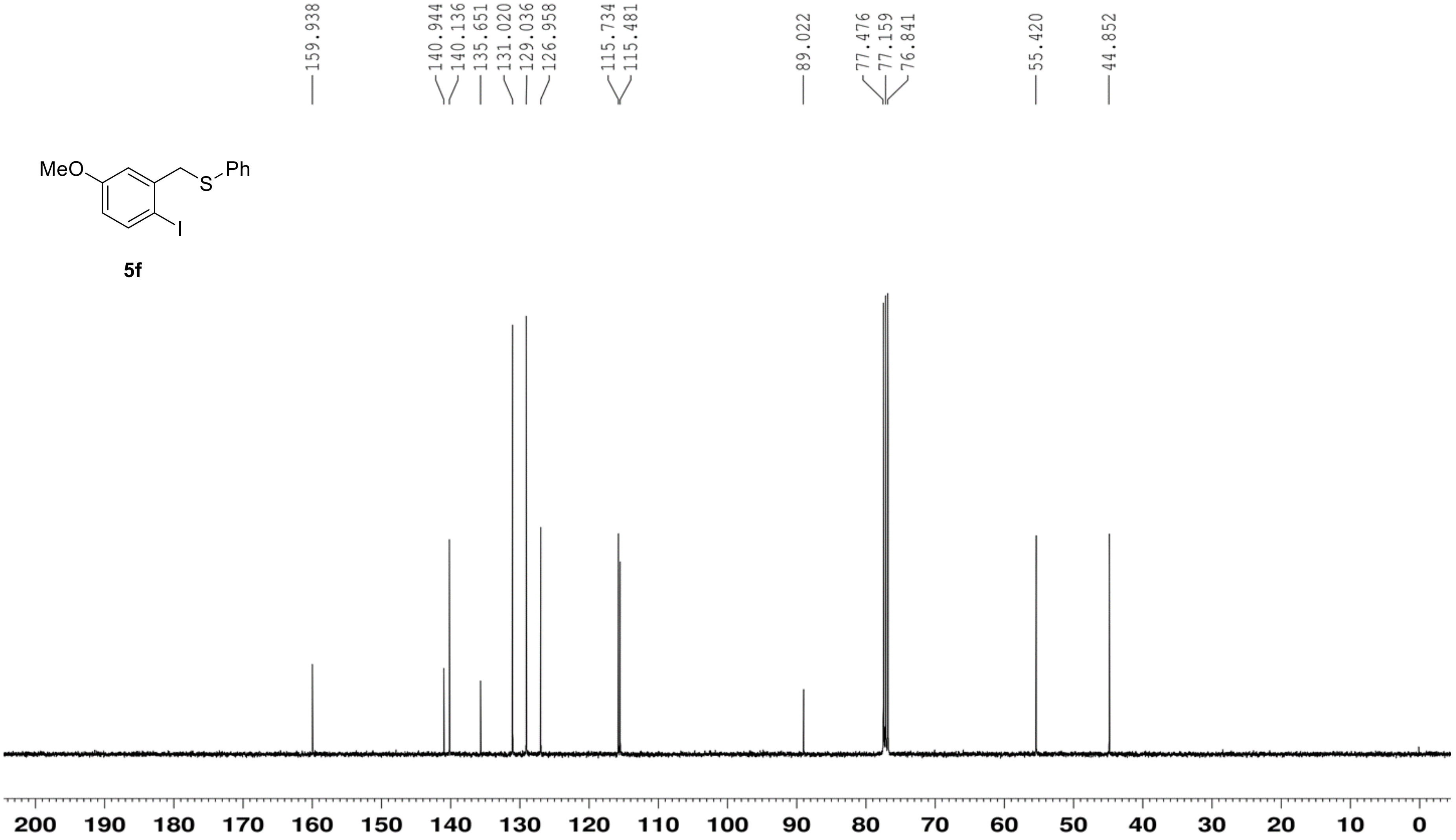

Supplementary Figure 28. ^{13}C NMR spectrum of **5f**.

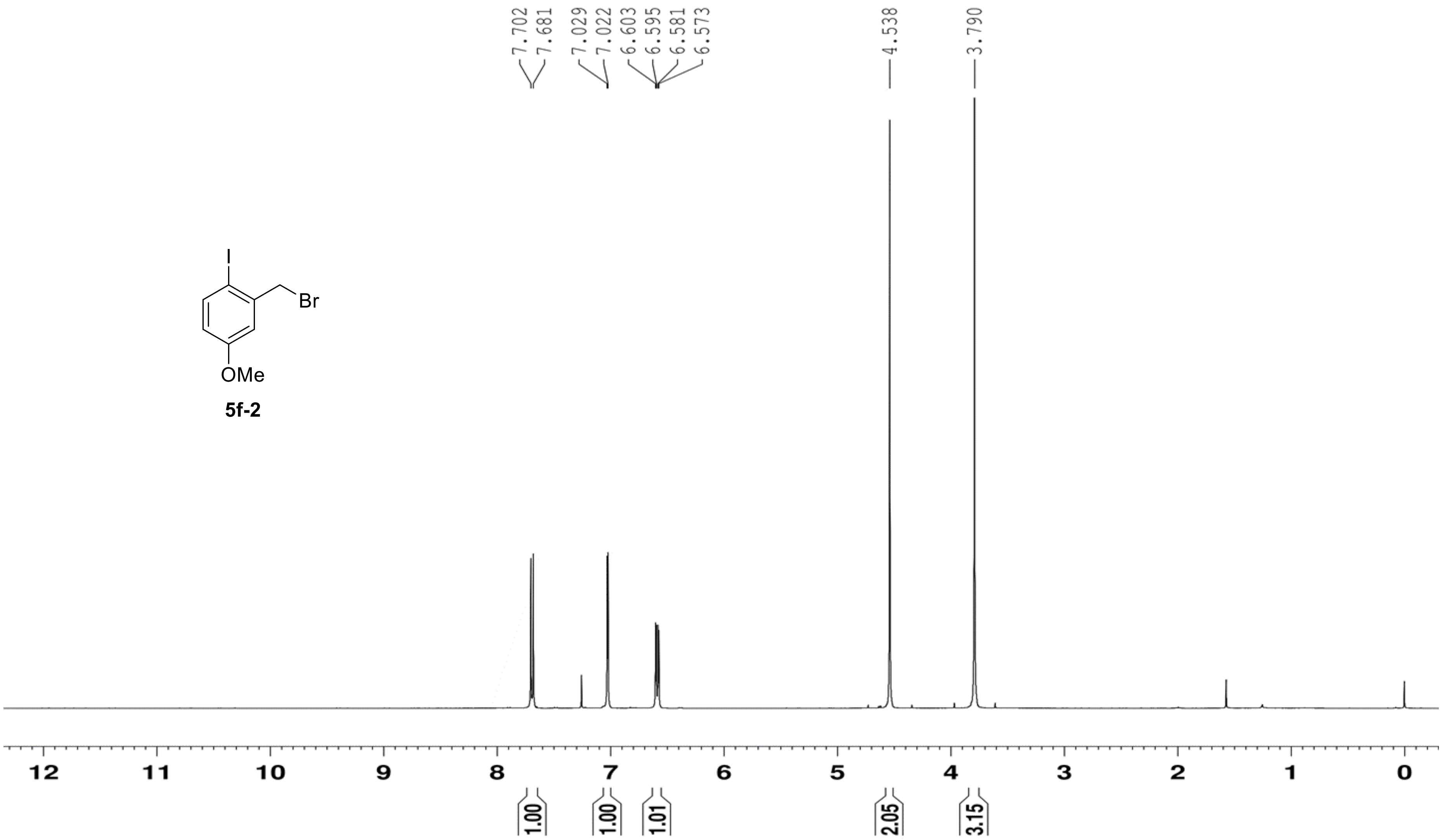

Supplementary Figure 29. ^{1}H NMR spectrum of **5f-2**.

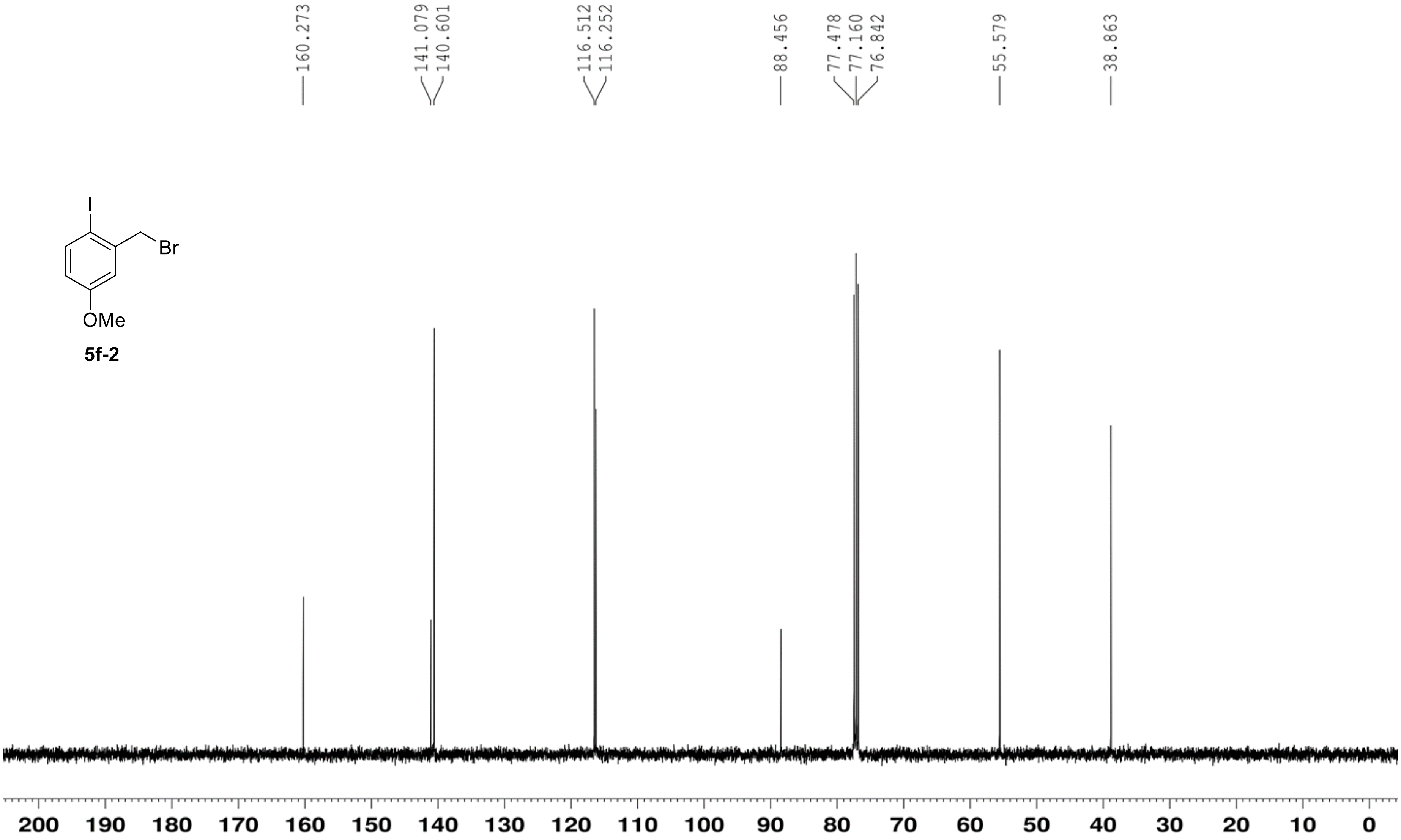

Supplementary Figure 30. ^{13}C NMR spectrum of **5f-2**.

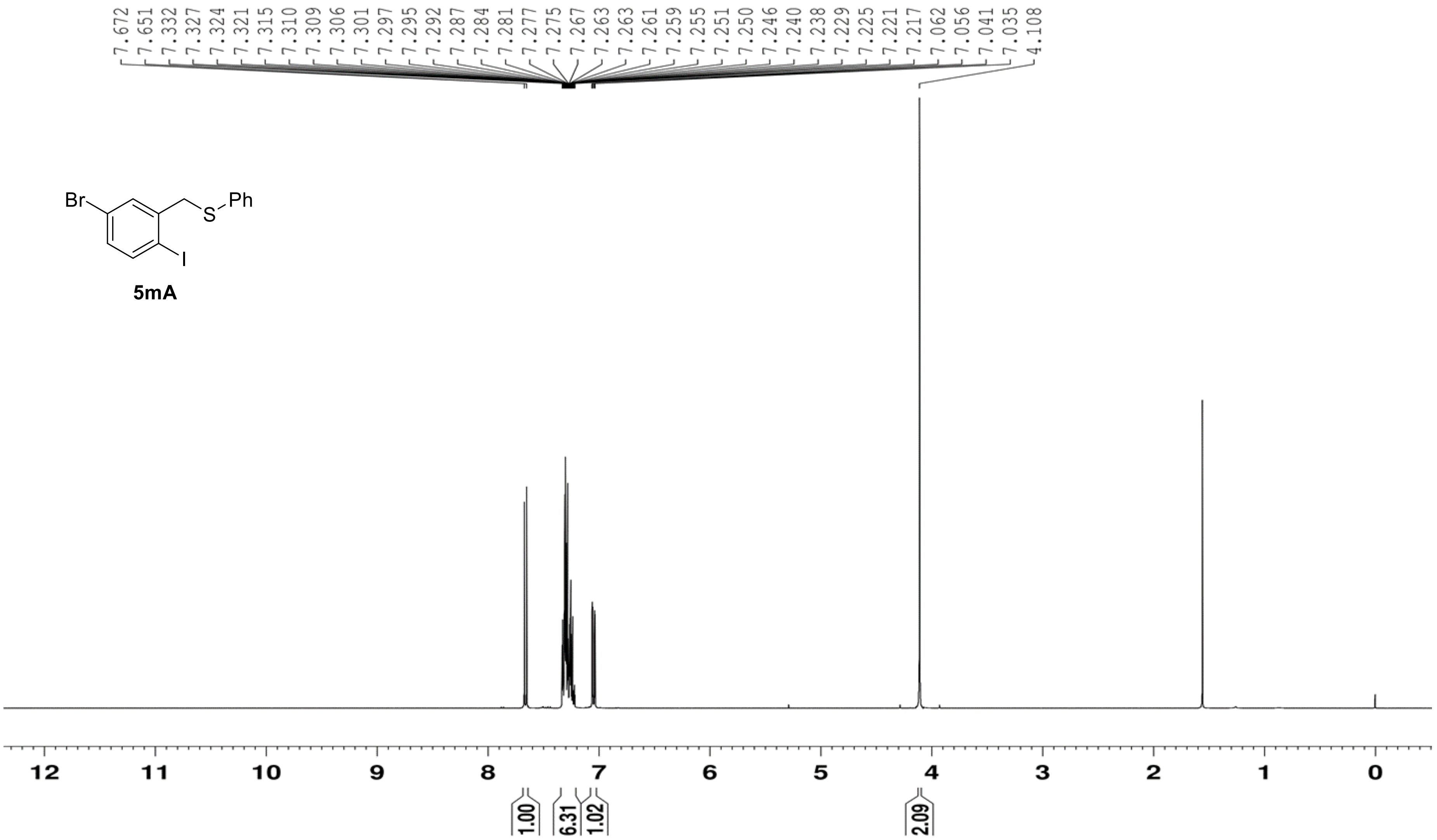

Supplementary Figure 31. ^{1}H NMR spectrum of **5mA**.

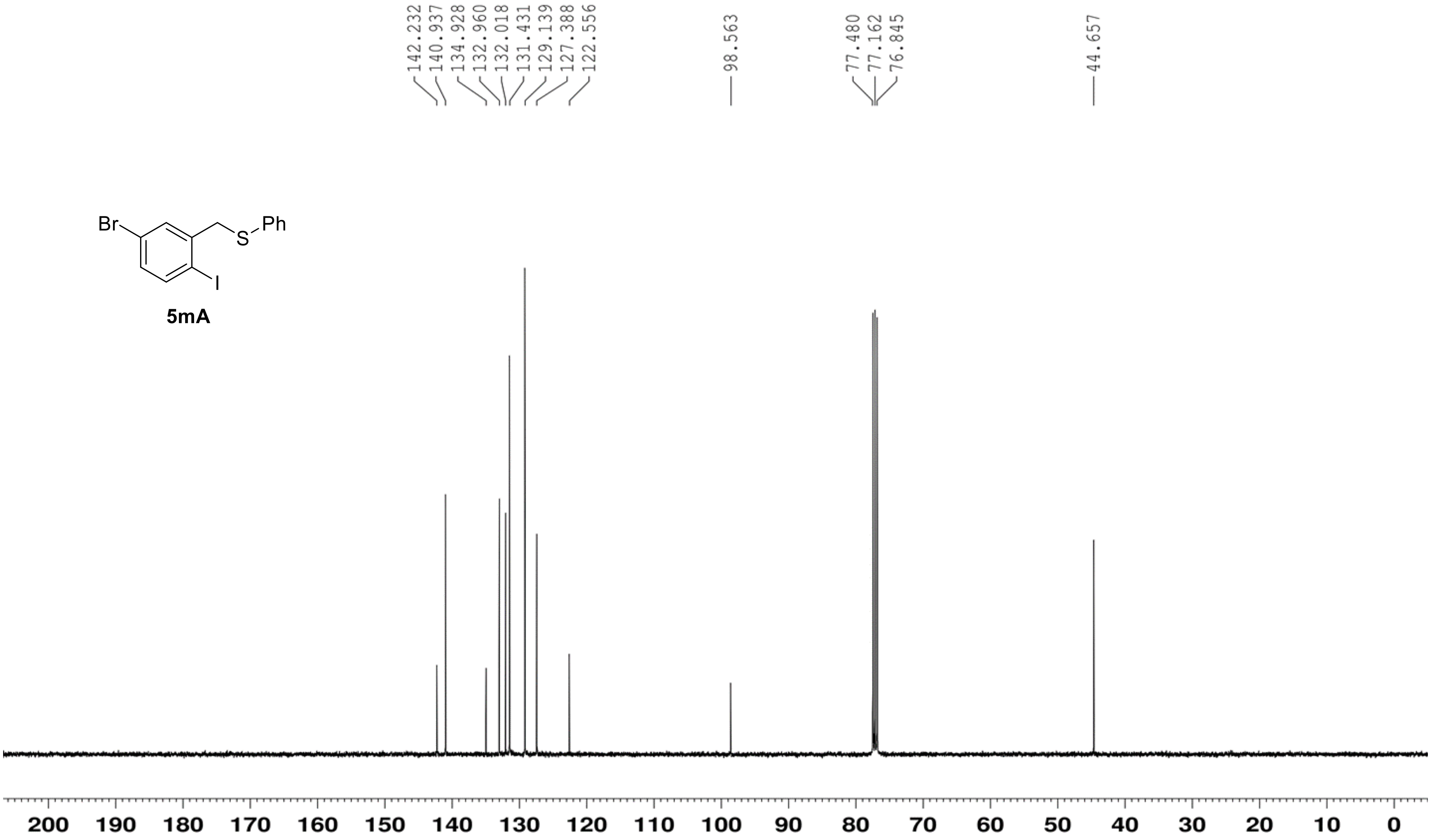

Supplementary Figure 32. ^{13}C NMR spectrum of **5mA**.

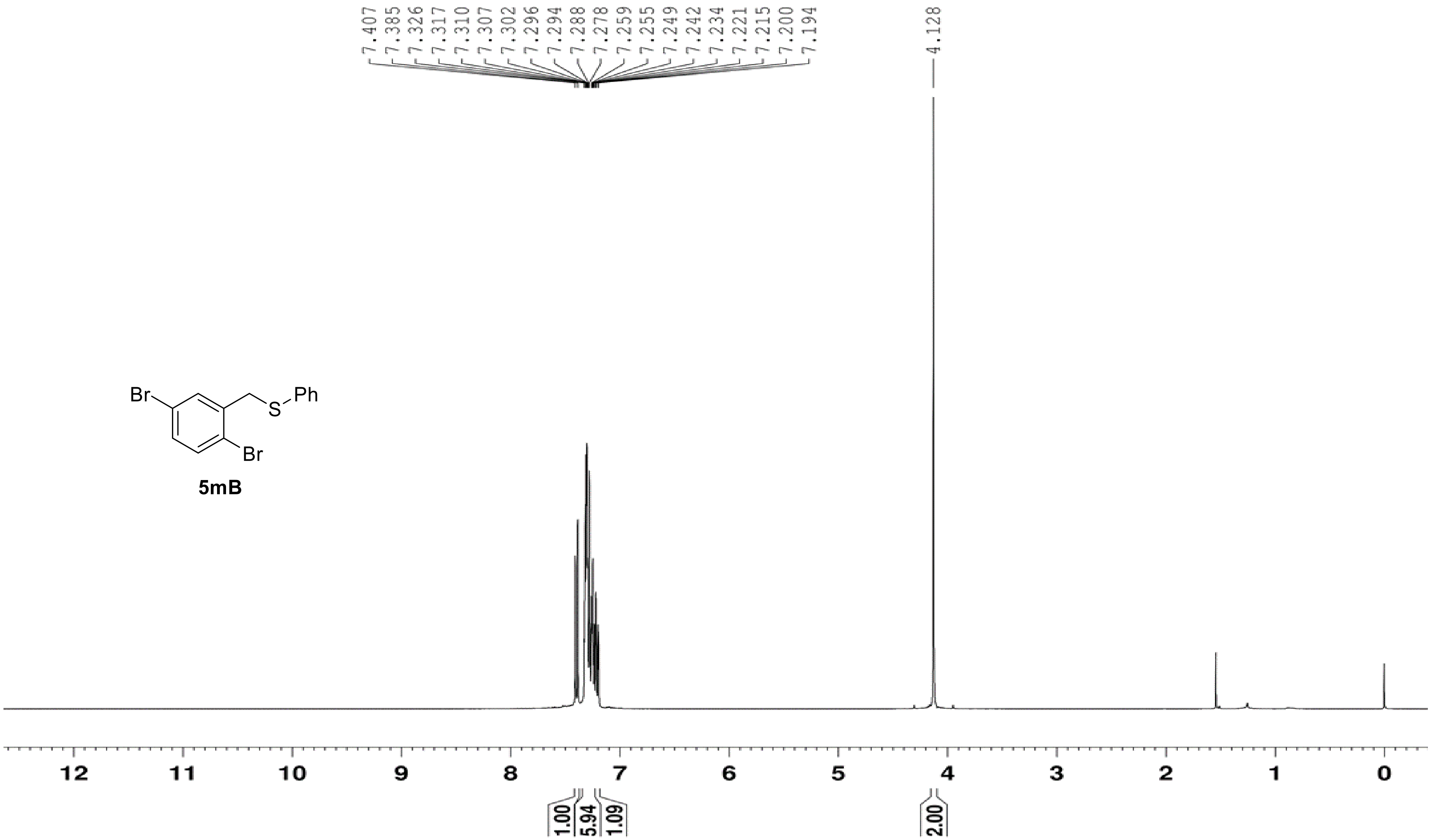

Supplementary Figure 33. ^{1}H NMR spectrum of **5mB**.

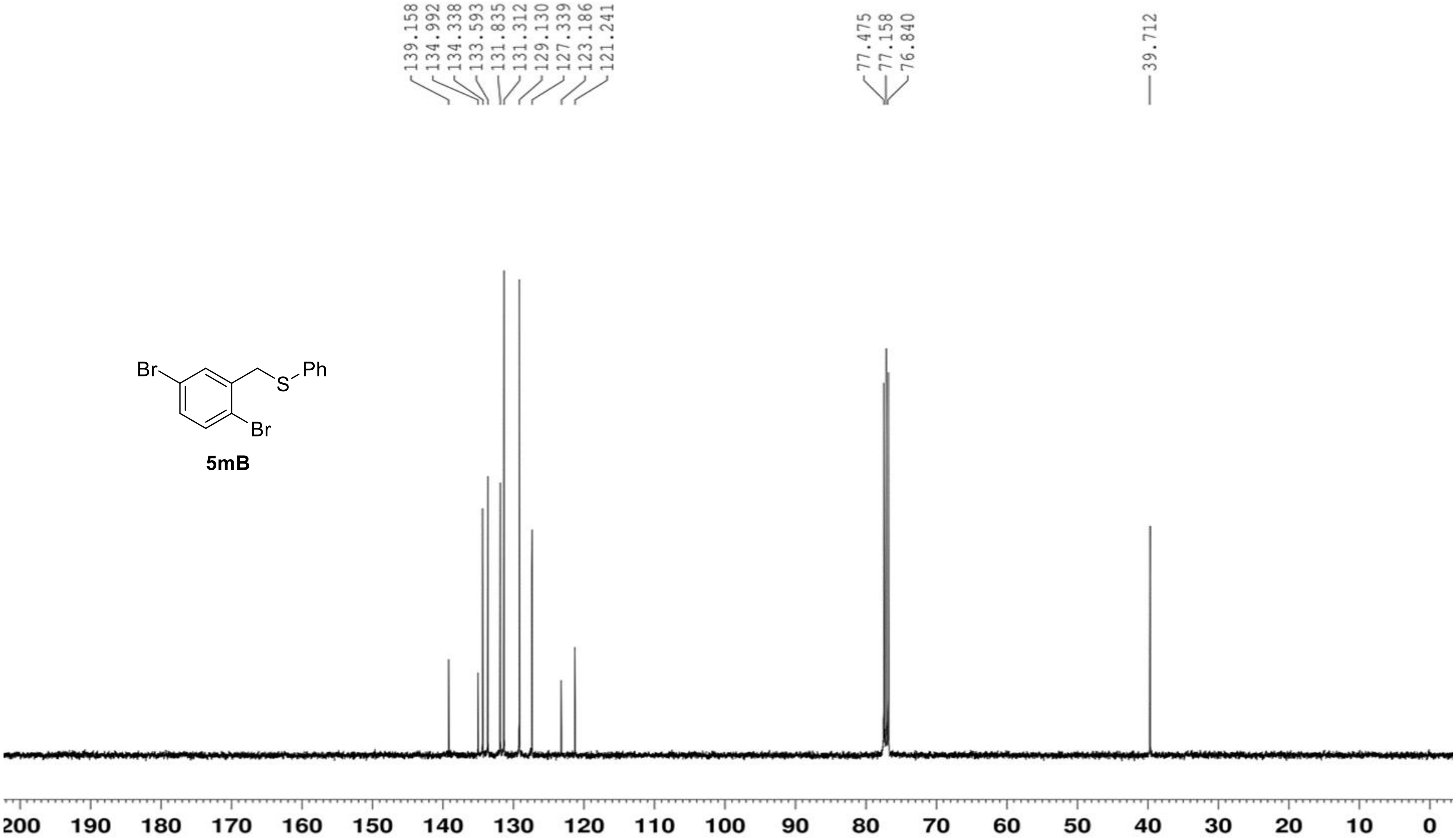

Supplementary Figure 34. ^{13}C NMR spectrum of **5mB**.

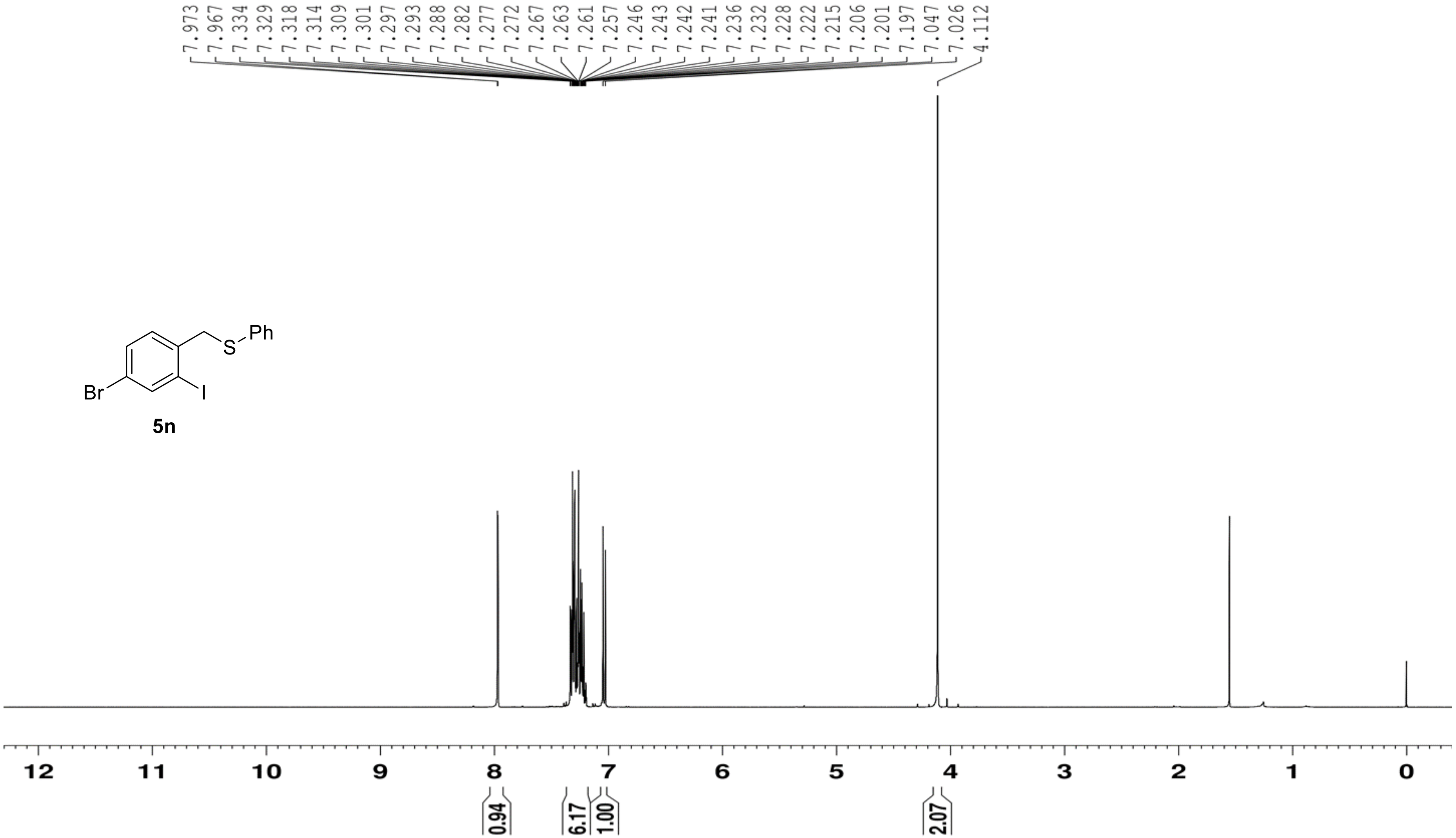

Supplementary Figure 35. ^{1}H NMR spectrum of **5n**.

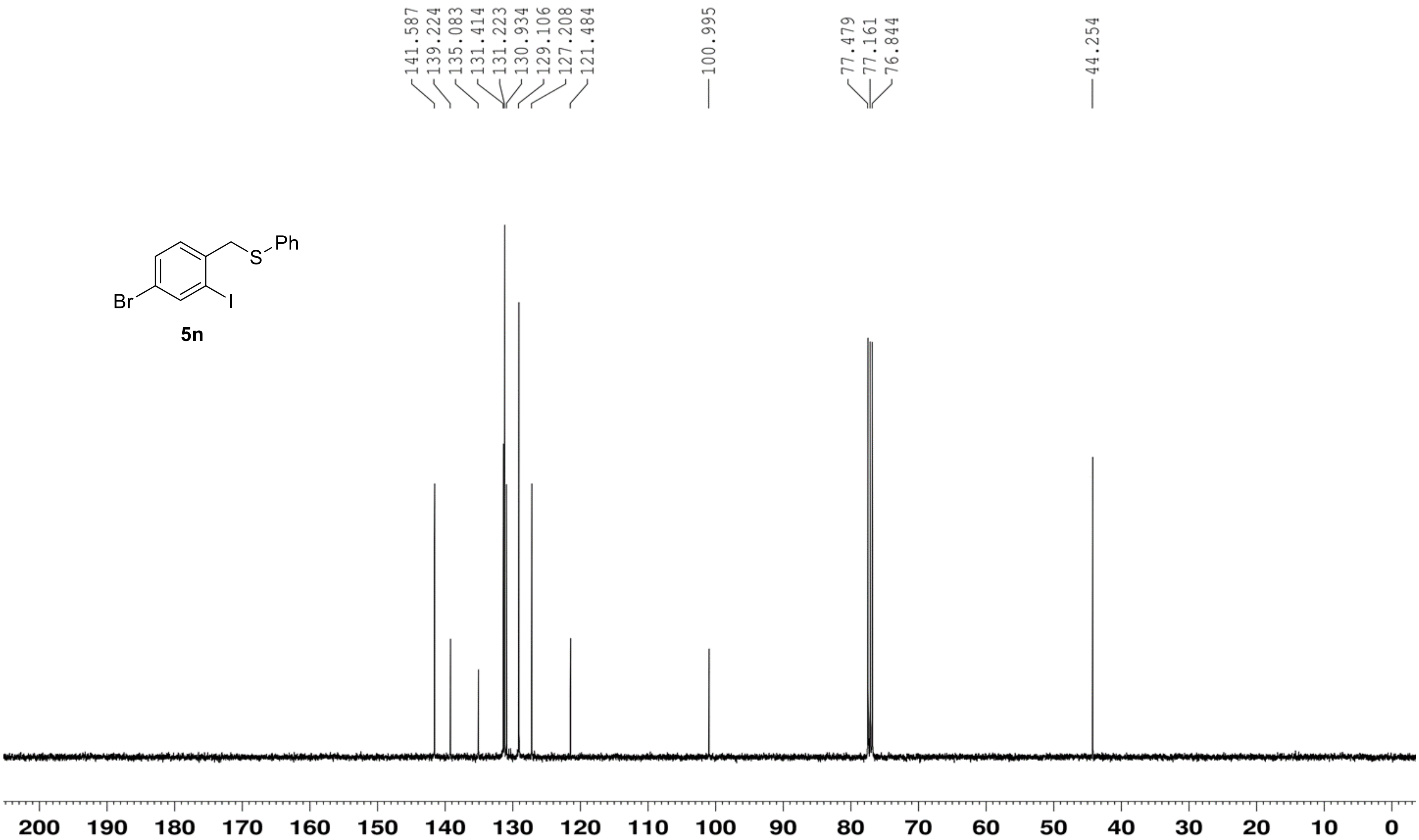

Supplementary Figure 36. ^{13}C NMR spectrum of **5n**.

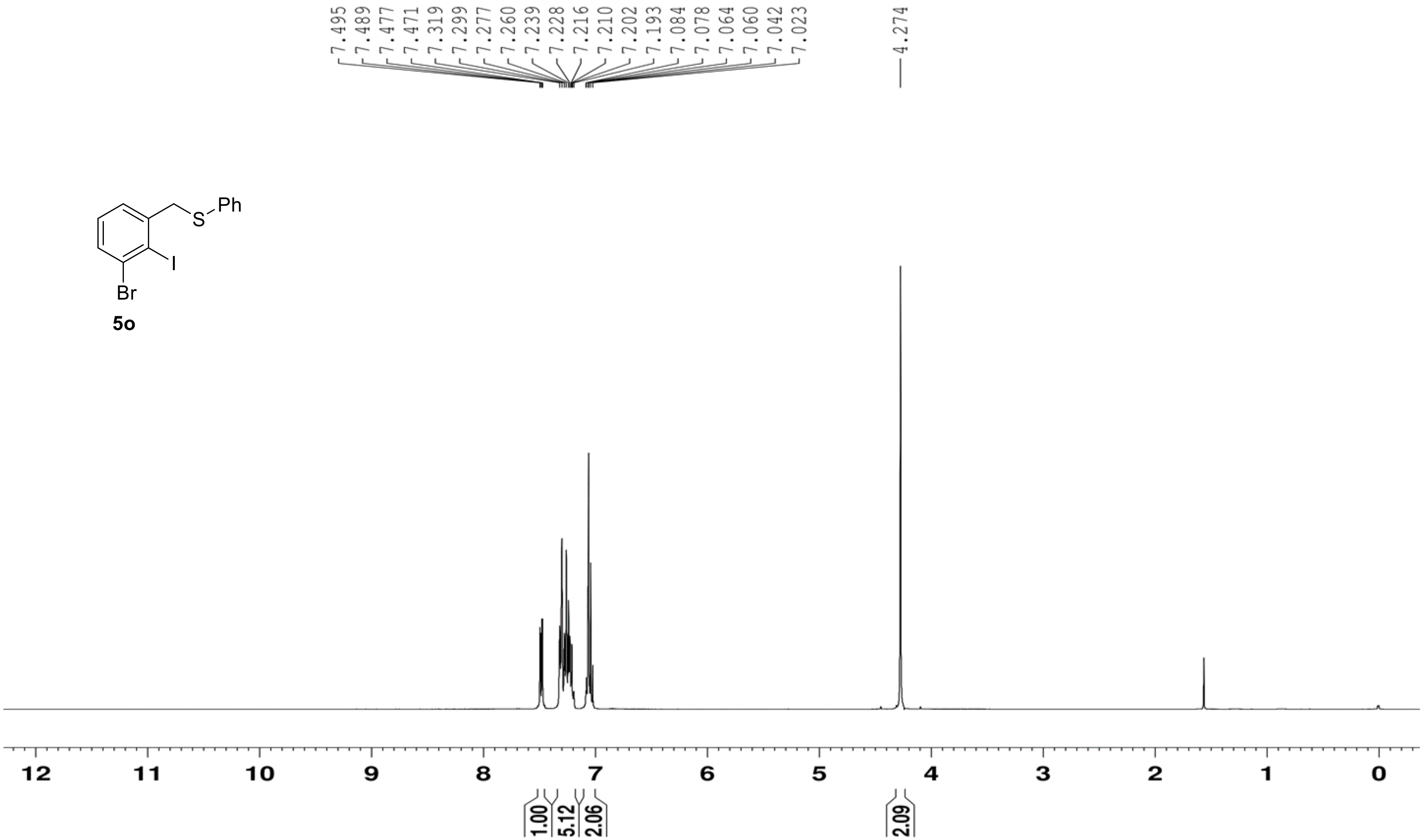

Supplementary Figure 37. ^{1}H NMR spectrum of **5o**.

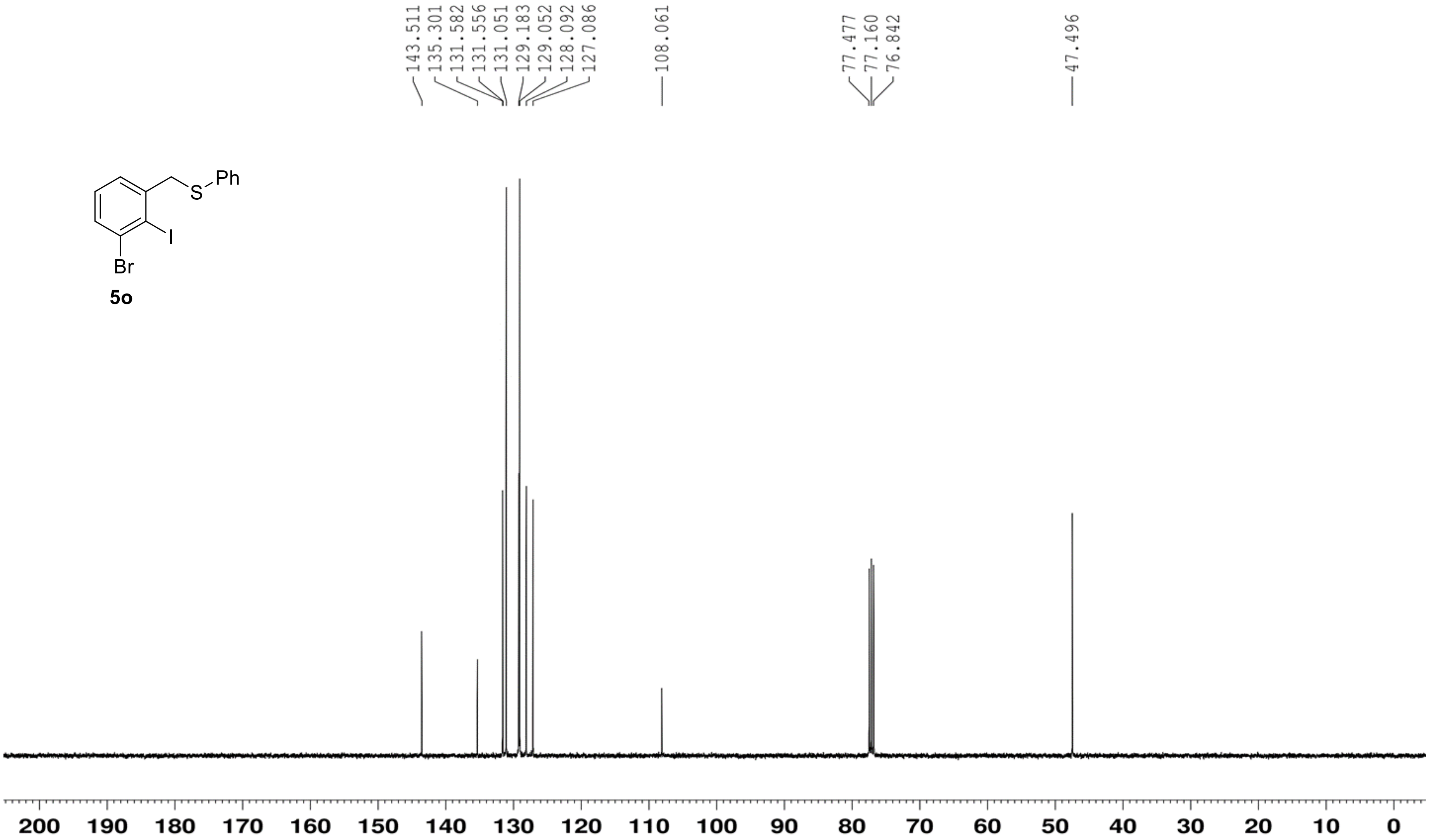

Supplementary Figure 38. ^{13}C NMR spectrum of **5o**.

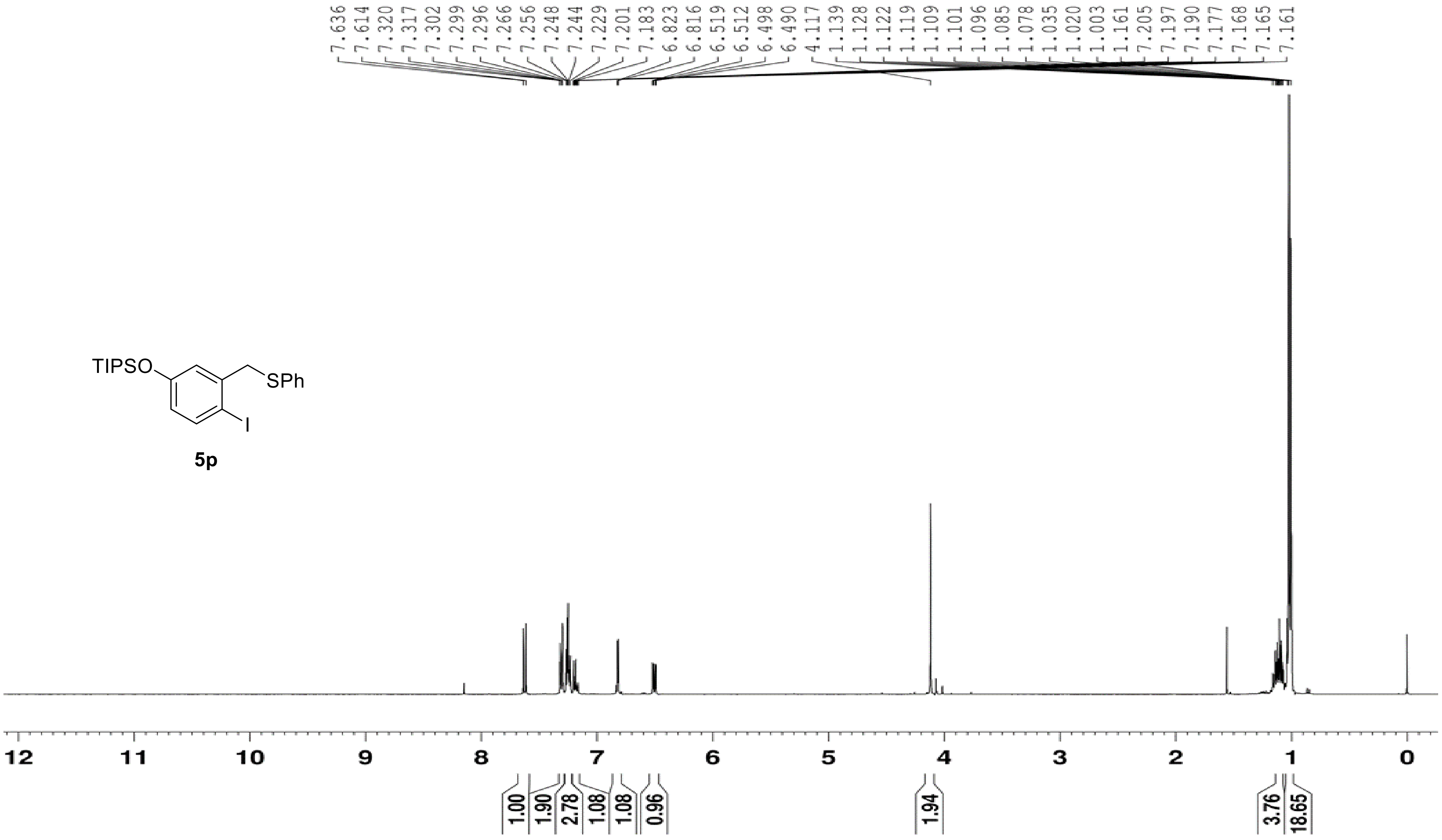

Supplementary Figure 39. ^{1}H NMR spectrum of **5p**.

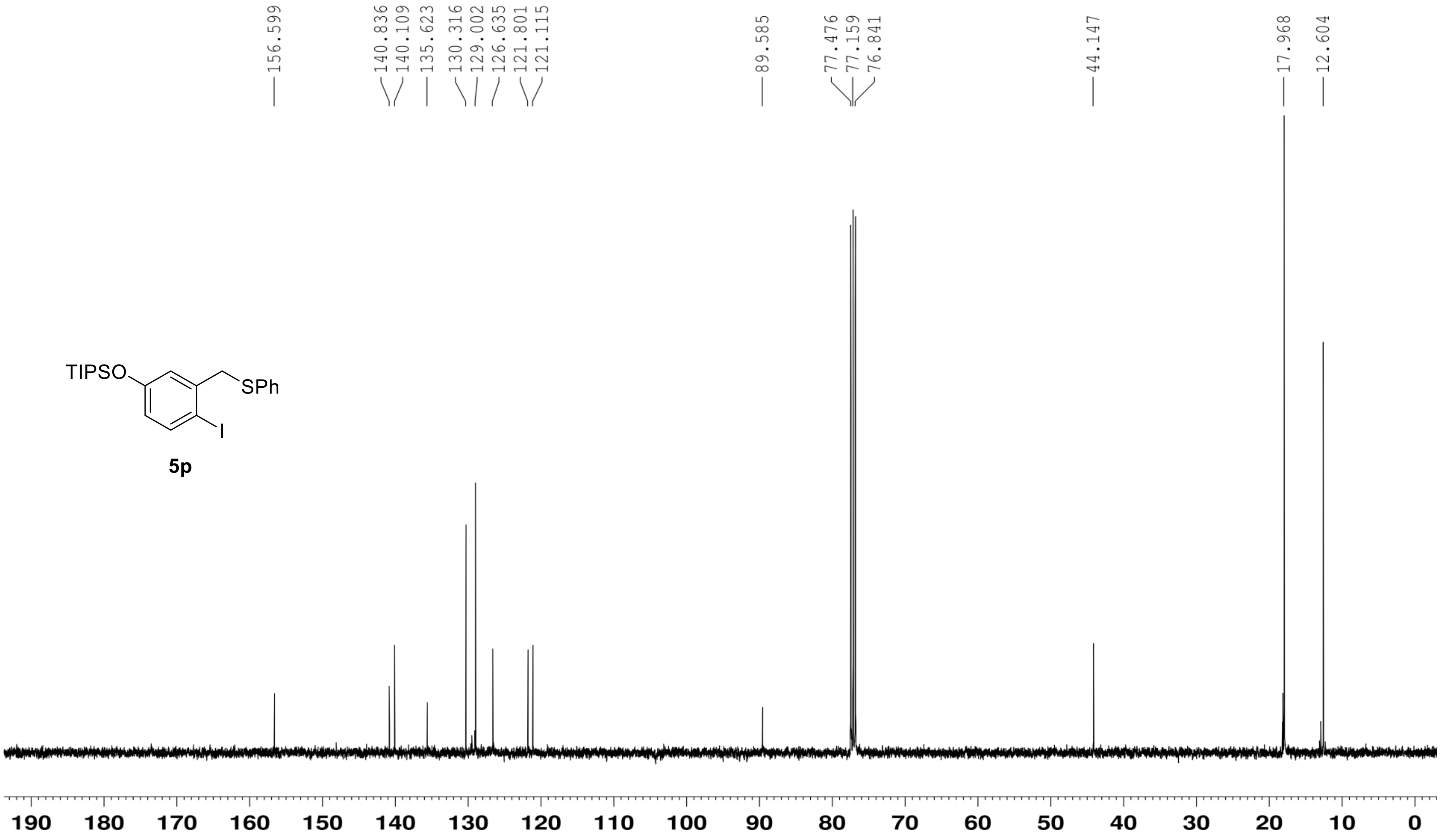

Supplementary Figure 40. ^{13}C NMR spectrum of **5p**.

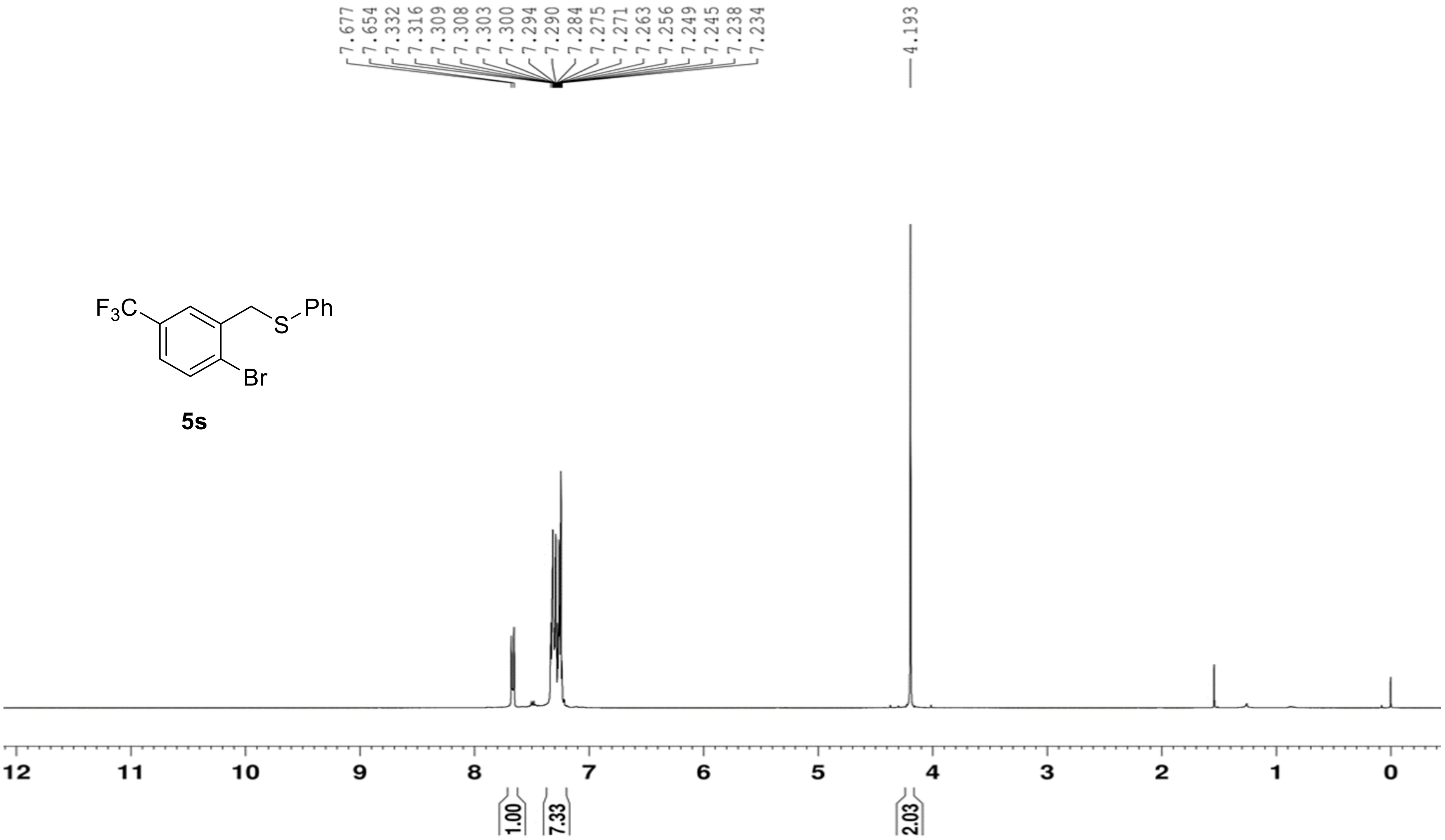

Supplementary Figure 41. ^{1}H NMR spectrum of **5s**.

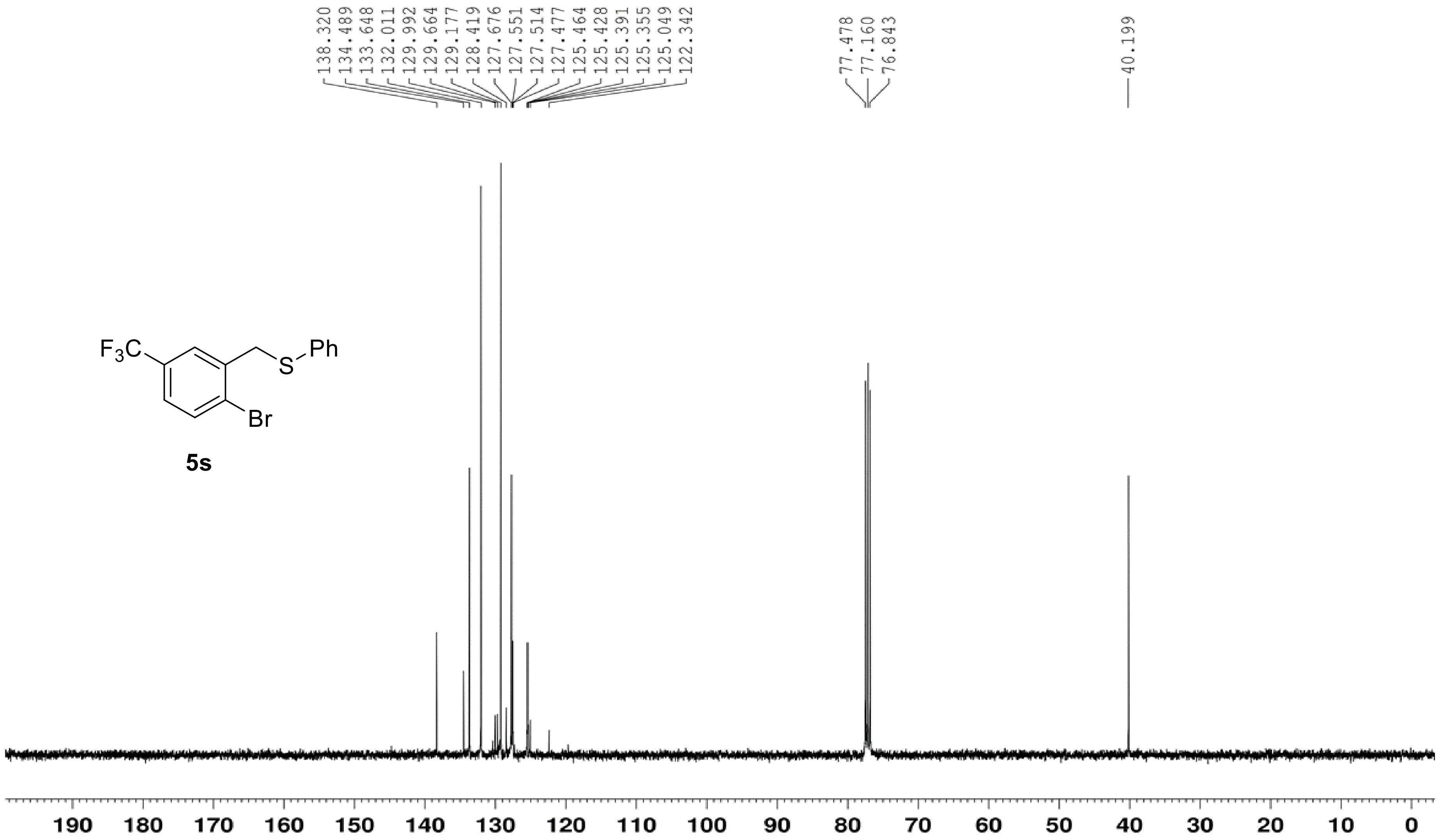

Supplementary Figure 42. ^{13}C NMR spectrum of **5s**.

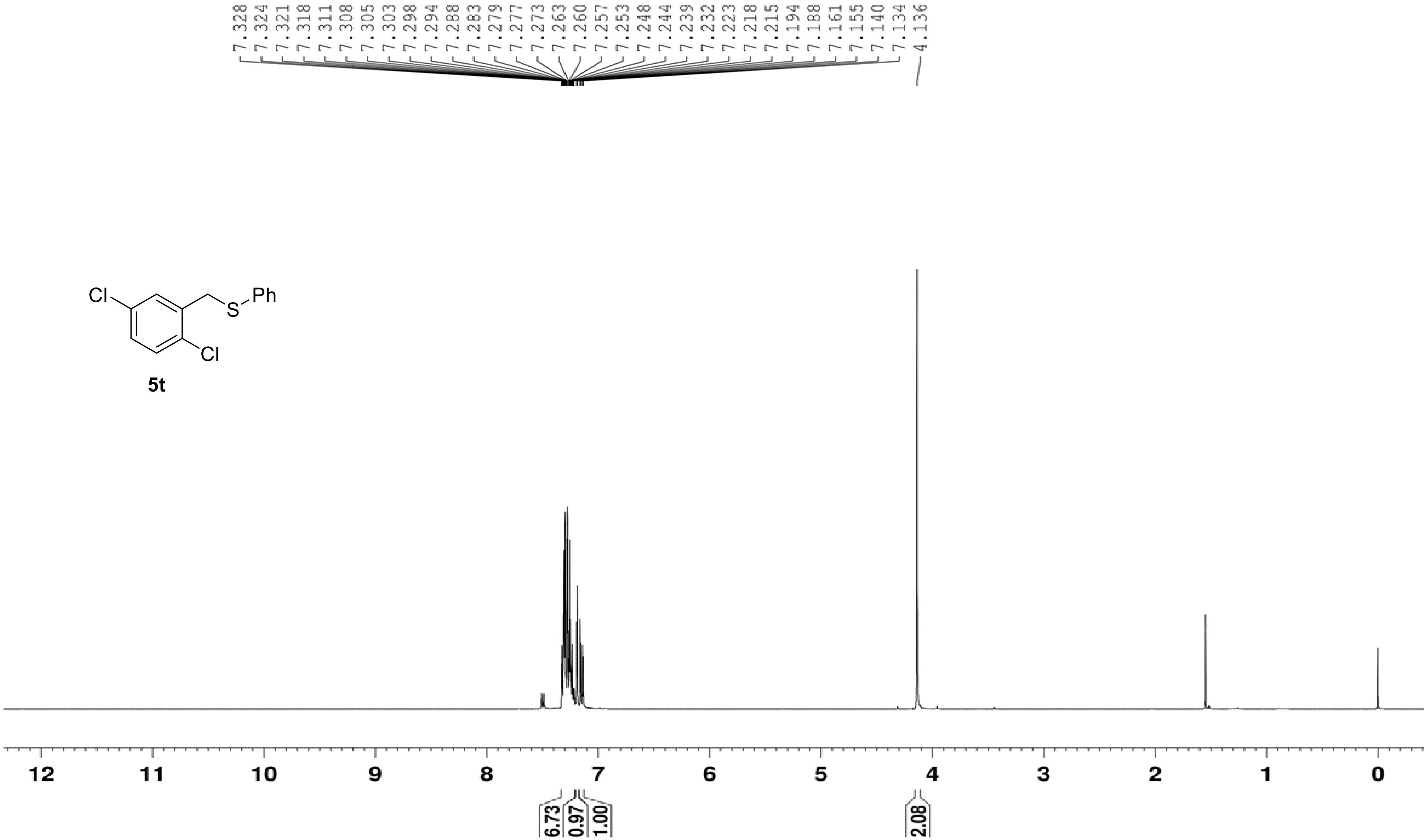

Supplementary Figure 43. ^{1}H NMR spectrum of **5t**.

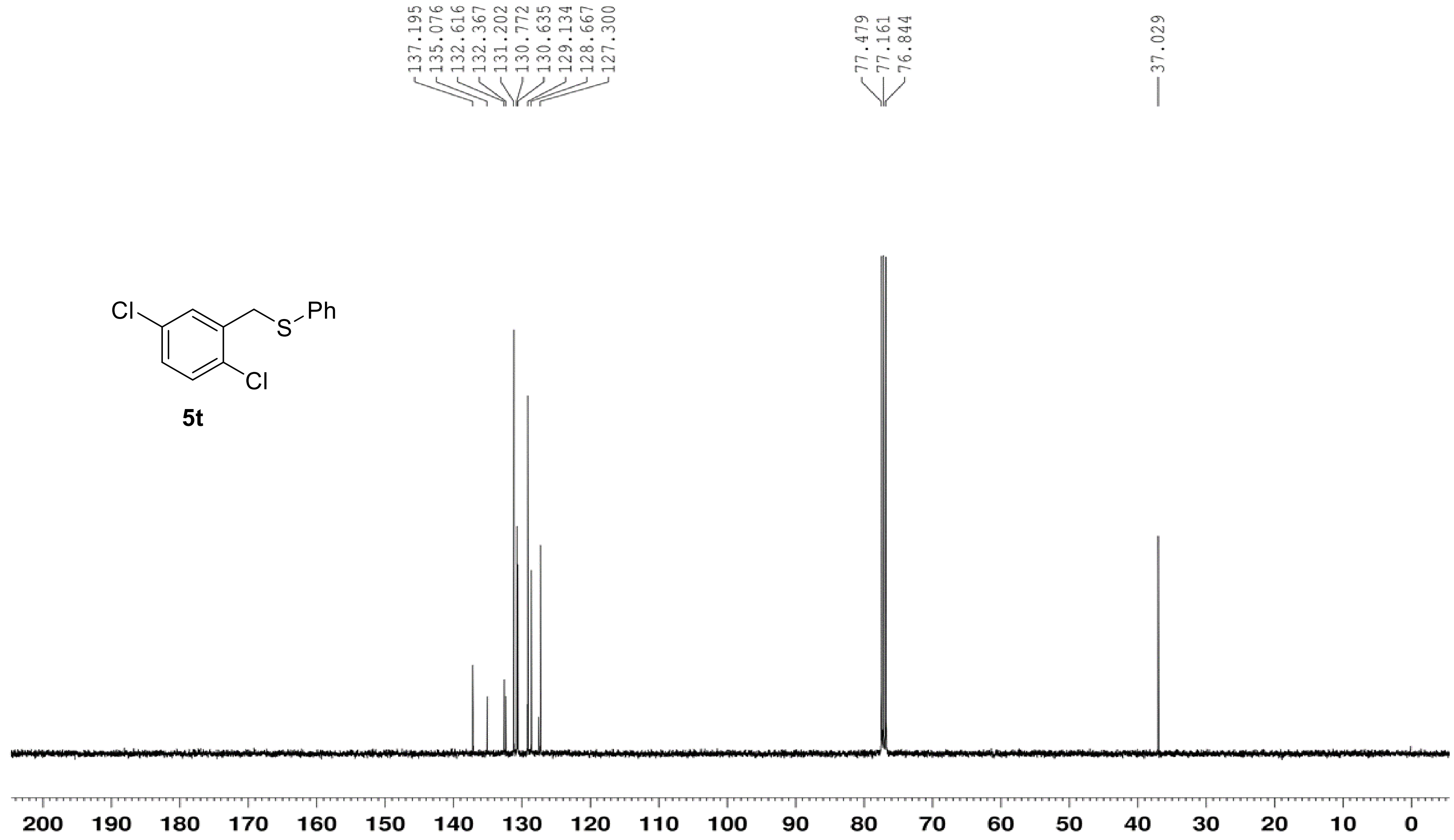

Supplementary Figure 44. ^{13}C NMR spectrum of **5t**.

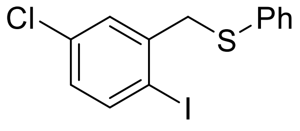

5y

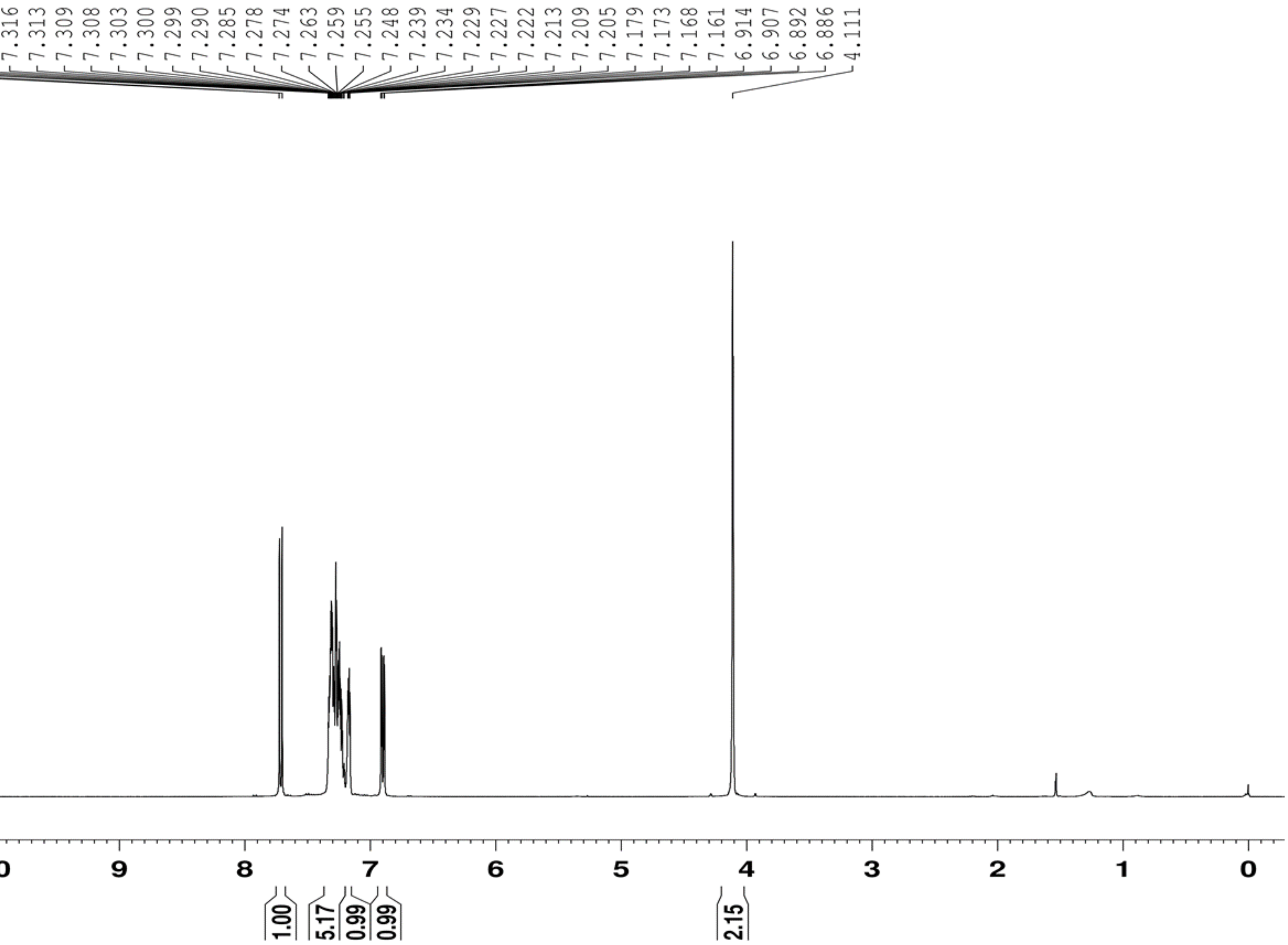

Supplementary Figure 45. ^{1}H NMR spectrum of **5y**.

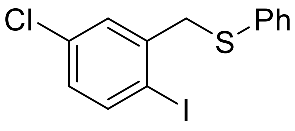

5y

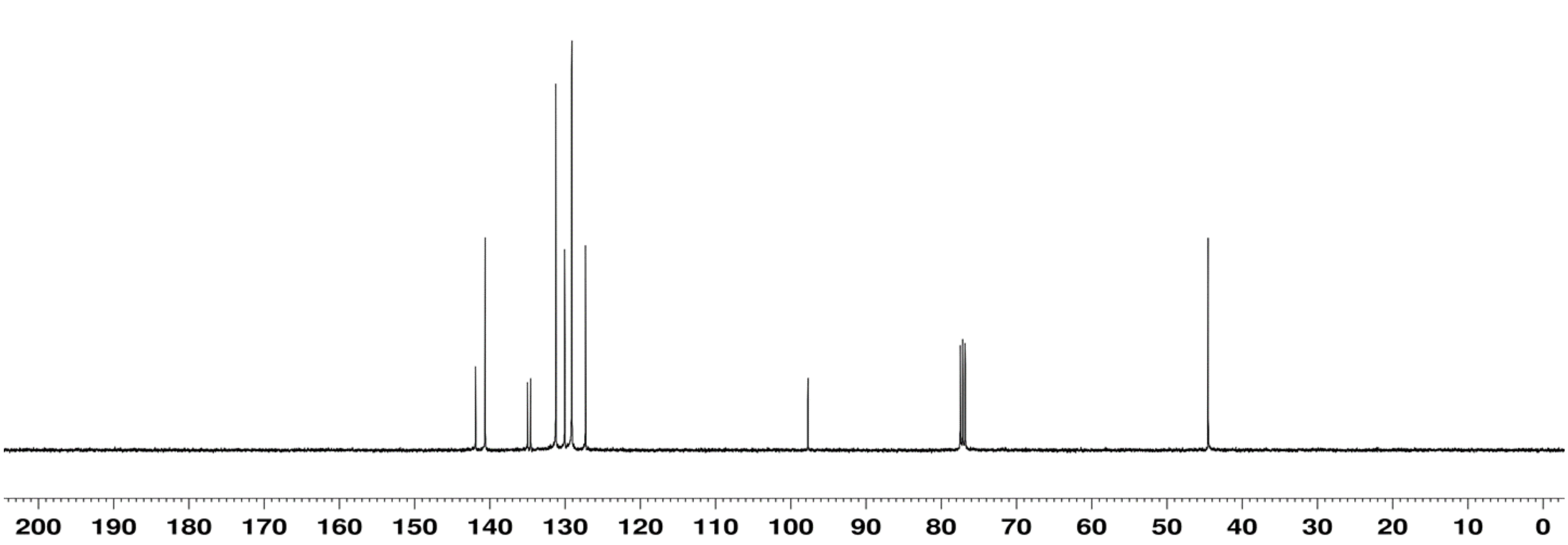

Supplementary Figure 46. ^{13}C NMR spectrum of **5y**.

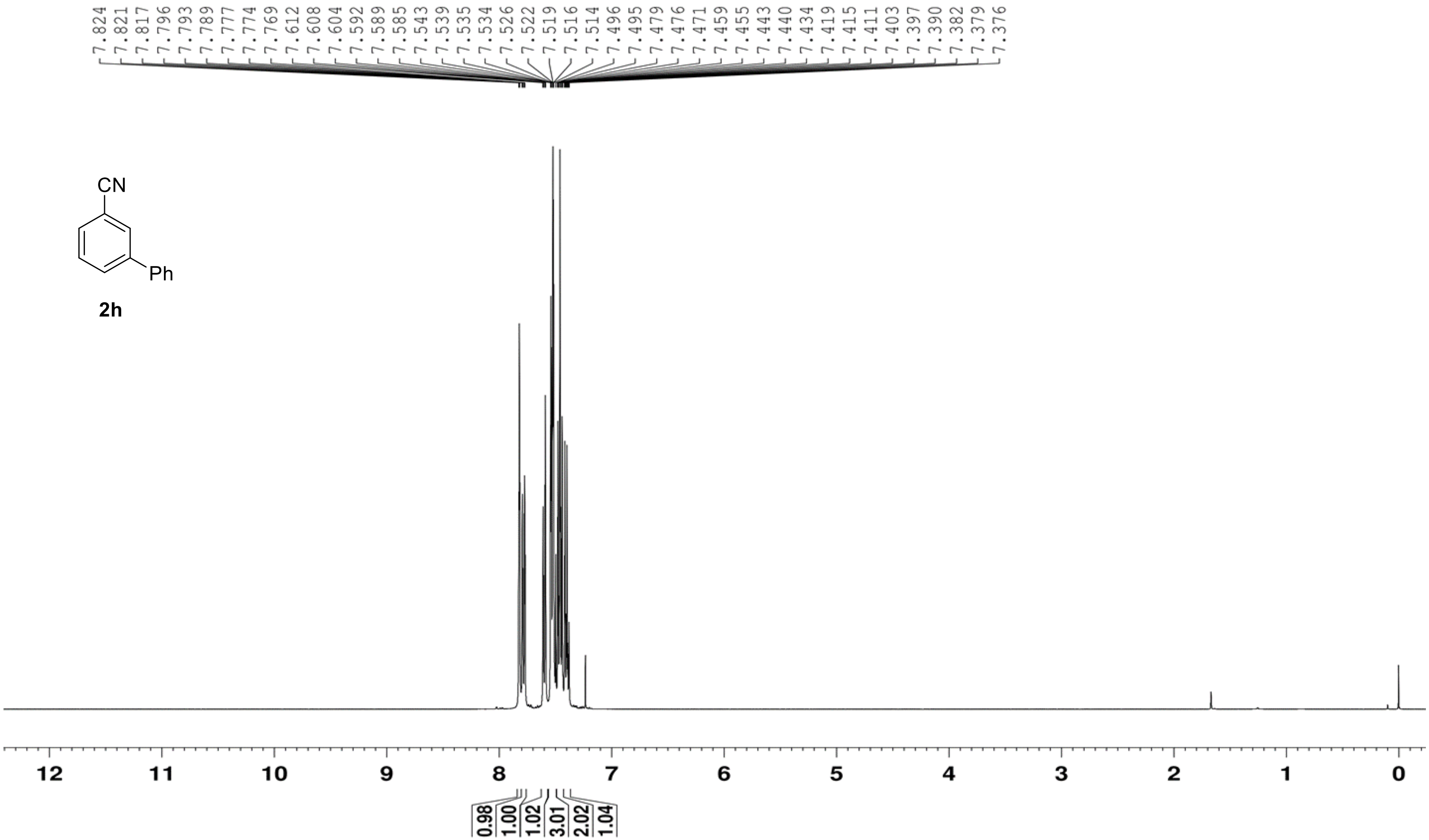

Supplementary Figure 47. ^{1}H NMR spectrum of **2h**.

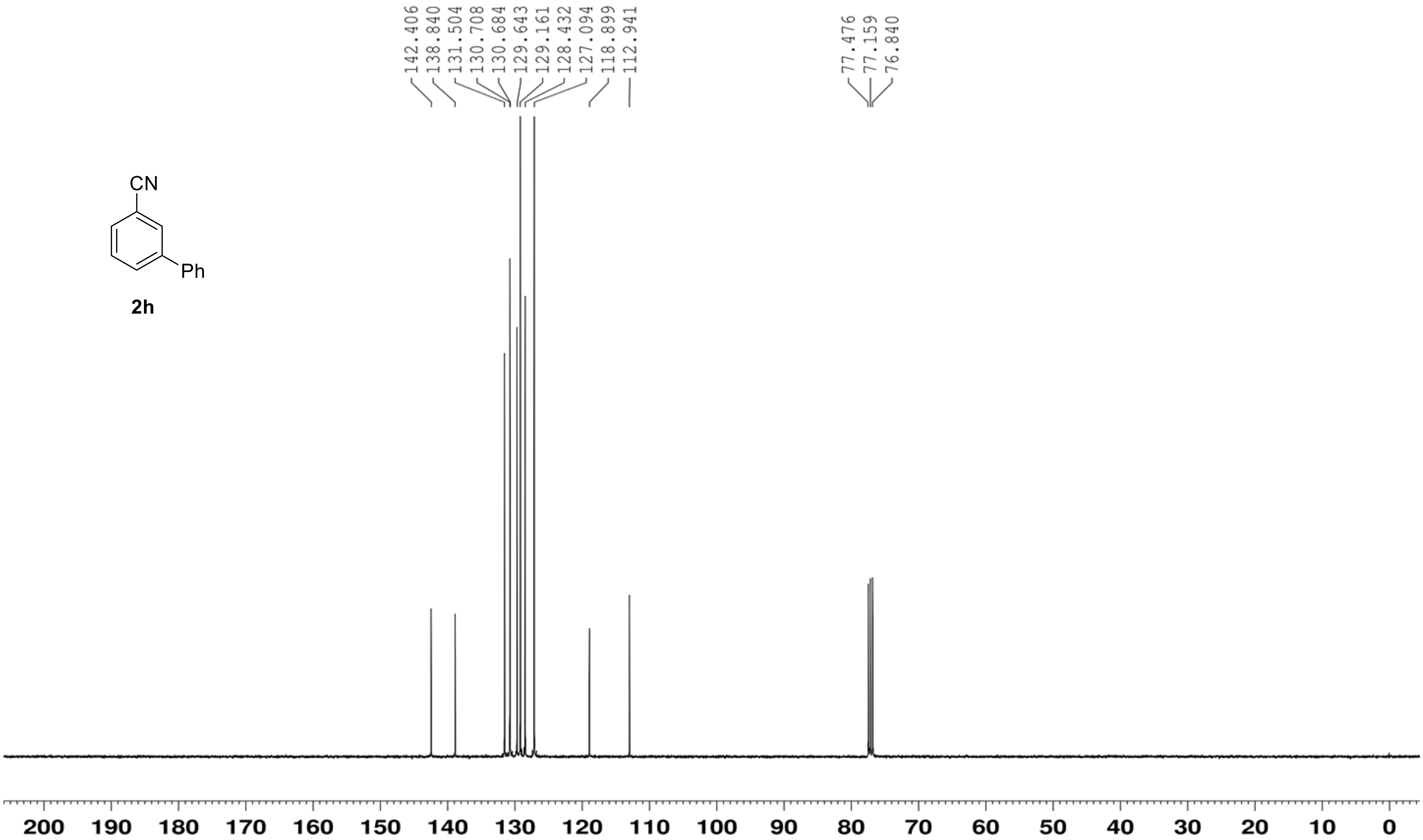

Supplementary Figure 48. ^{13}C NMR spectrum of **2h**.

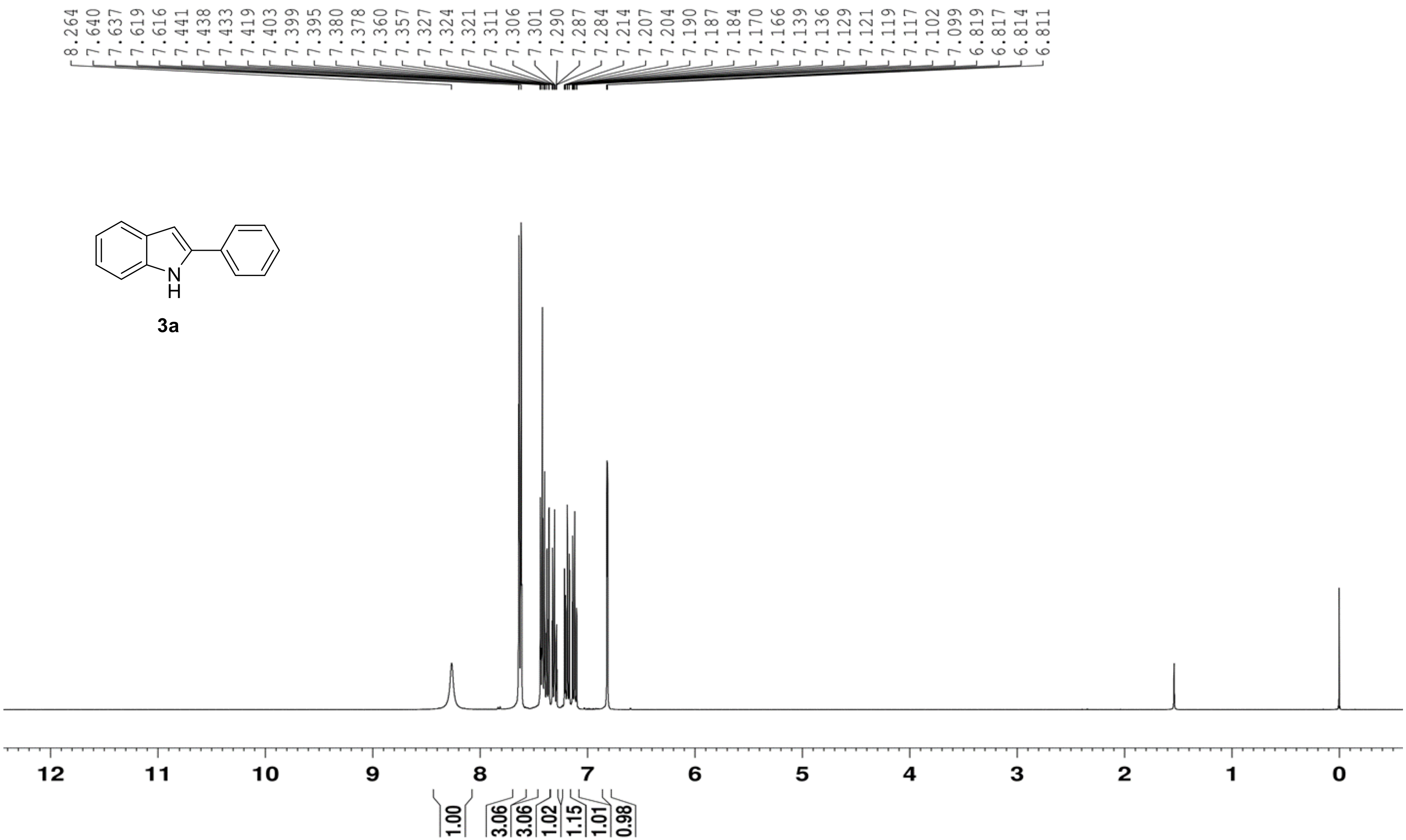

Supplementary Figure 49. ^{1}H NMR spectrum of **3a**.

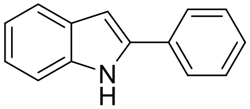

3a

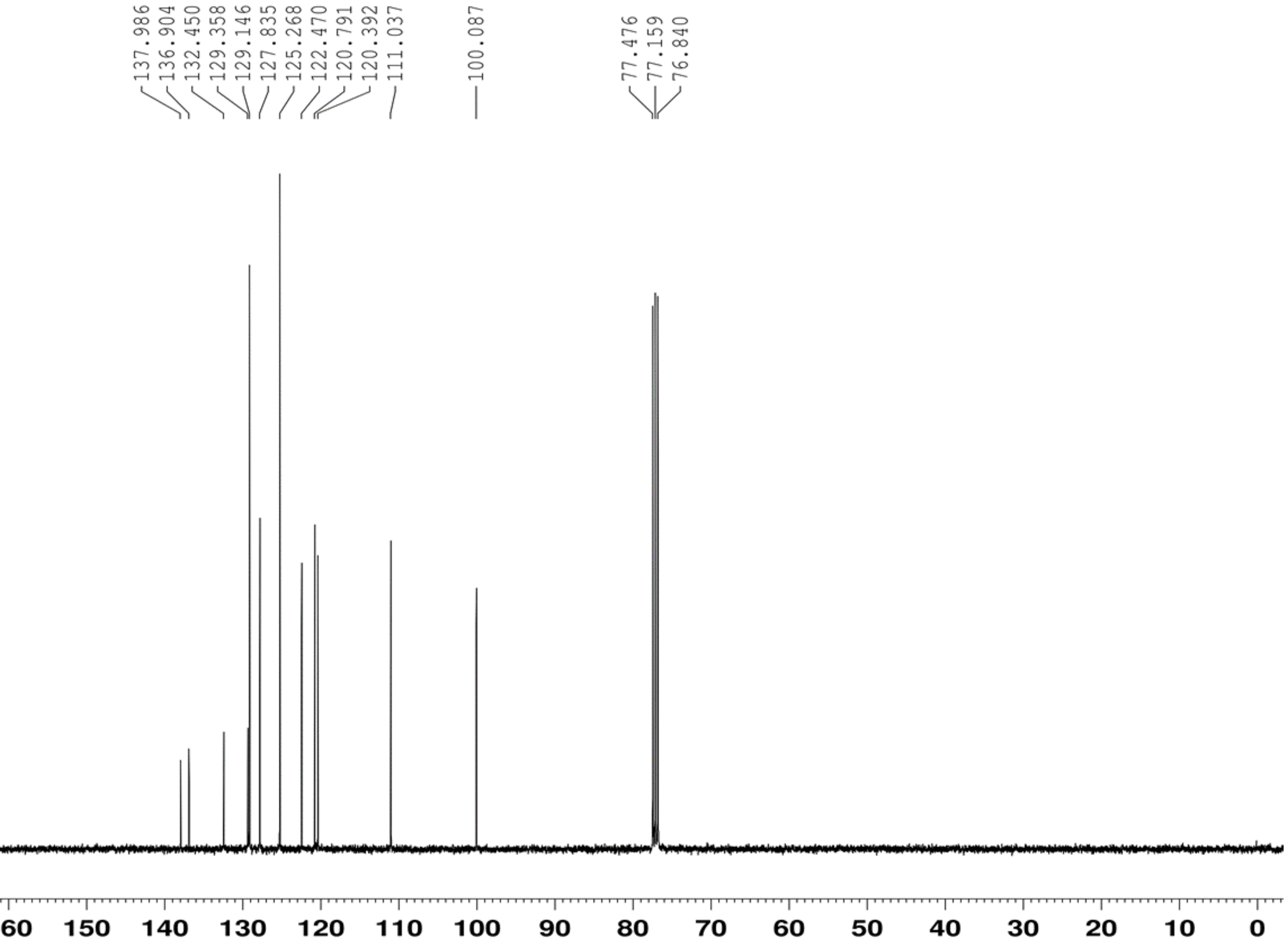

Supplementary Figure 50. ^{13}C NMR spectrum of **3a**.

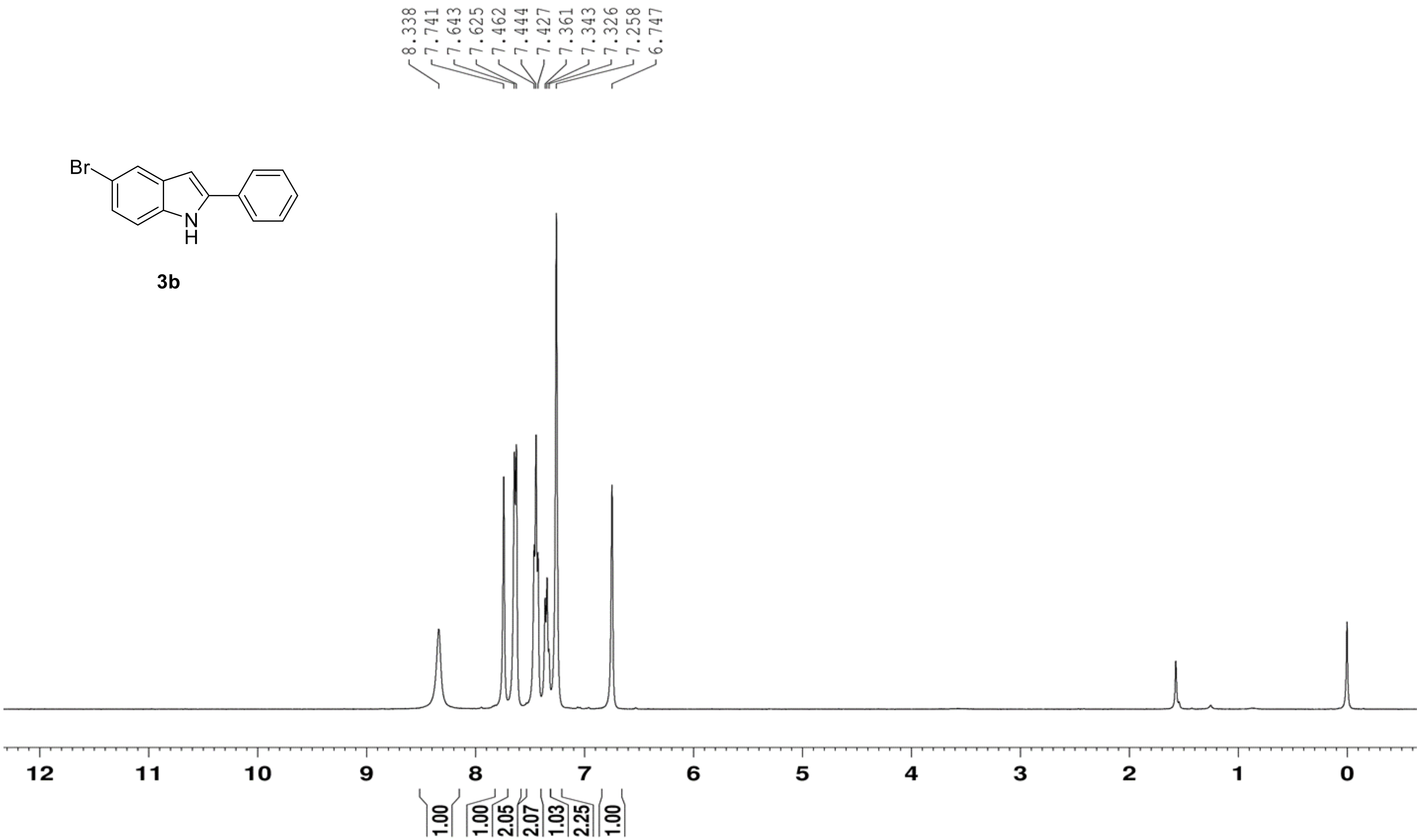

Supplementary Figure 51. ^{1}H NMR spectrum of **3b**.

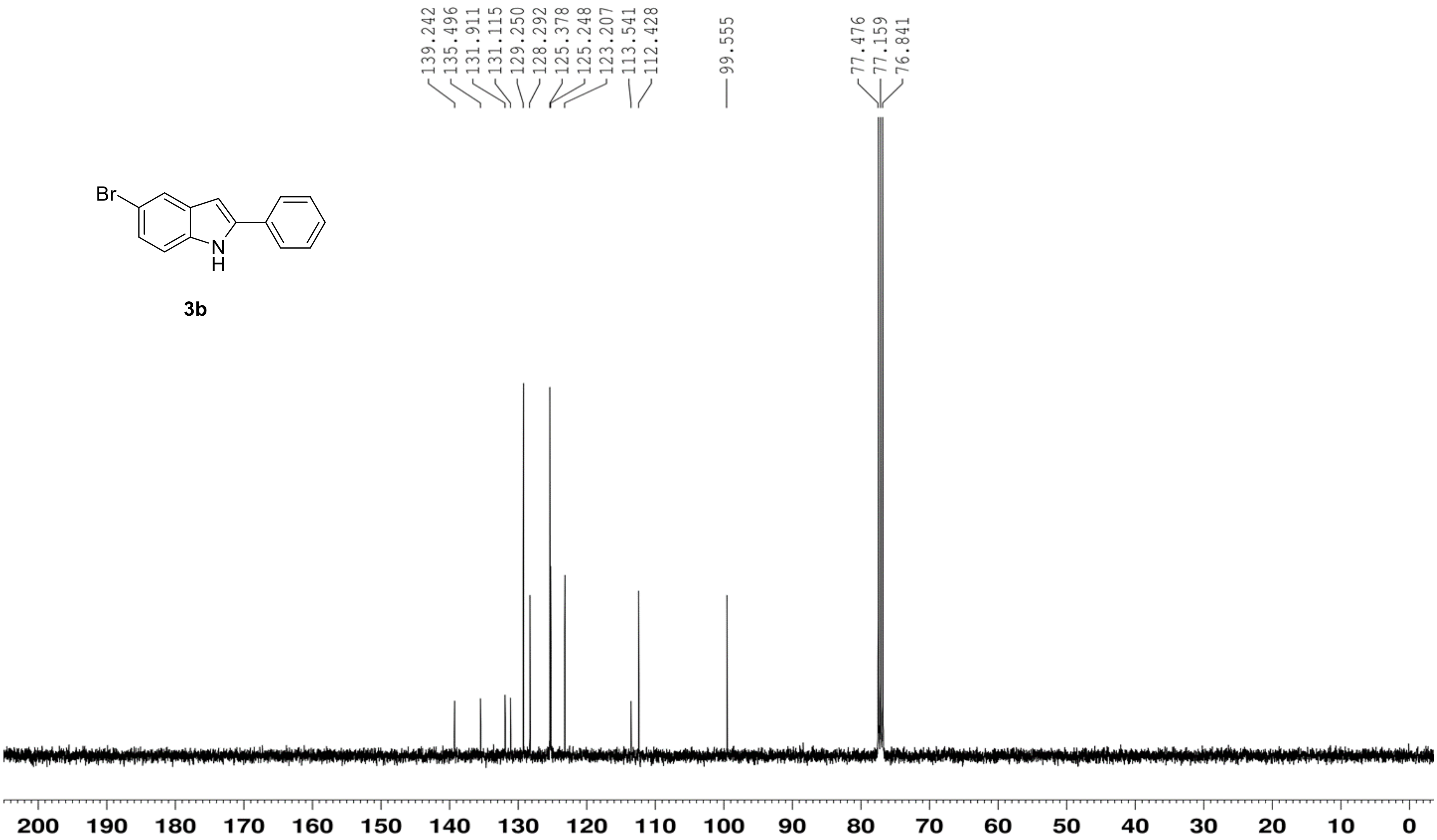

Supplementary Figure 52. ^{13}C NMR spectrum of **3b**.

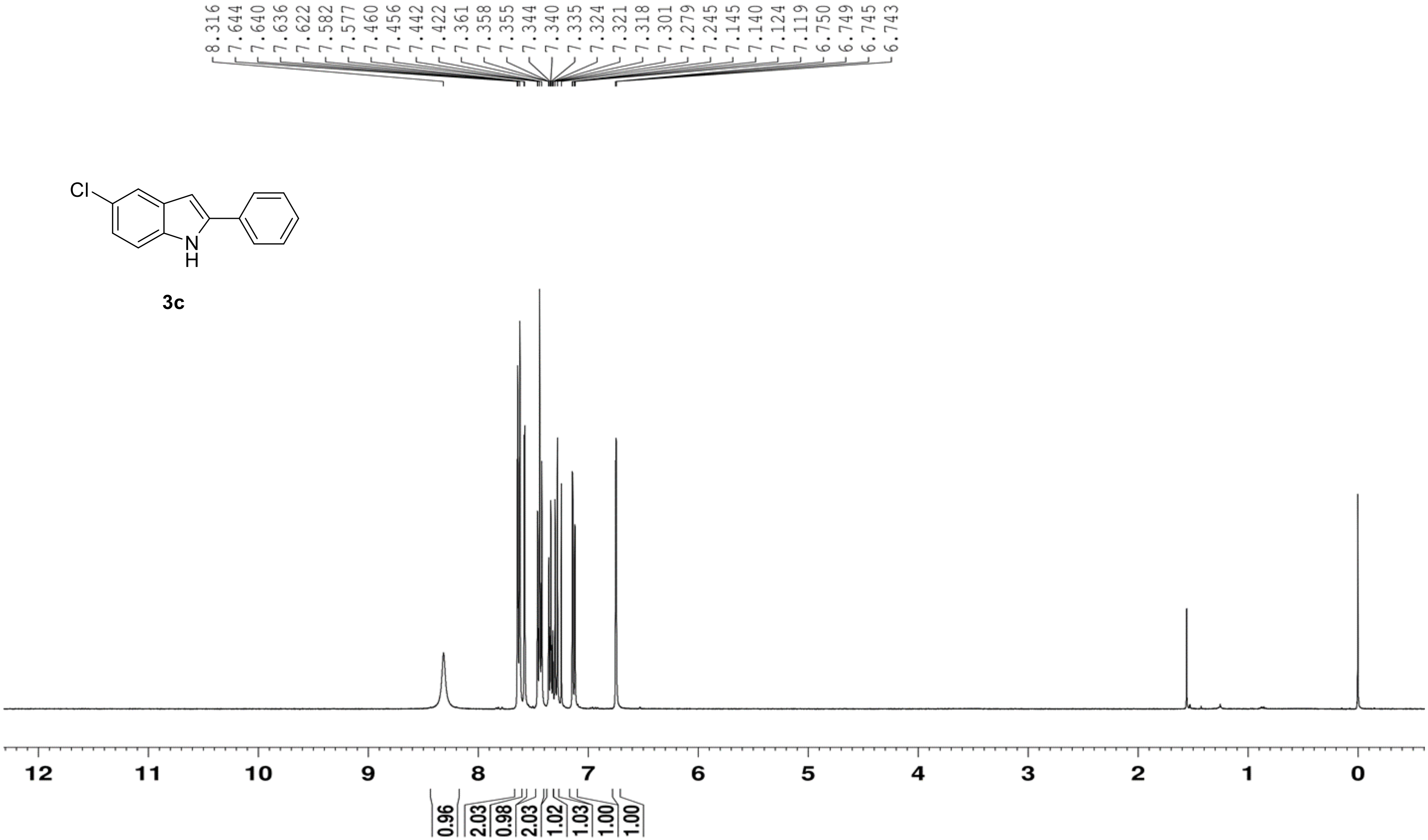

Supplementary Figure 53. ^{1}H NMR spectrum of **3c**.

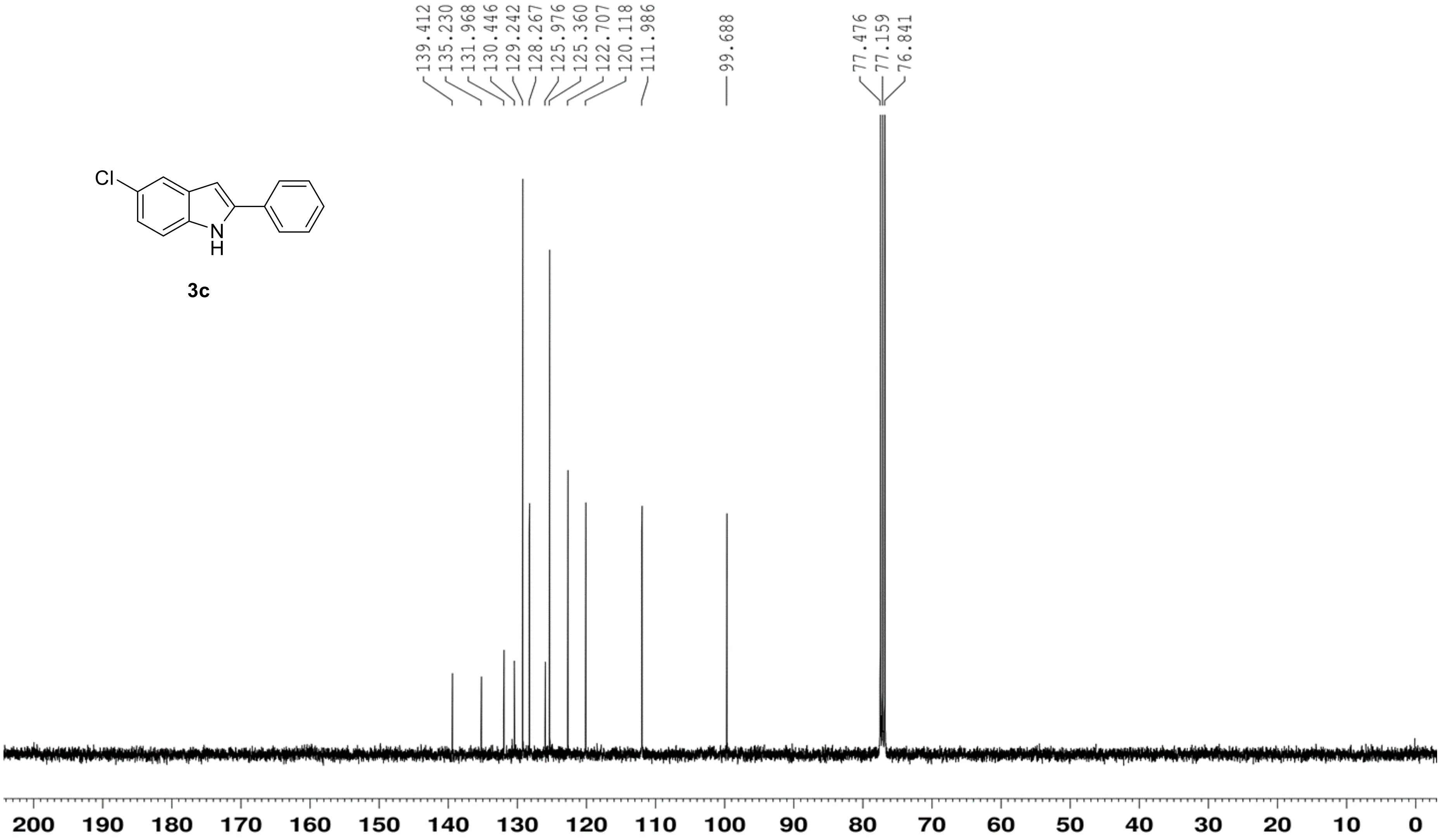

3c

Supplementary Figure 54. ^{13}C NMR spectrum of **3c**.

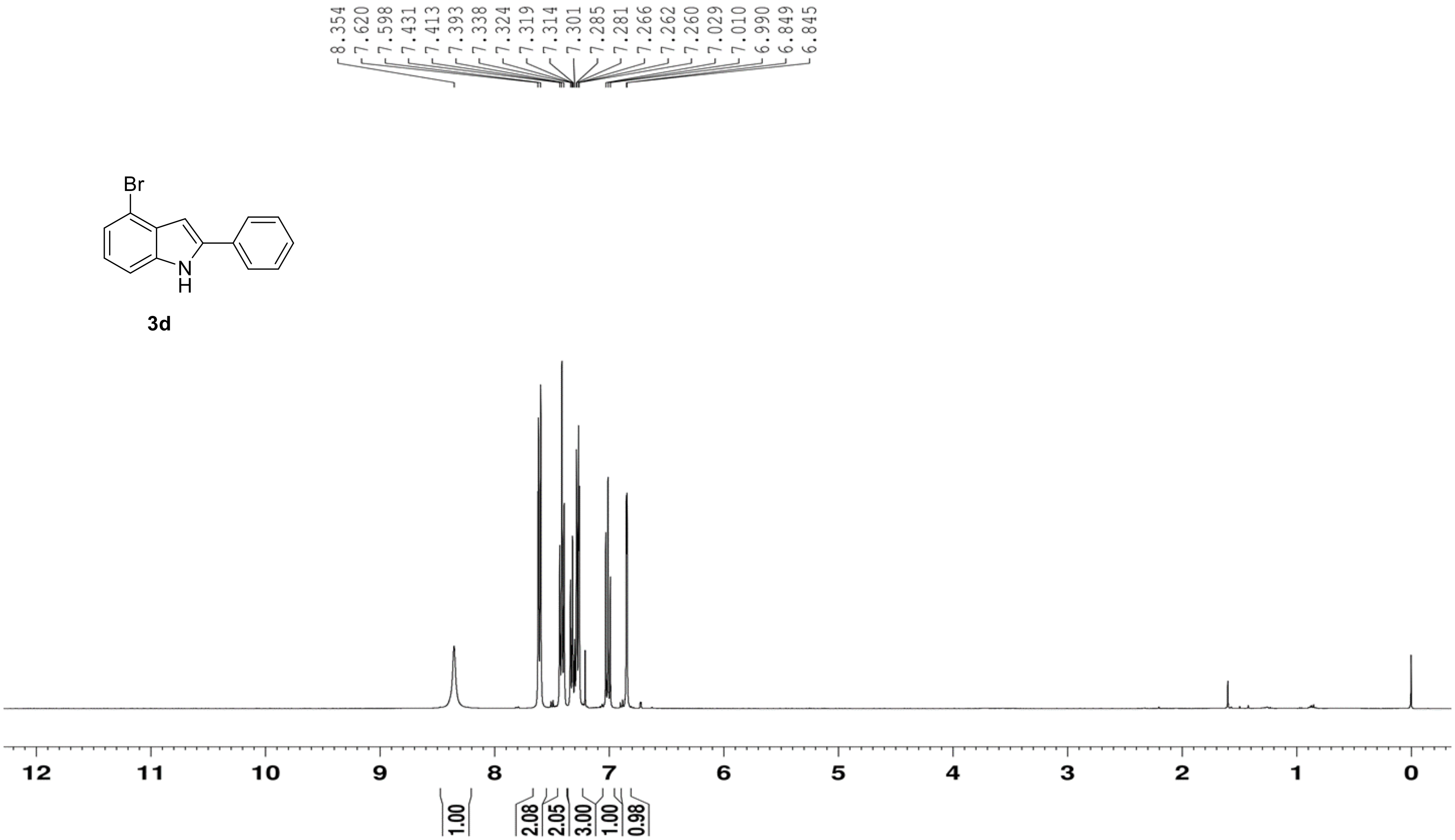

Supplementary Figure 55. ^{1}H NMR spectrum of **3d**.

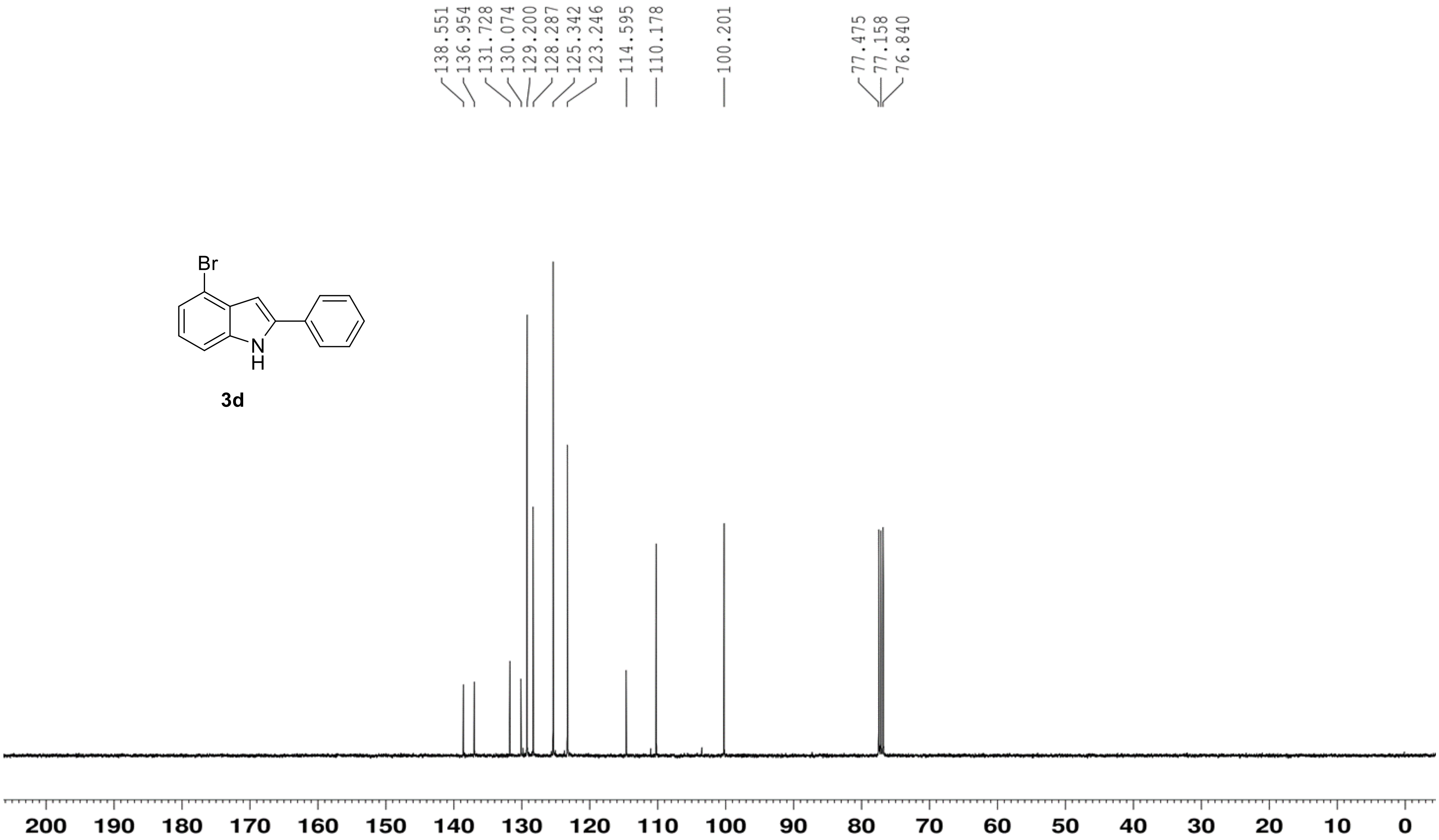

Supplementary Figure 56. ^{13}C NMR spectrum of **3d**.

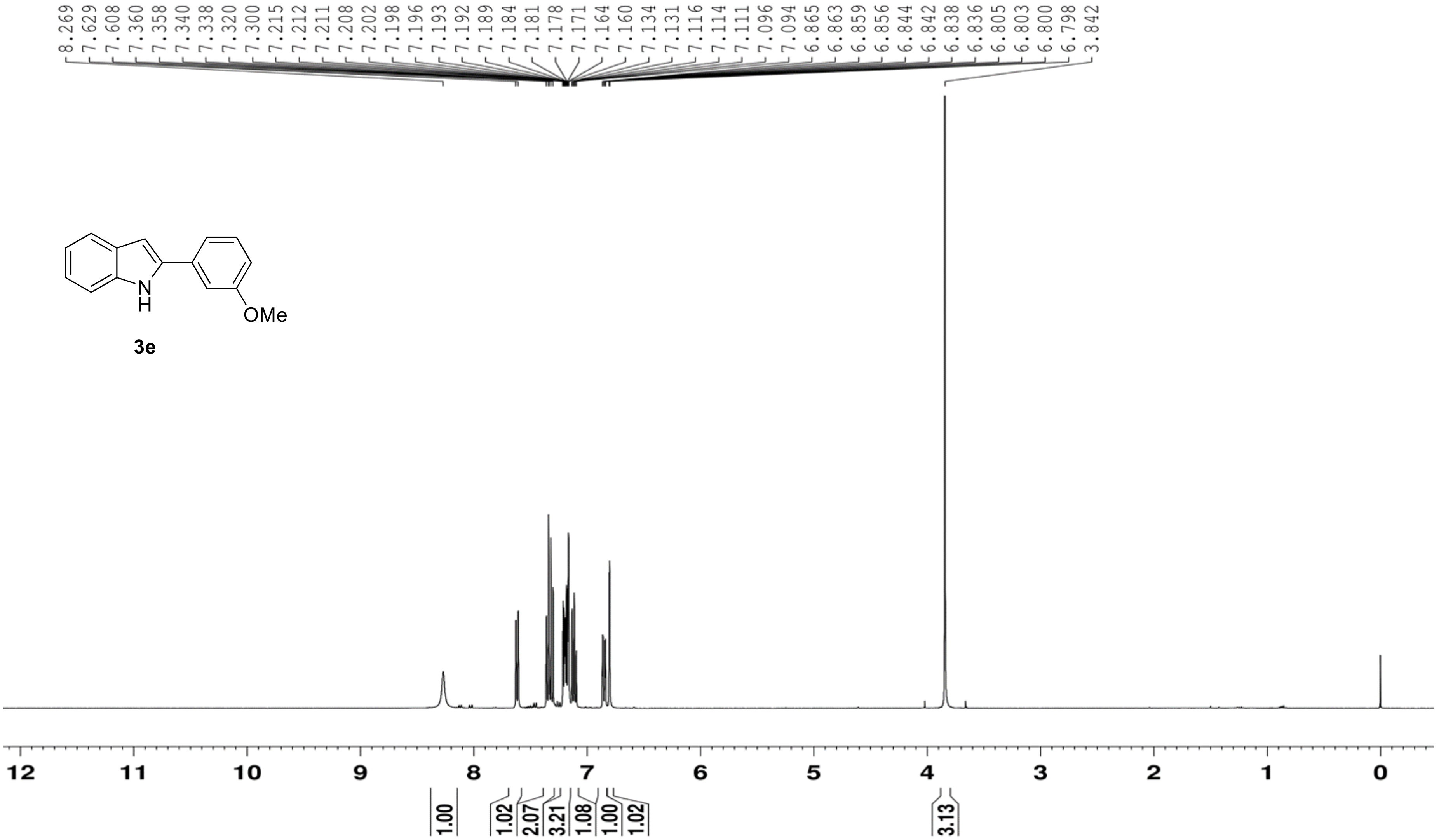

Supplementary Figure 57. ^{1}H NMR spectrum of **3e**.

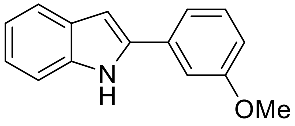

3e

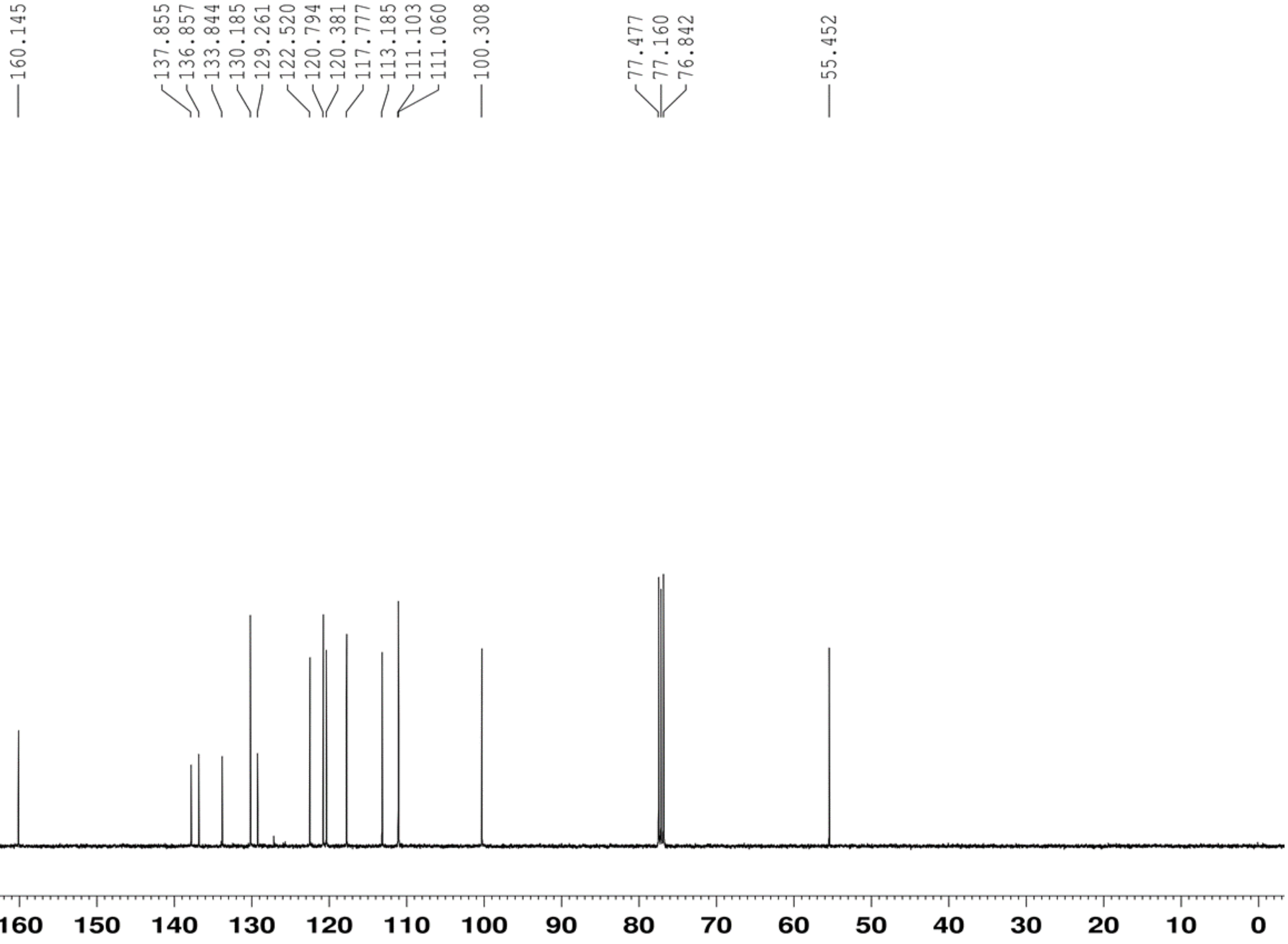

Supplementary Figure 58. ^{13}C NMR spectrum of **3e**.

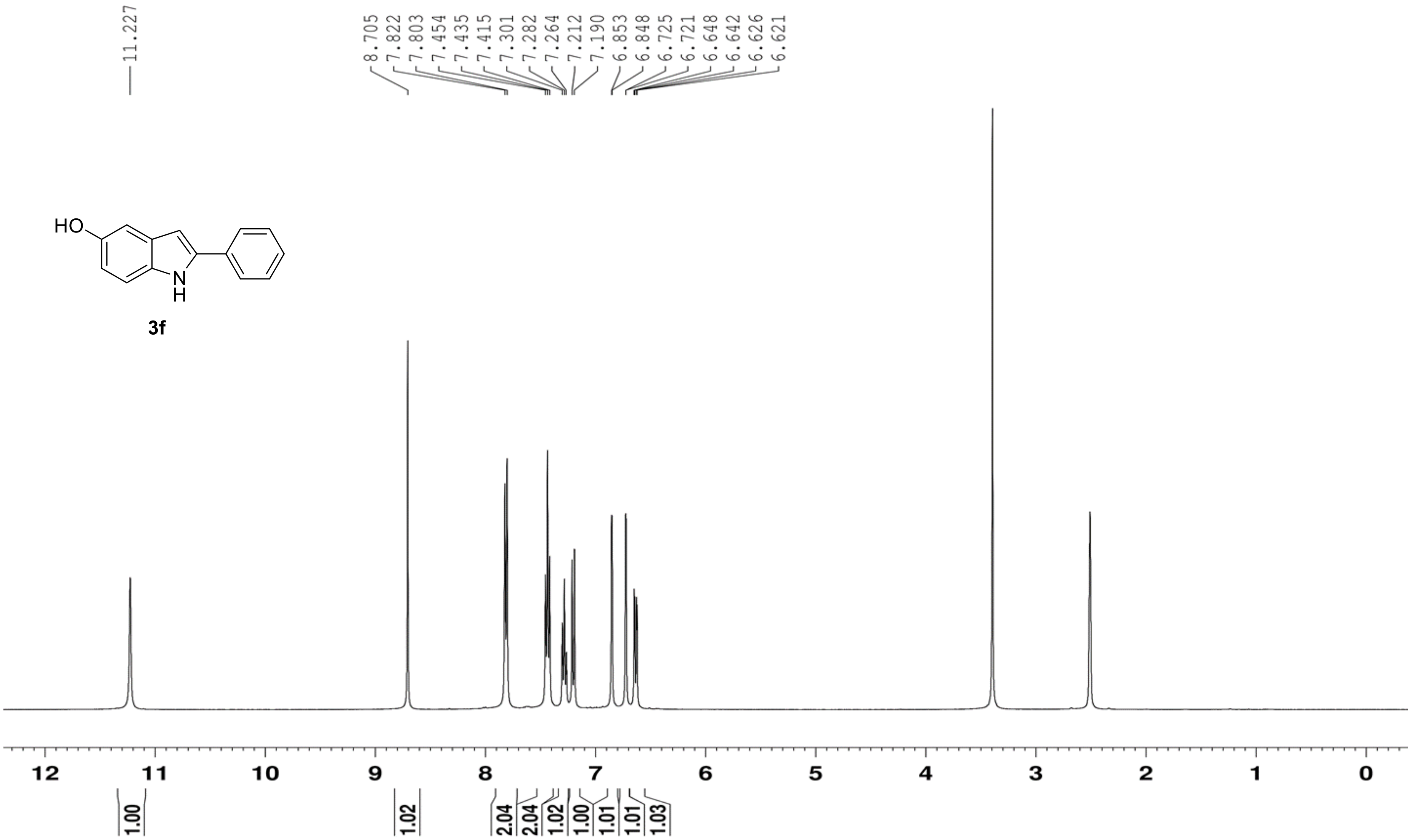

Supplementary Figure 59. ^{1}H NMR spectrum of **3f**.

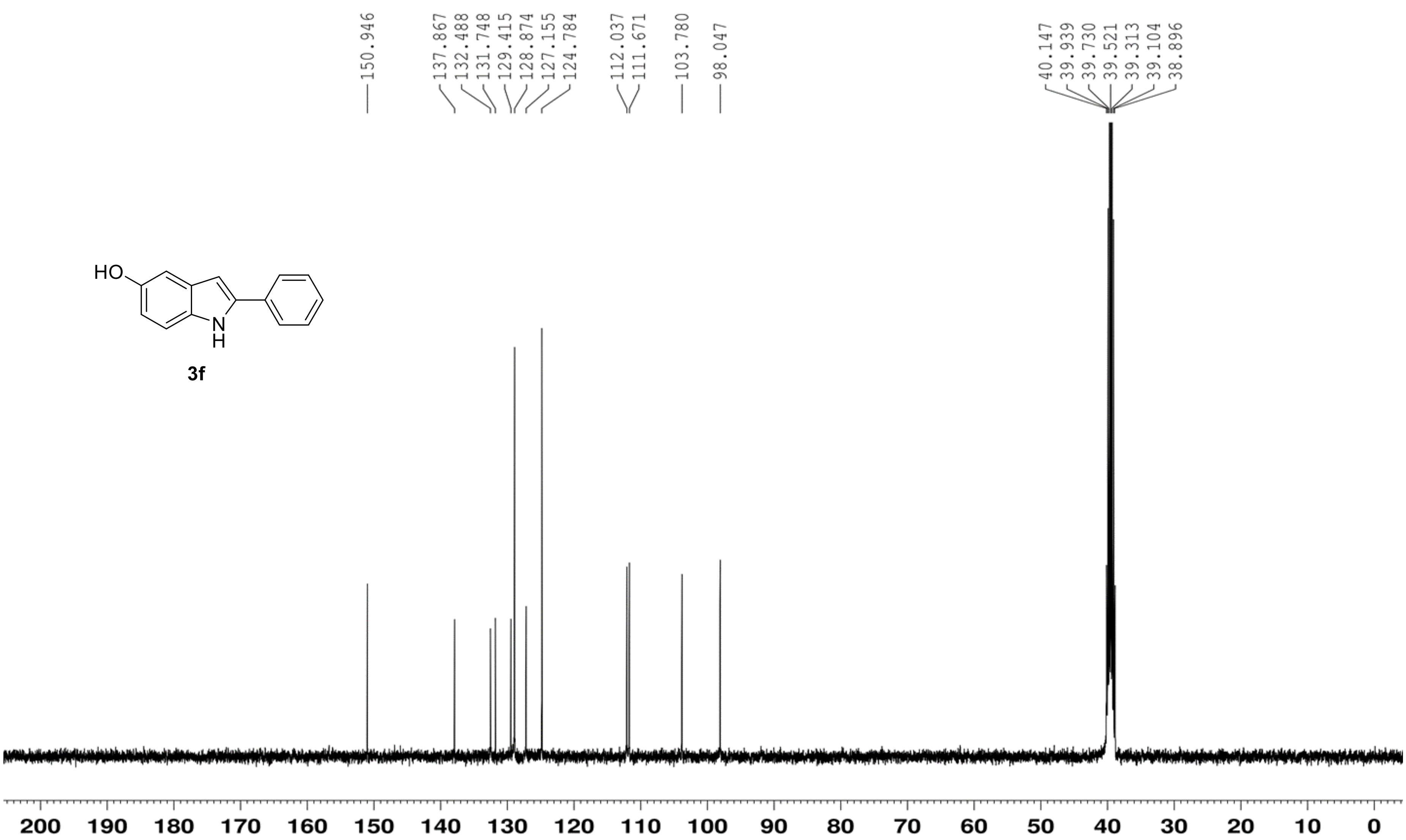

Supplementary Figure 60. ^{13}C NMR spectrum of **3f**.

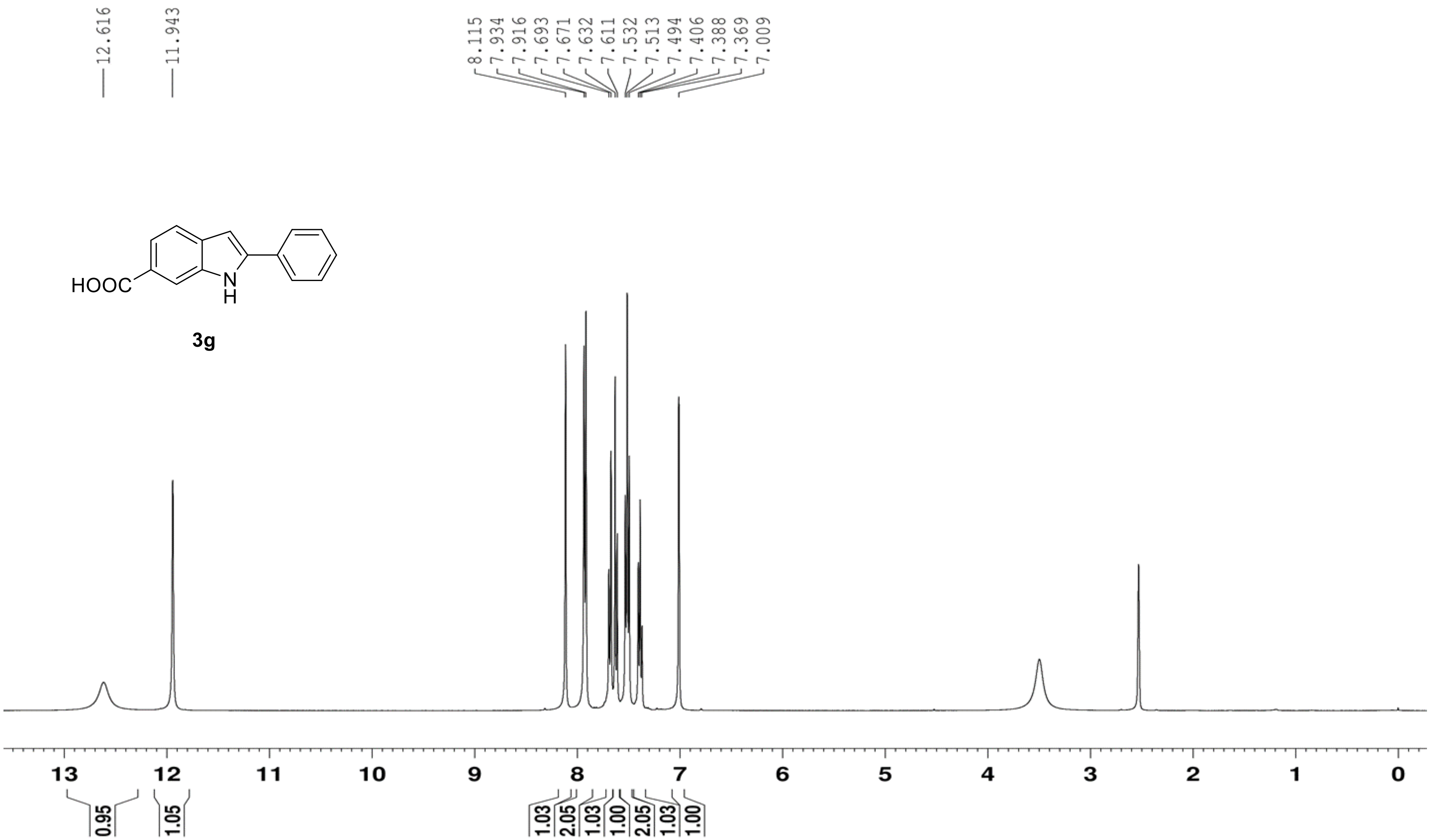

Supplementary Figure 61. ^{1}H NMR spectrum of **3g**.

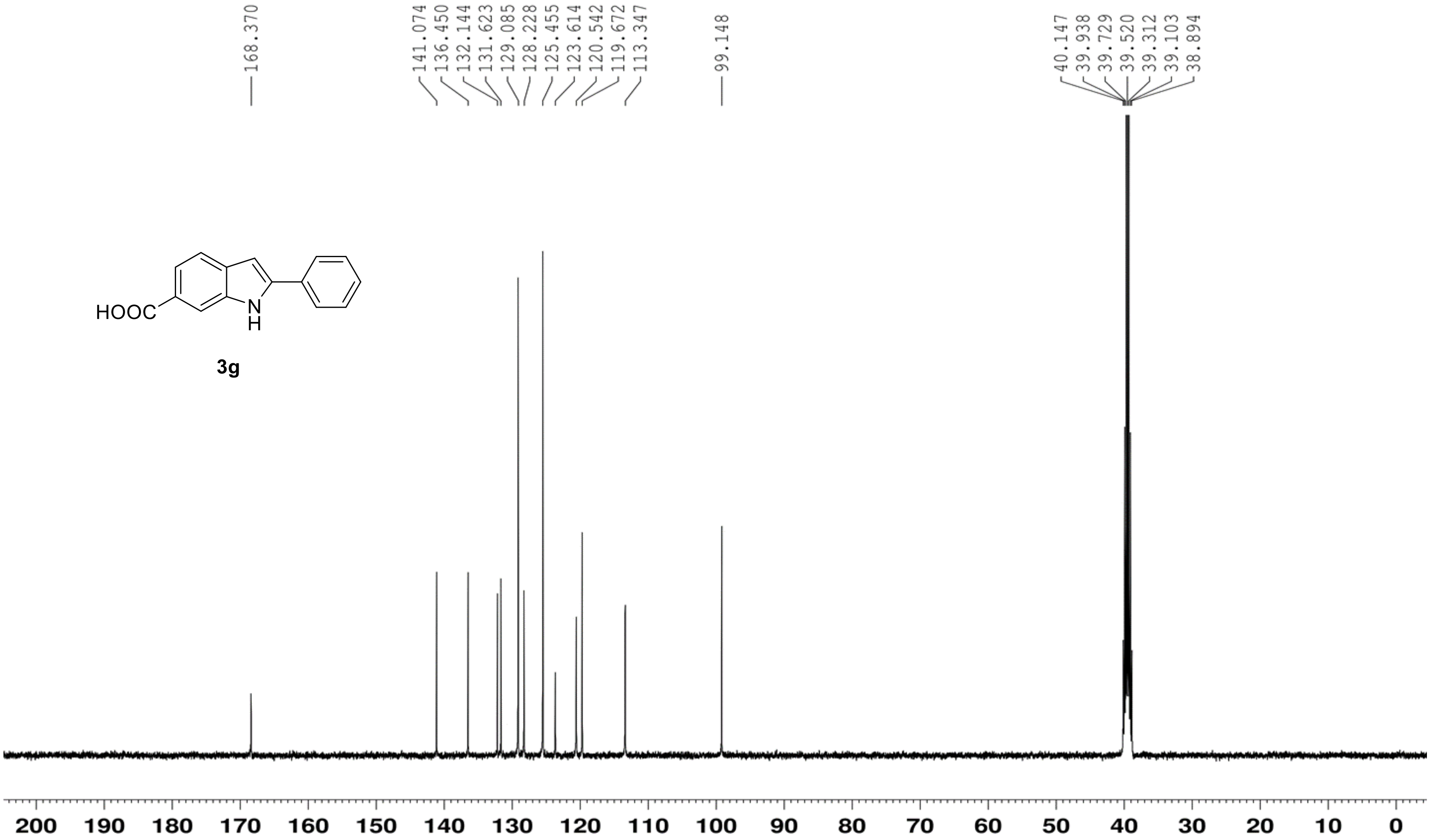

Supplementary Figure 62. ^{13}C NMR spectrum of **3g**.

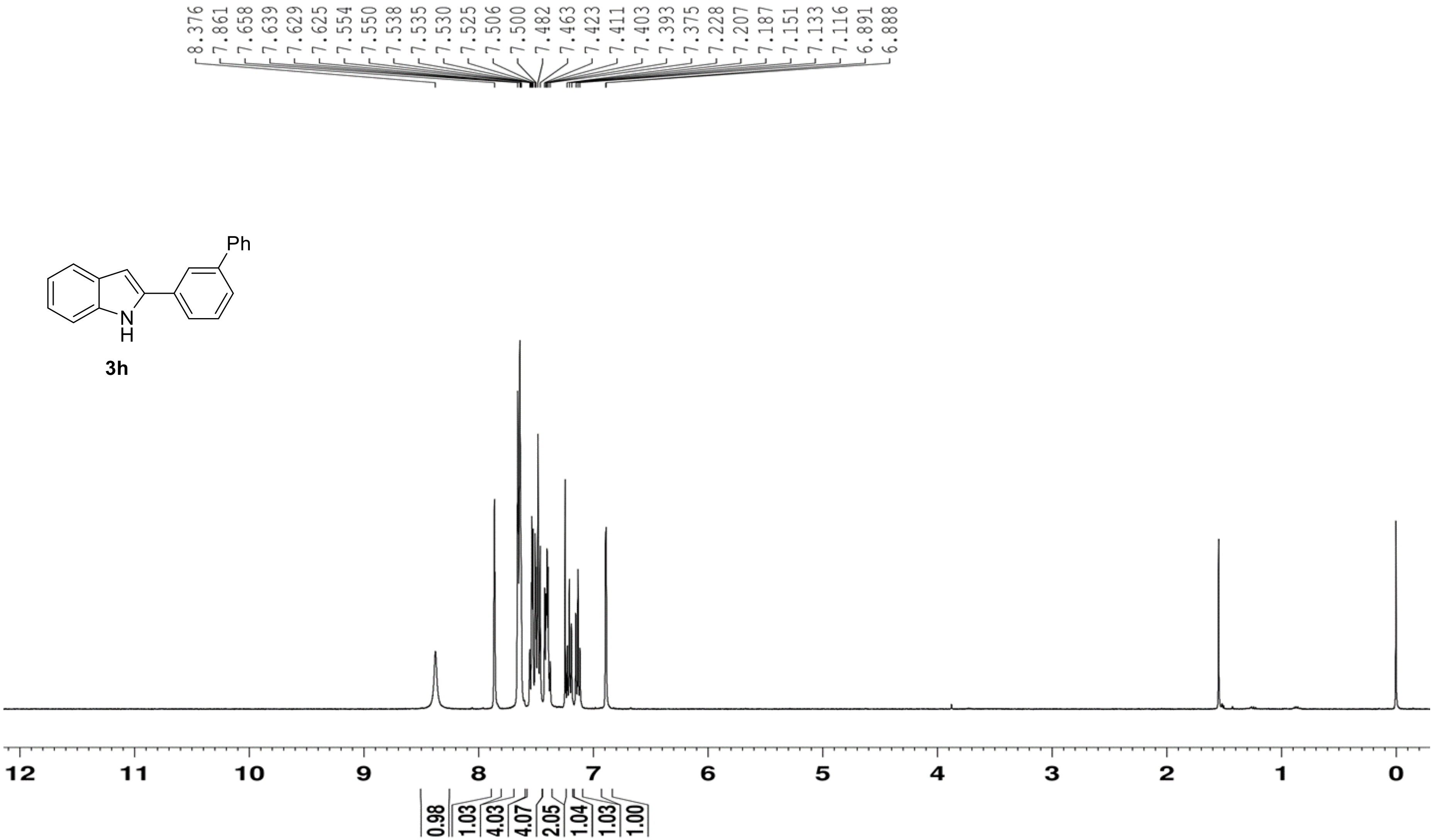

Supplementary Figure 63. ^{1}H NMR spectrum of **3h**.

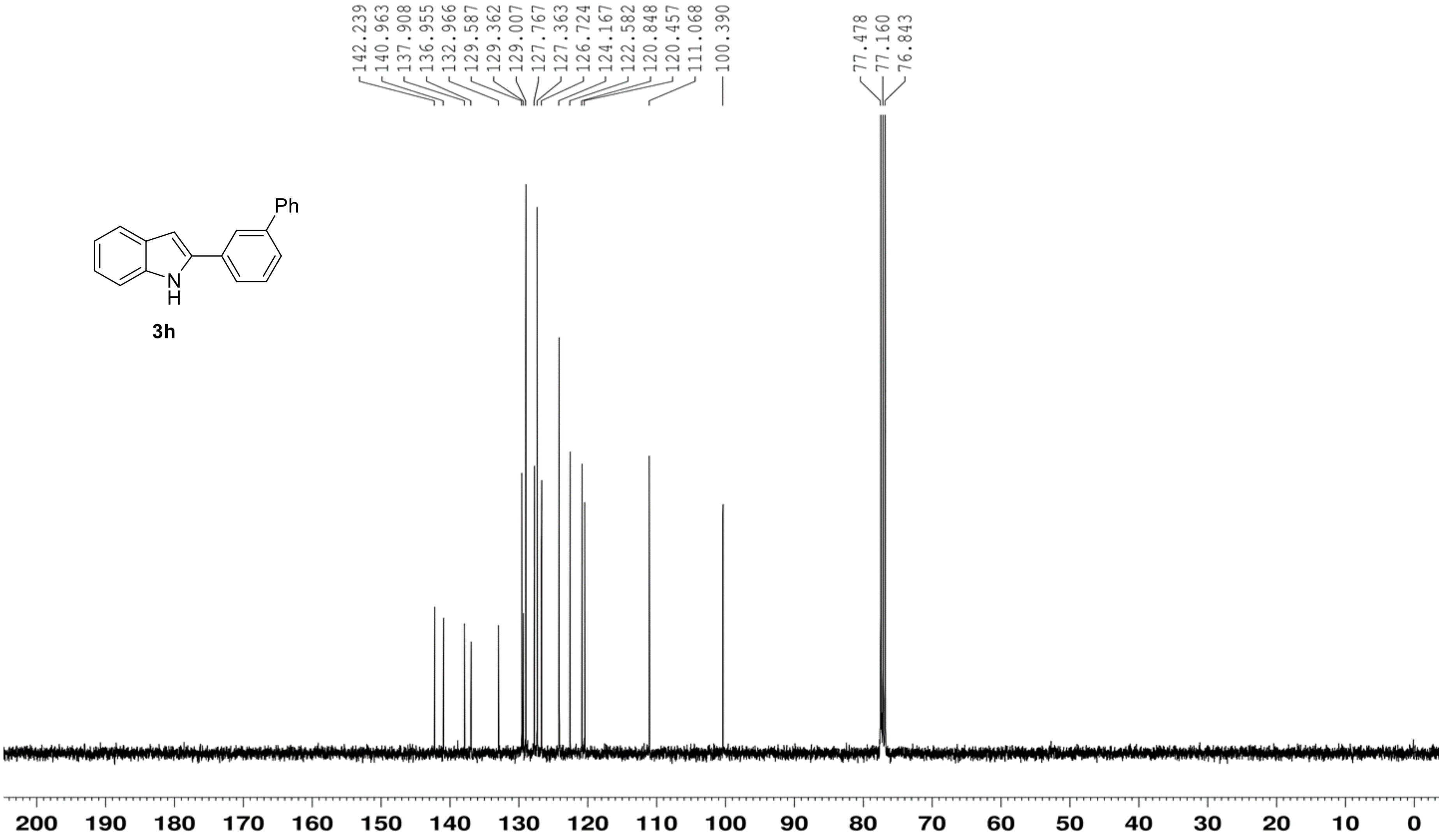

3h

Supplementary Figure 64. ^{13}C NMR spectrum of **3h**.

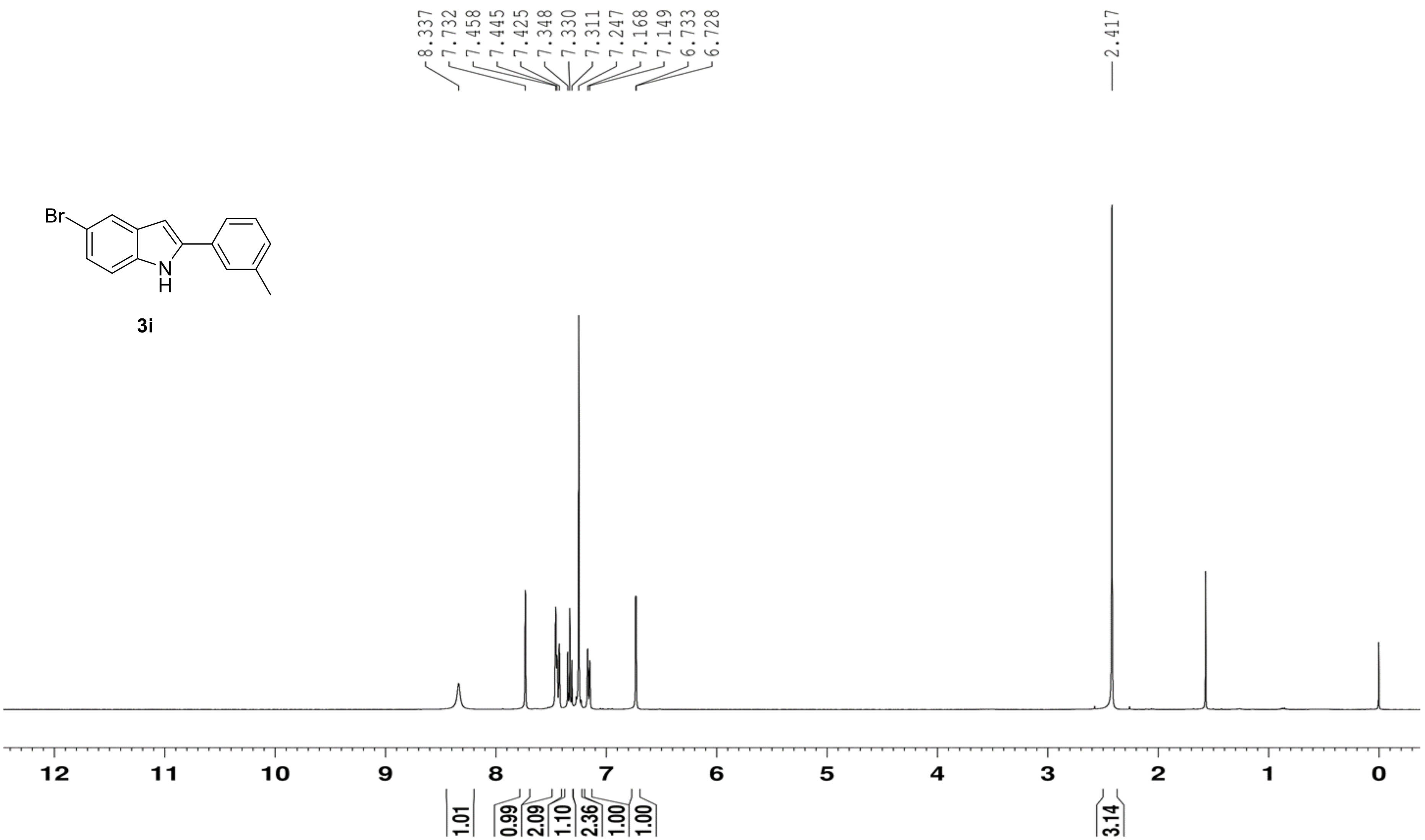

Supplementary Figure 65. ^{1}H NMR spectrum of **3i**.

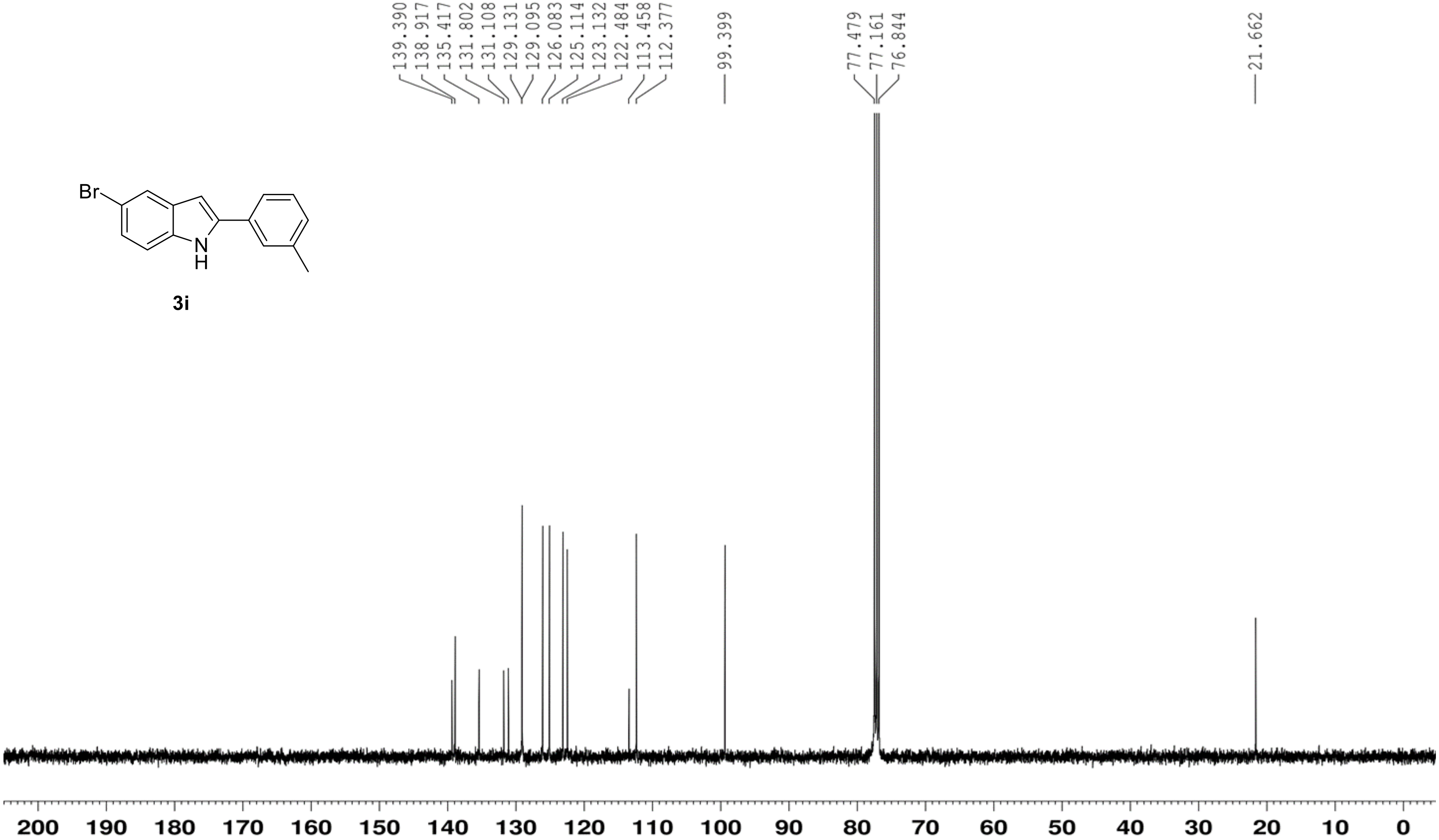

3i

Supplementary Figure 66. ^{13}C NMR spectrum of **3i**.

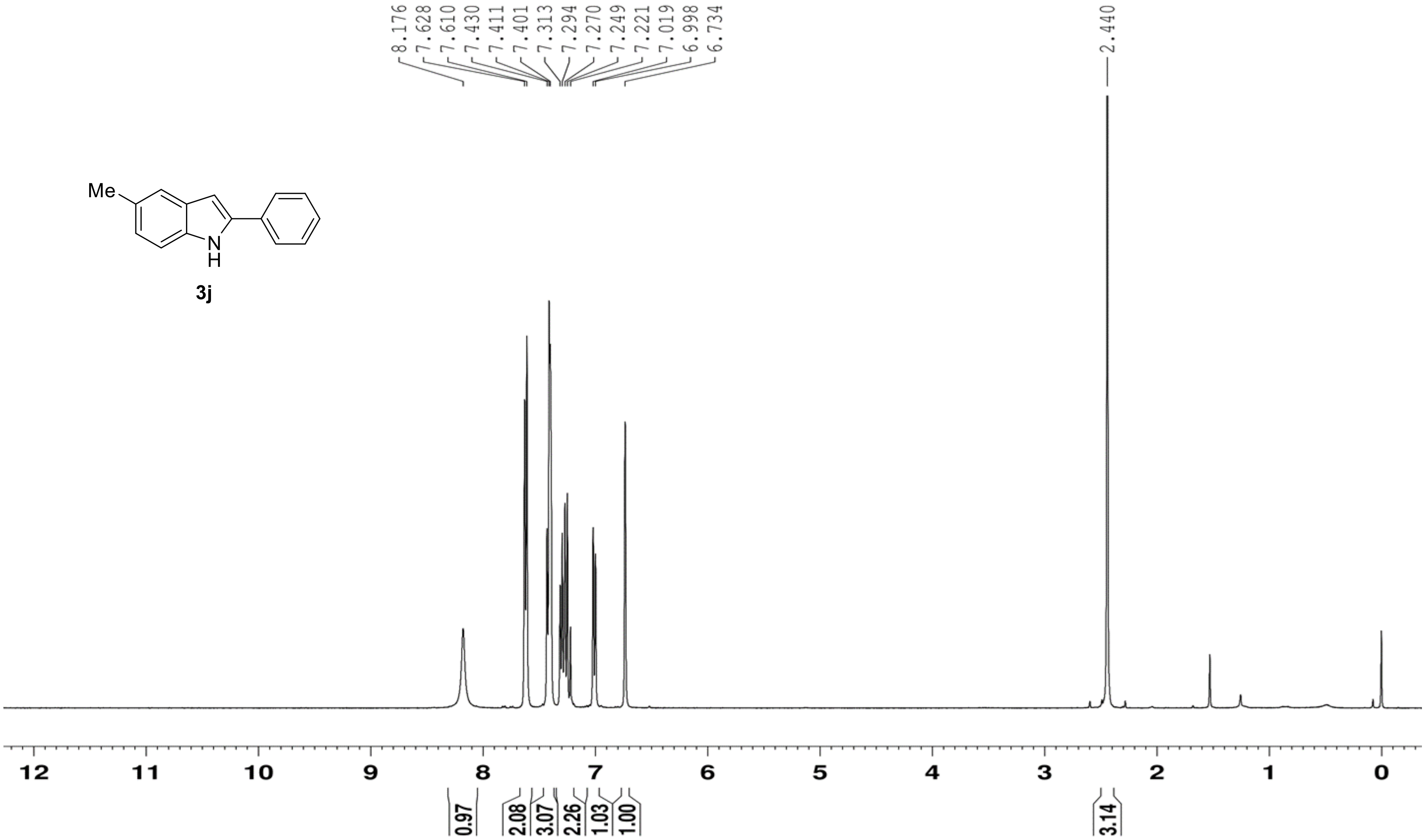

Supplementary Figure 67. ^{1}H NMR spectrum of **3j**.

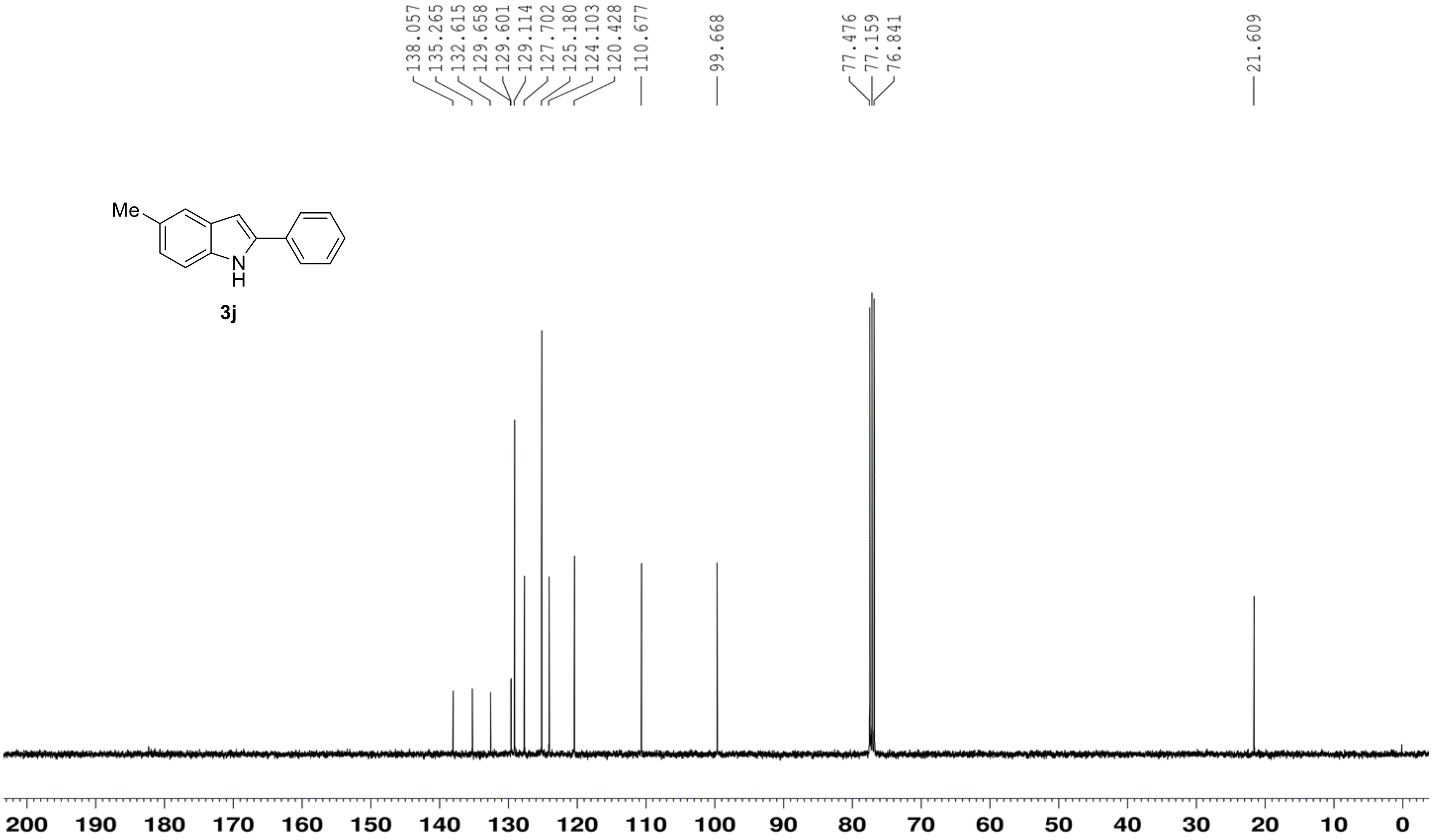

Supplementary Figure 68. ^{13}C NMR spectrum of **3j**.

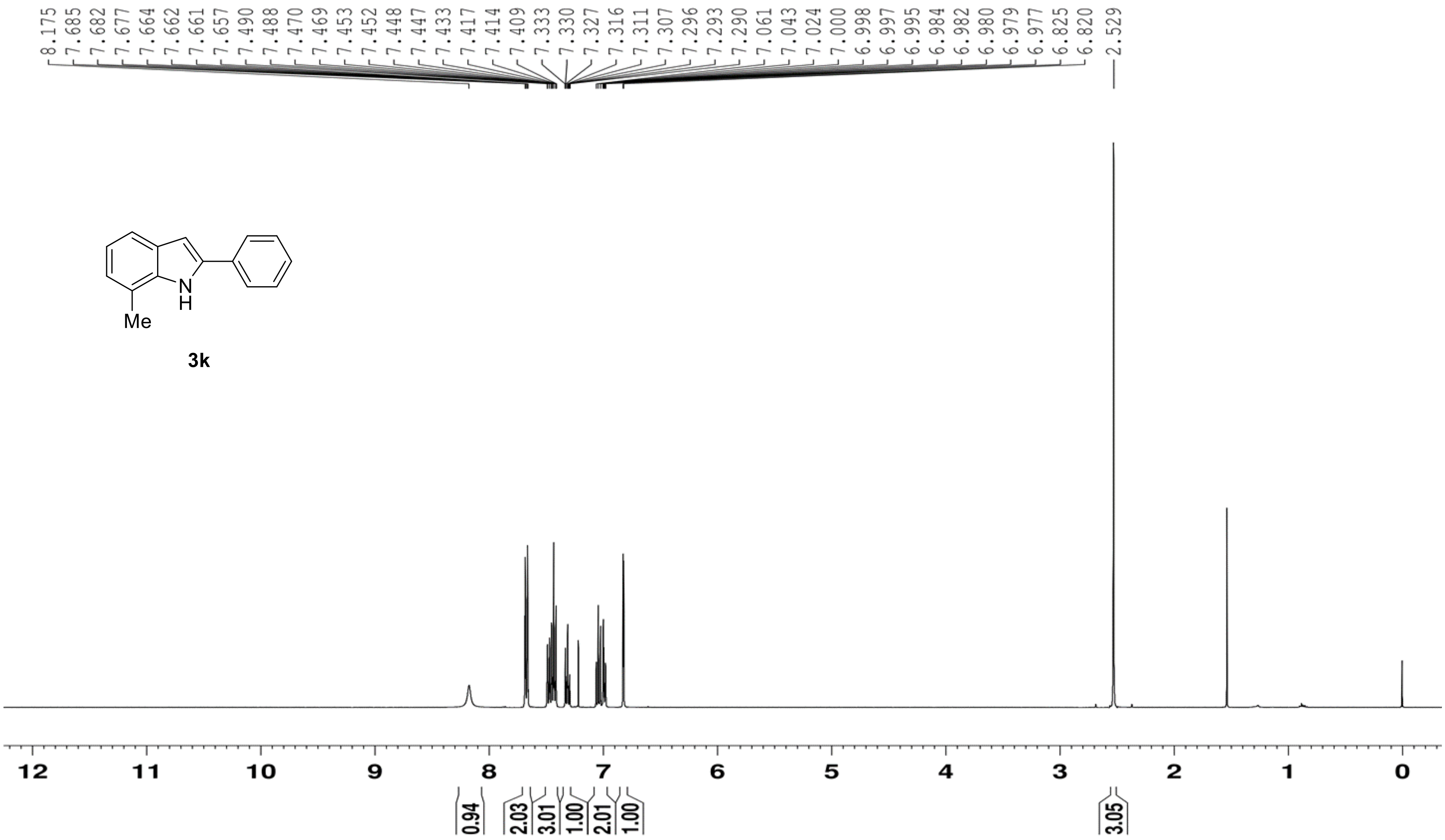

Supplementary Figure 69. ^{1}H NMR spectrum of **3k**.

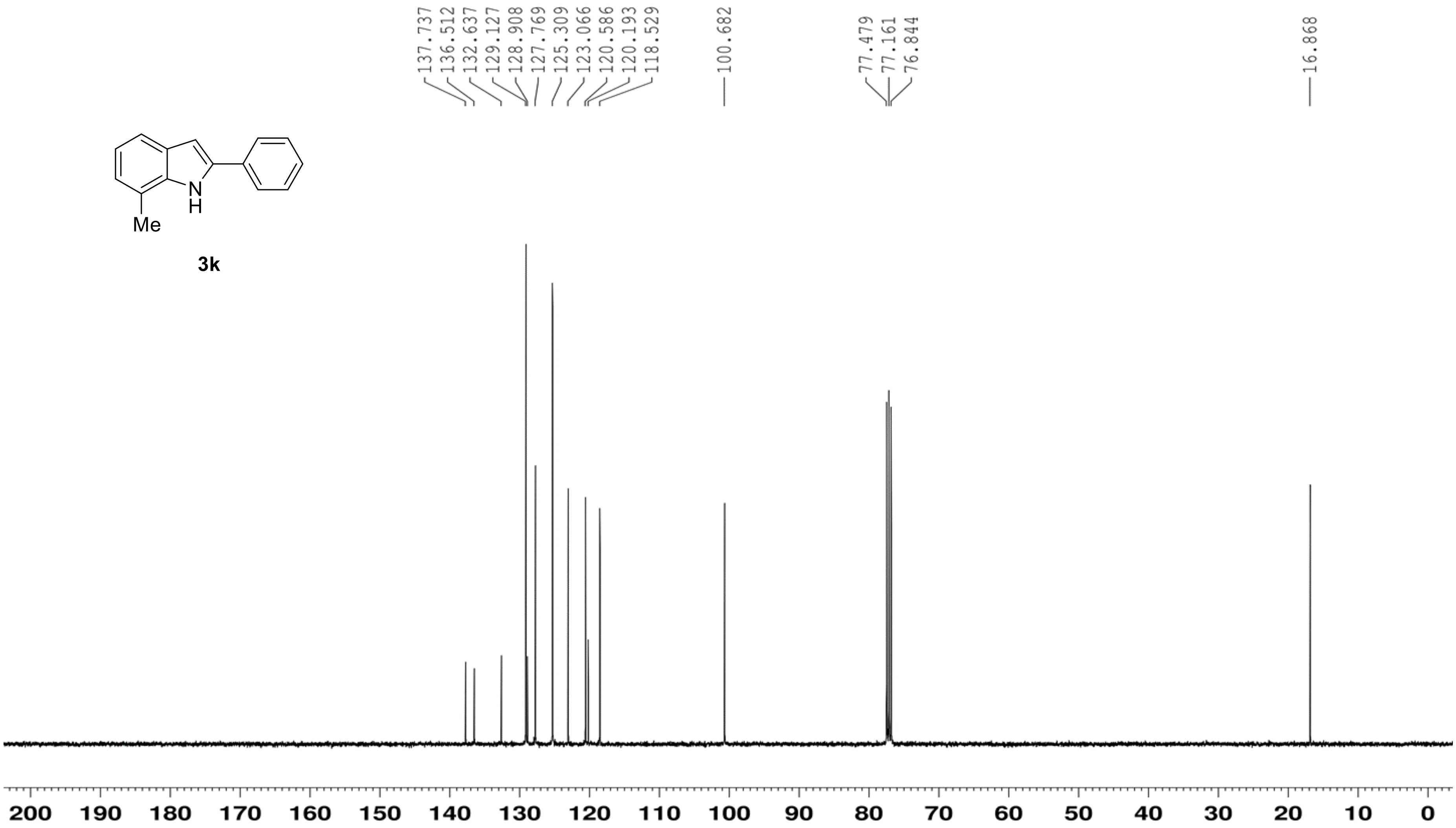

Supplementary Figure 70. ^{13}C NMR spectrum of **3k**.

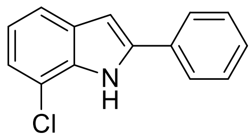

3l

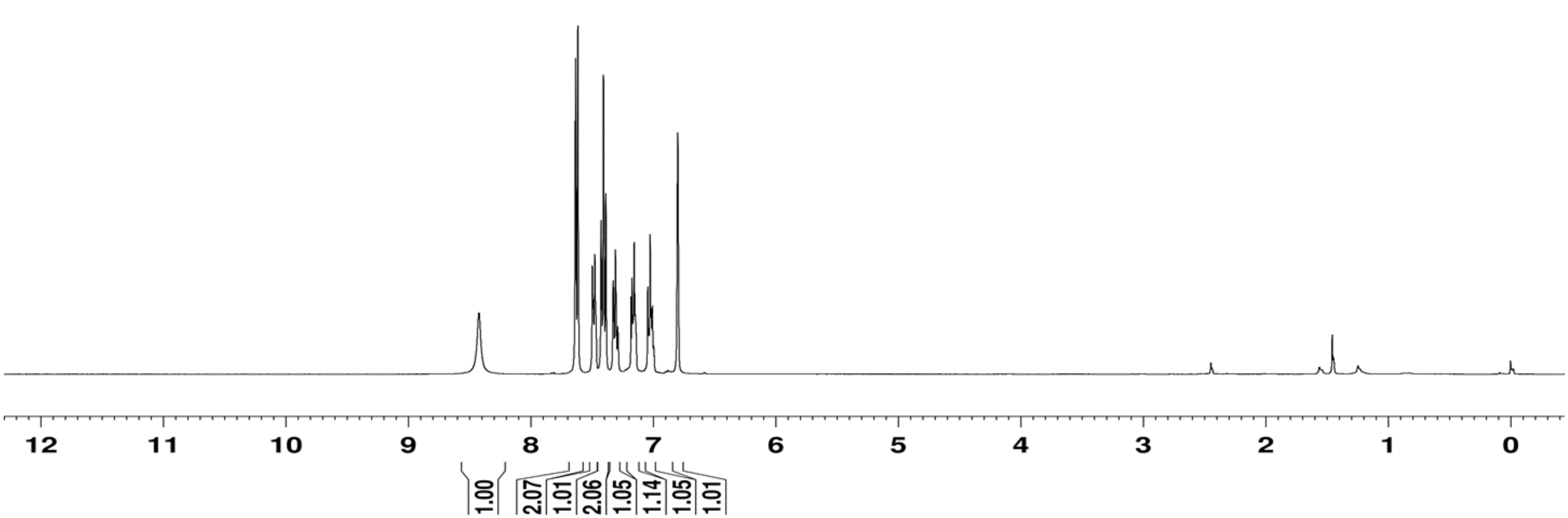

Supplementary Figure 71. ^{1}H NMR spectrum of **3l**.

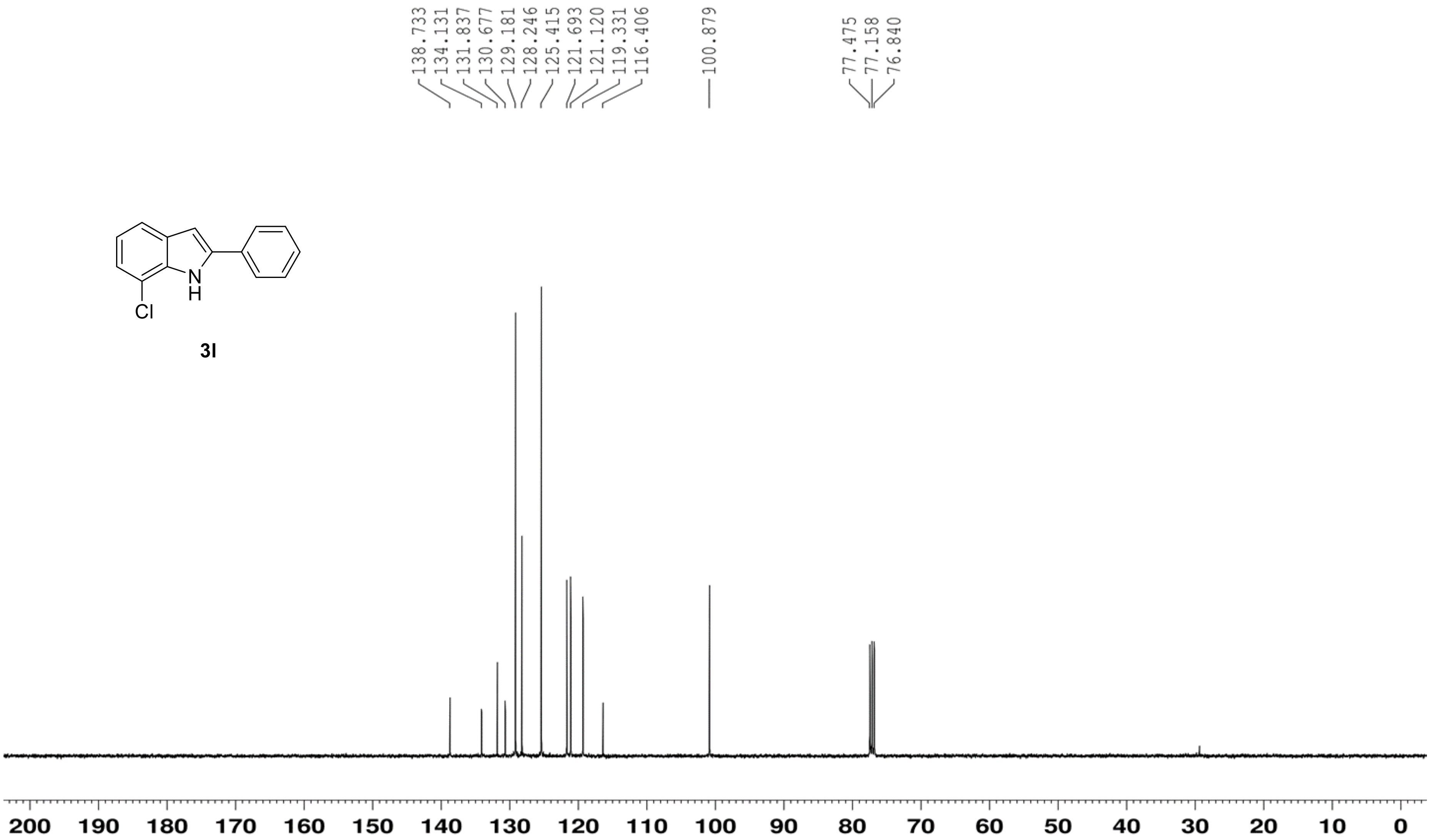

Supplementary Figure 72. ^{13}C NMR spectrum of **3l**.

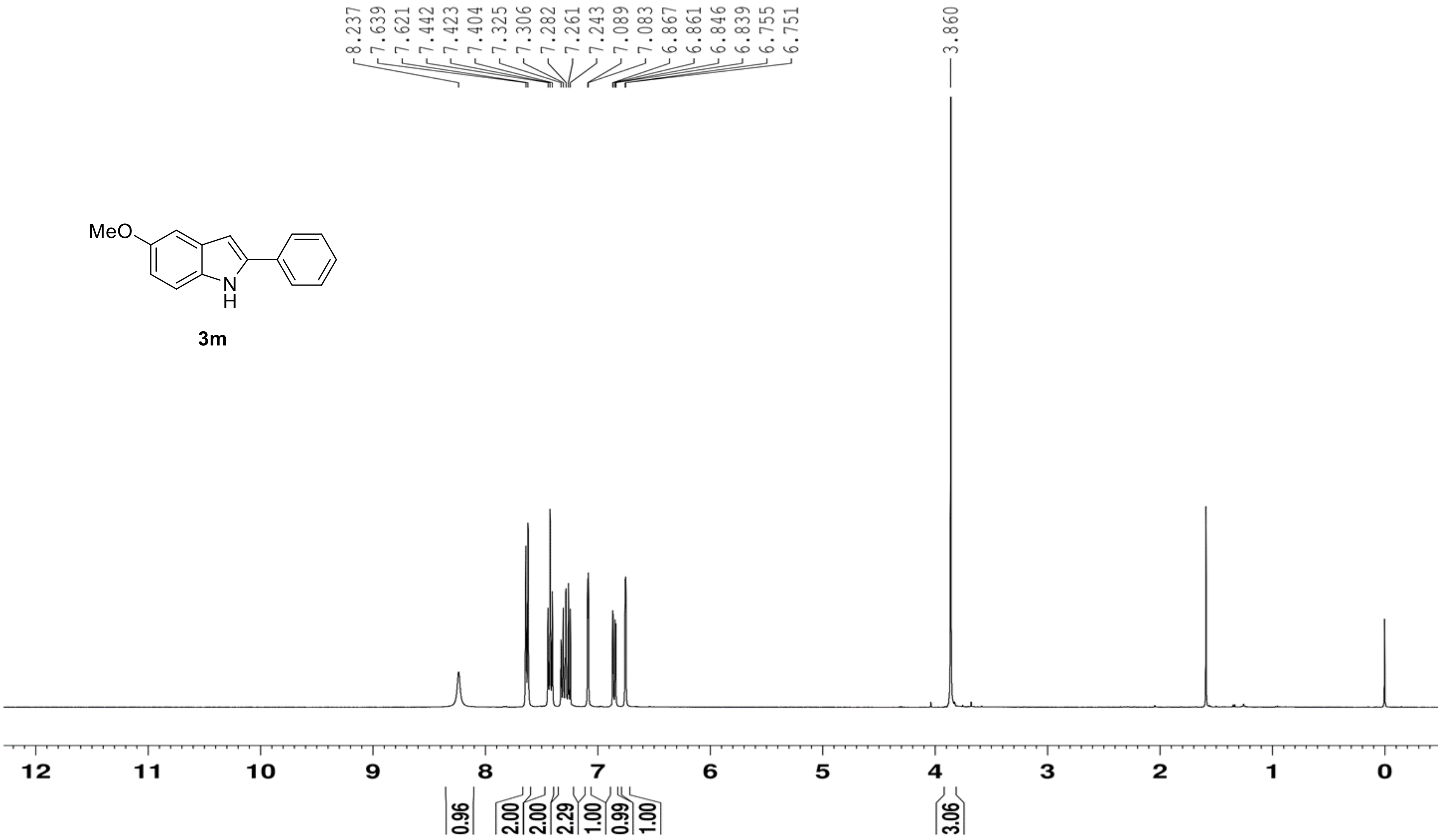

Supplementary Figure 73. ^{1}H NMR spectrum of **3m**.

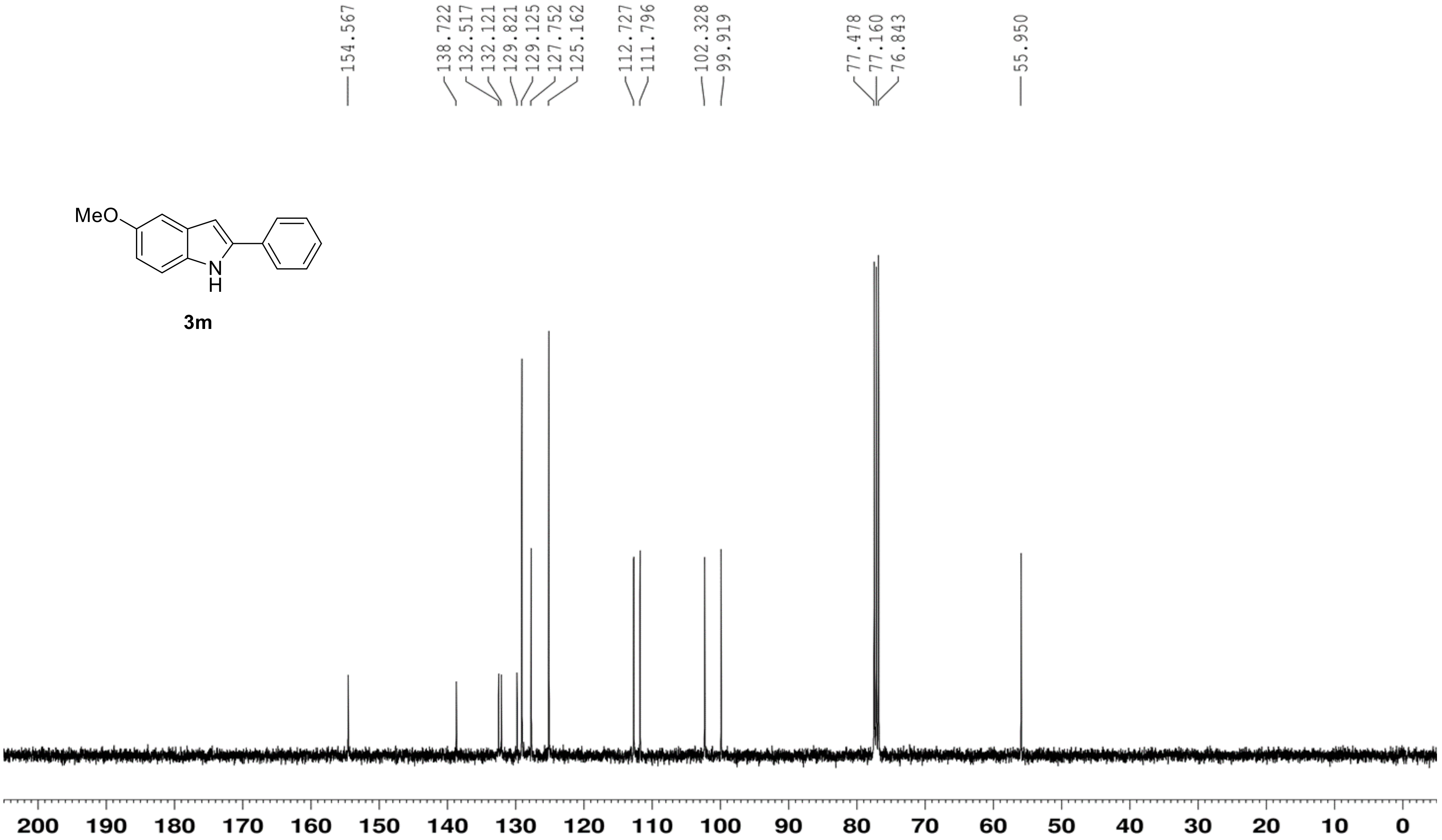

Supplementary Figure 74. ^{13}C NMR spectrum of **3m**.

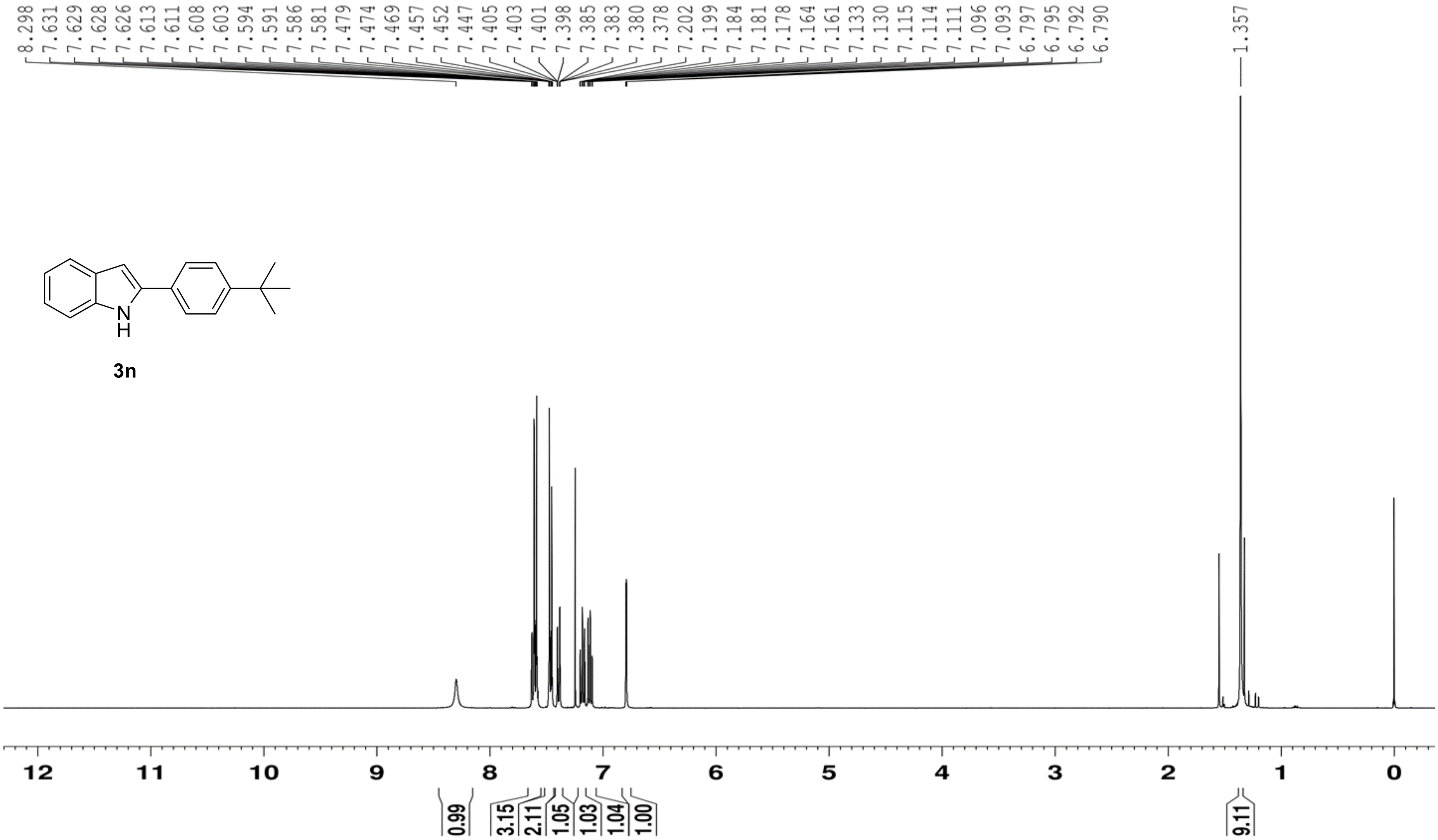

Supplementary Figure 75. ^{1}H NMR spectrum of **3n**.

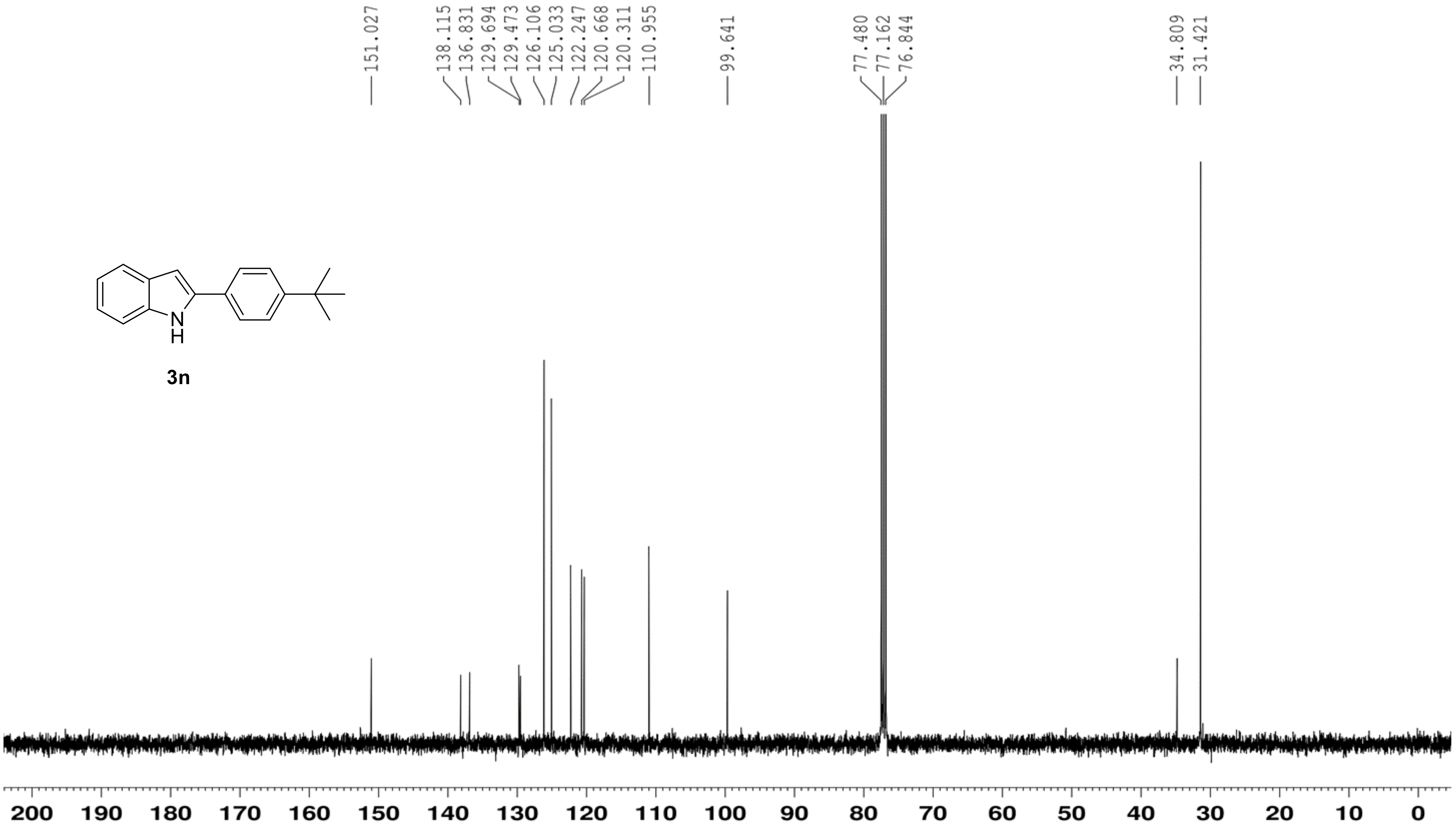

Supplementary Figure 76. ^{13}C NMR spectrum of **3n**.

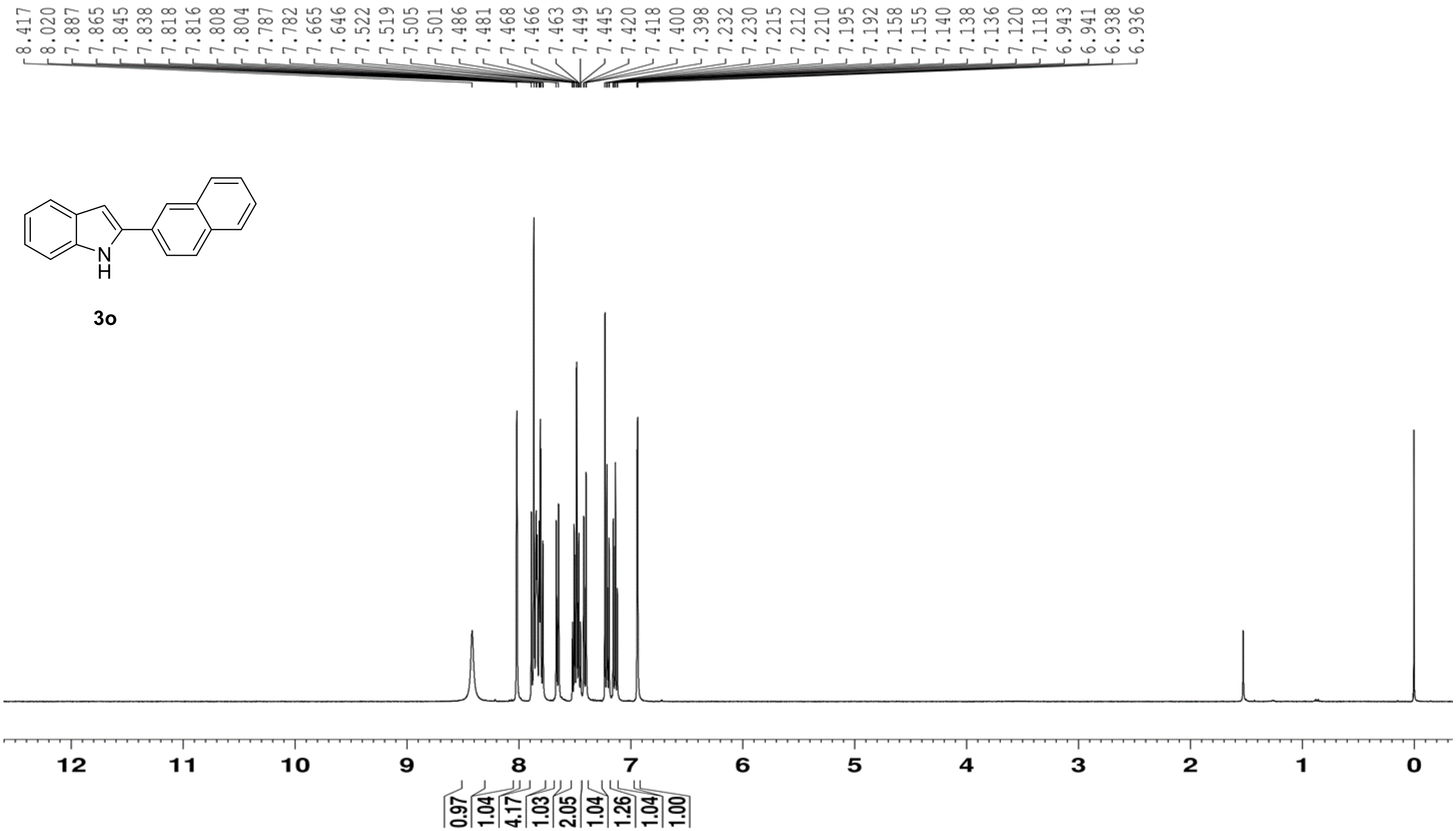

Supplementary Figure 77. ^{1}H NMR spectrum of **3o**.

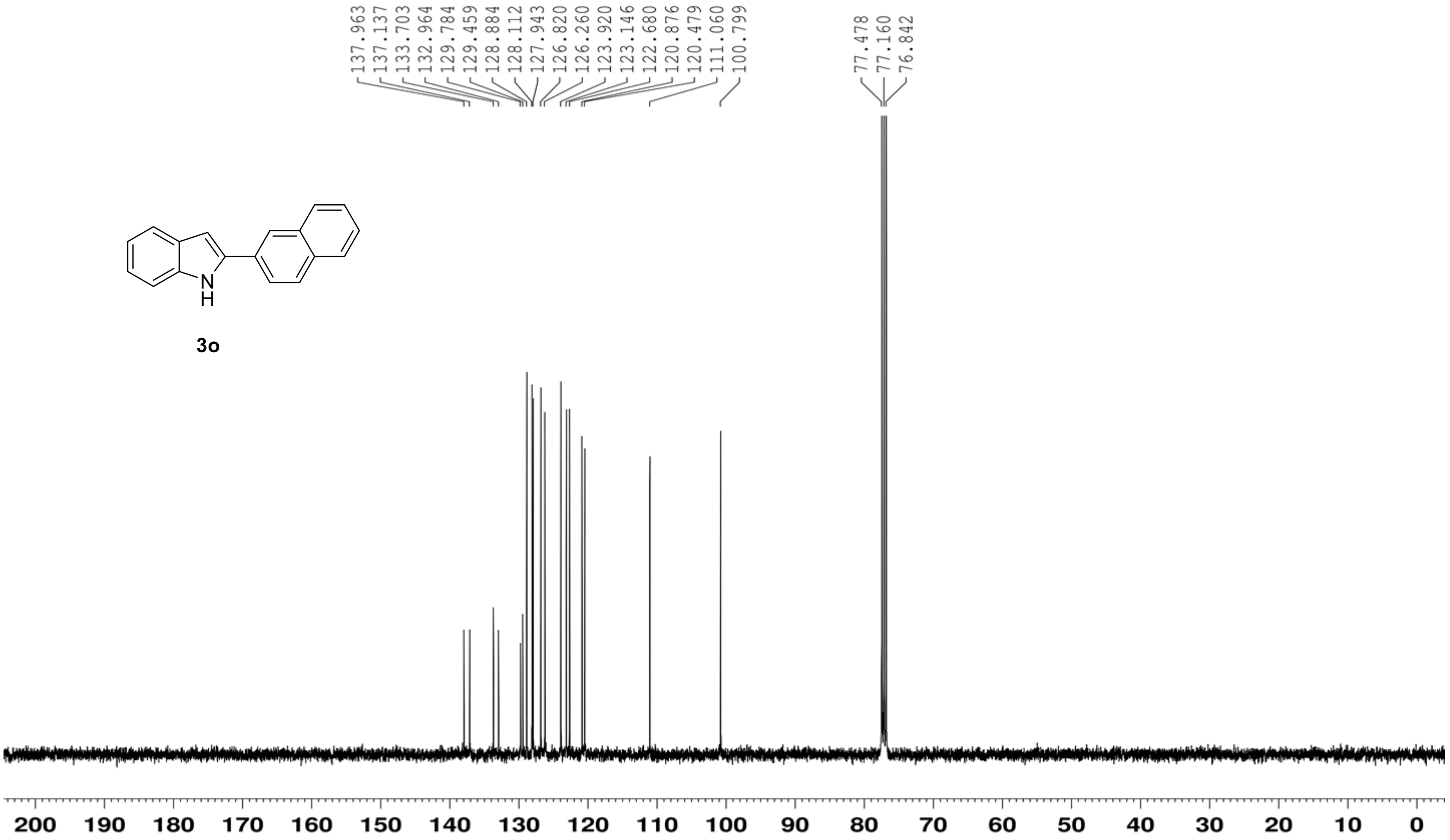

Supplementary Figure 78. ^{13}C NMR spectrum of **3o**.

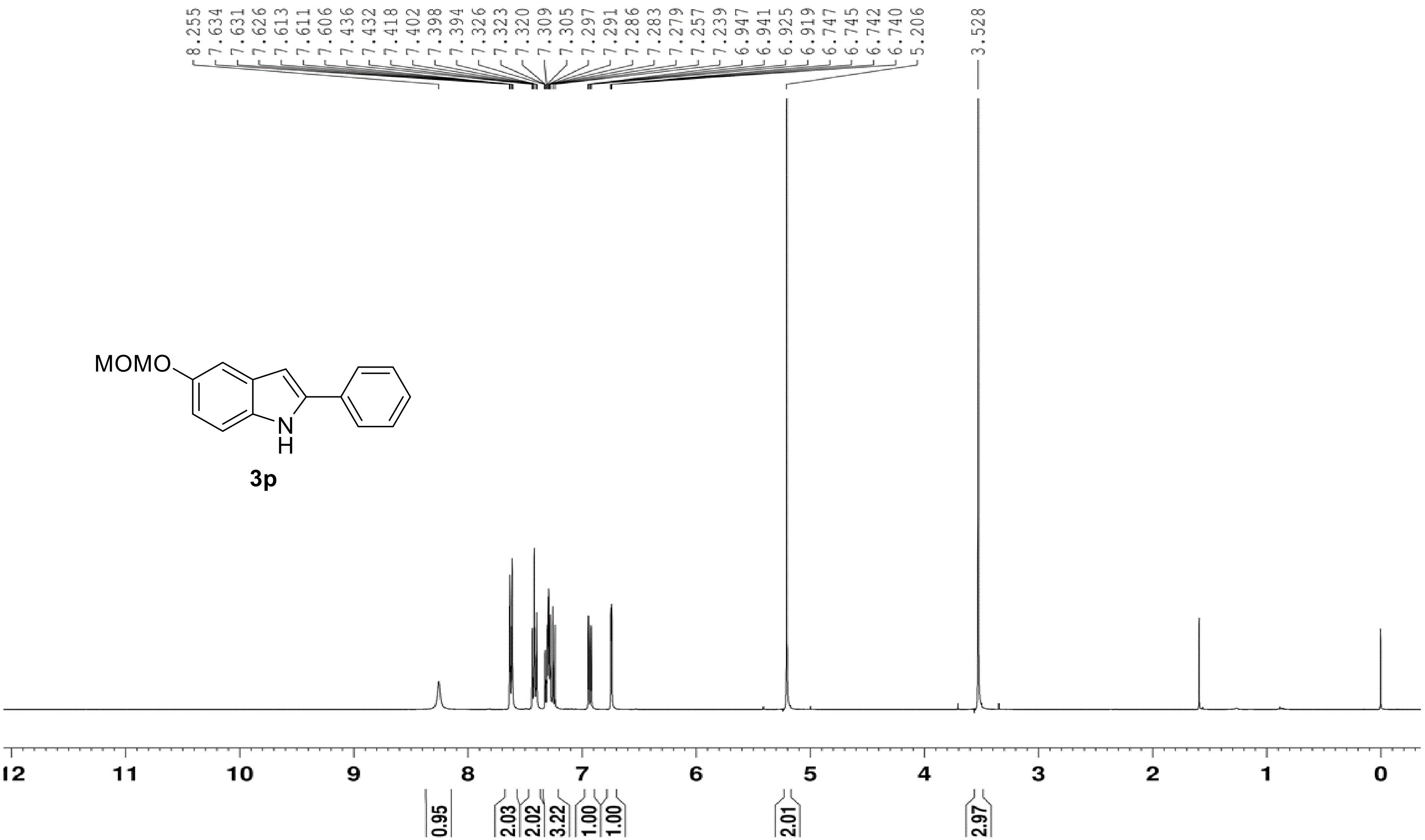

Supplementary Figure 79. ^{1}H NMR spectrum of **3p**.

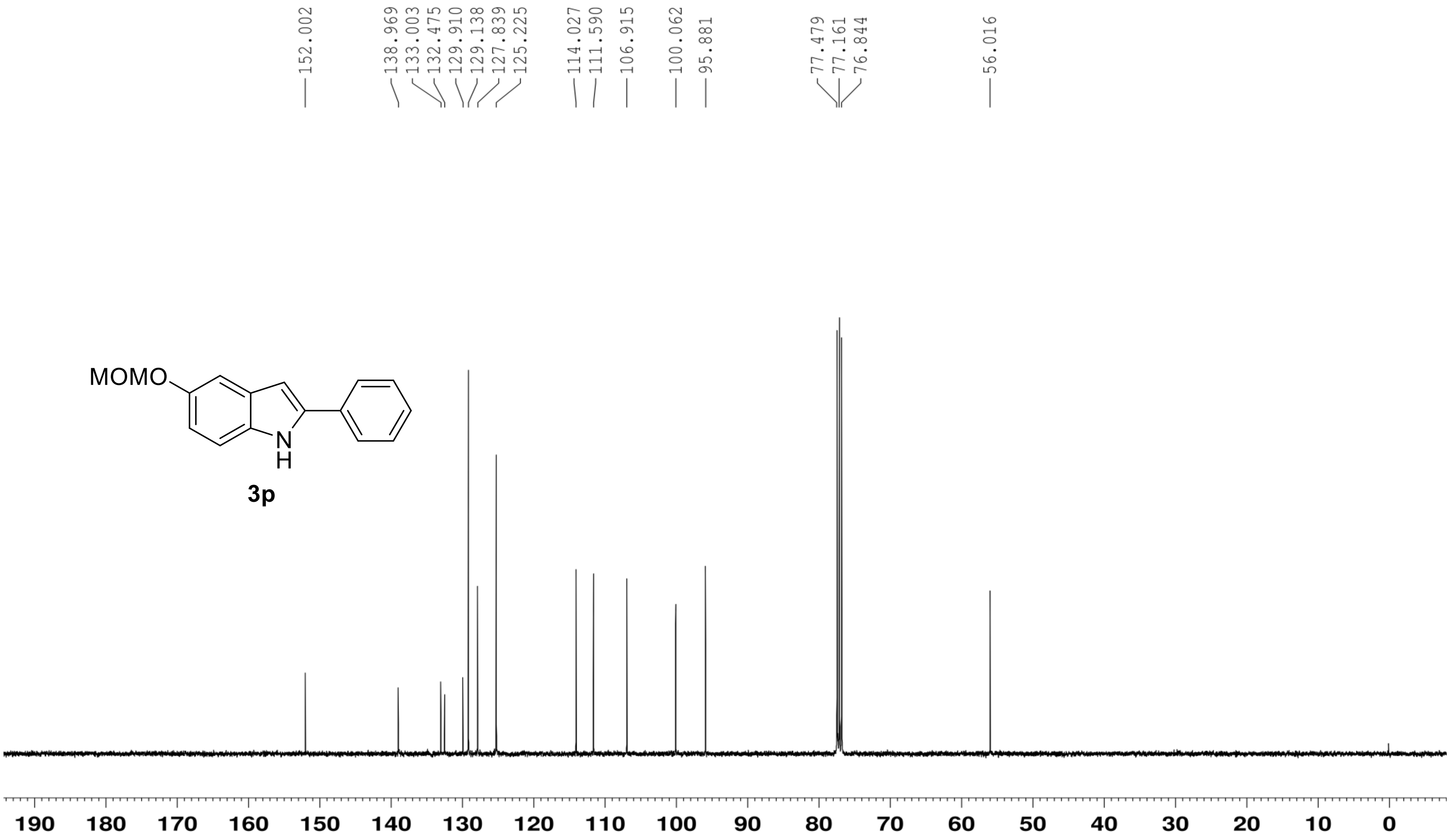

Supplementary Figure 80. ^{13}C NMR spectrum of **3p**.

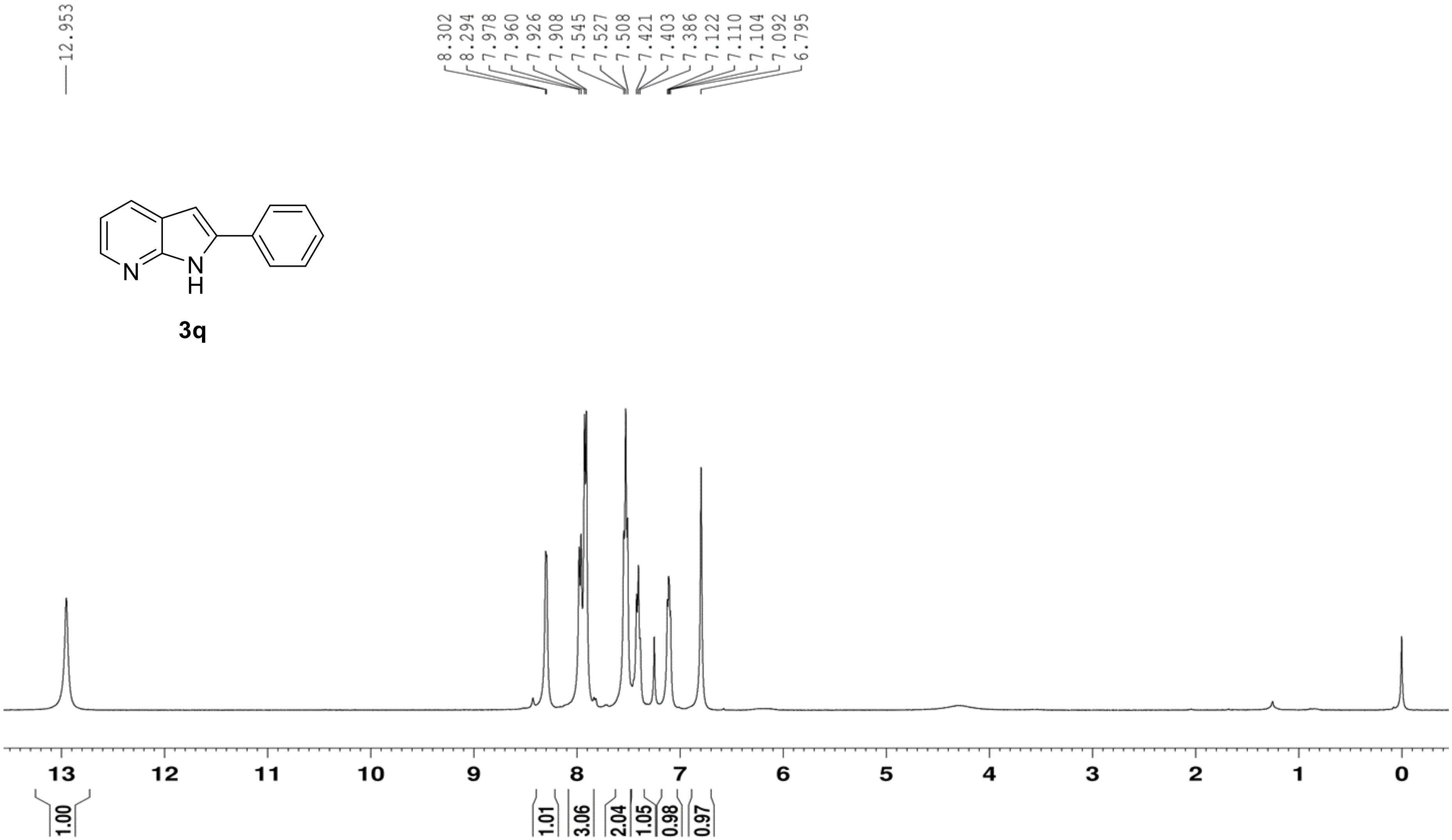

Supplementary Figure 81. ^{1}H NMR spectrum of **3q**.

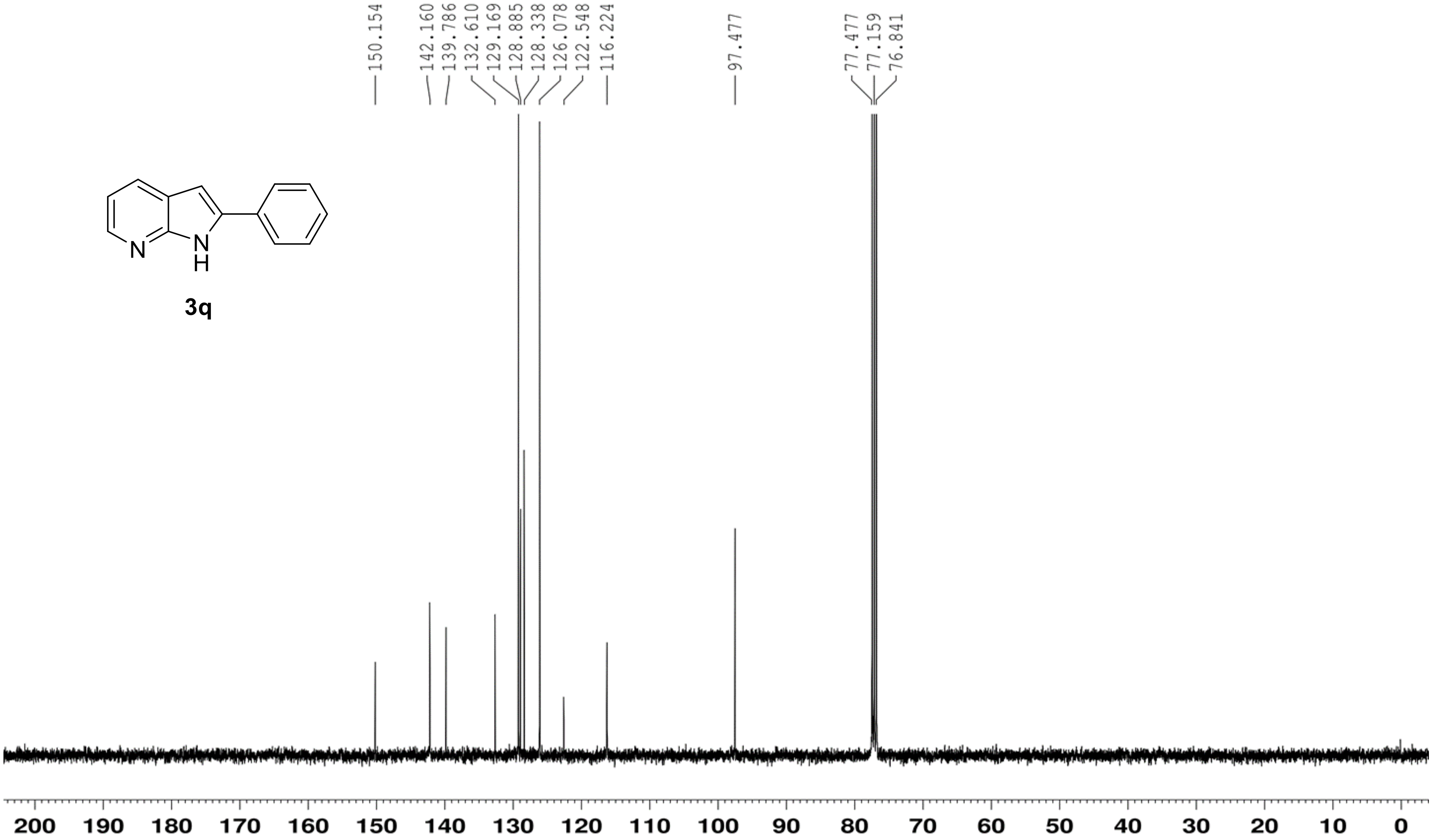

Supplementary Figure 82. ^{13}C NMR spectrum of **3q**.

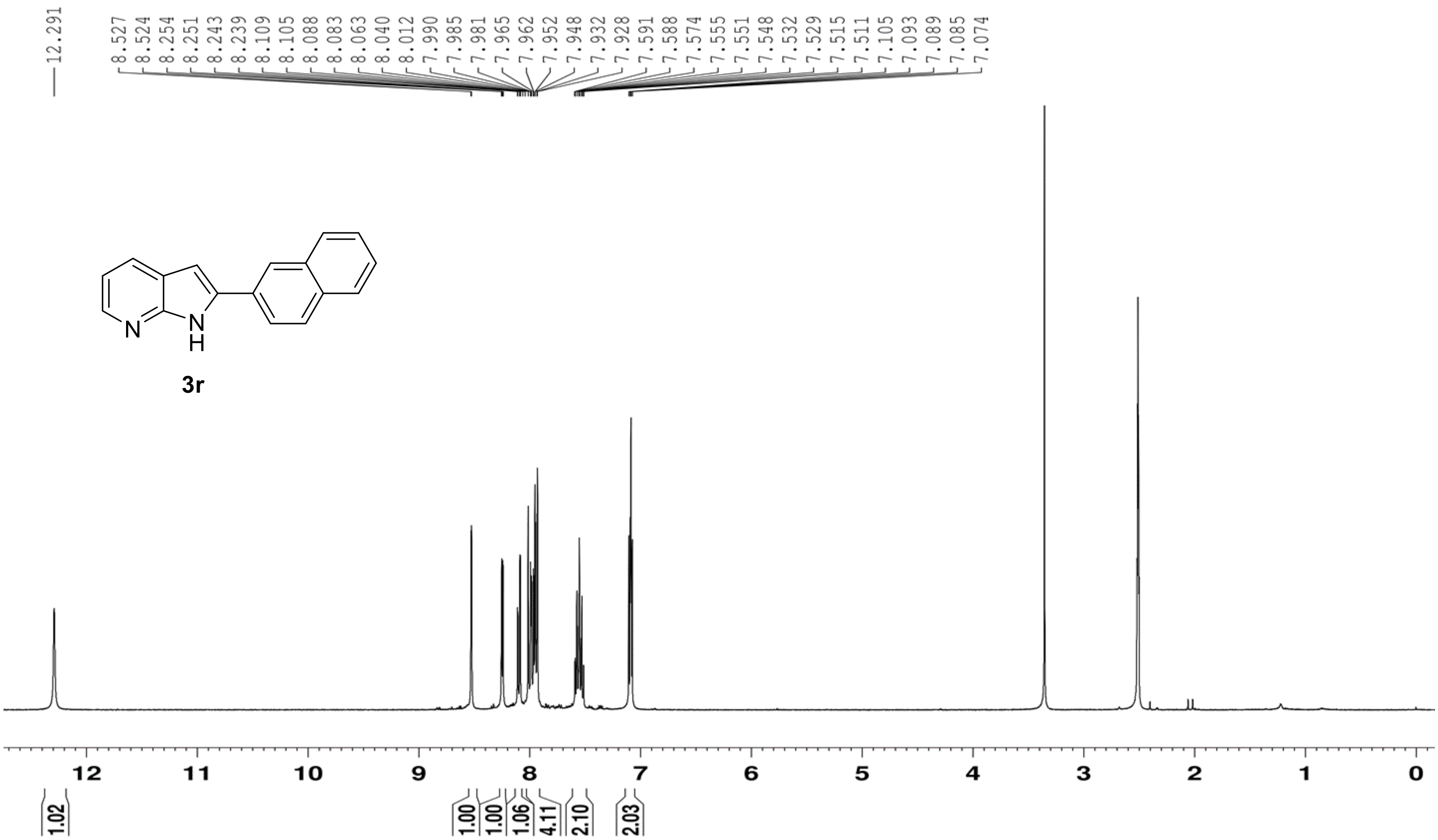

Supplementary Figure 83. ^{1}H NMR spectrum of **3r**.

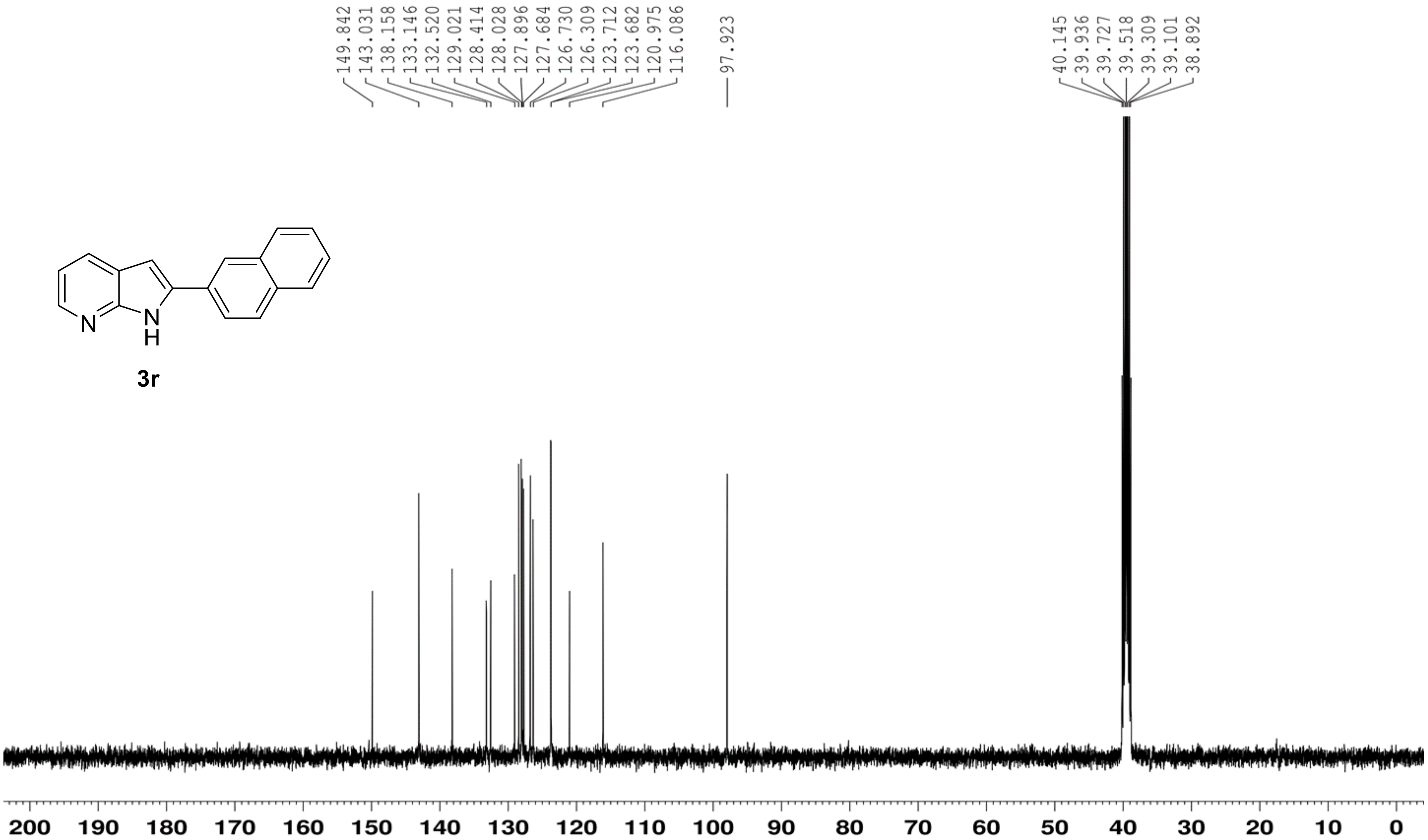

Supplementary Figure 84. ^{13}C NMR spectrum of **3r**.

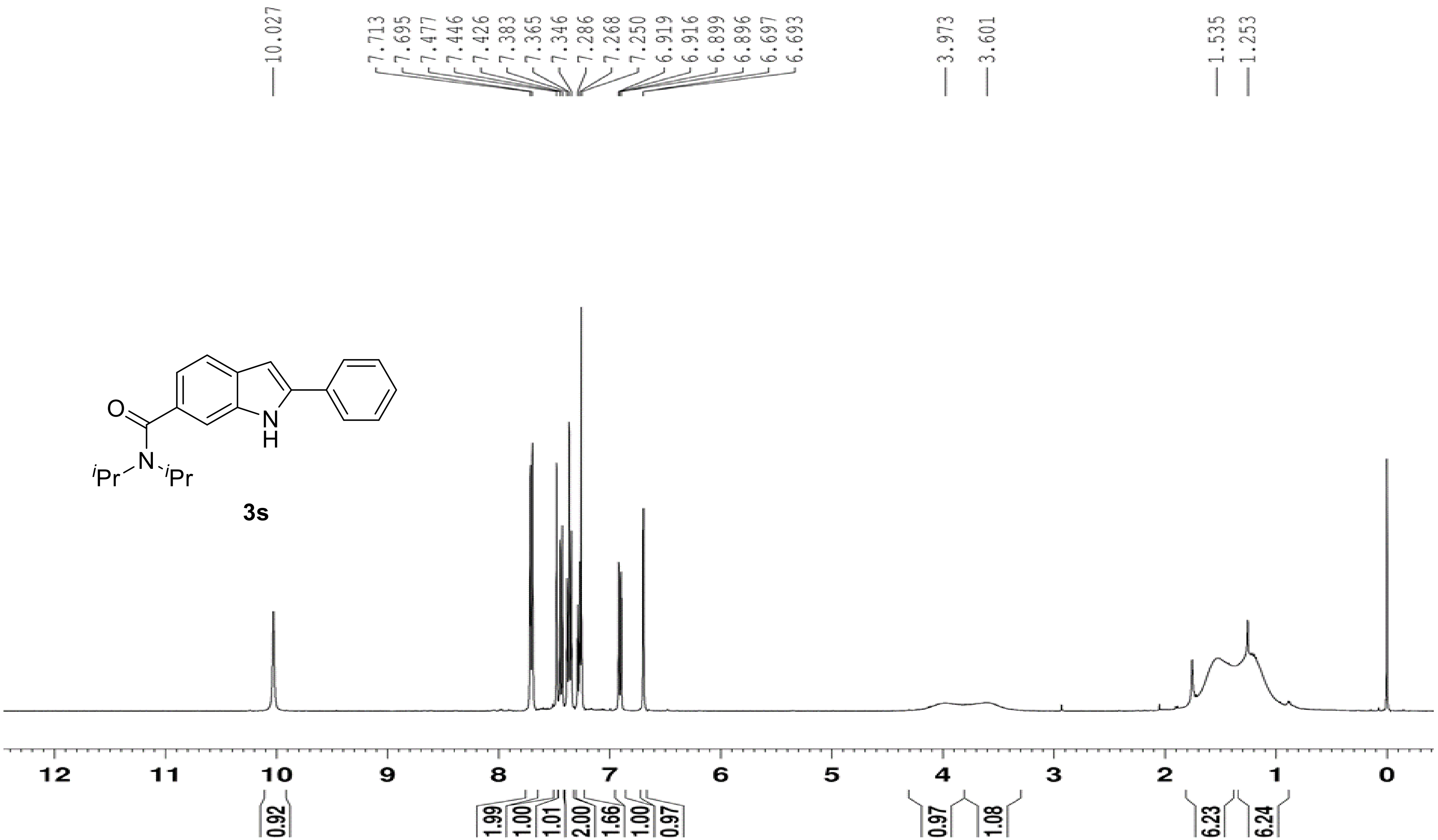

Supplementary Figure 85. ^{1}H NMR spectrum of **3s**.

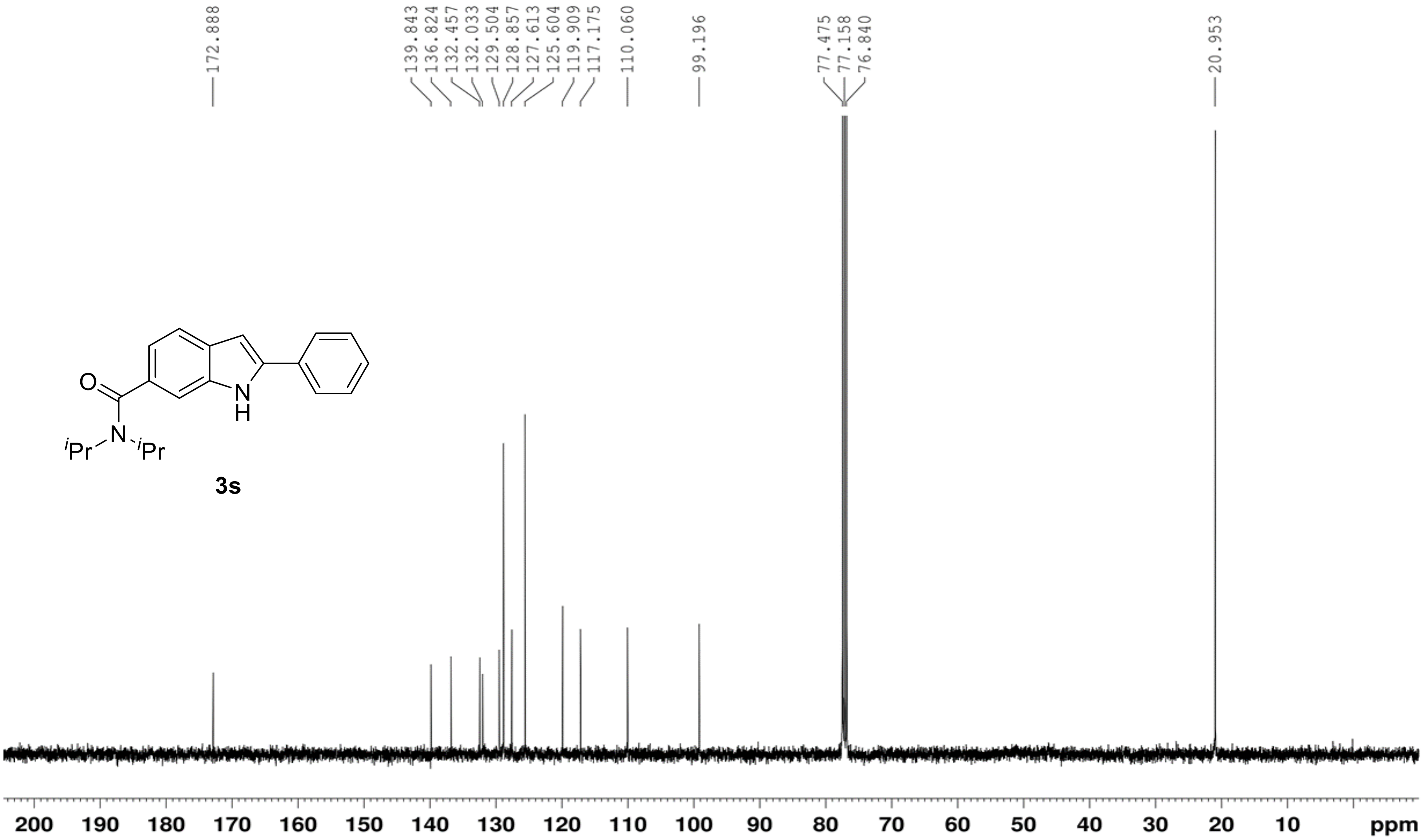

Supplementary Figure 86. ^{13}C NMR spectrum of **3s**.

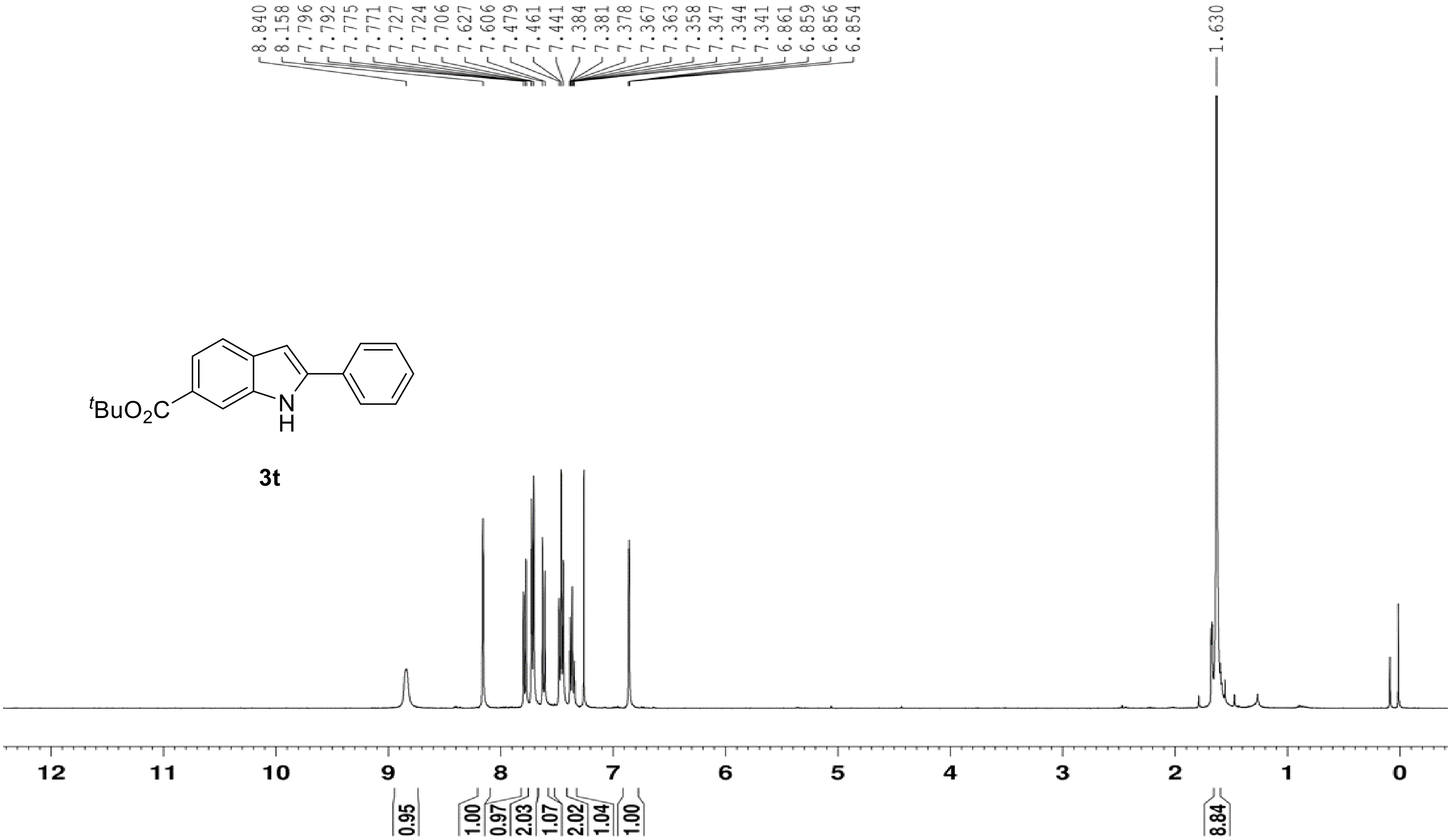

Supplementary Figure 87. ^{1}H NMR spectrum of **3t**.

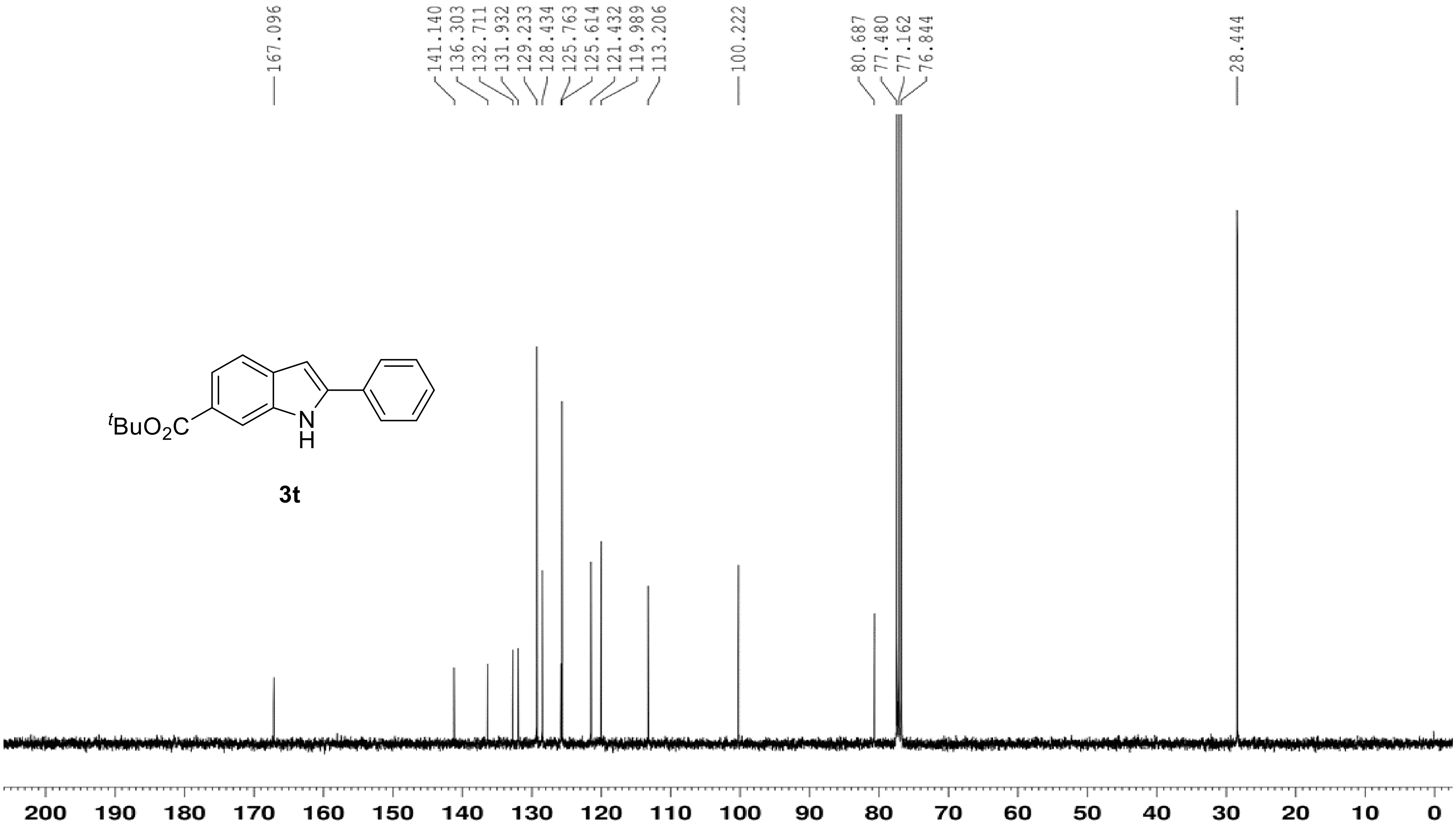

Supplementary Figure 88. ^{13}C NMR spectrum of **3t**.

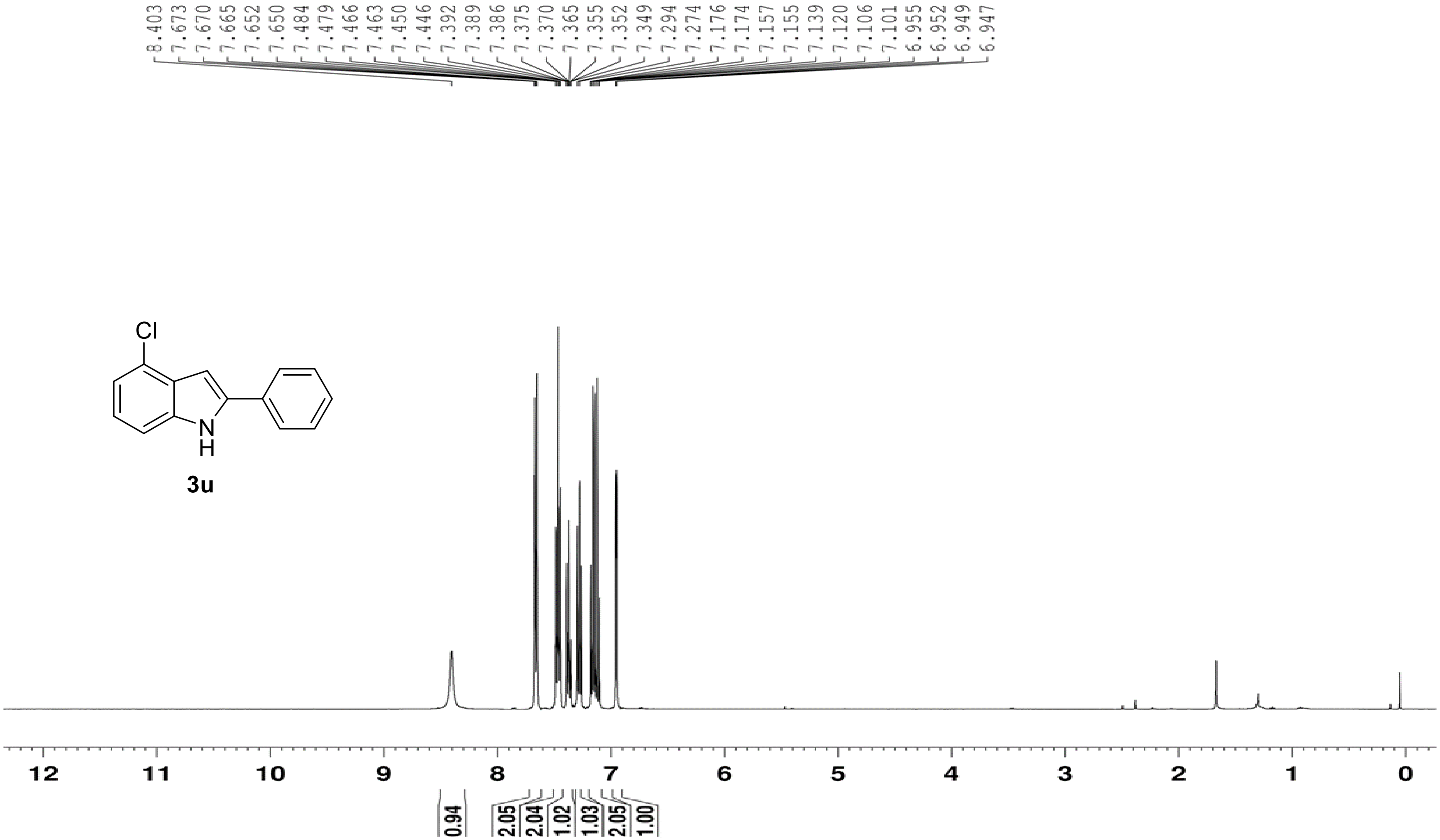

Supplementary Figure 89. ^{1}H NMR spectrum of **3u**.

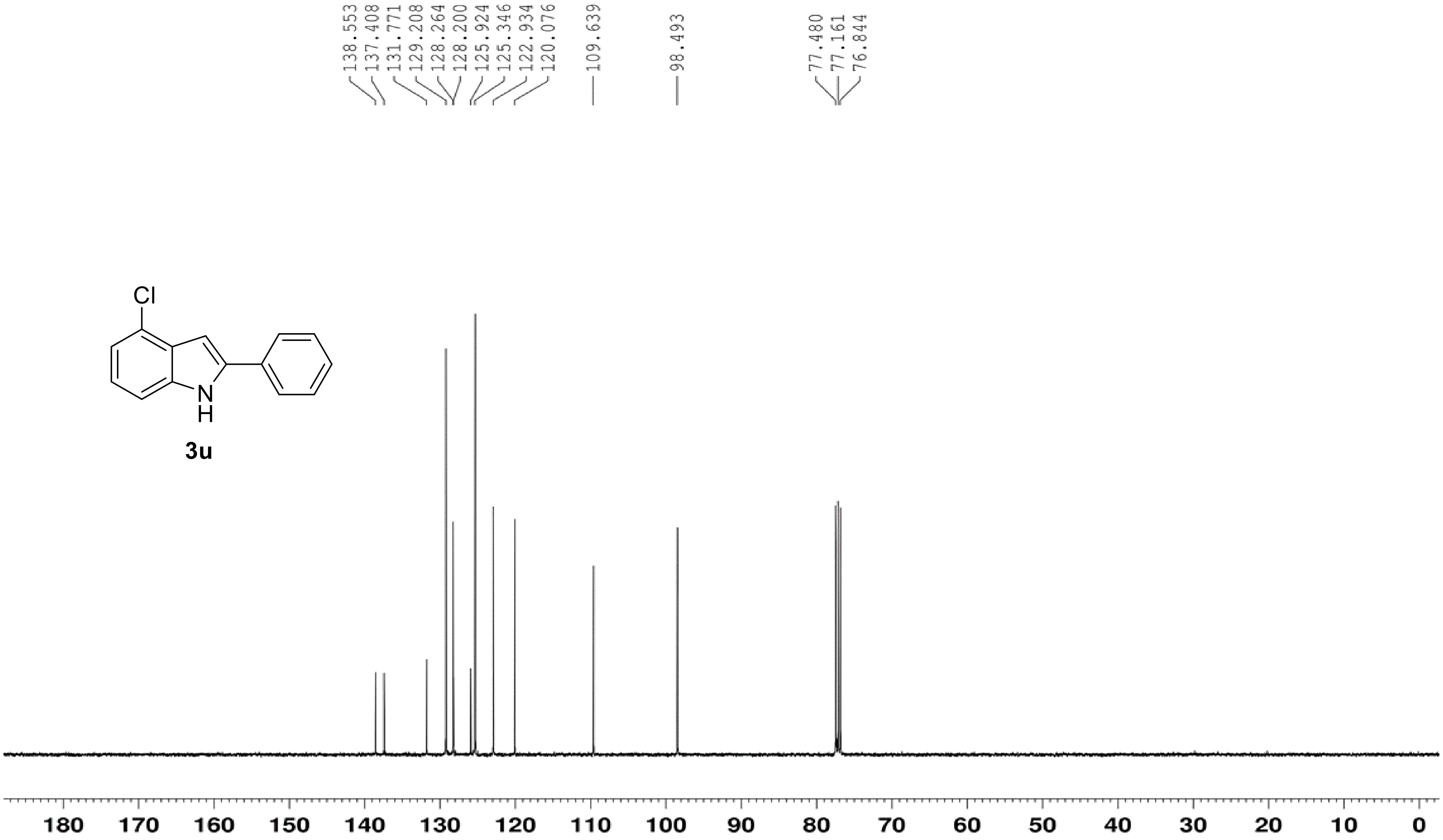

Supplementary Figure 90. ^{13}C NMR spectrum of **3u**.

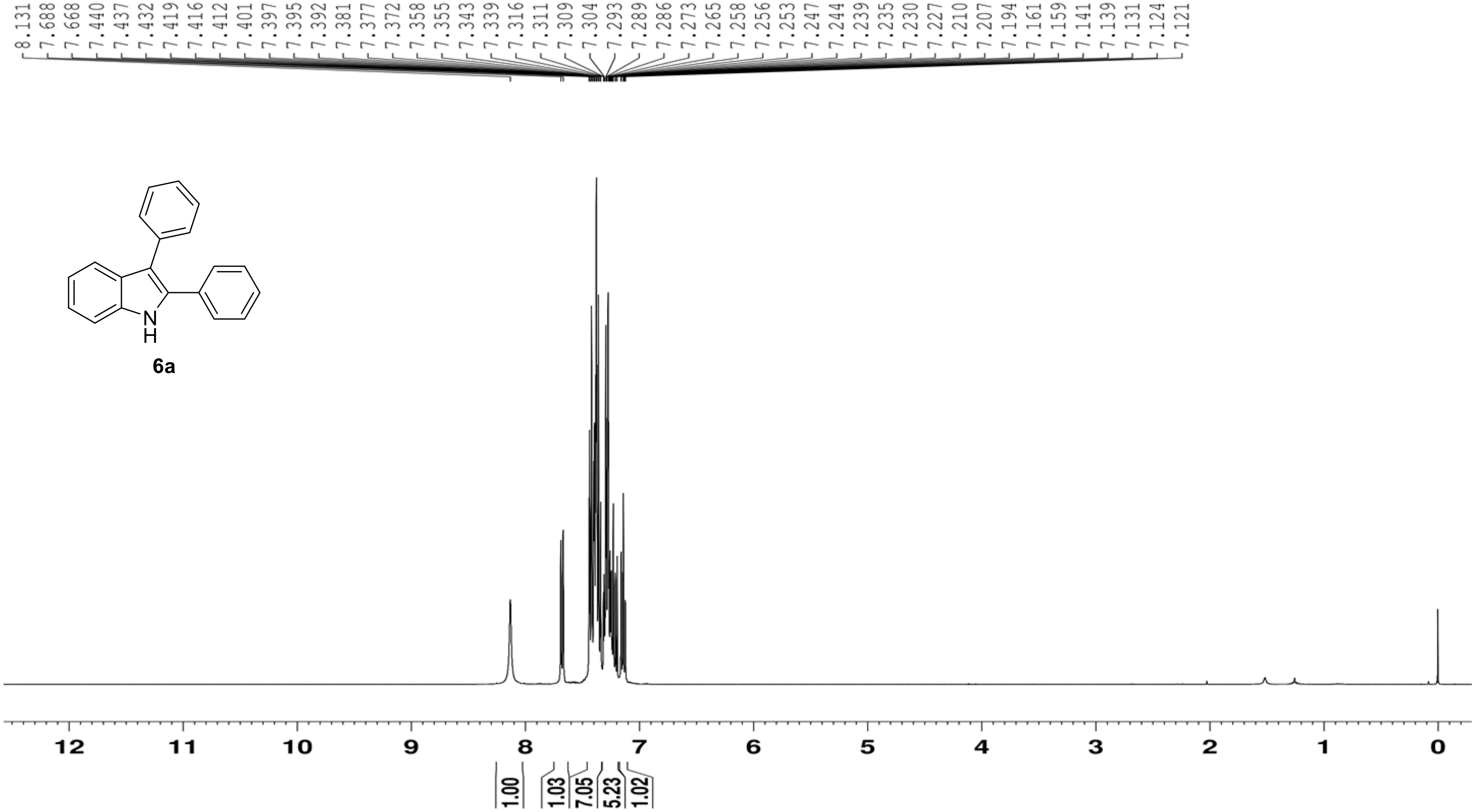

Supplementary Figure 91. ^{1}H NMR spectrum of **6a**.

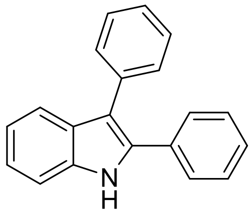

6a

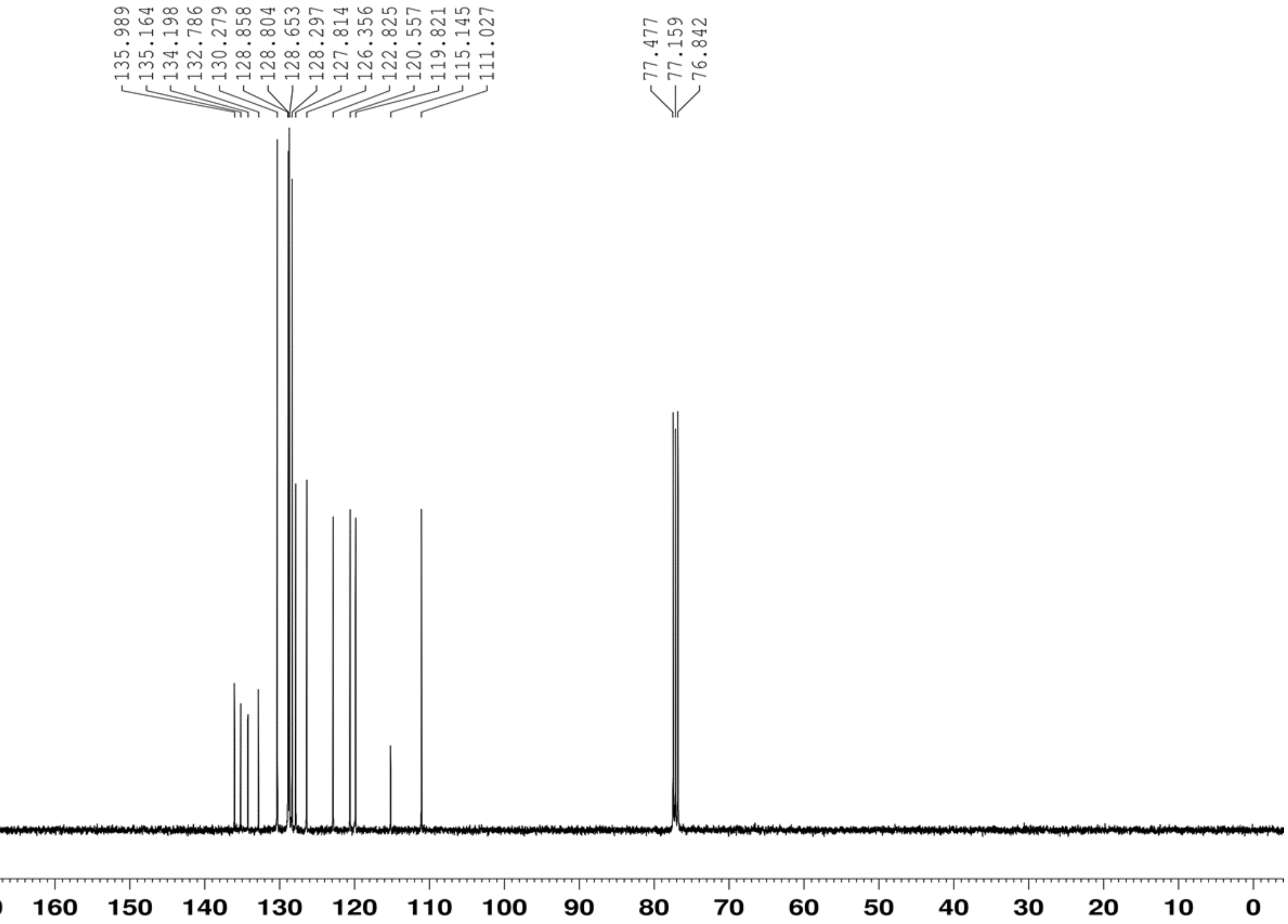

Supplementary Figure 92. ^{13}C NMR spectrum of **6a**.

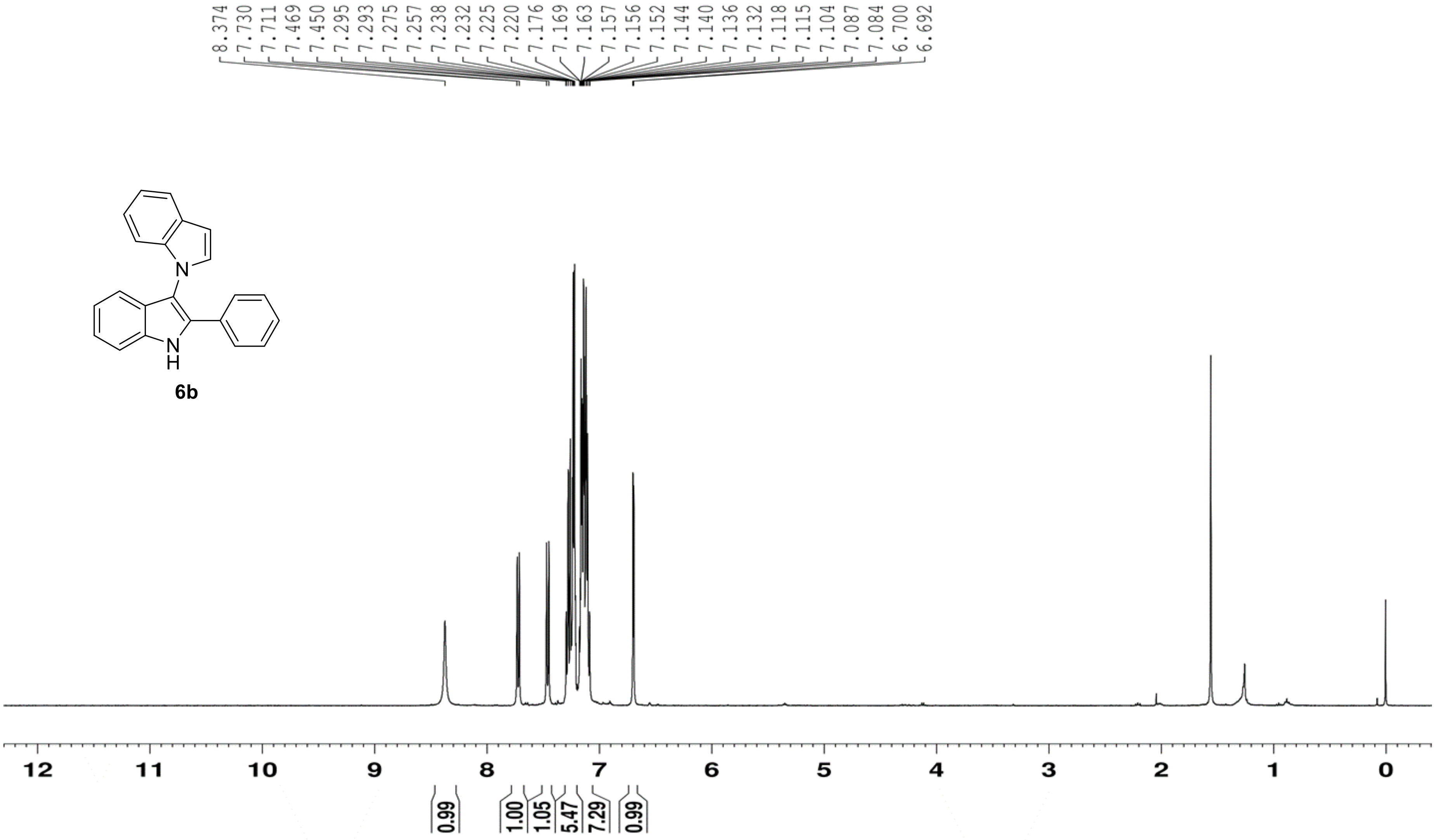

Supplementary Figure 93. ^{1}H NMR spectrum of **6b**.

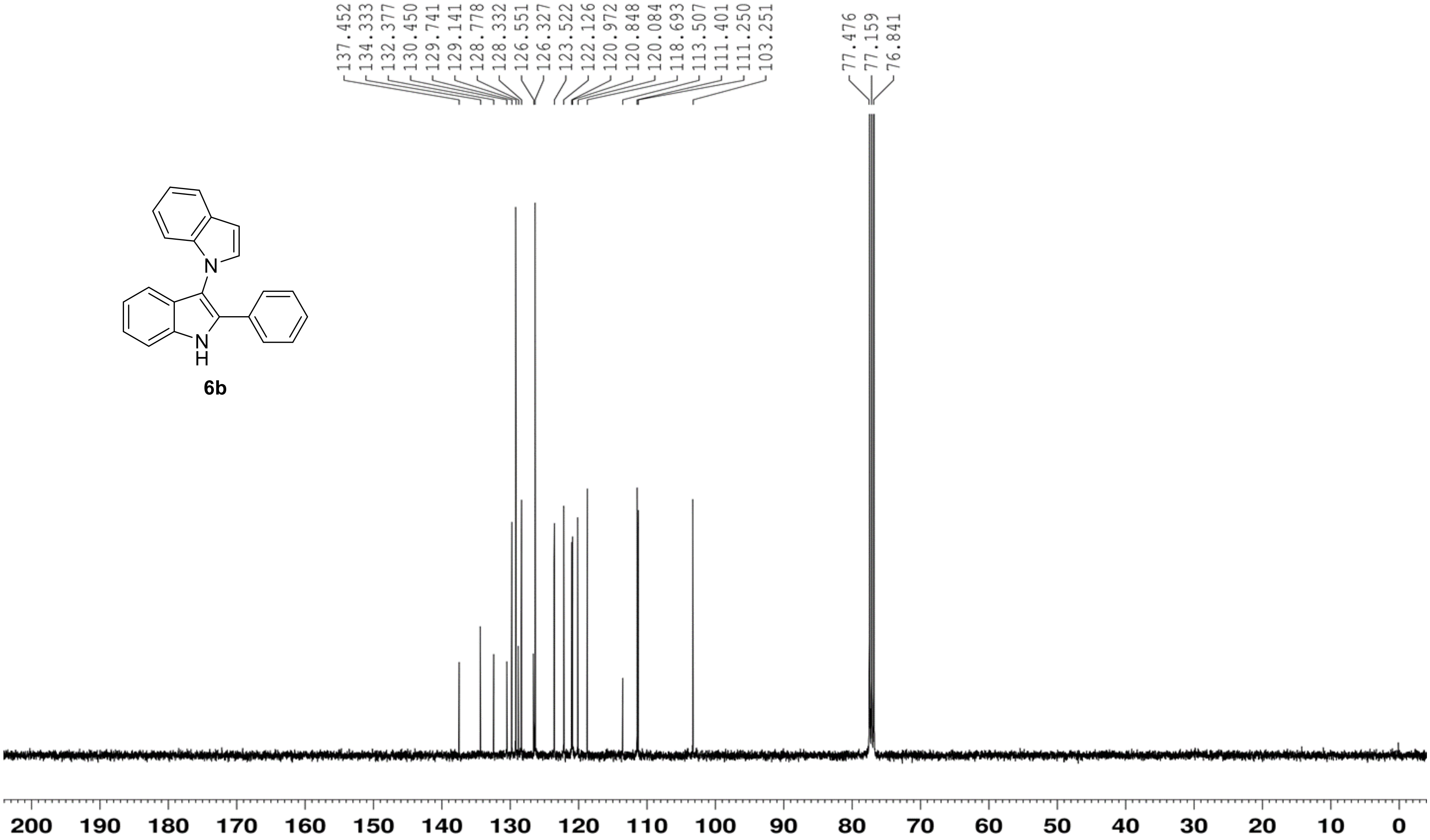

Supplementary Figure 94. ^{13}C NMR spectrum of **6b**.

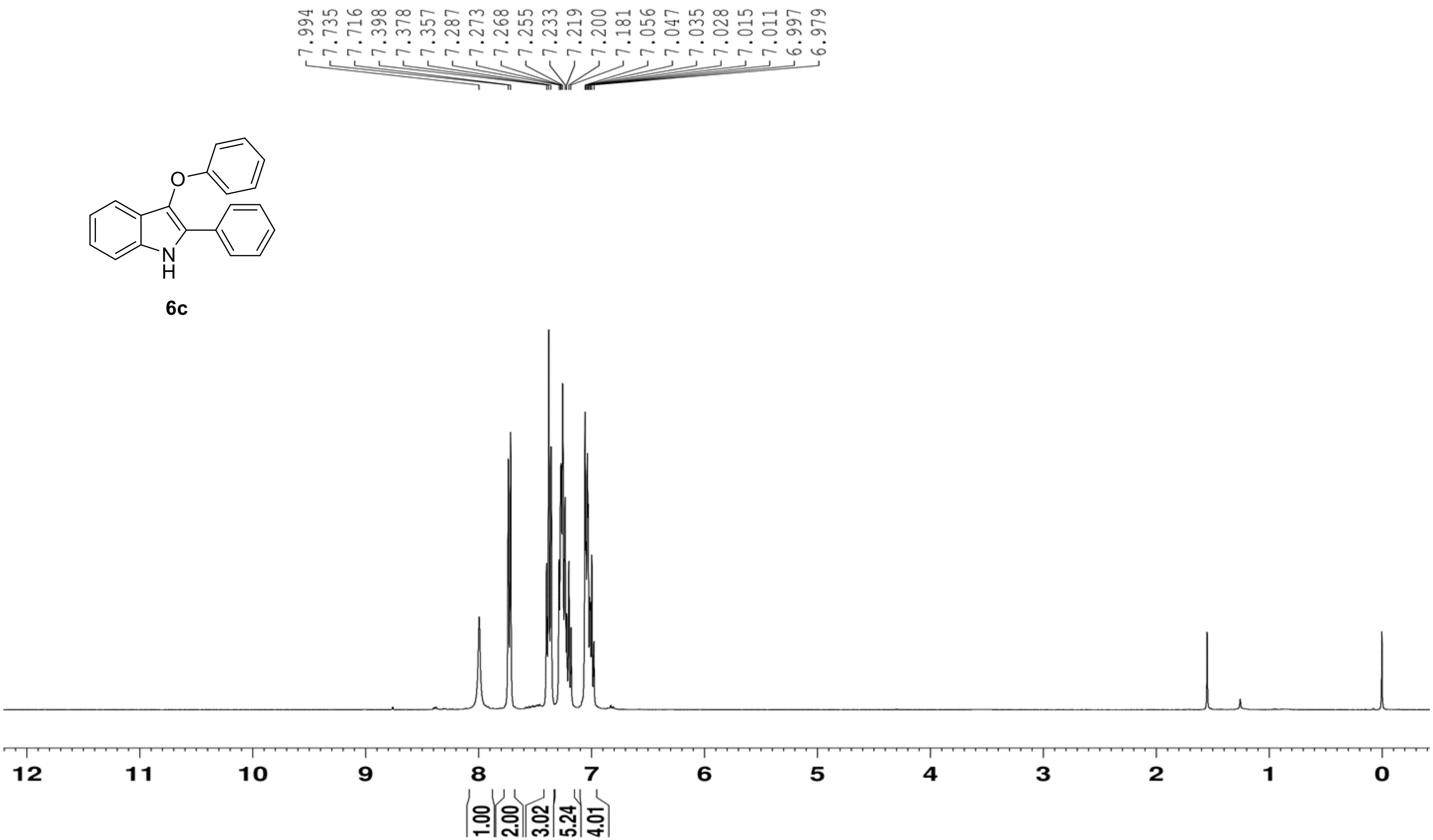

Supplementary Figure 95. ^{1}H NMR spectrum of **6c**.

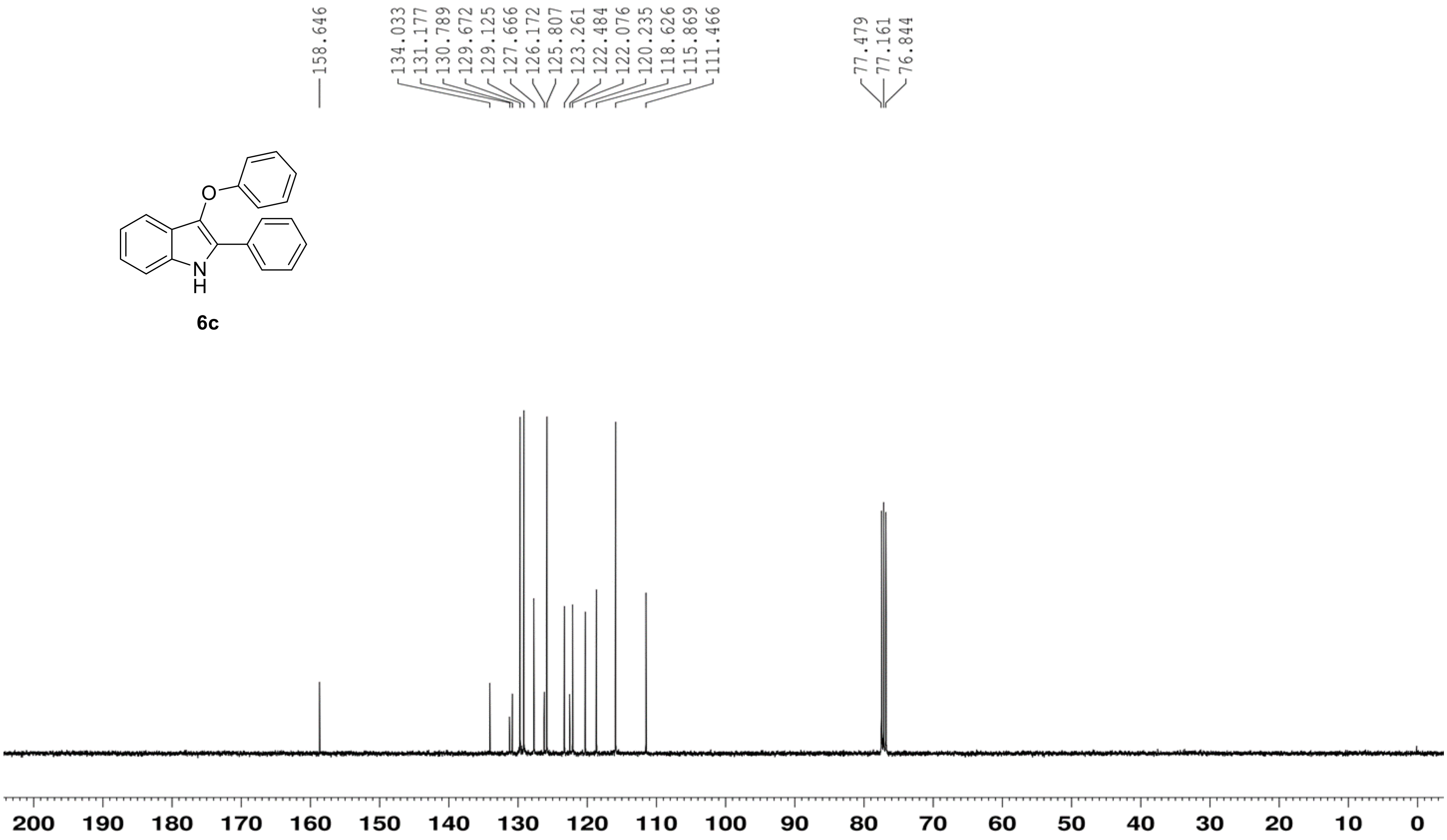

Supplementary Figure 96. ^{13}C NMR spectrum of **6c**.

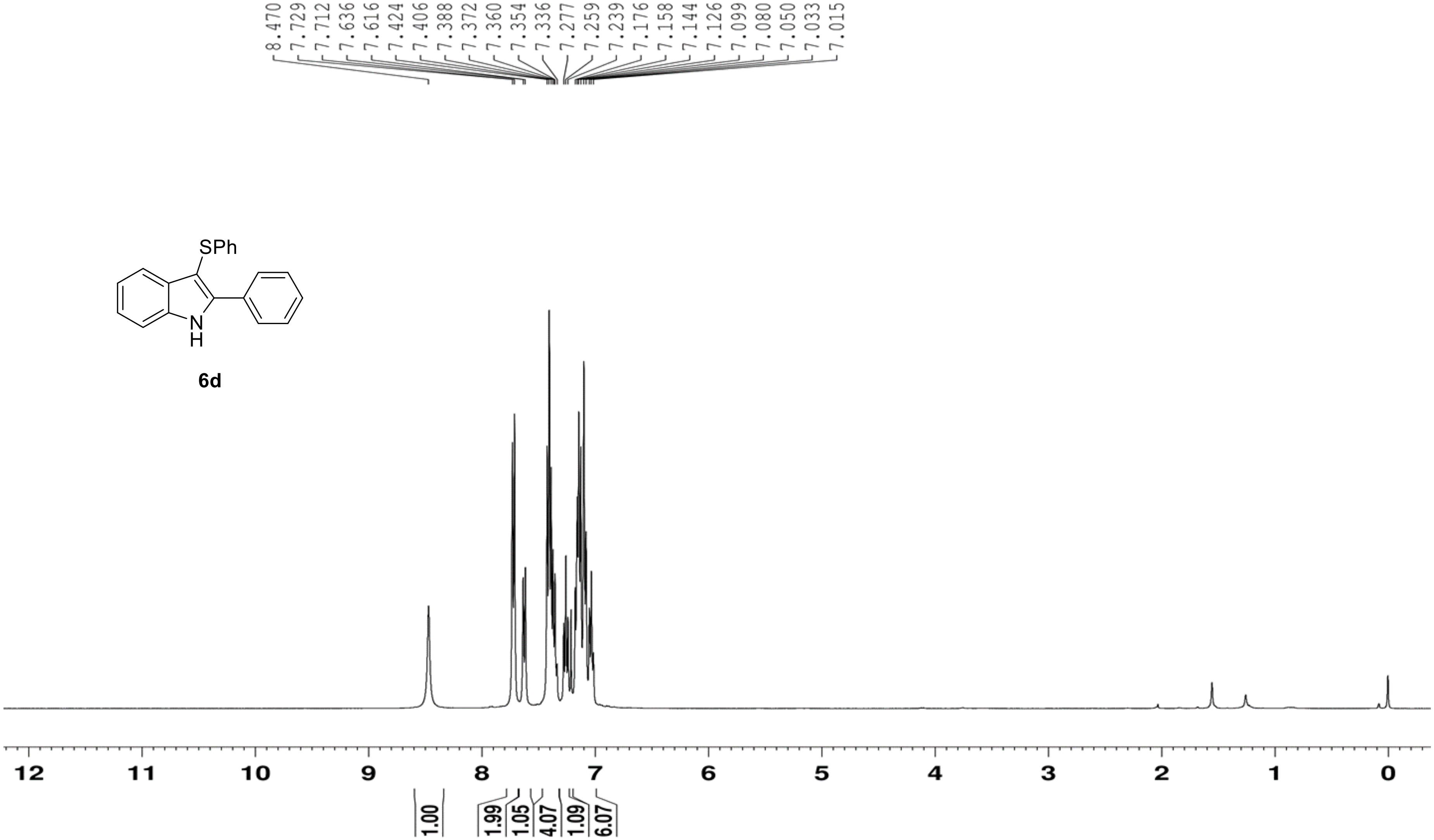

Supplementary Figure 97. ^{1}H NMR spectrum of **6d**.

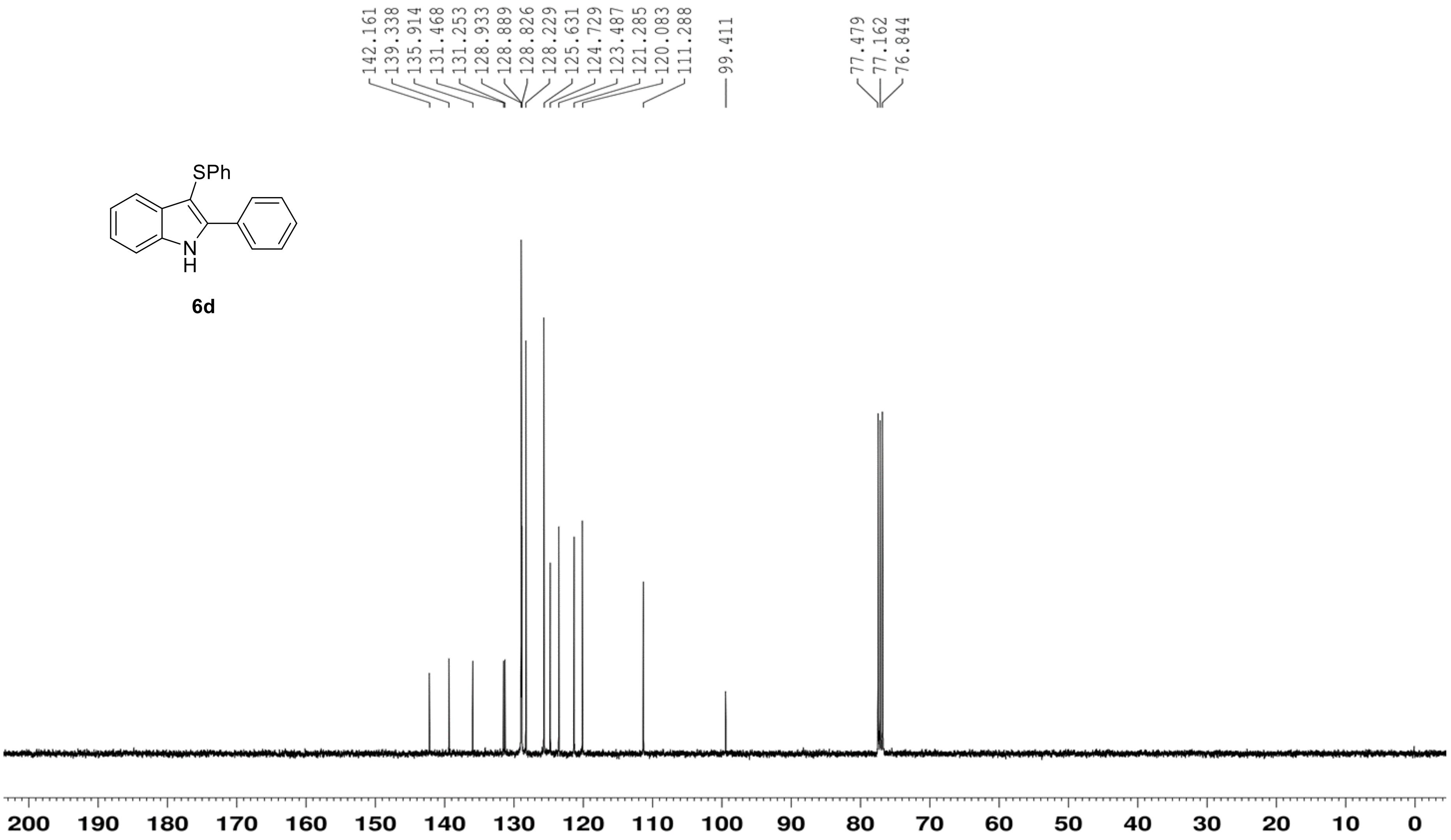

Supplementary Figure 98. ^{13}C NMR spectrum of **6d**.

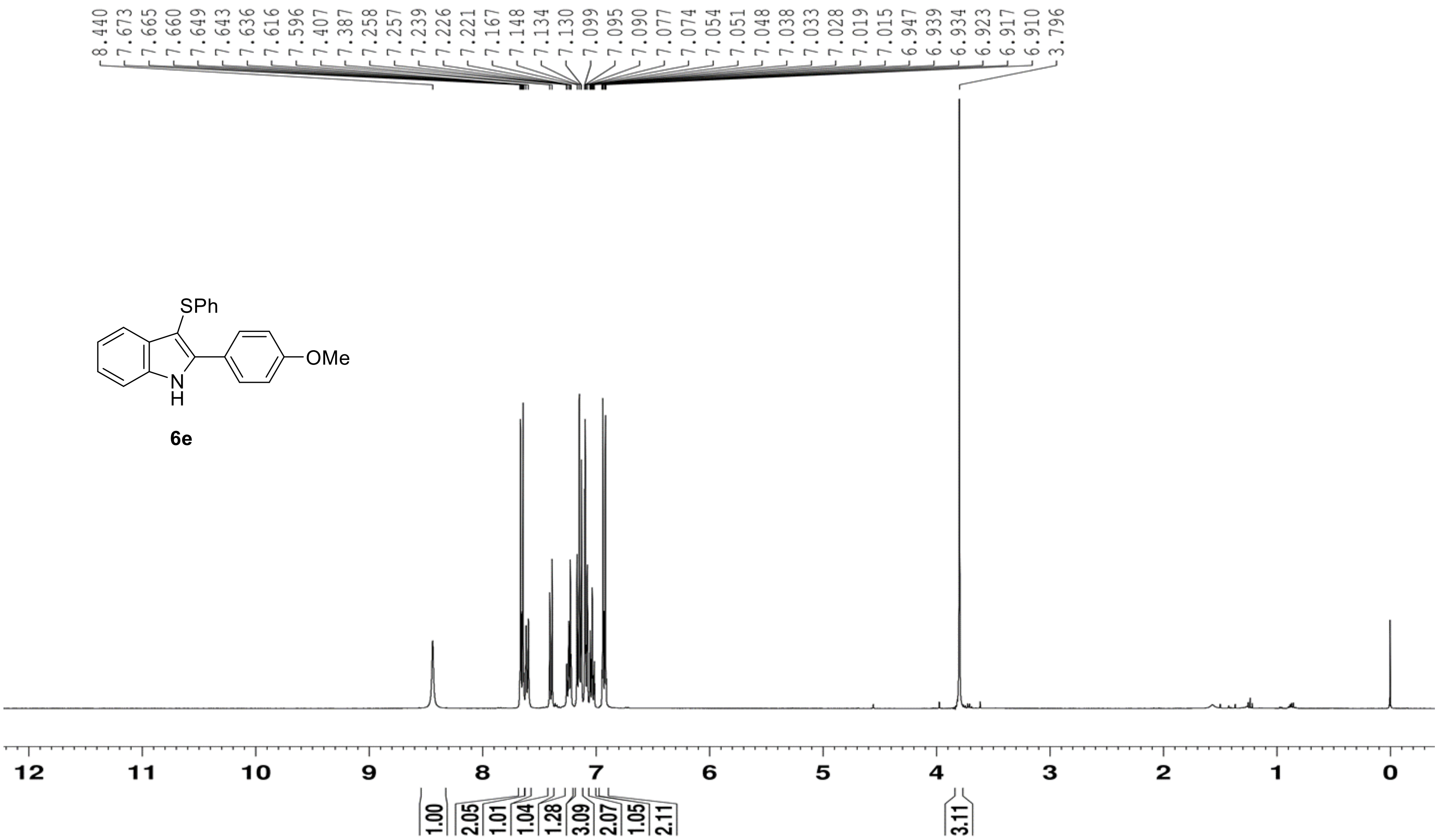

Supplementary Figure 99. ^{1}H NMR spectrum of **6e**.

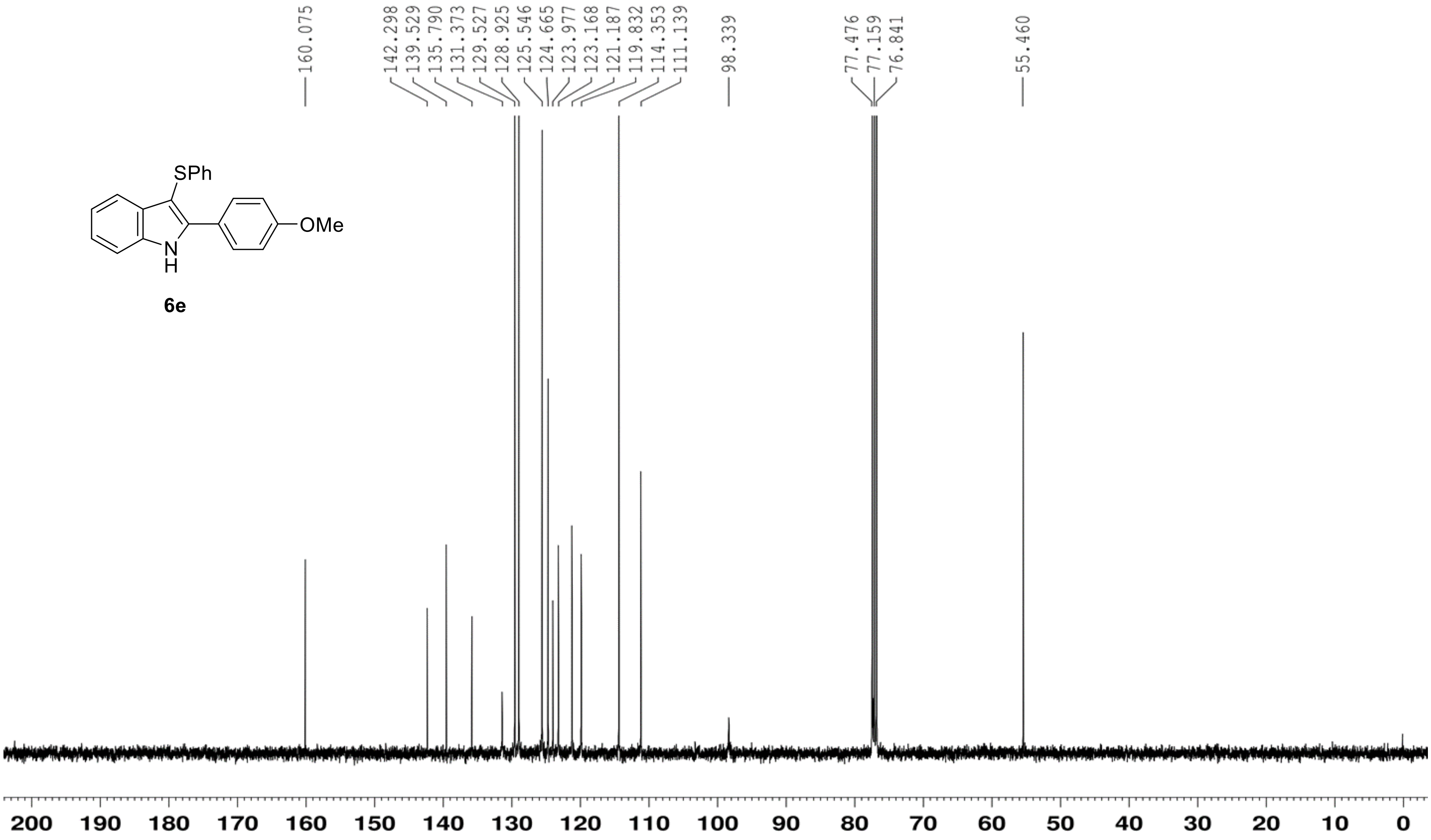

Supplementary Figure 100. ^{13}C NMR spectrum of **6e**.

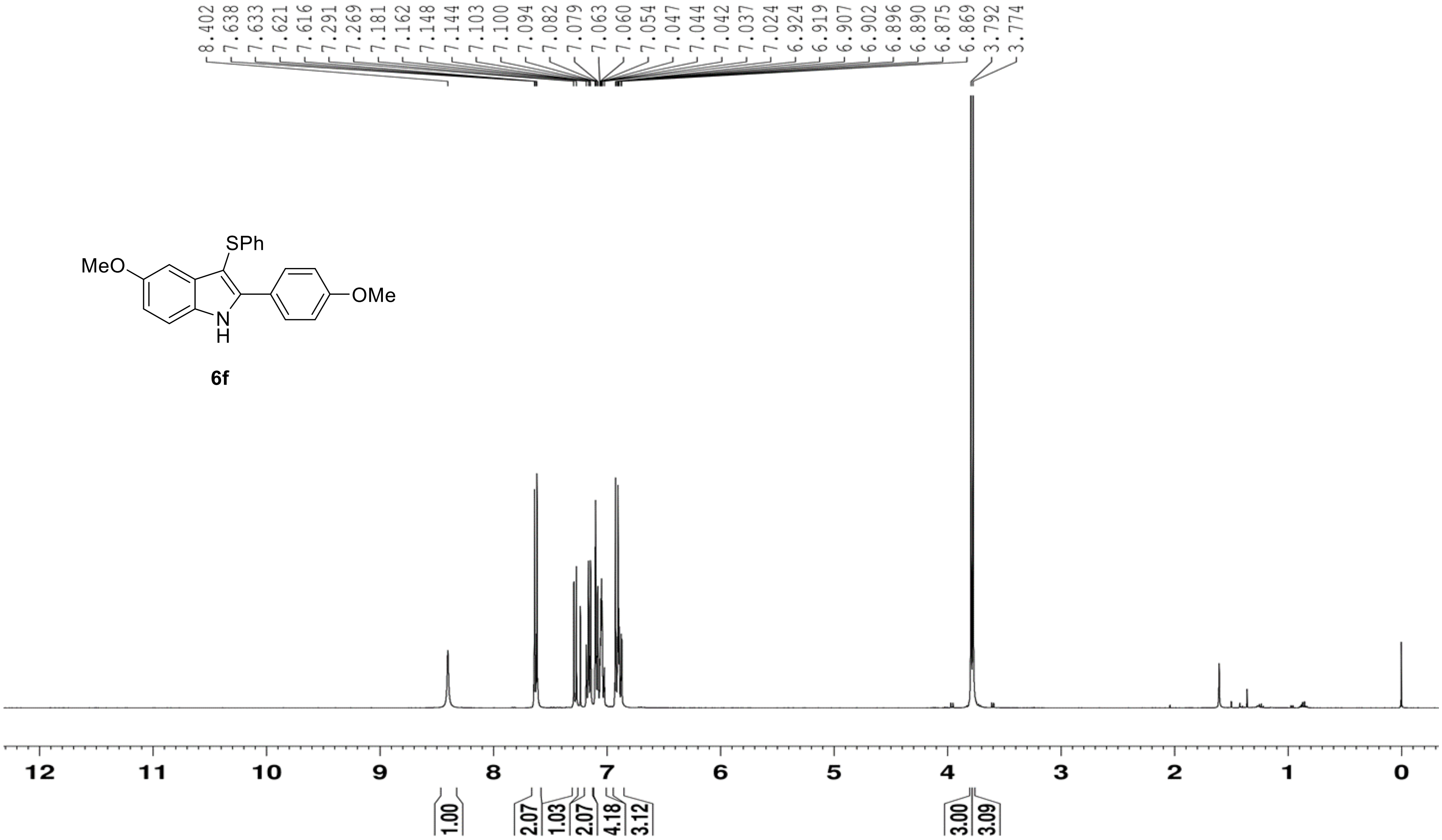

Supplementary Figure 101. ^{1}H NMR spectrum of **6f**.

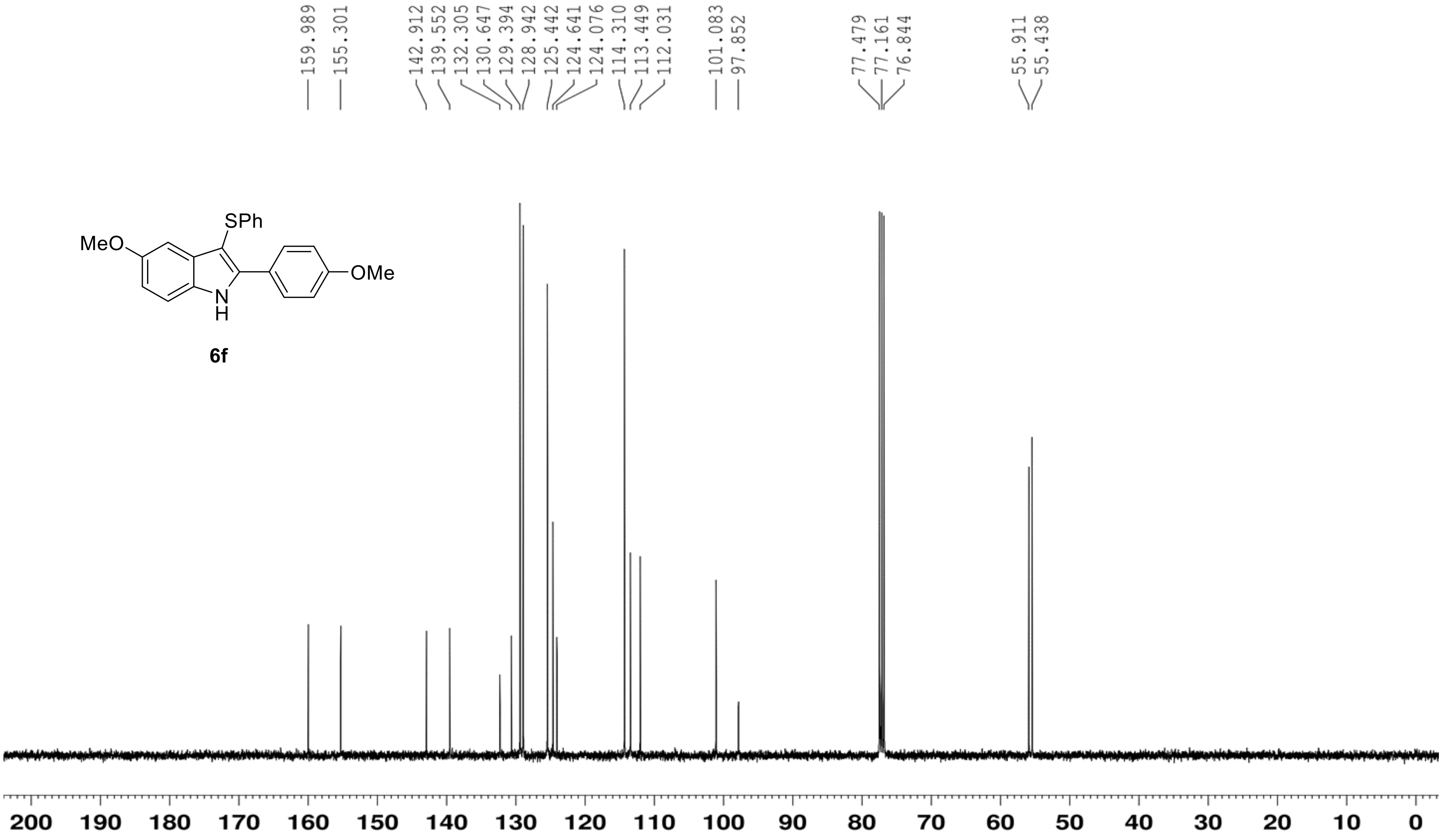

Supplementary Figure 102. ^{13}C NMR spectrum of **6f**.

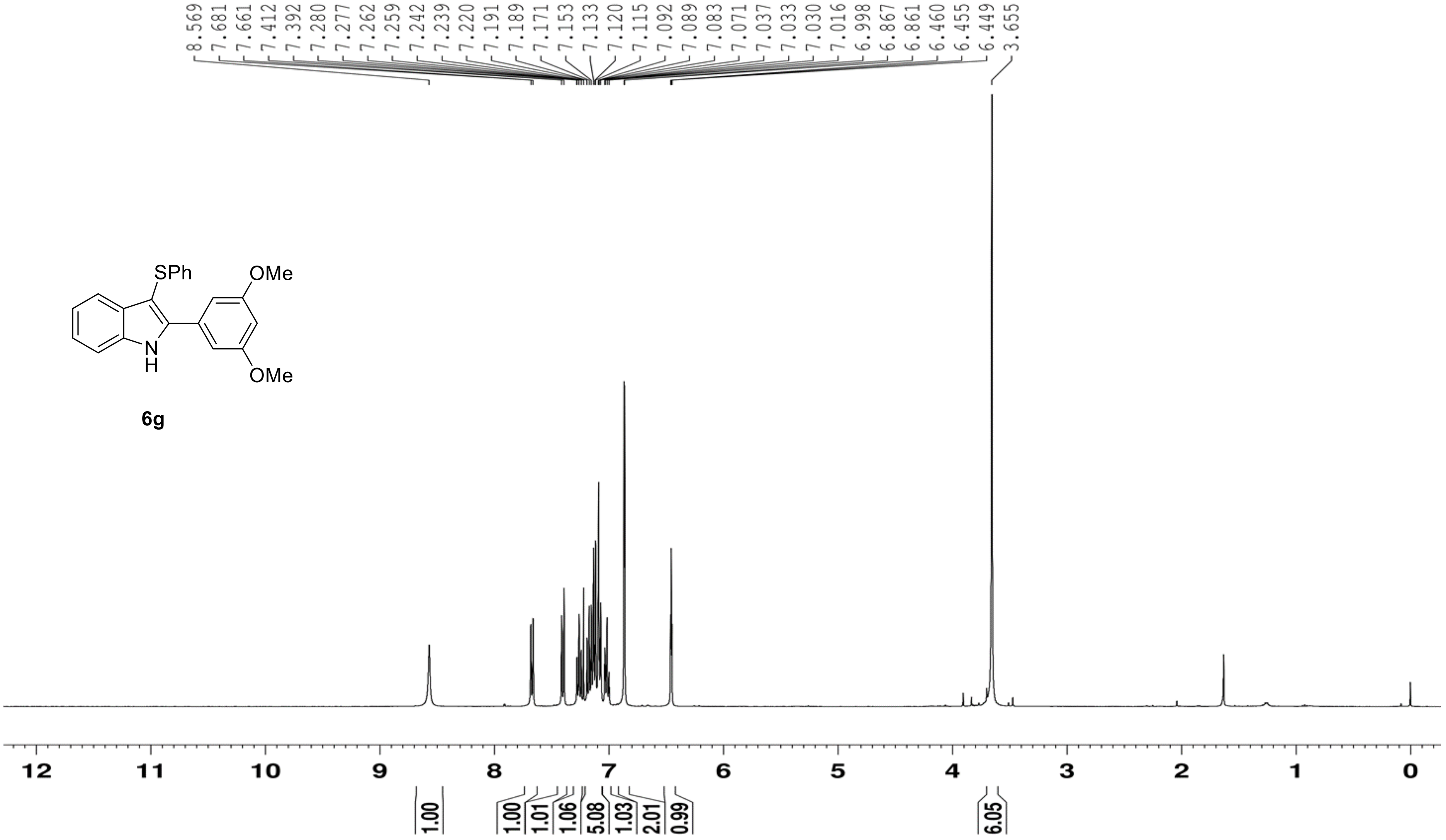

Supplementary Figure 103. ^{1}H NMR spectrum of **6g**.

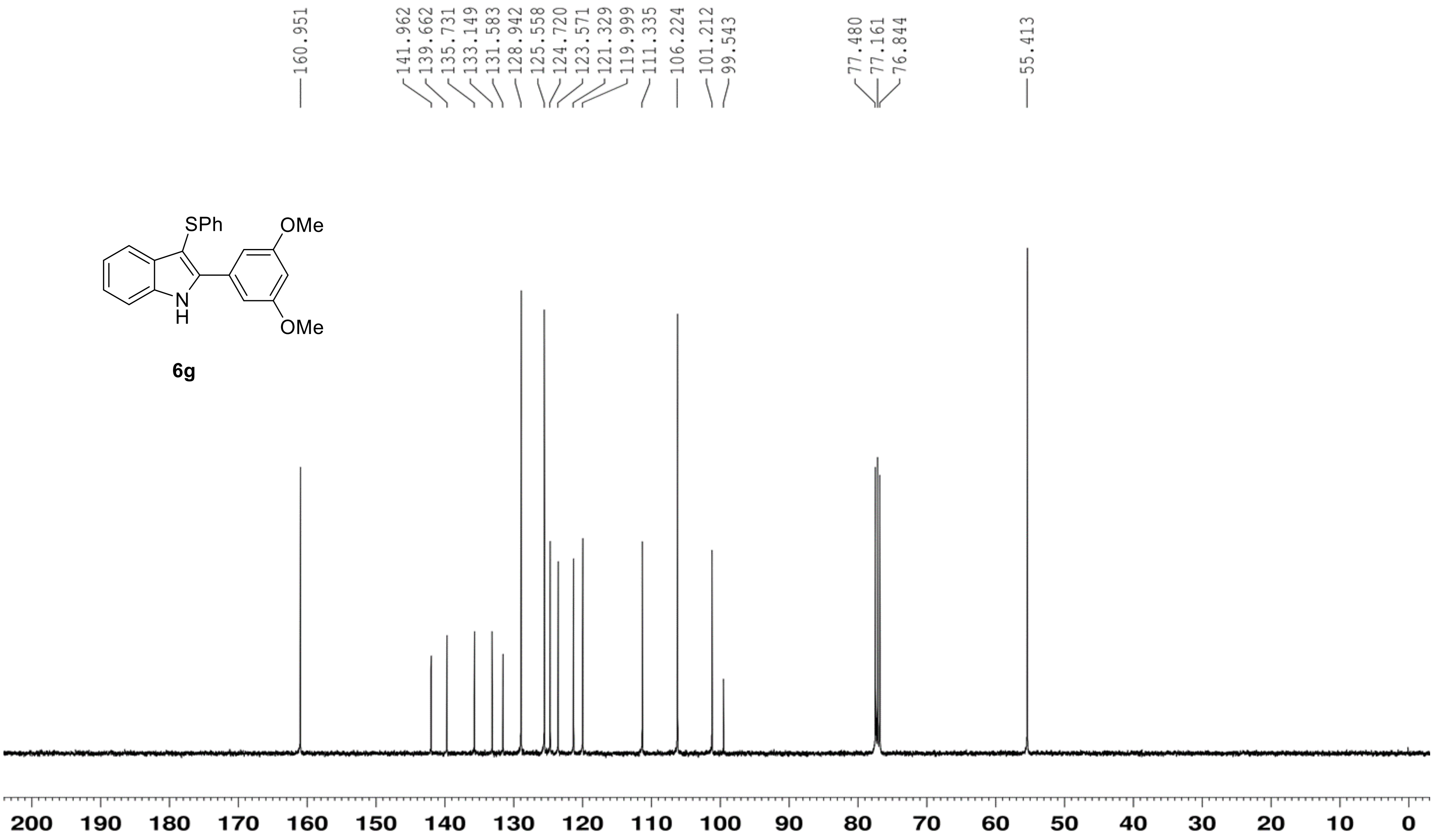

Supplementary Figure 104. ^{13}C NMR spectrum of **6g**.

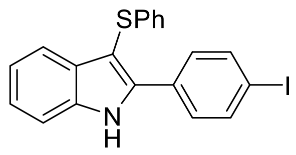

6h

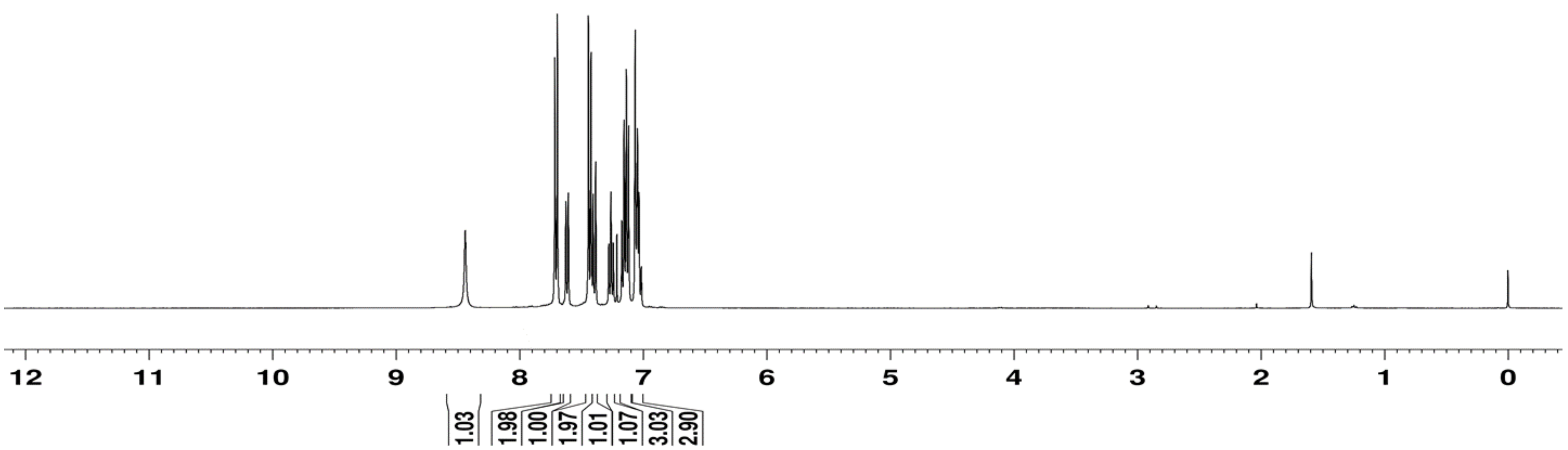

Supplementary Figure 105. ^{1}H NMR spectrum of **6h**.

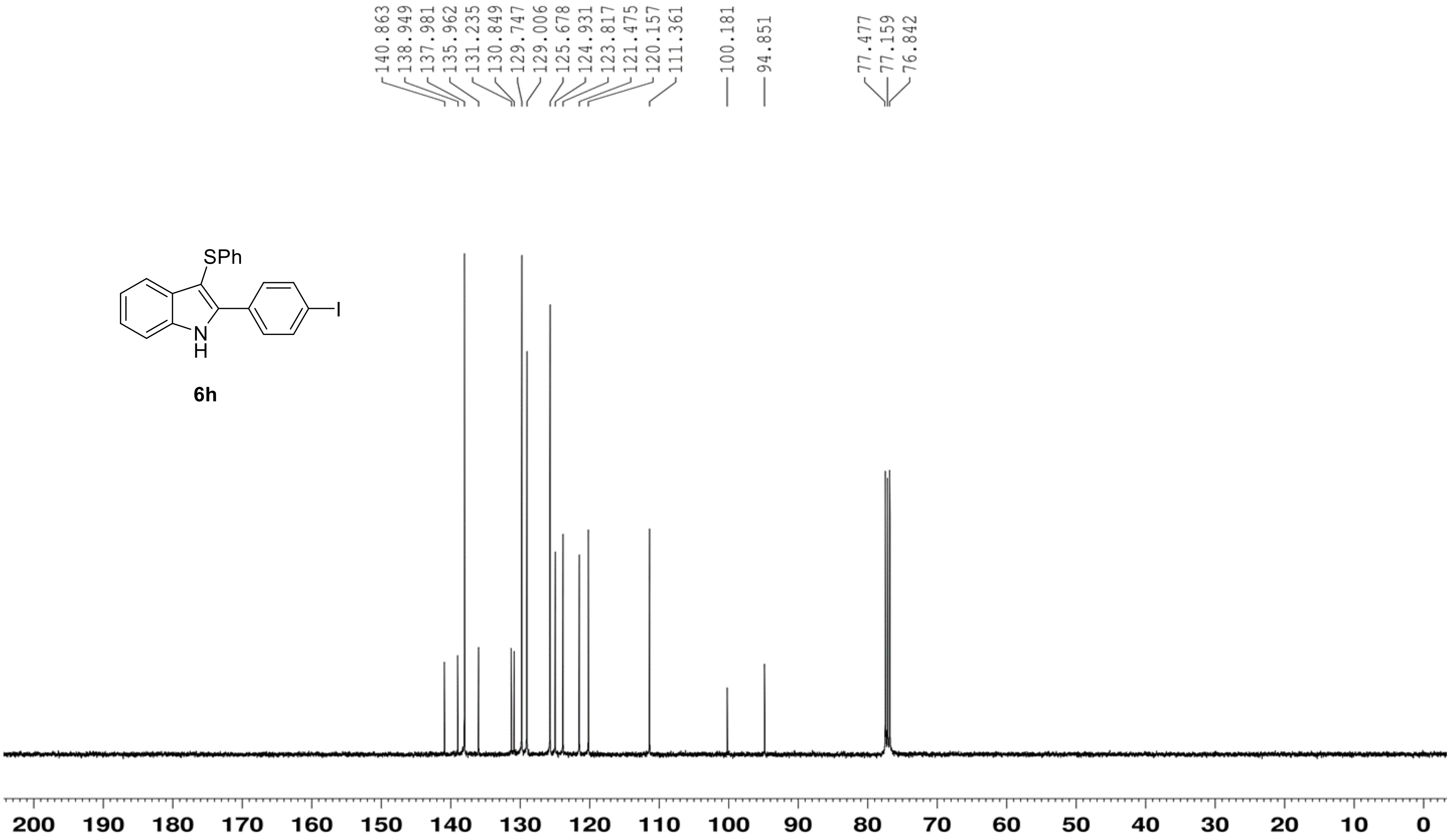

Supplementary Figure 106. ^{13}C NMR spectrum of **6h**.

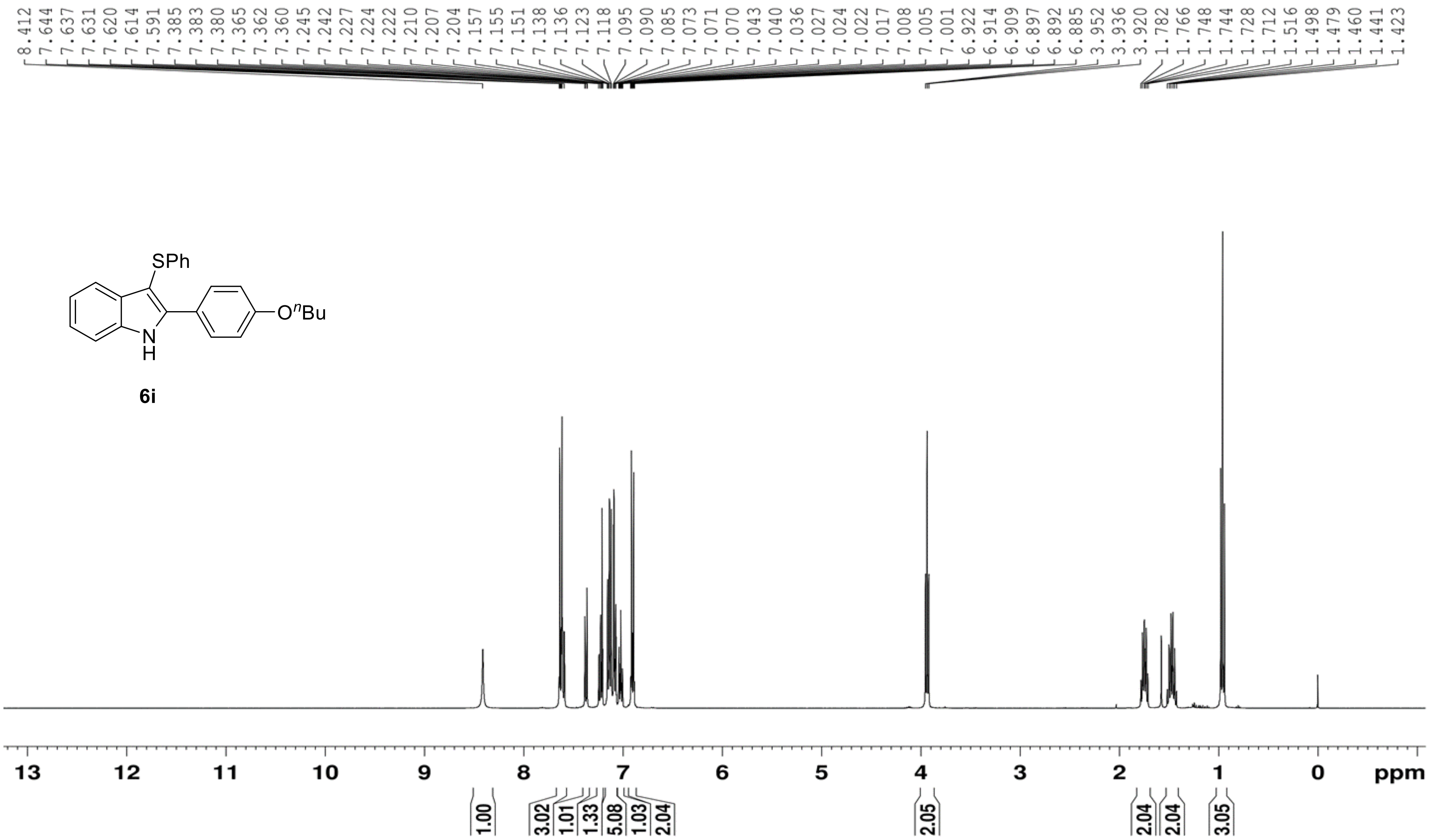

Supplementary Figure 107. ^{1}H NMR spectrum of **6i**.

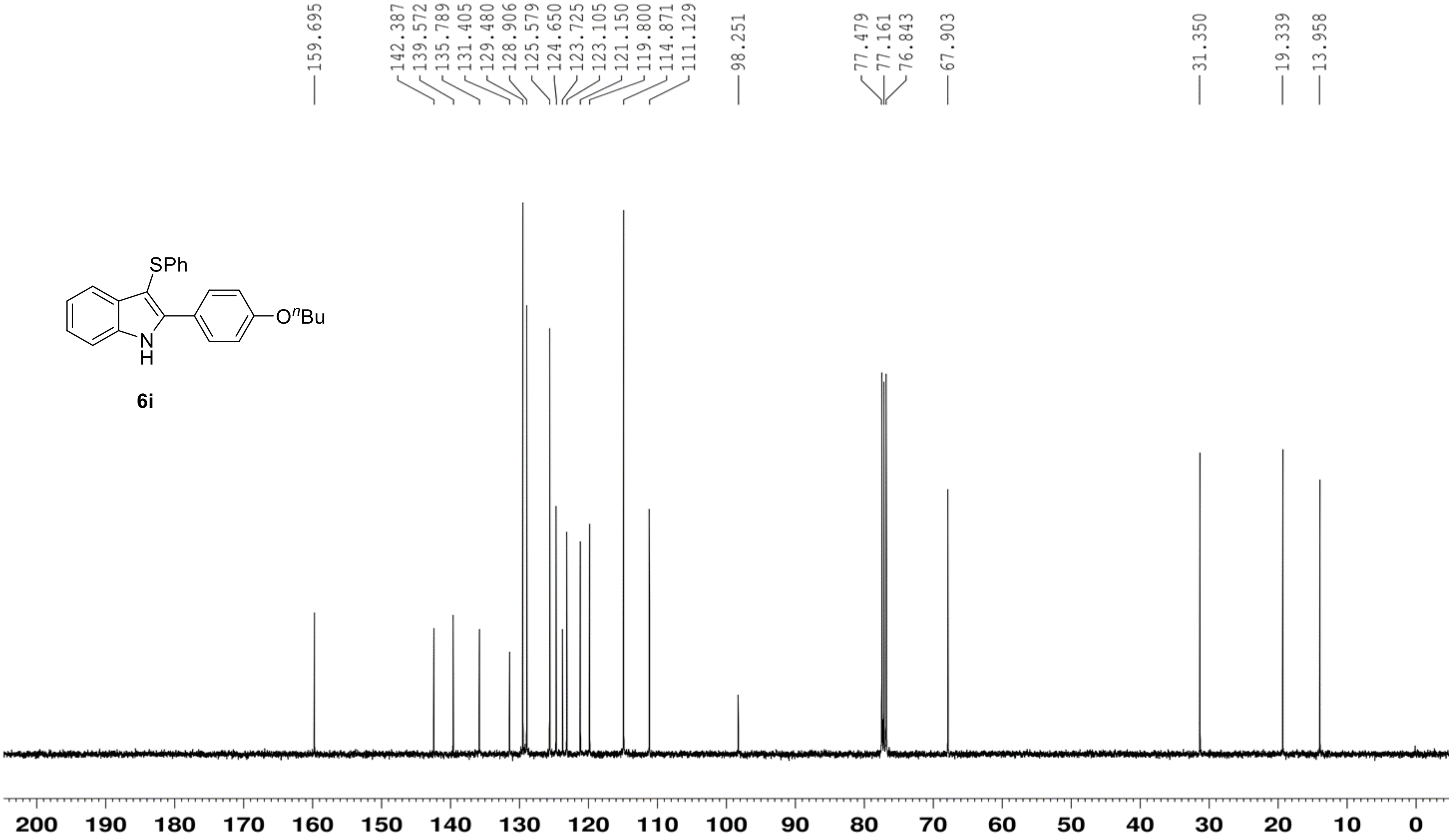

Supplementary Figure 108. ^{13}C NMR spectrum of **6i**.

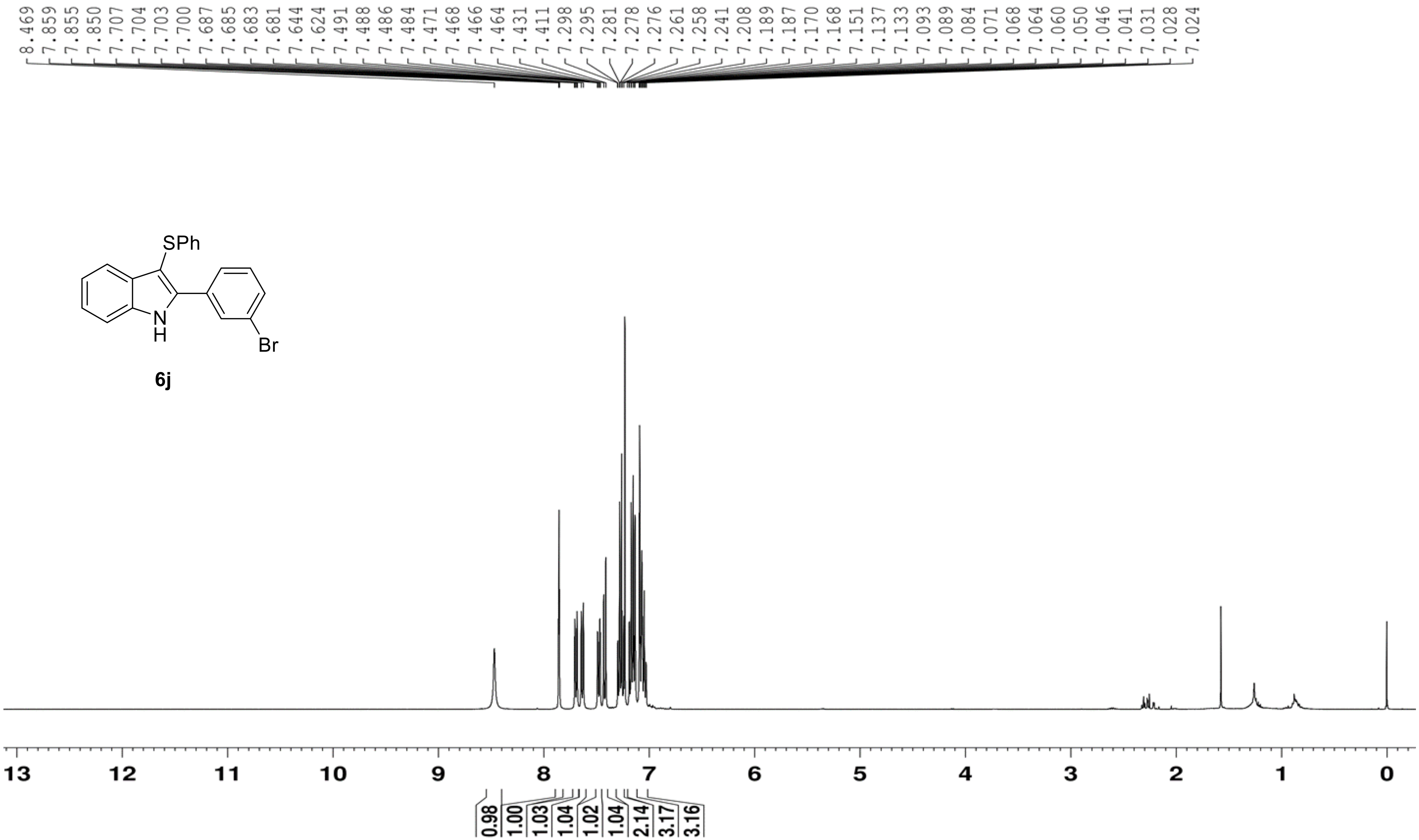

Supplementary Figure 109. ^{1}H NMR spectrum of **6j**.

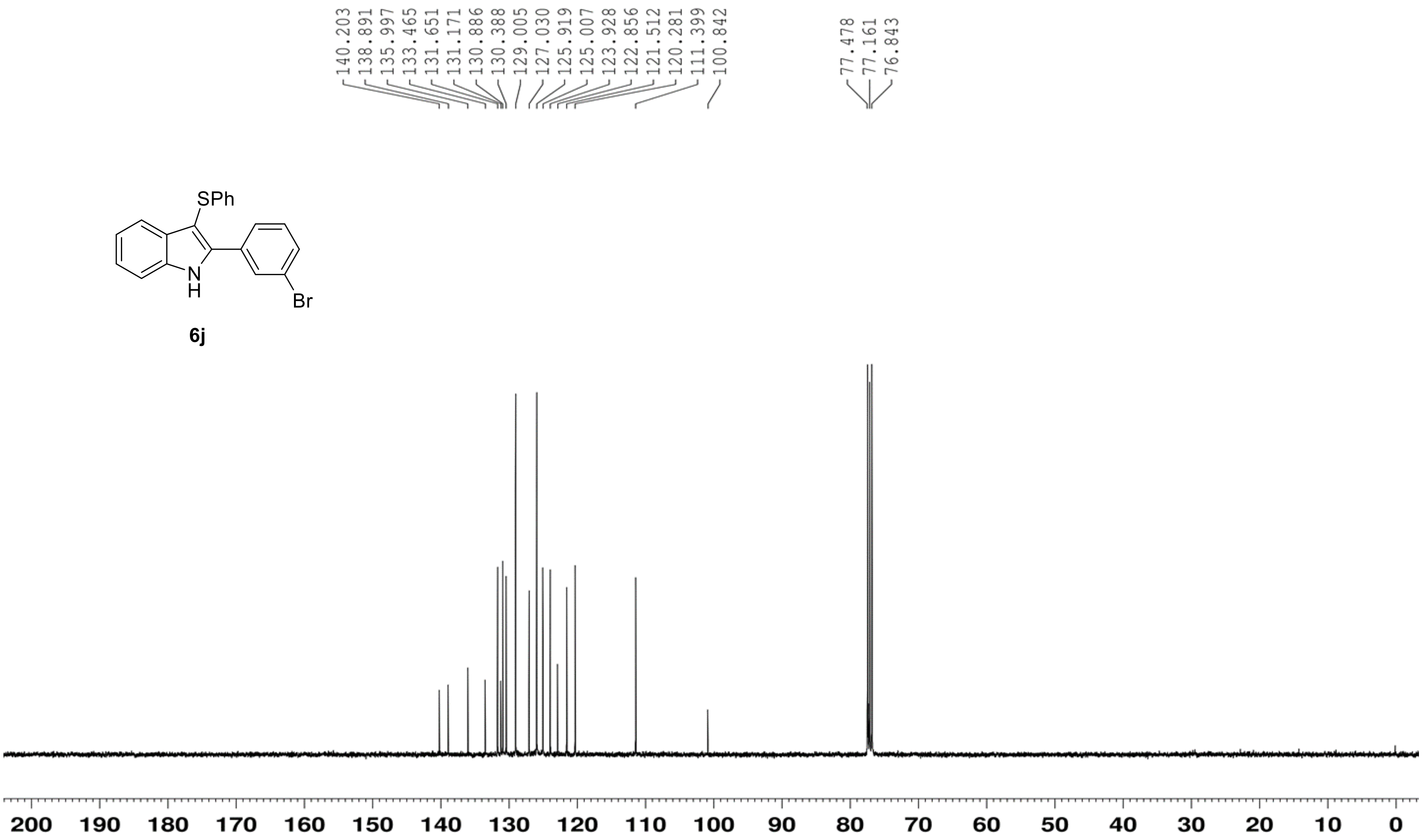

Supplementary Figure 110. ^{13}C NMR spectrum of **6j**.

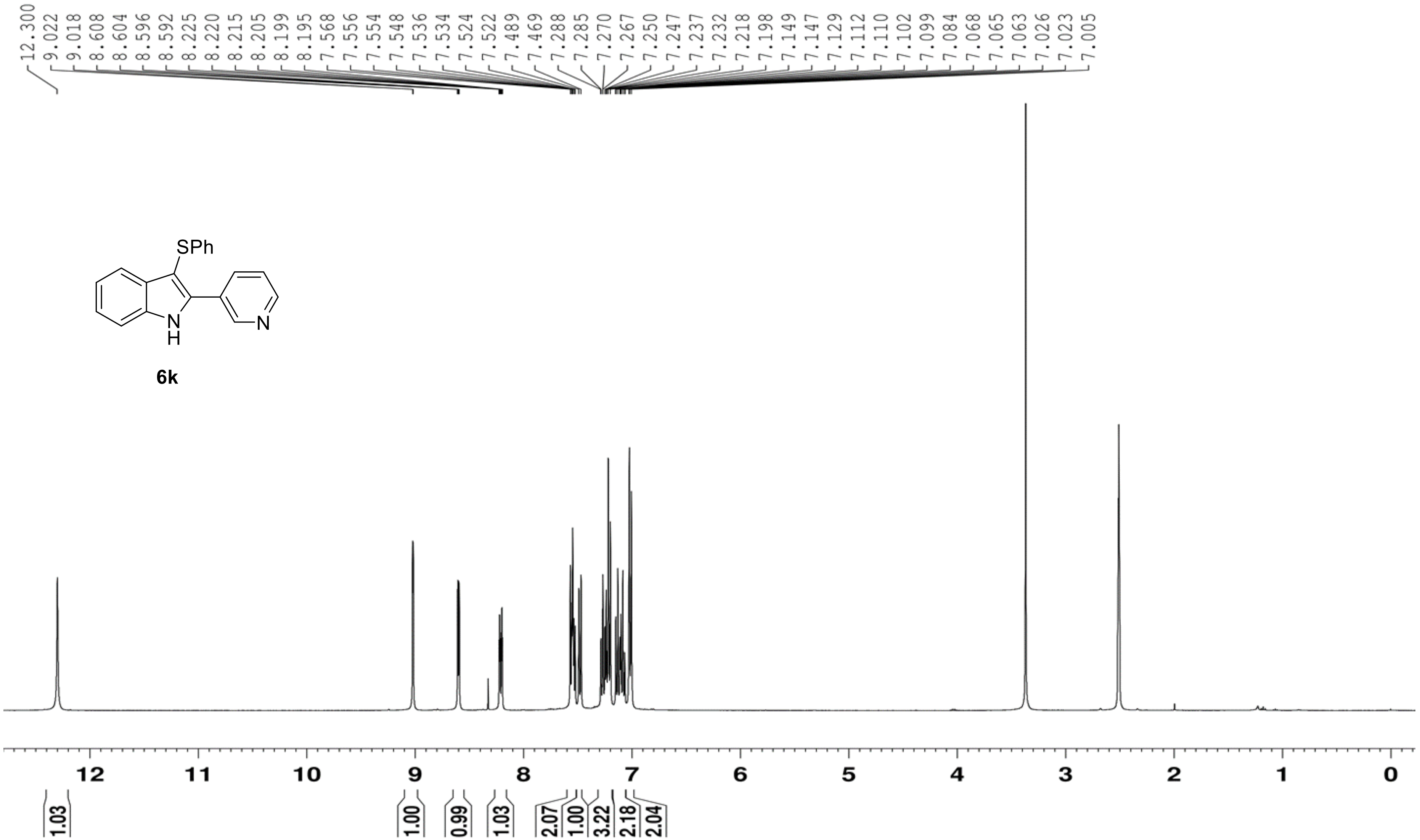

Supplementary Figure 111. ^{1}H NMR spectrum of **6k**.

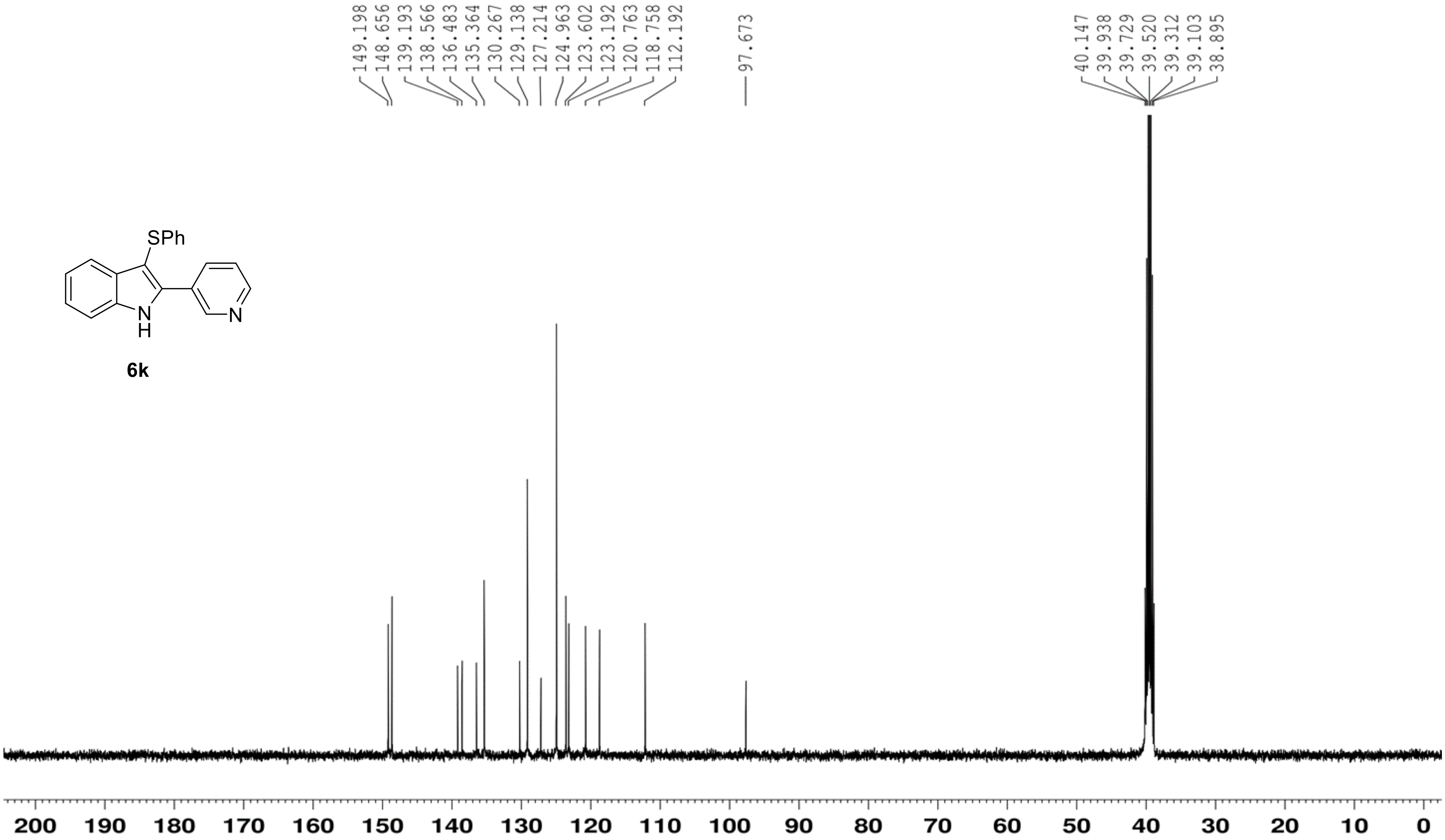

Supplementary Figure 112. ^{13}C NMR spectrum of **6k**.

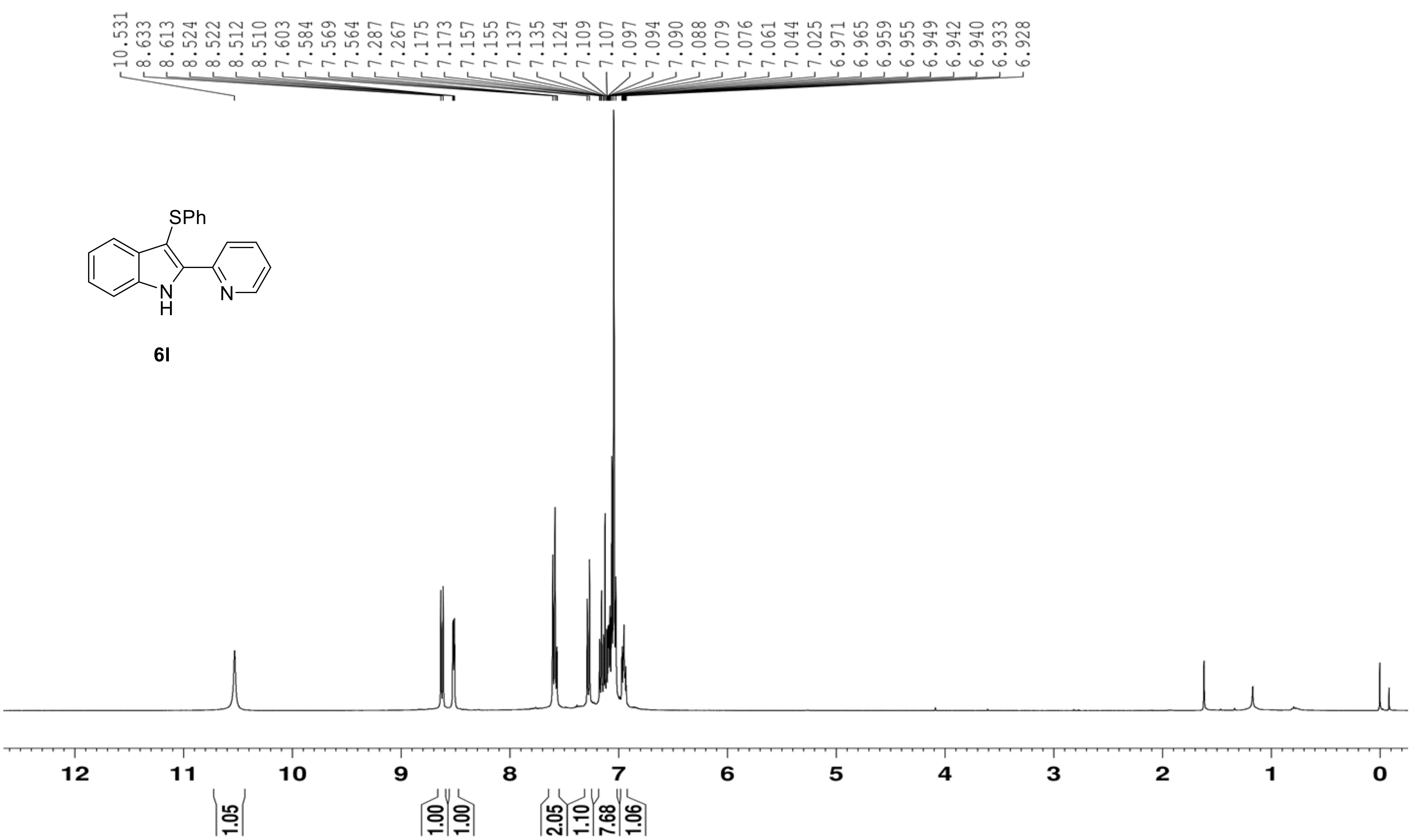

Supplementary Figure 113. ^{1}H NMR spectrum of **6l**.

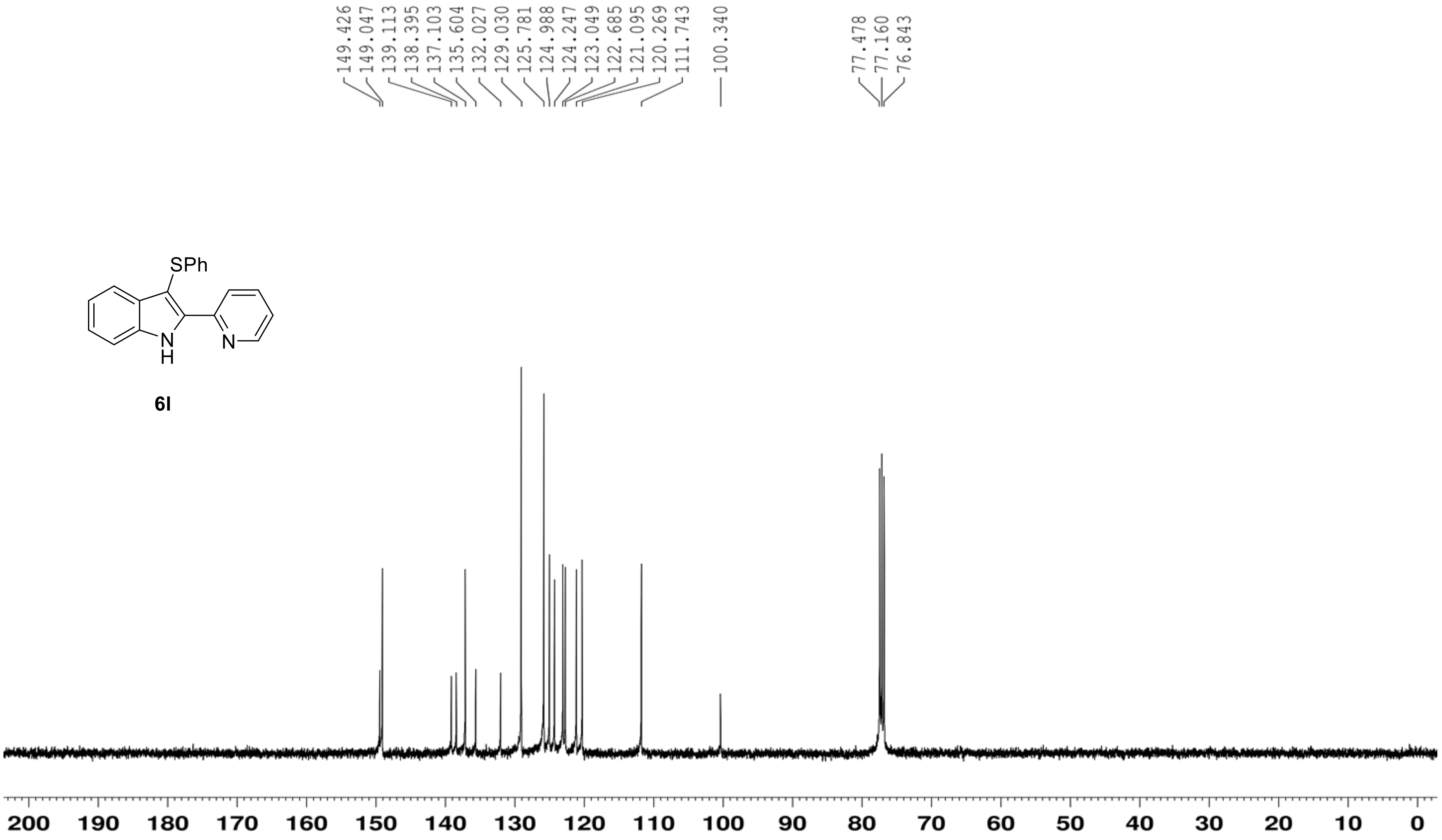

Supplementary Figure 114. ^{13}C NMR spectrum of **6l**.

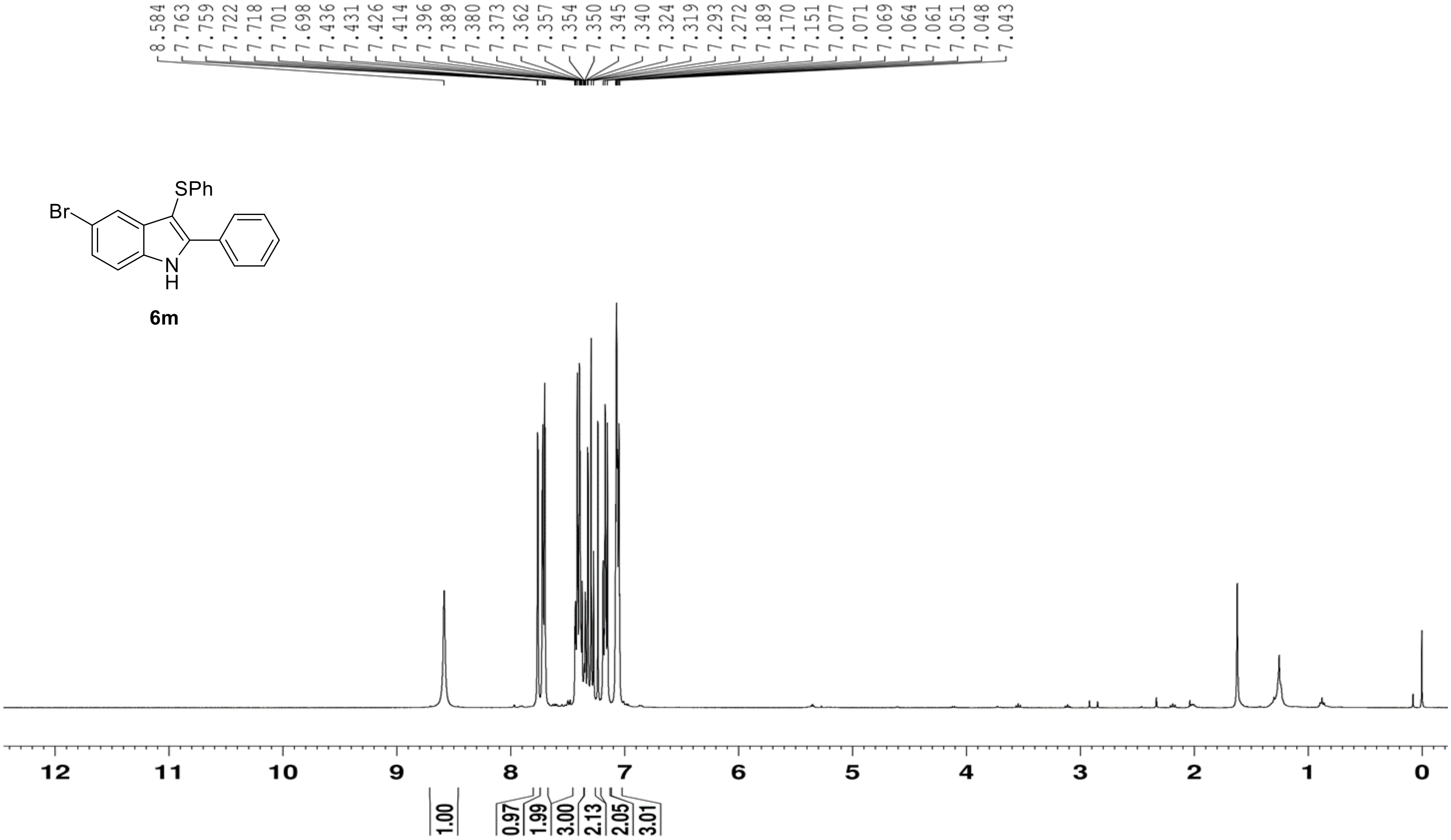

Supplementary Figure 115. ^{1}H NMR spectrum of **6m**.

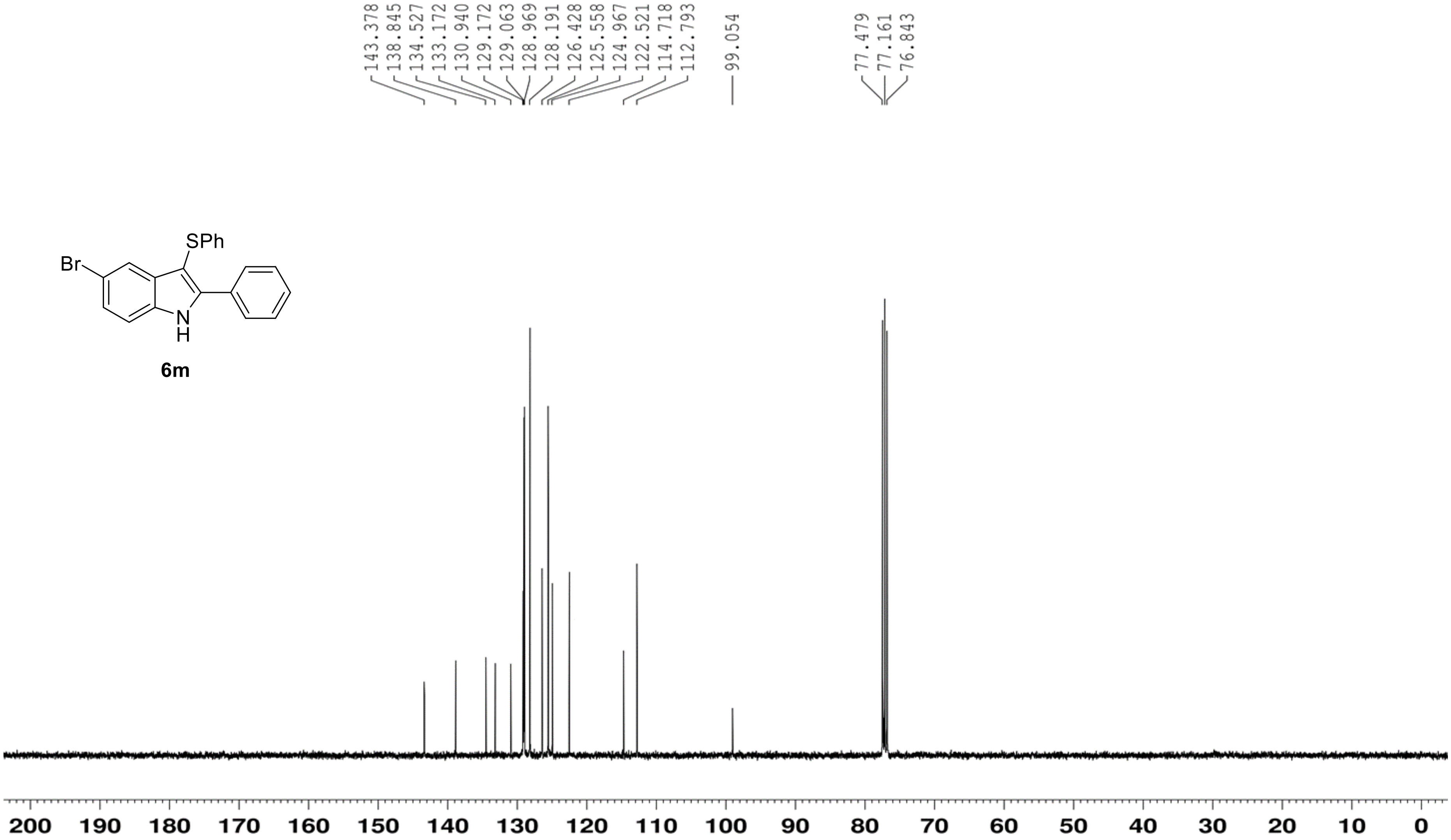

Supplementary Figure 116. ^{13}C NMR spectrum of **6m**.

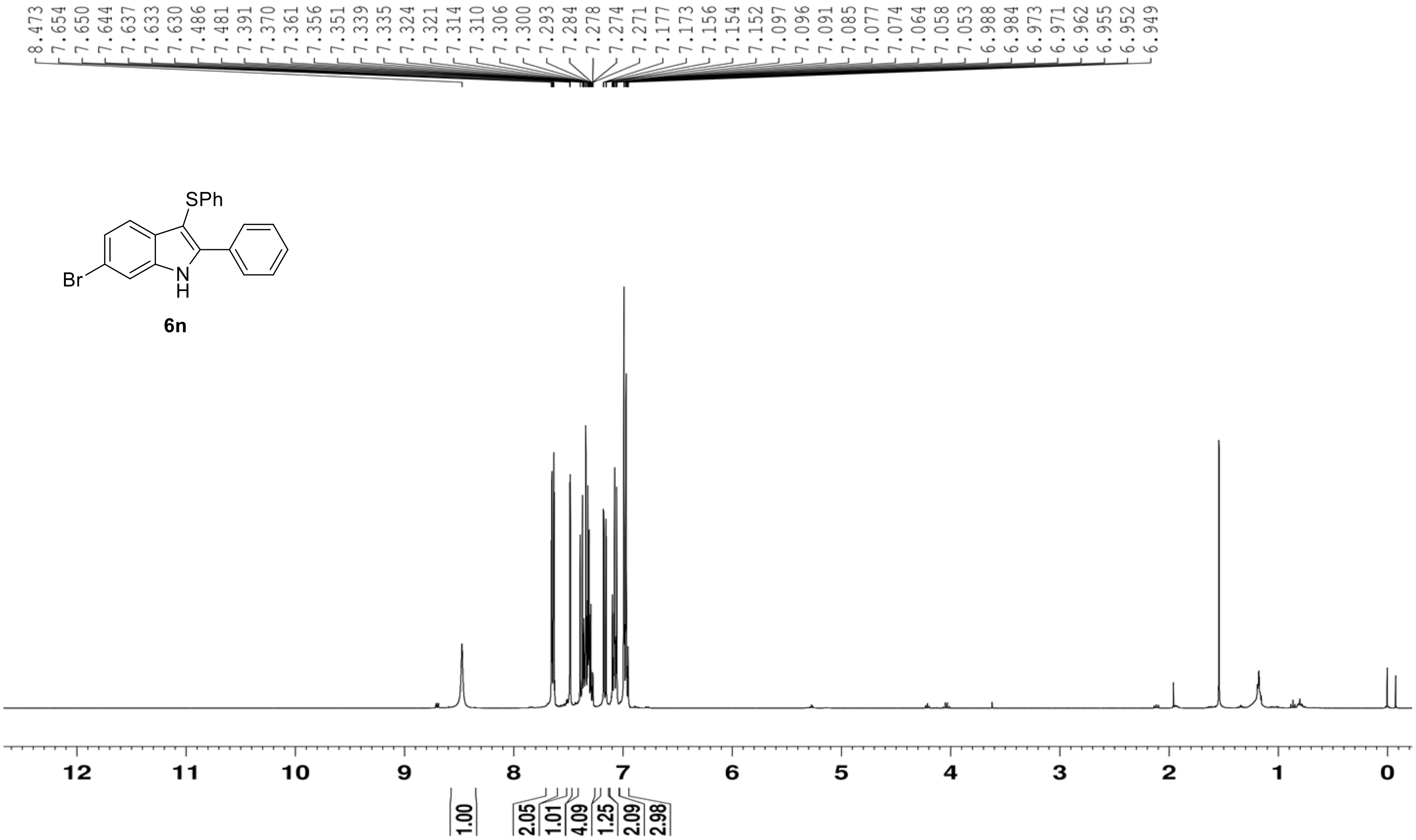

Supplementary Figure 117. ^{1}H NMR spectrum of **6n**.

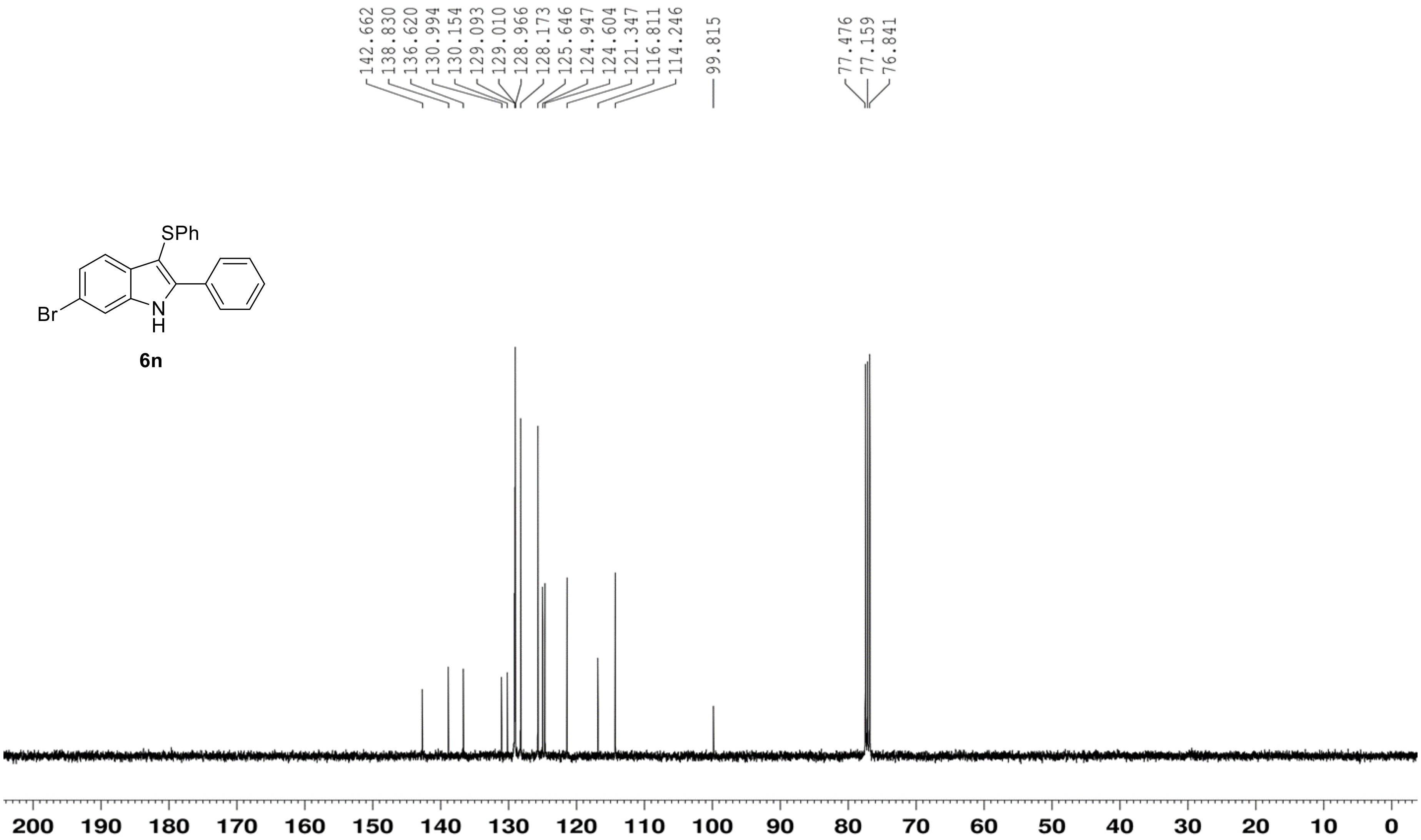

Supplementary Figure 118. ^{13}C NMR spectrum of **6n**.

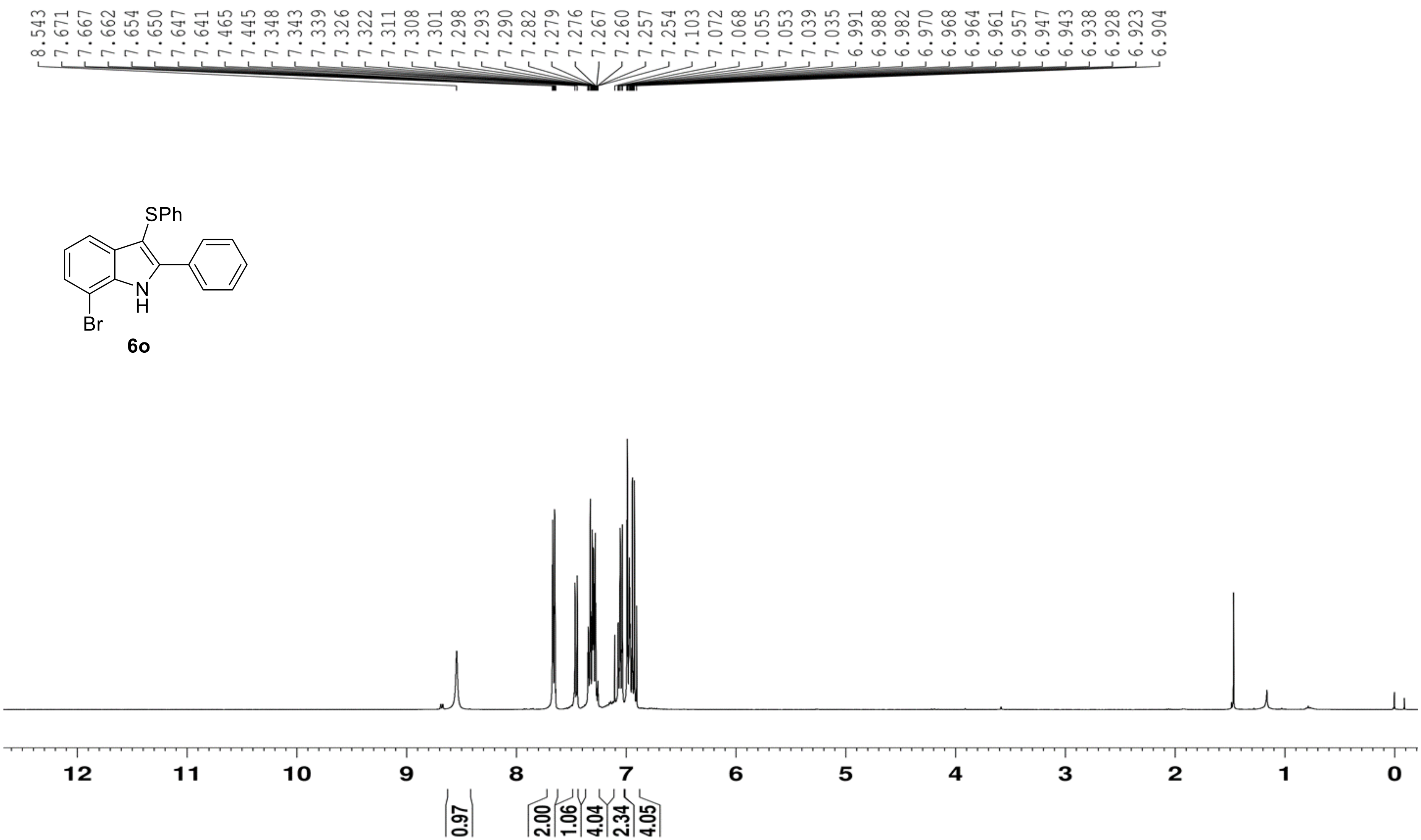

Supplementary Figure 119. ^{1}H NMR spectrum of **6o**.

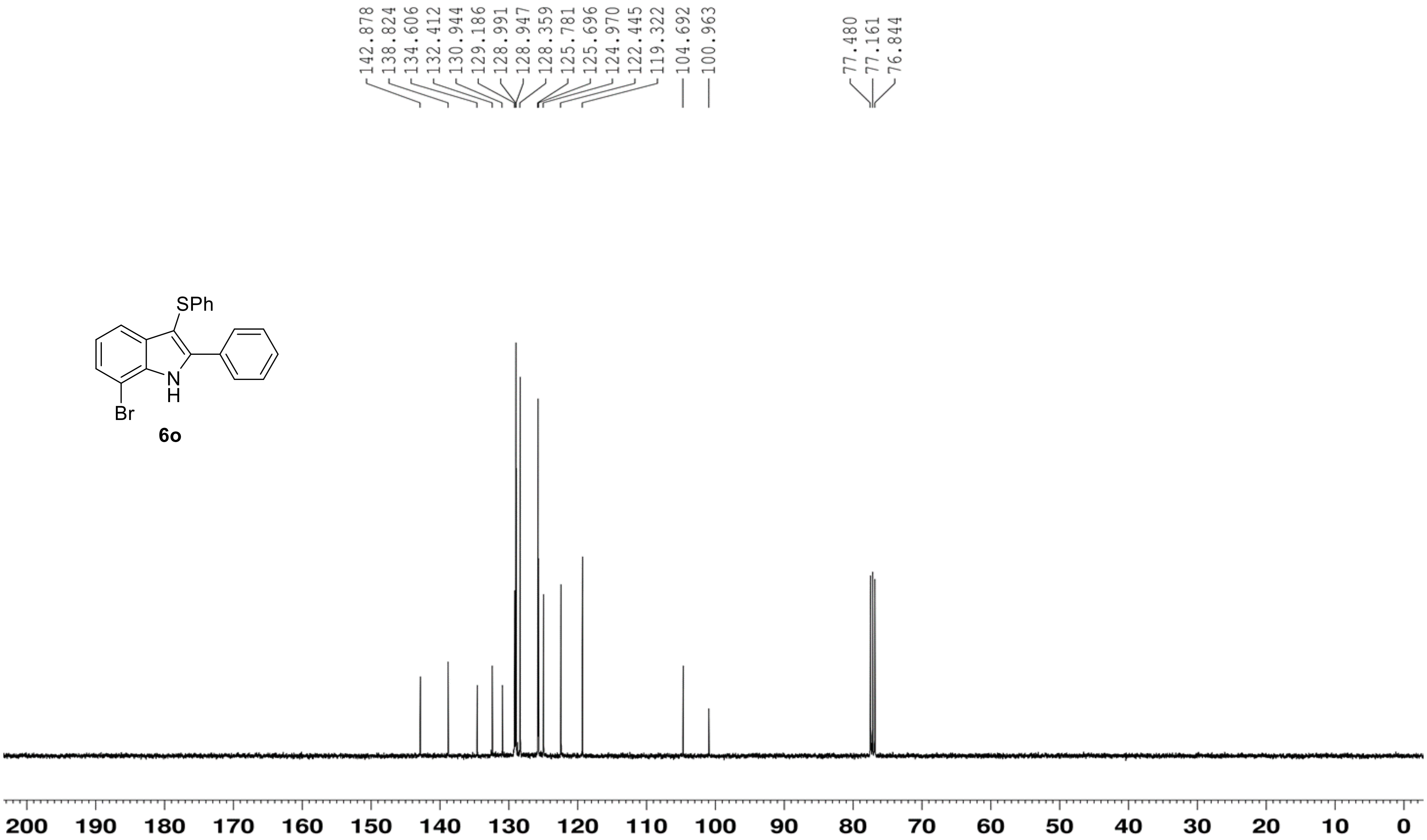

Supplementary Figure 120. ^{13}C NMR spectrum of **6o**.

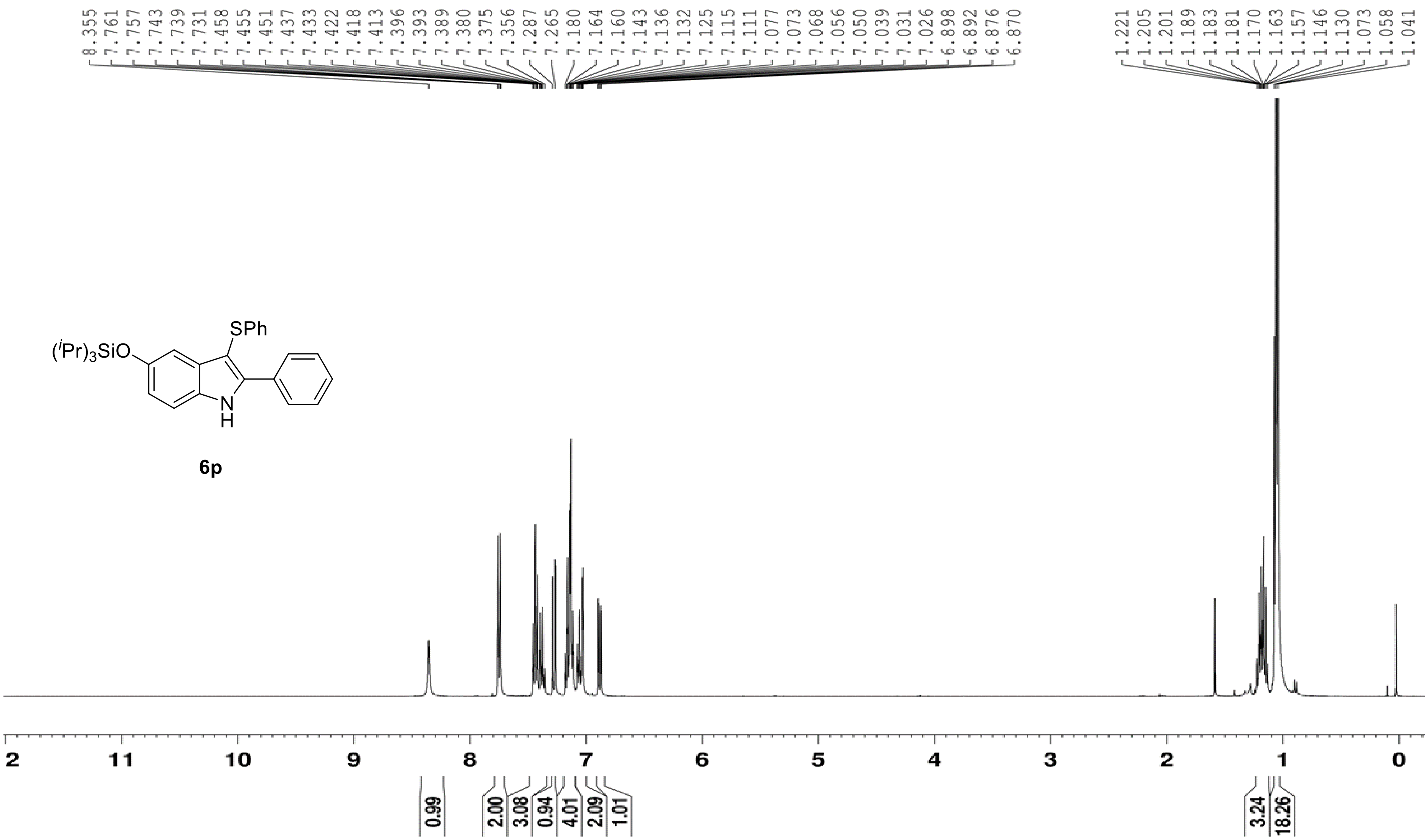

Supplementary Figure 121. ^{1}H NMR spectrum of **6p**.

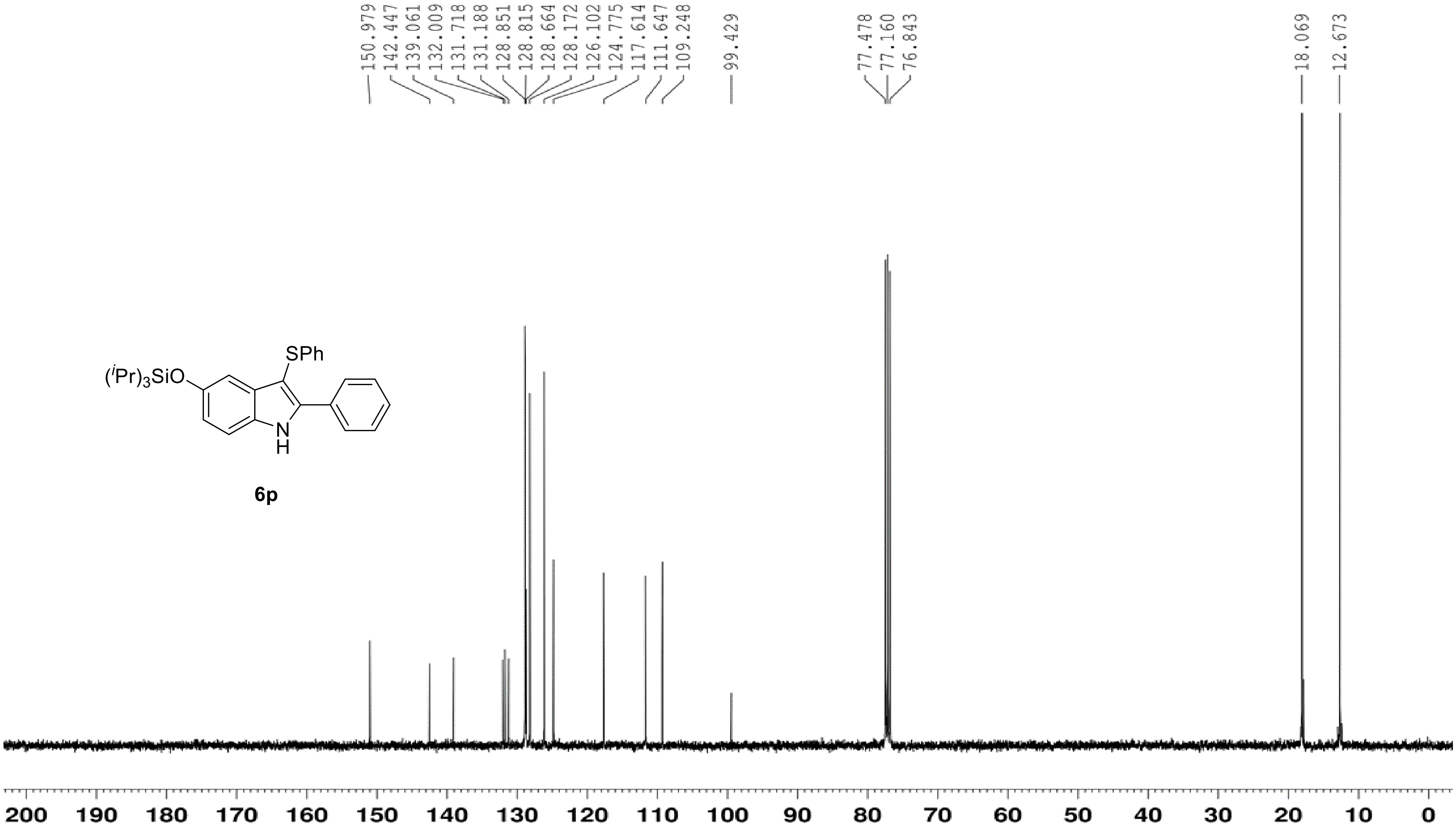

Supplementary Figure 122. ^{13}C NMR spectrum of **6p**.

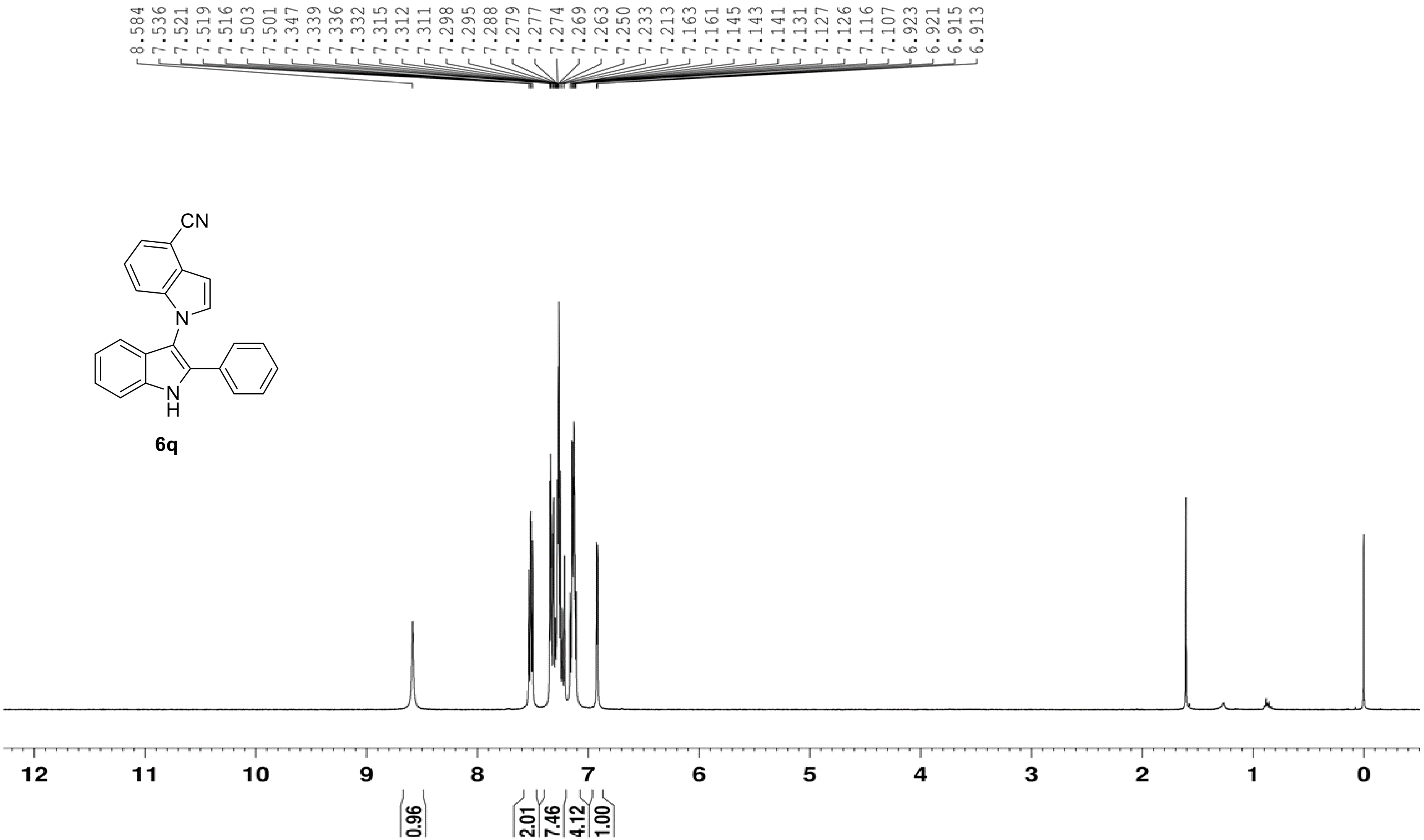

Supplementary Figure 123. ^{1}H NMR spectrum of **6q**.

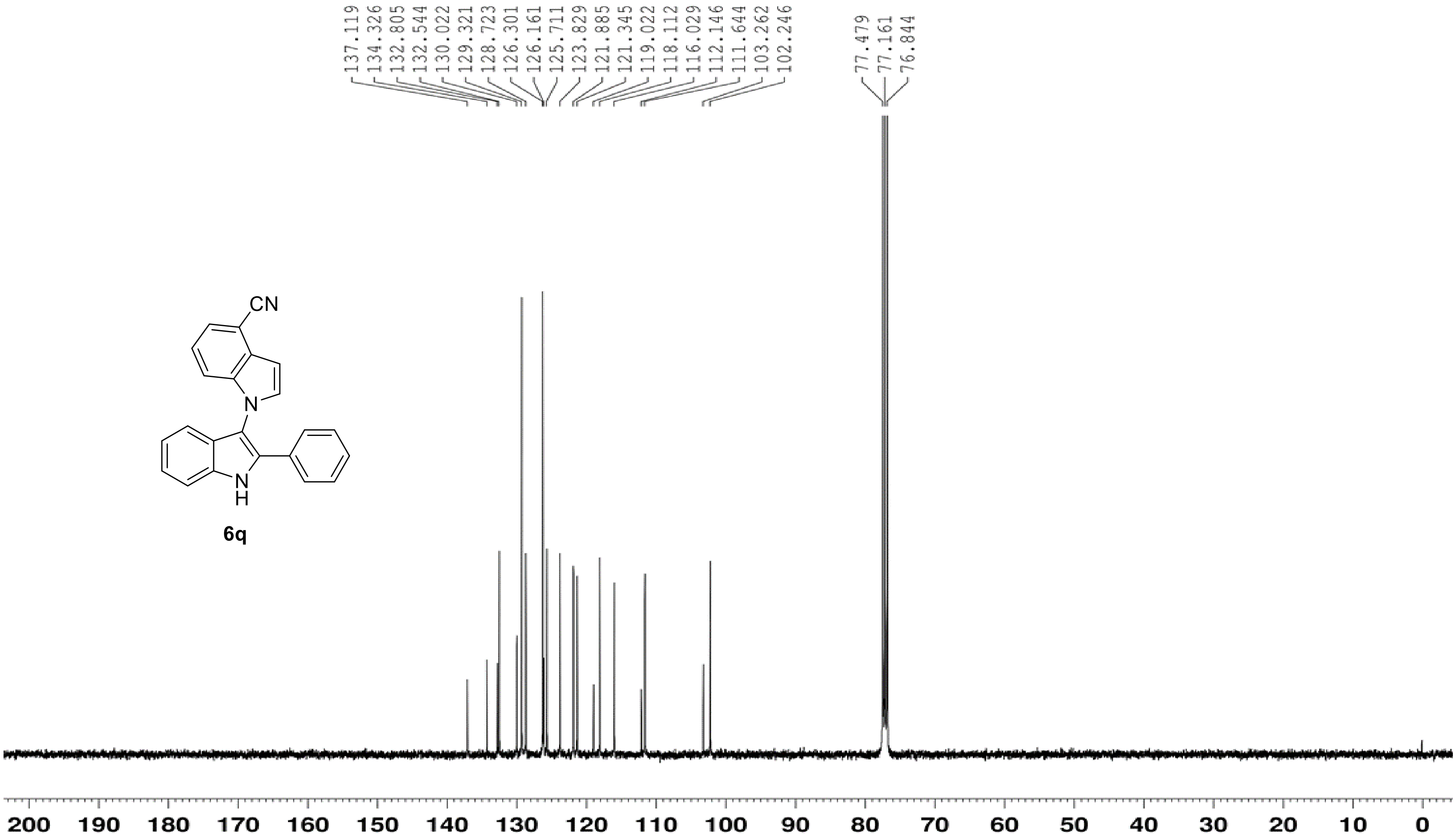

Supplementary Figure 124. ^{13}C NMR spectrum of **6q**.

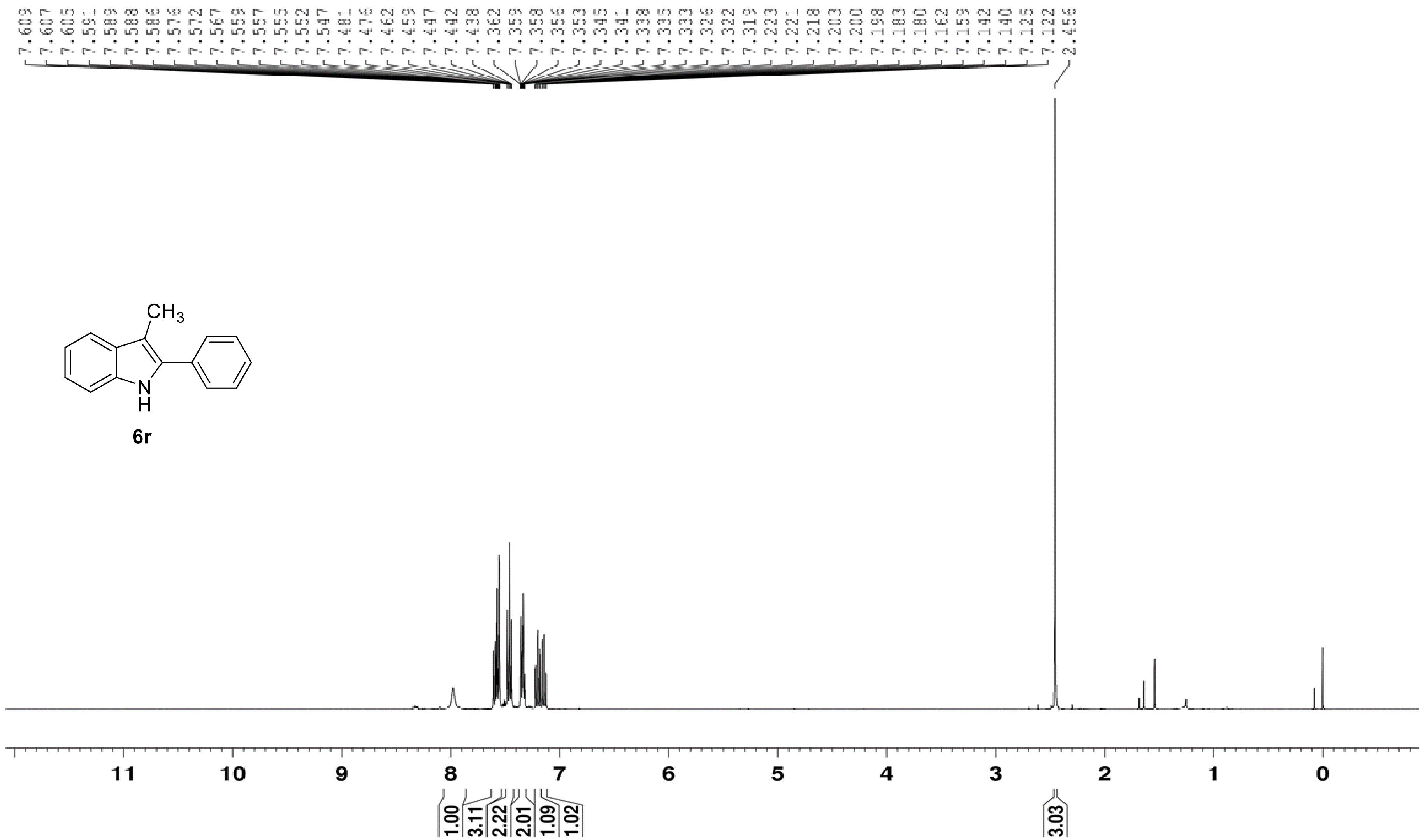

Supplementary Figure 125. ^{1}H NMR spectrum of **6r**.

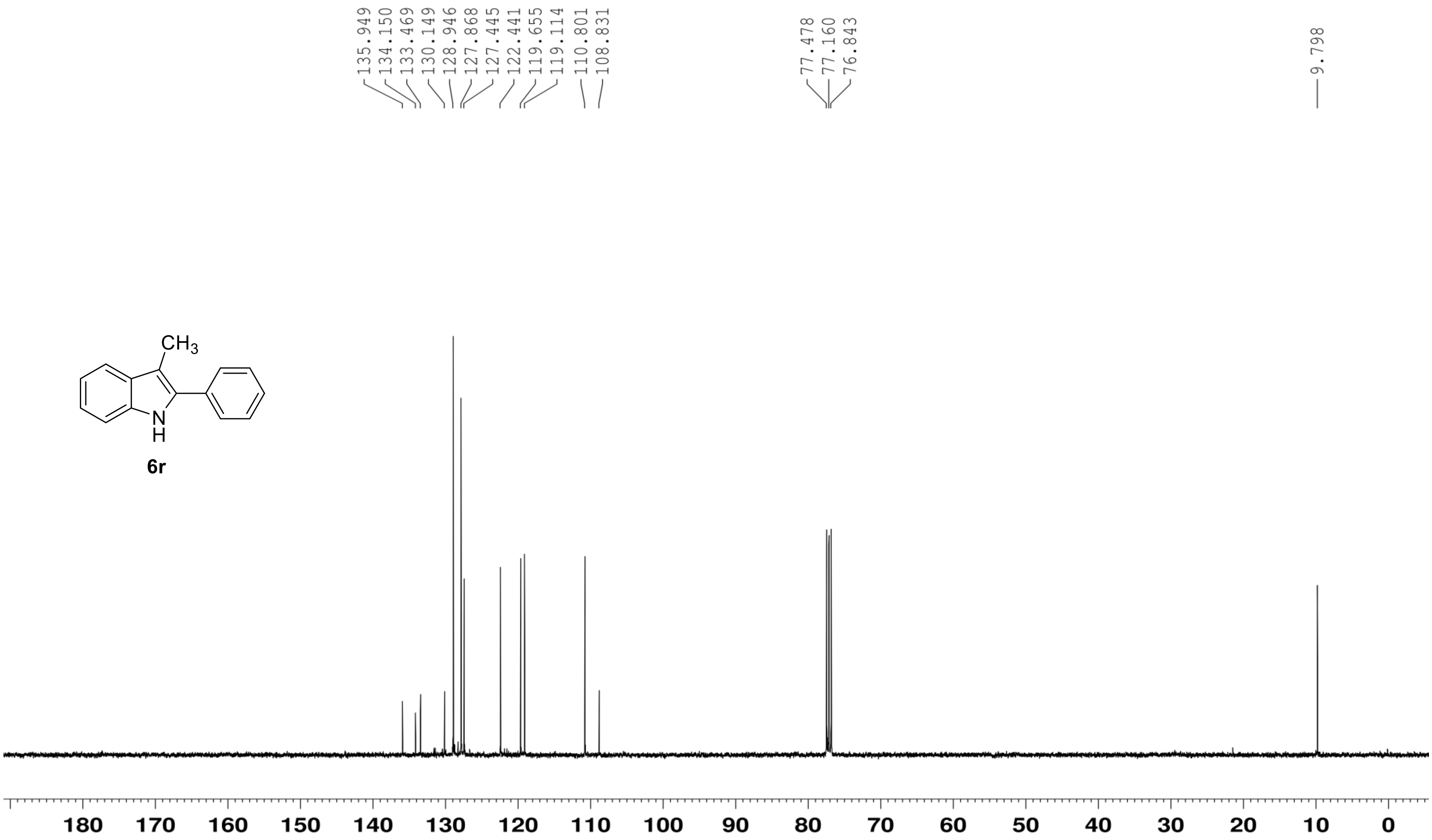

Supplementary Figure 126. ^{13}C NMR spectrum of **6r**.

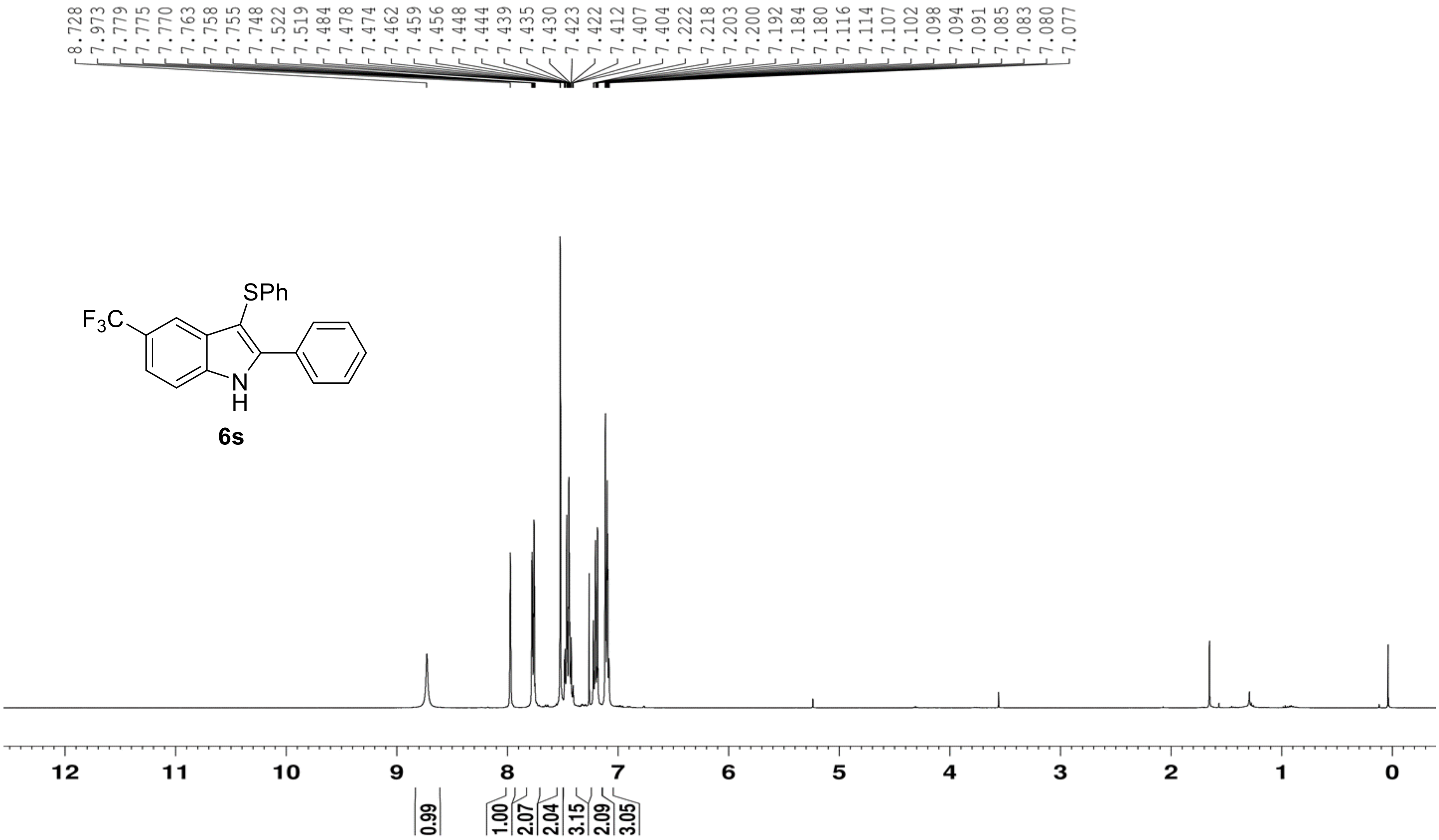

Supplementary Figure 127. ^{1}H NMR spectrum of **6s**.

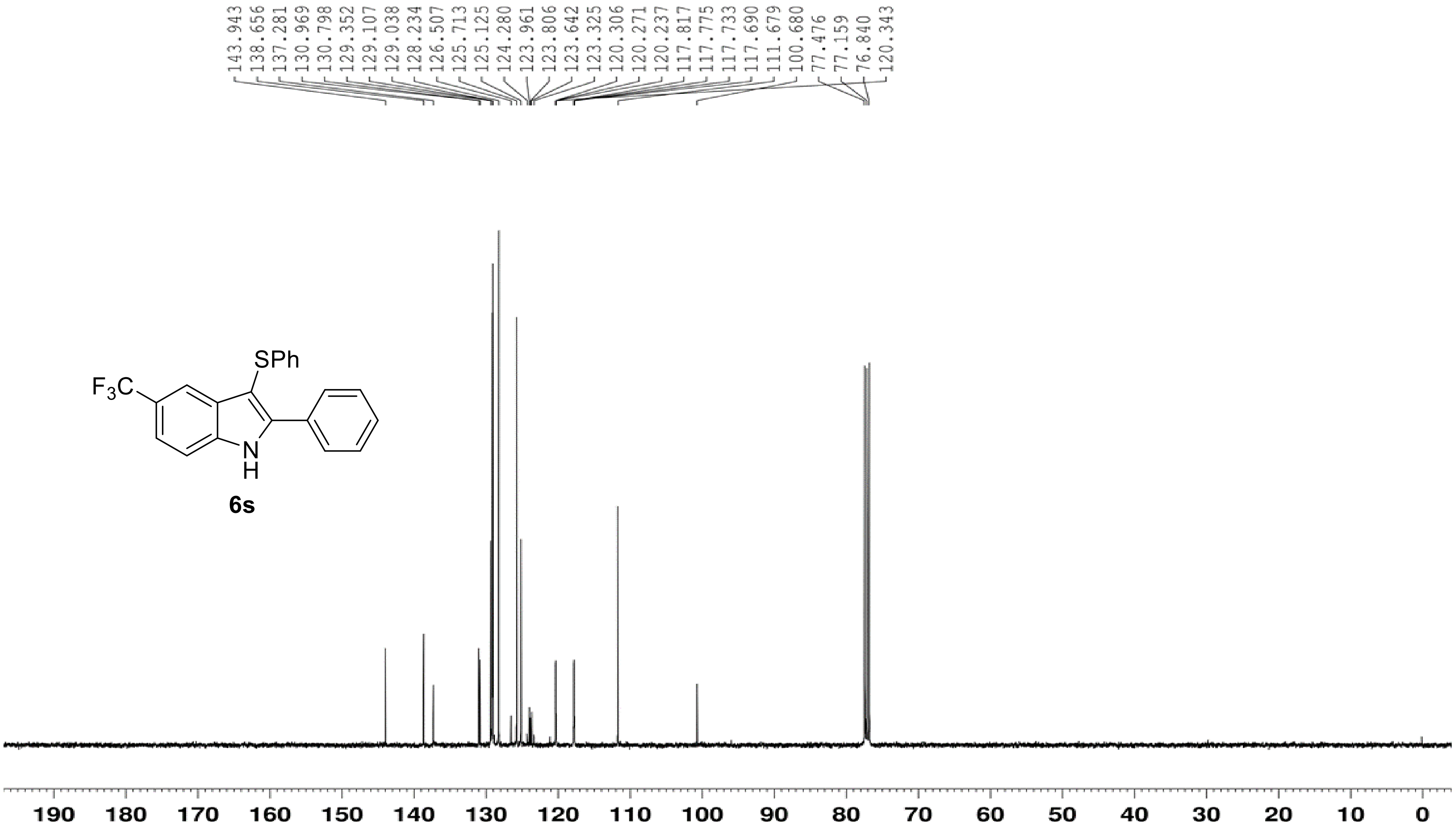

Supplementary Figure 128. ^{13}C NMR spectrum of **6s**.

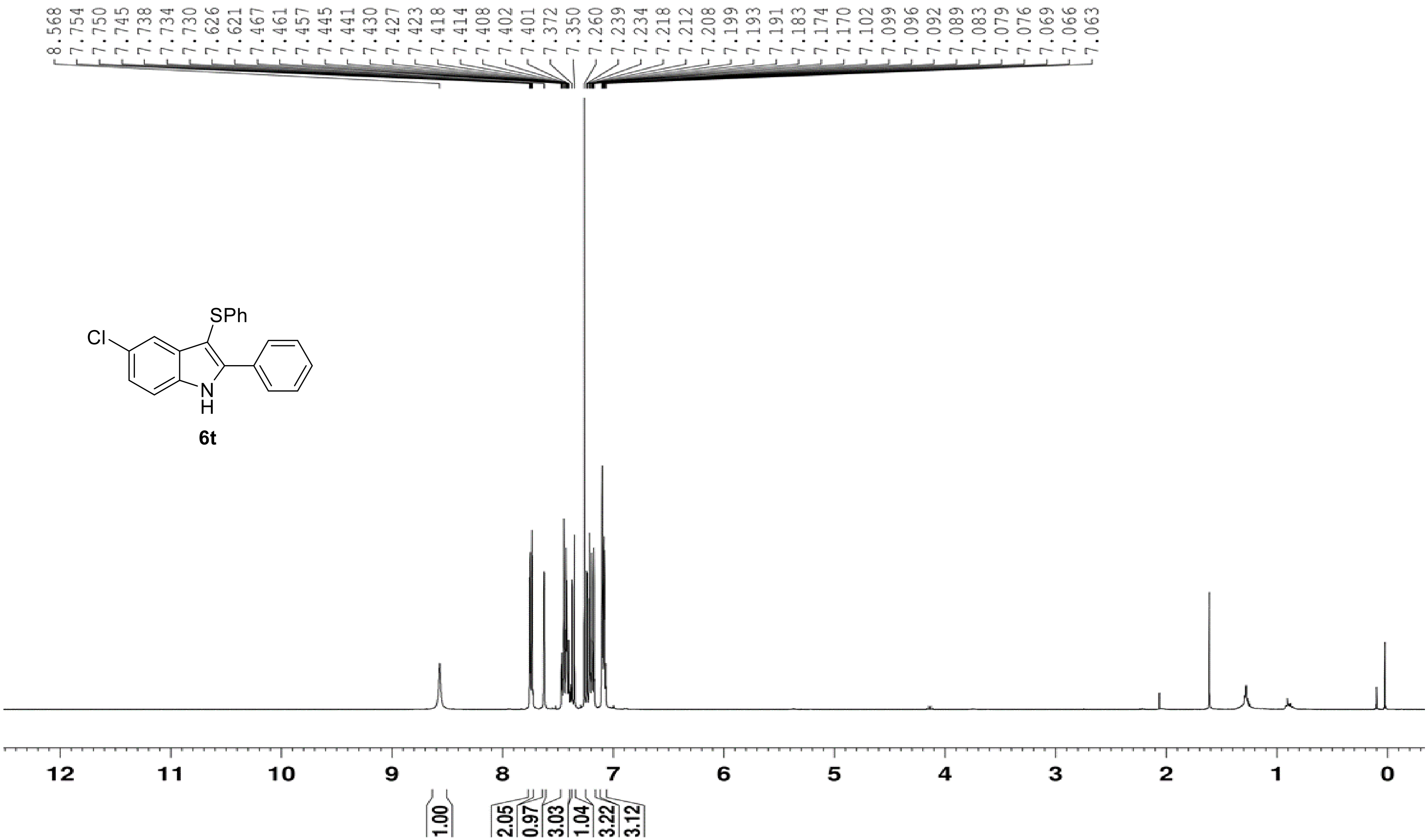

Supplementary Figure 129. ^{1}H NMR spectrum of **6t**.

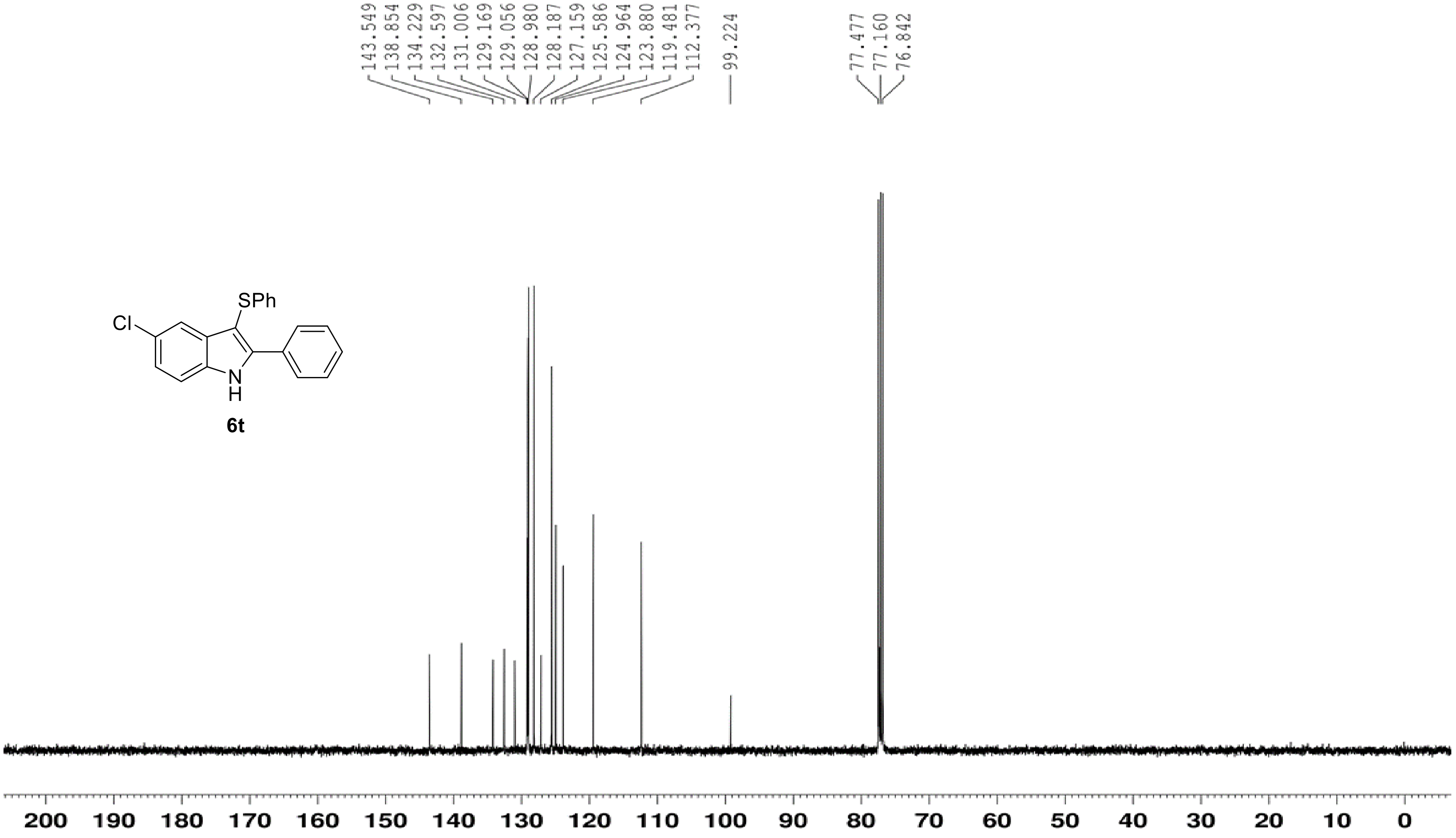

Supplementary Figure 130. ^{13}C NMR spectrum of **6t**.

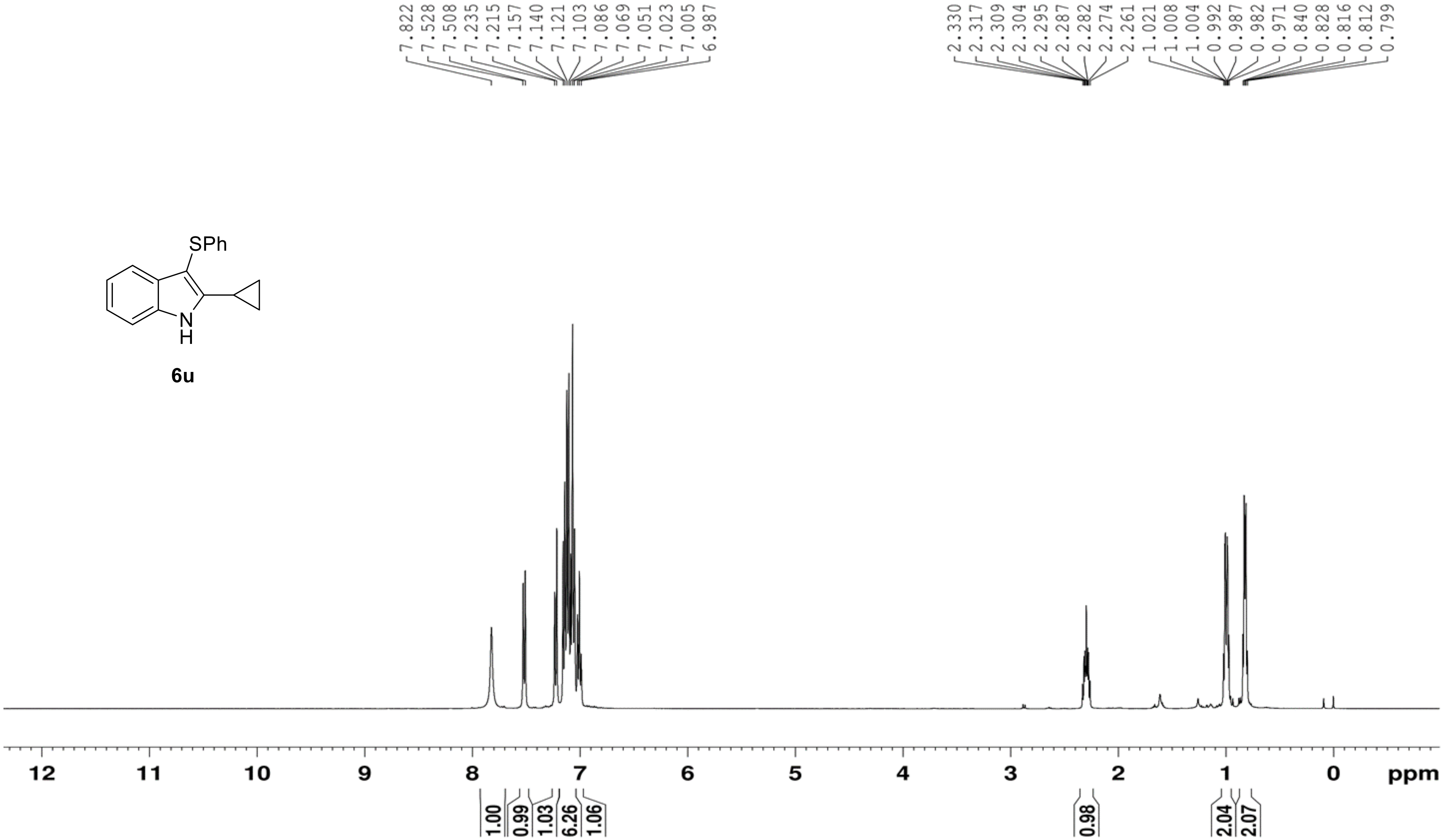

Supplementary Figure 131. ^{1}H NMR spectrum of **6u**.

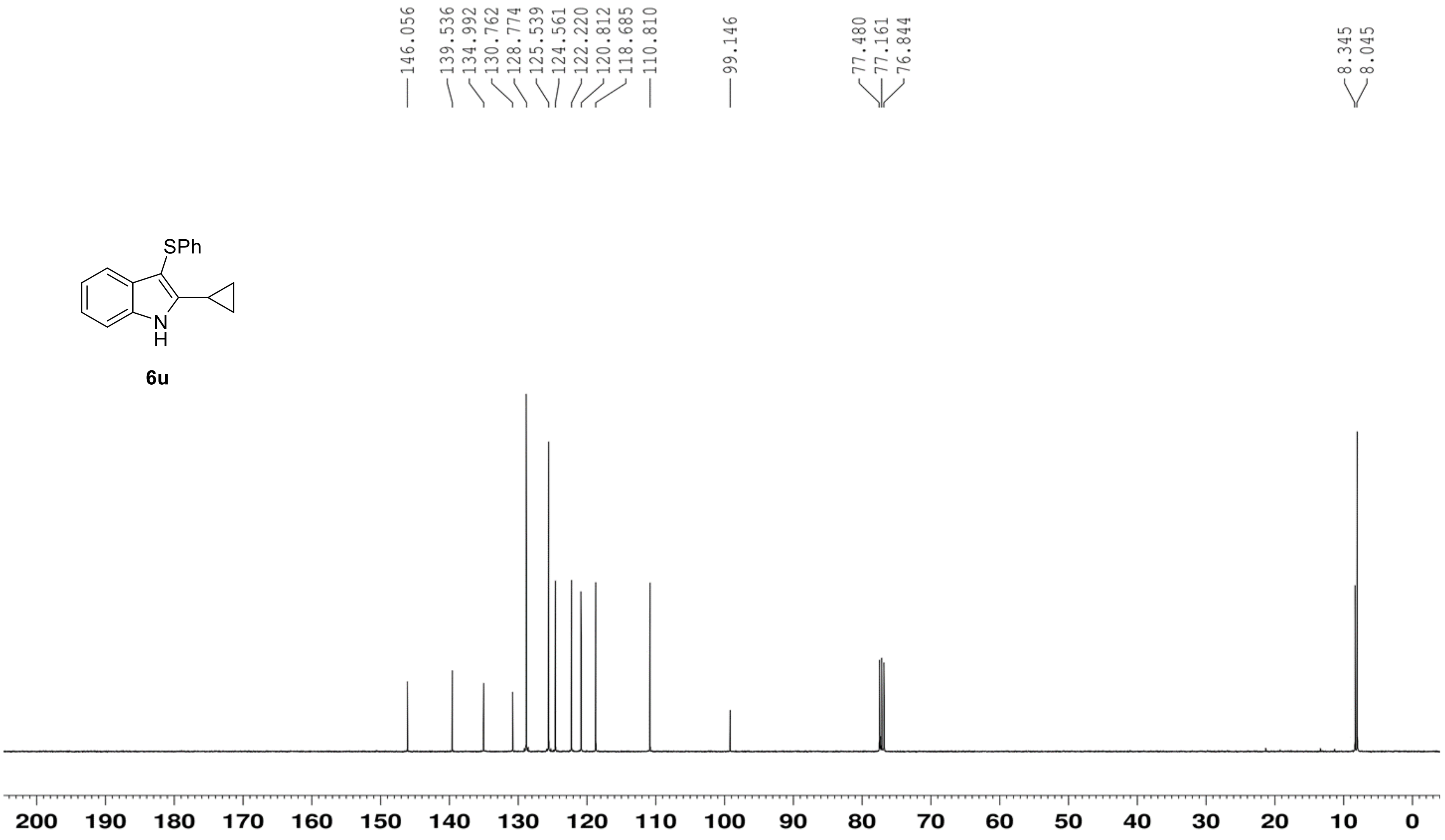

6u

Supplementary Figure 132. ^{13}C NMR spectrum of **6u**.

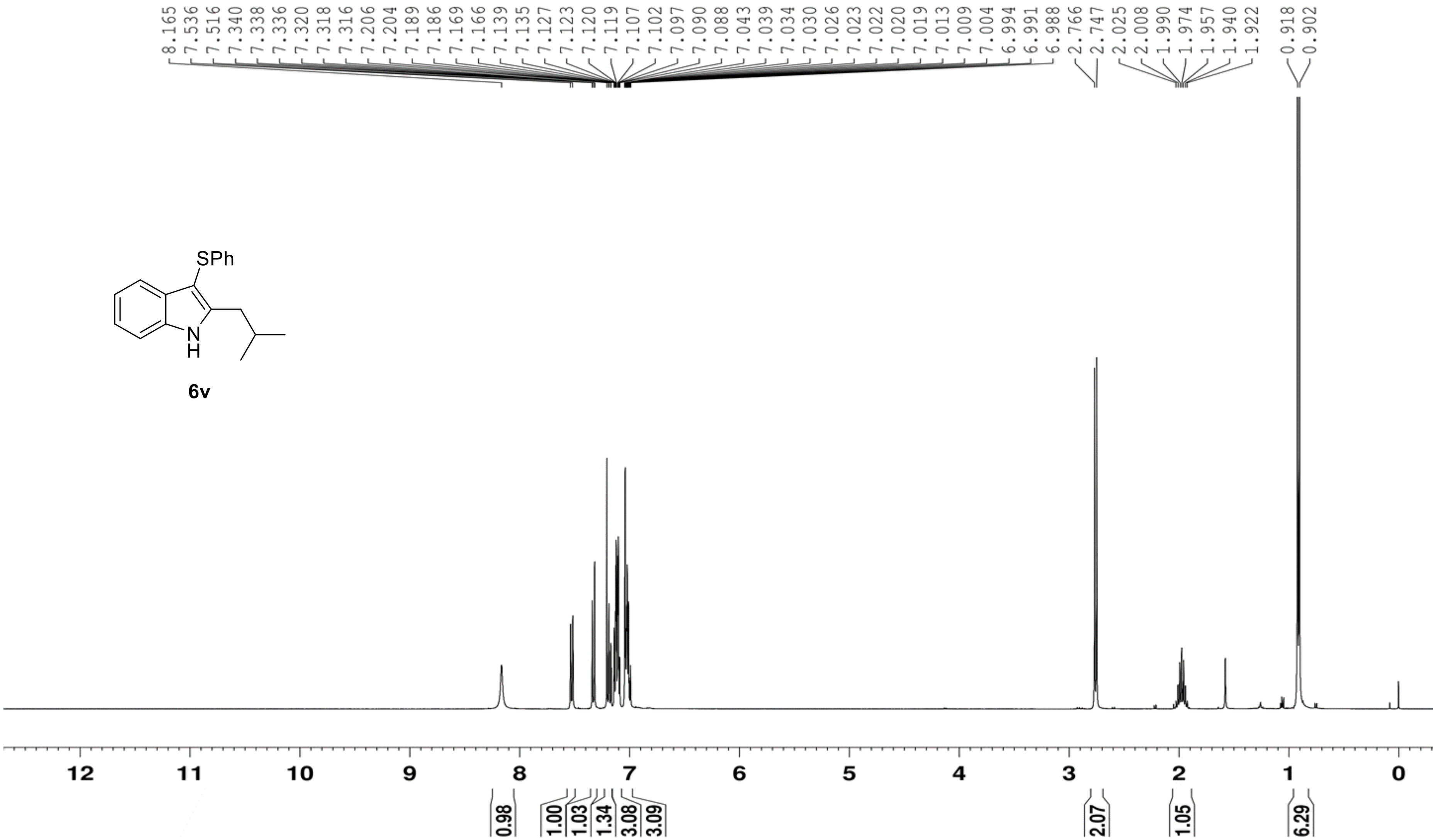

Supplementary Figure 133. ^{1}H NMR spectrum of **6v**.

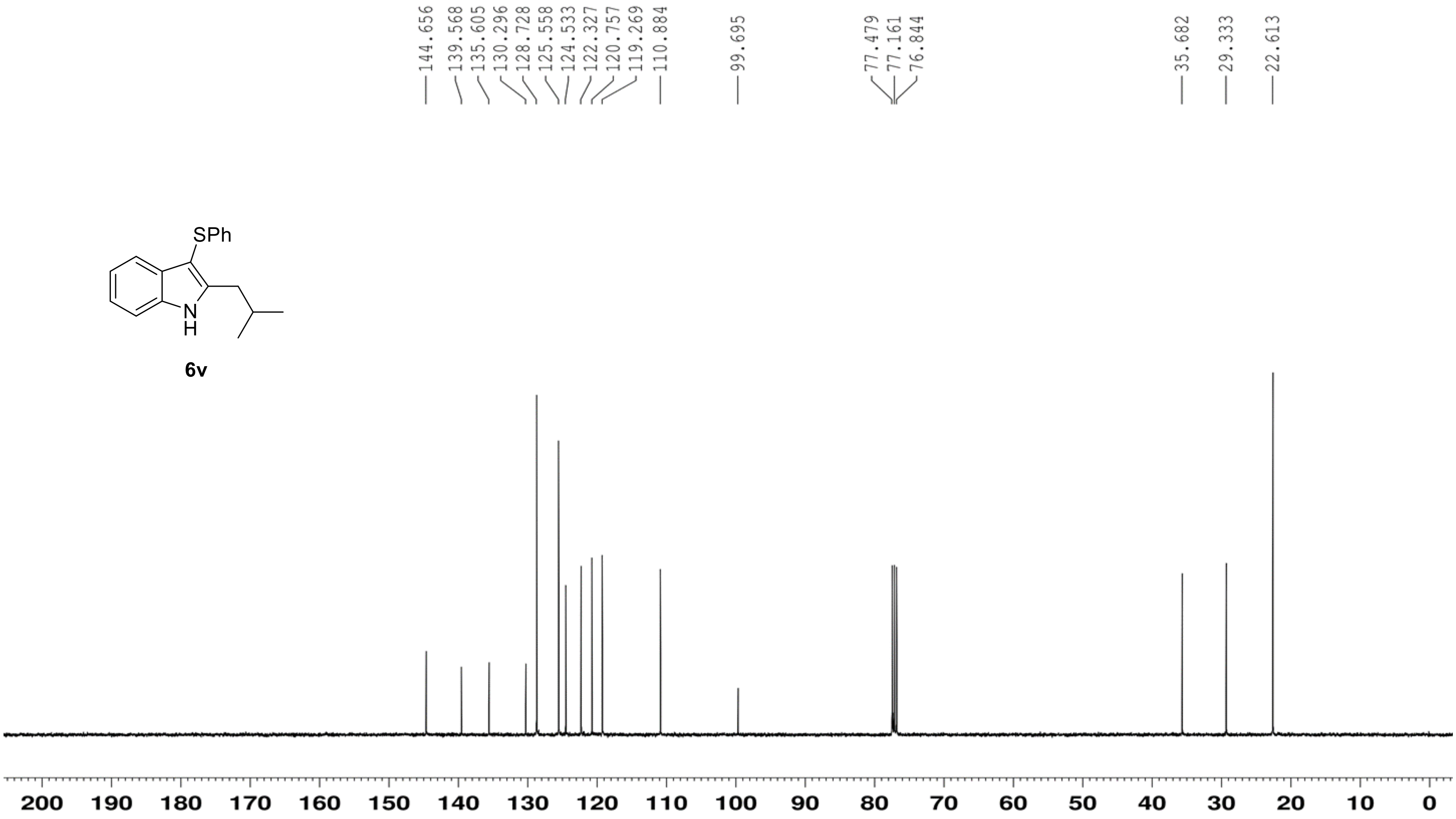

Supplementary Figure 134. ^{13}C NMR spectrum of **6v**.

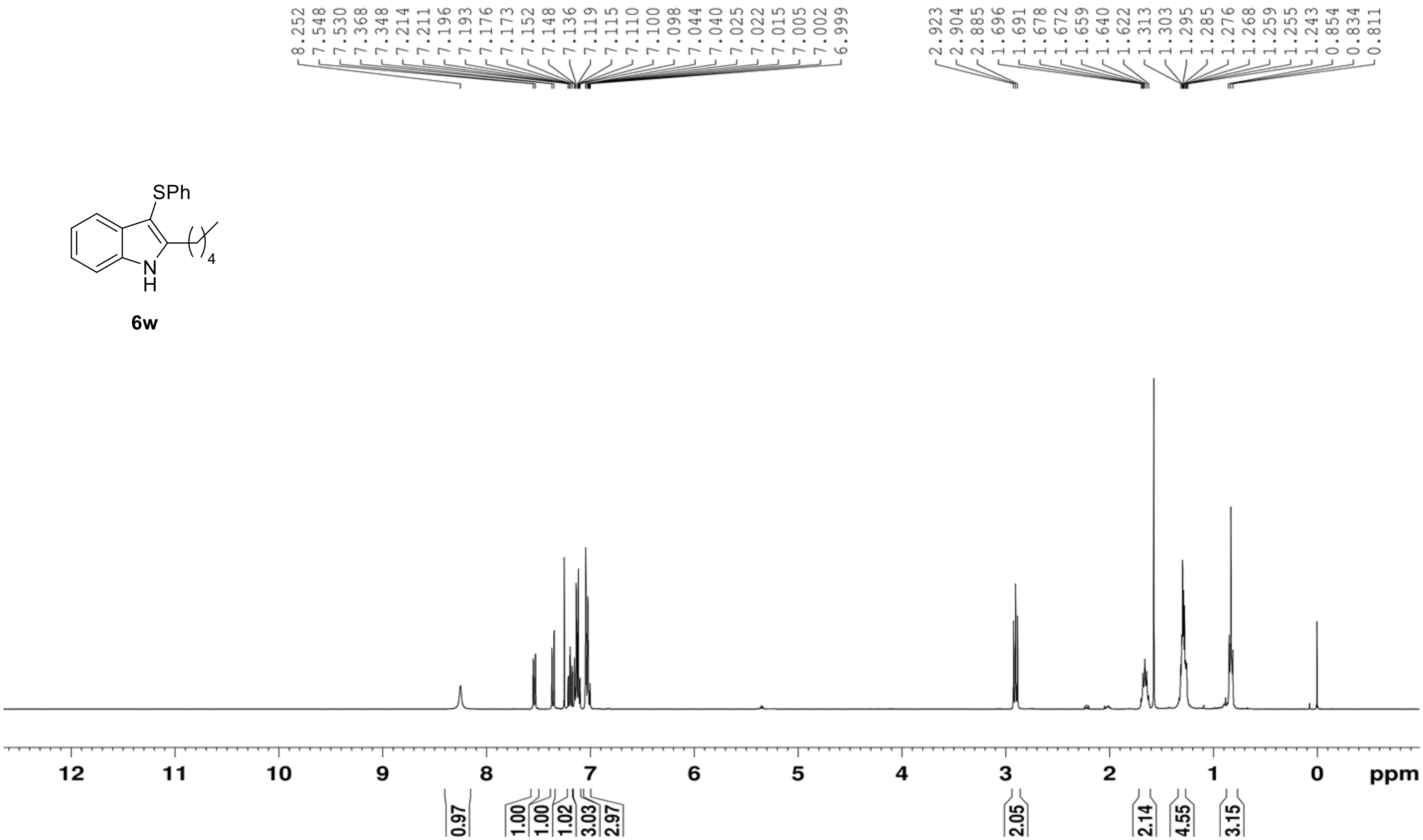

6w

Supplementary Figure 135. ^{1}H NMR spectrum of **6w**.

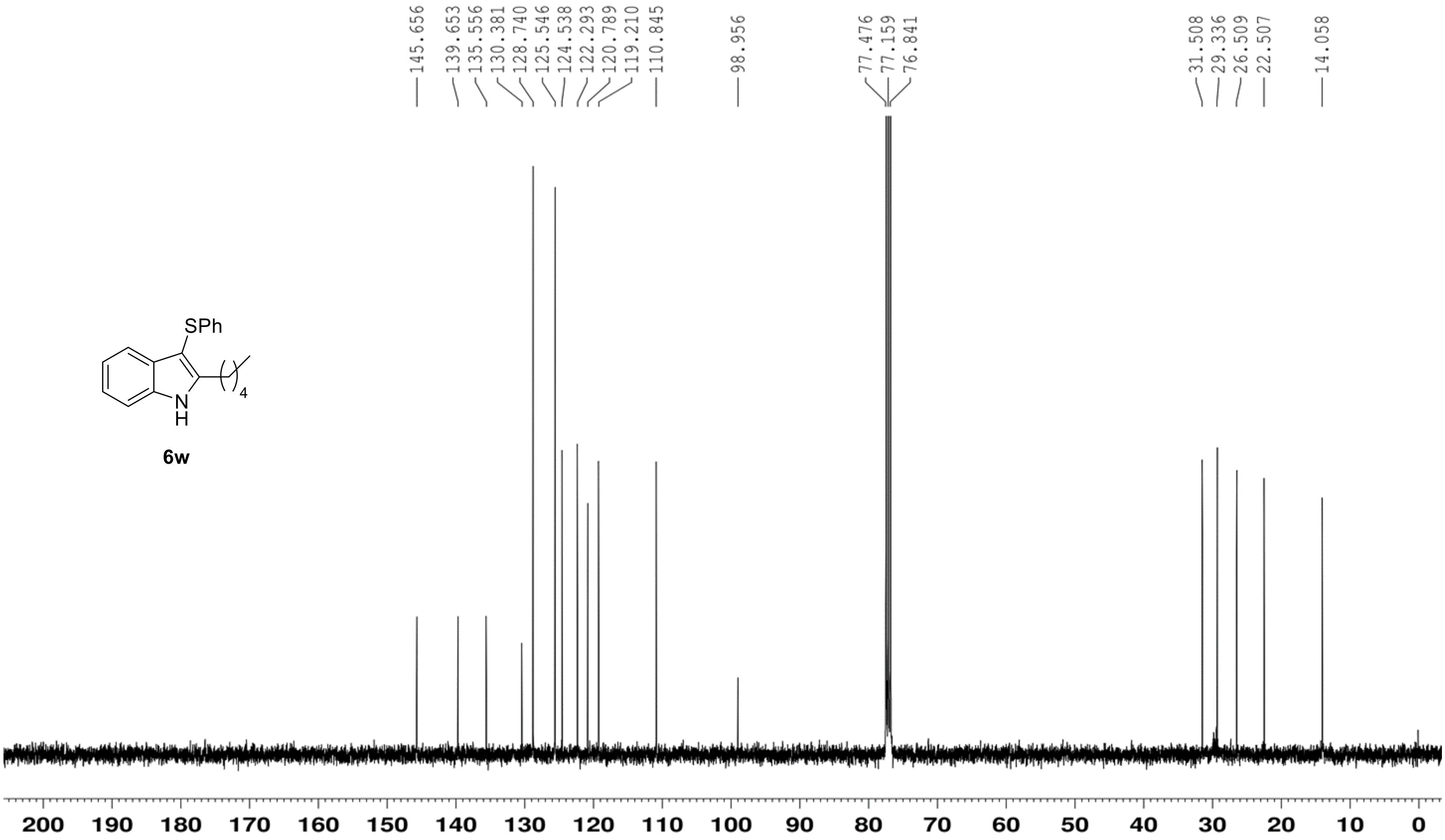

Supplementary Figure 136. ^{13}C NMR spectrum of **6w**.

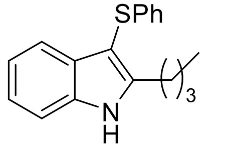

6x

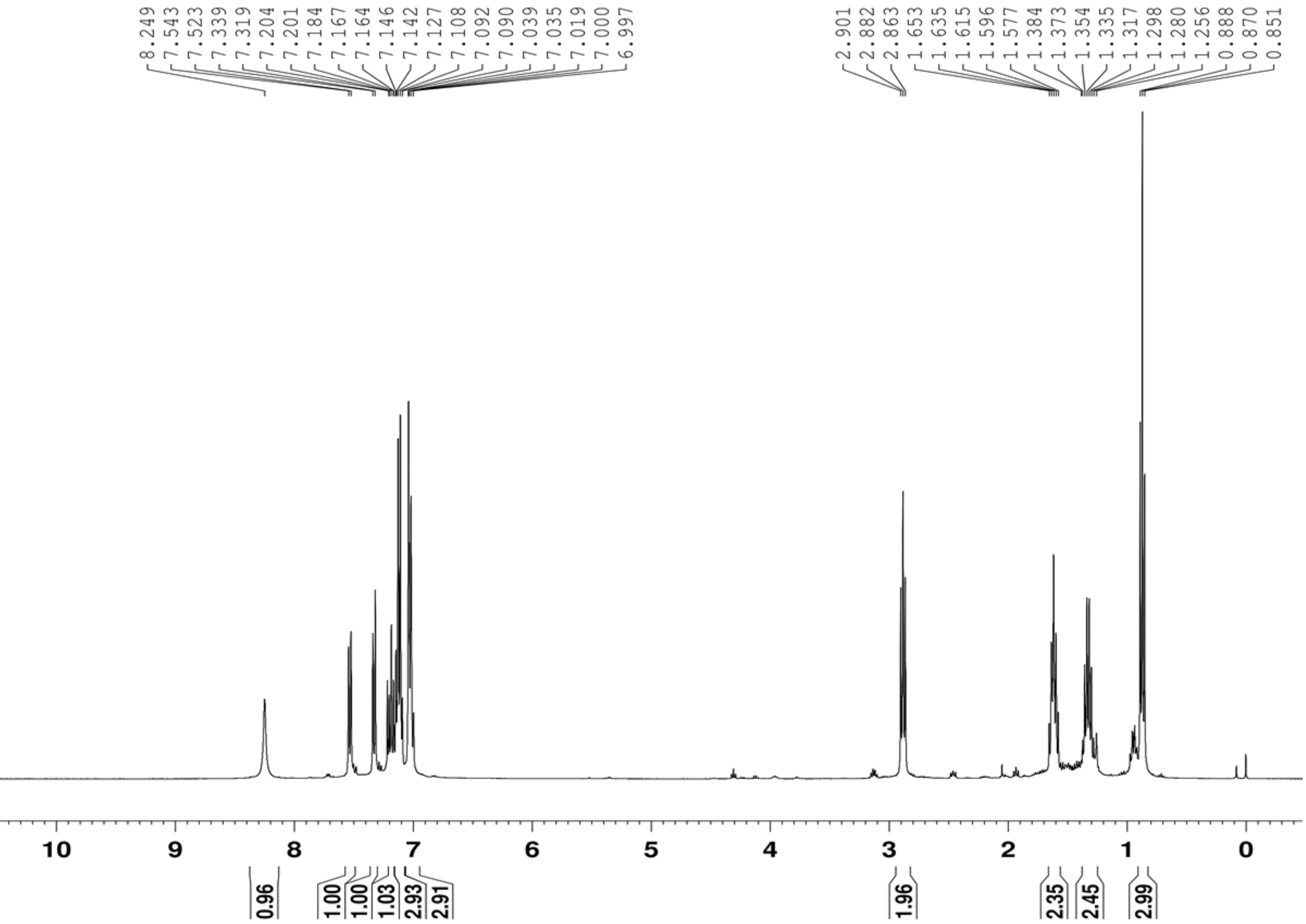

Supplementary Figure 137. ^{1}H NMR spectrum of **6x**.

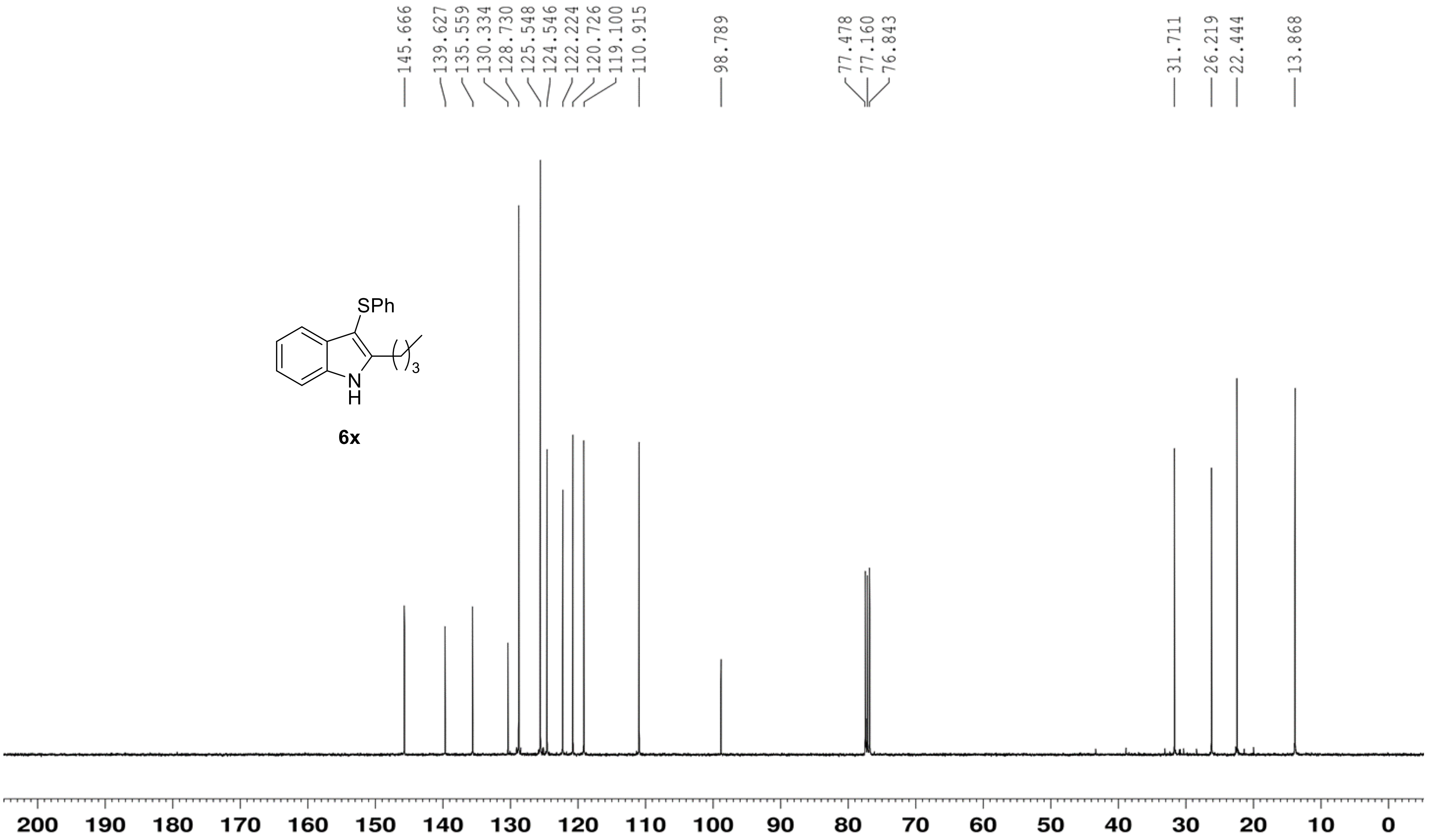

Supplementary Figure 138. ^{13}C NMR spectrum of **6x**.

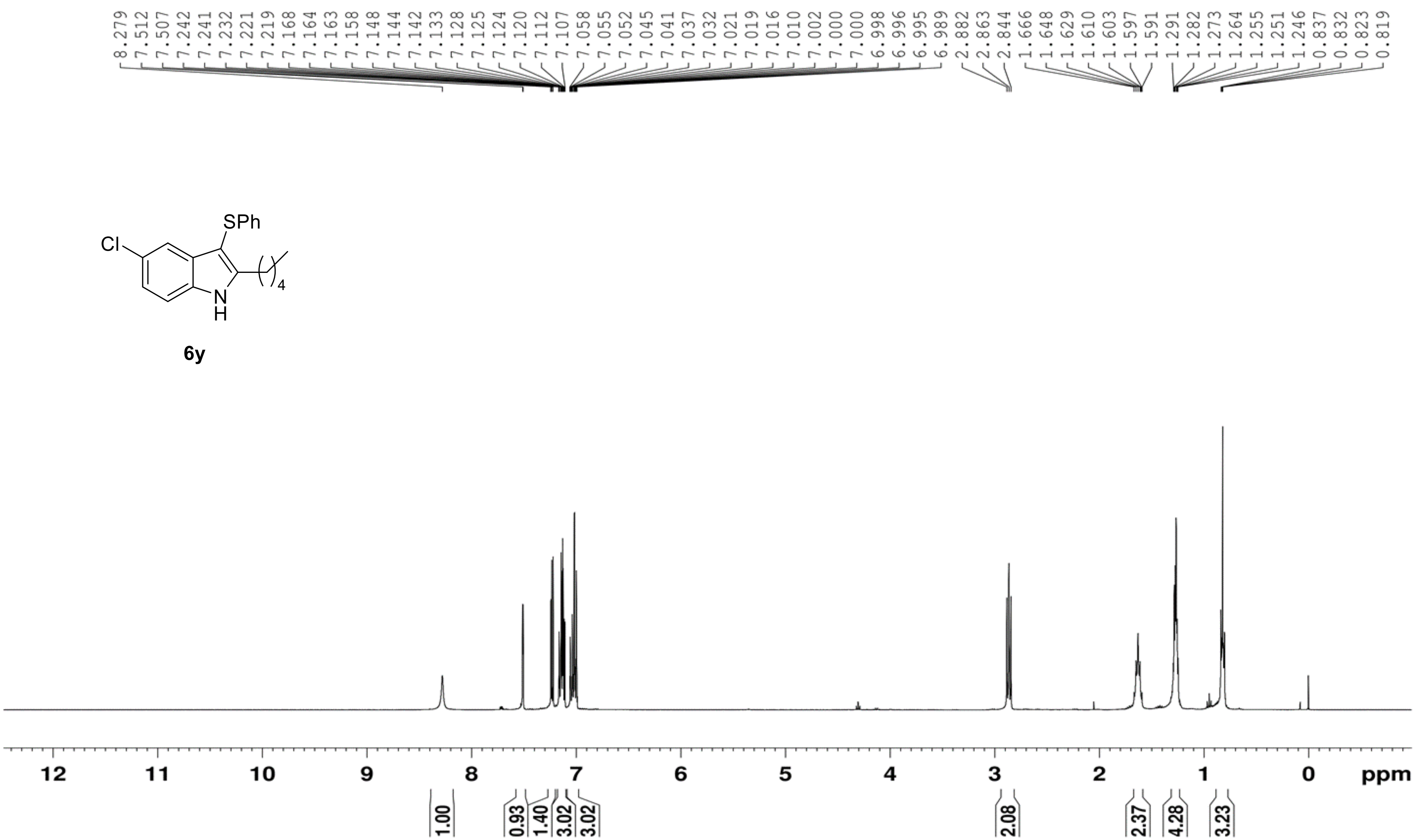

Supplementary Figure 139. ^{1}H NMR spectrum of **6y**.

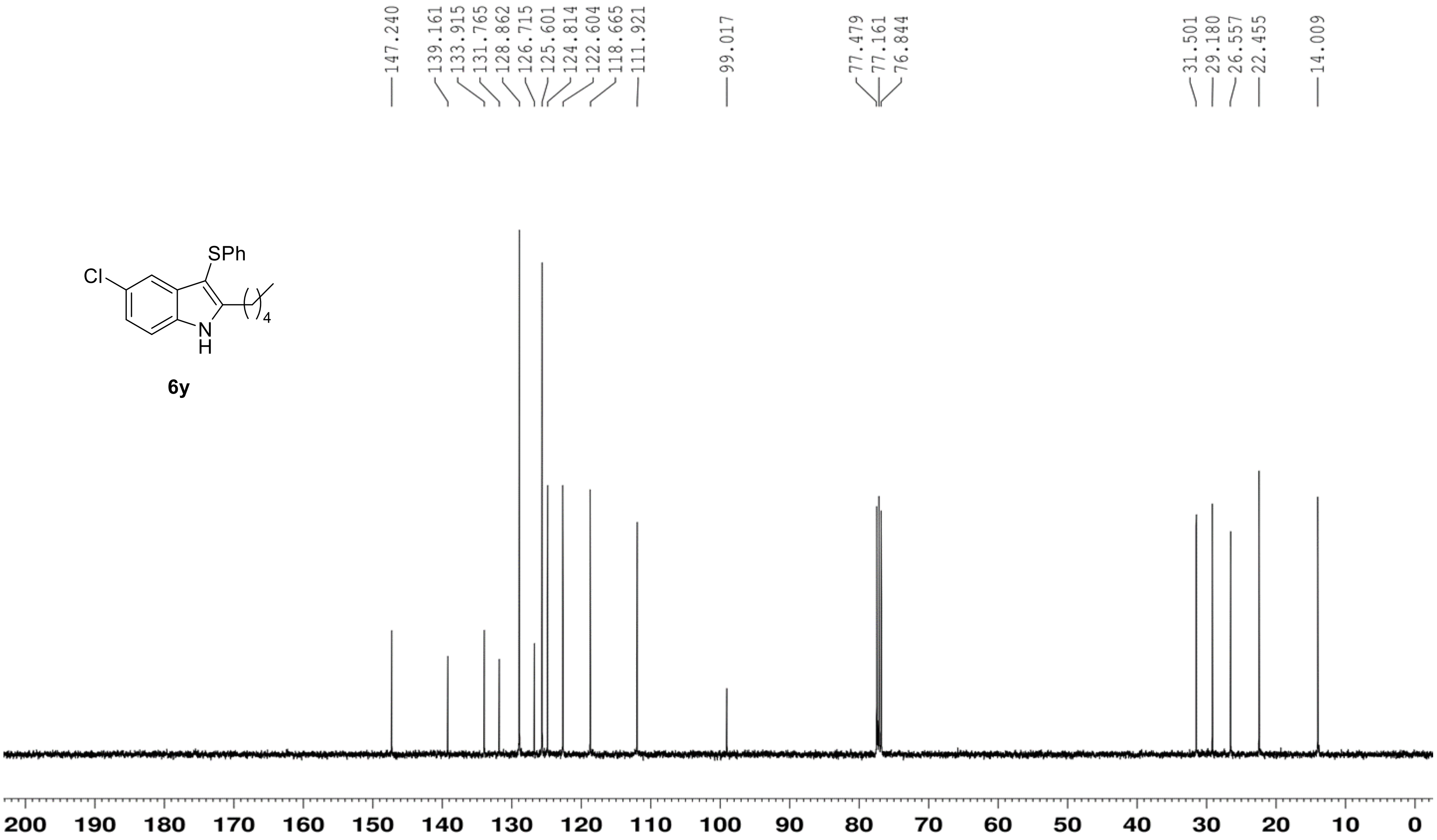

Supplementary Figure 140. ^{13}C NMR spectrum of **6y**.

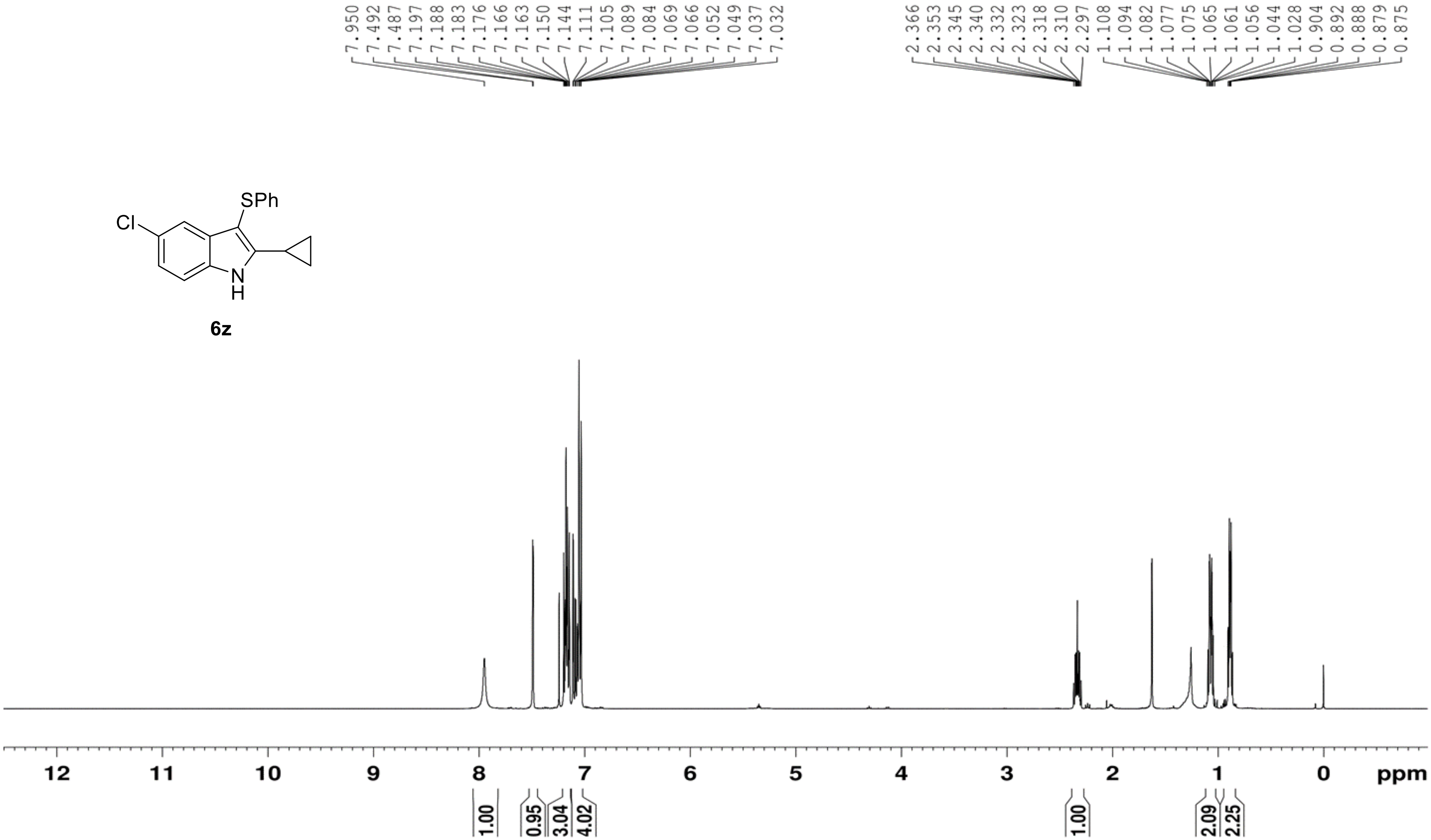

Supplementary Figure 141. ^{1}H NMR spectrum of **6z**.

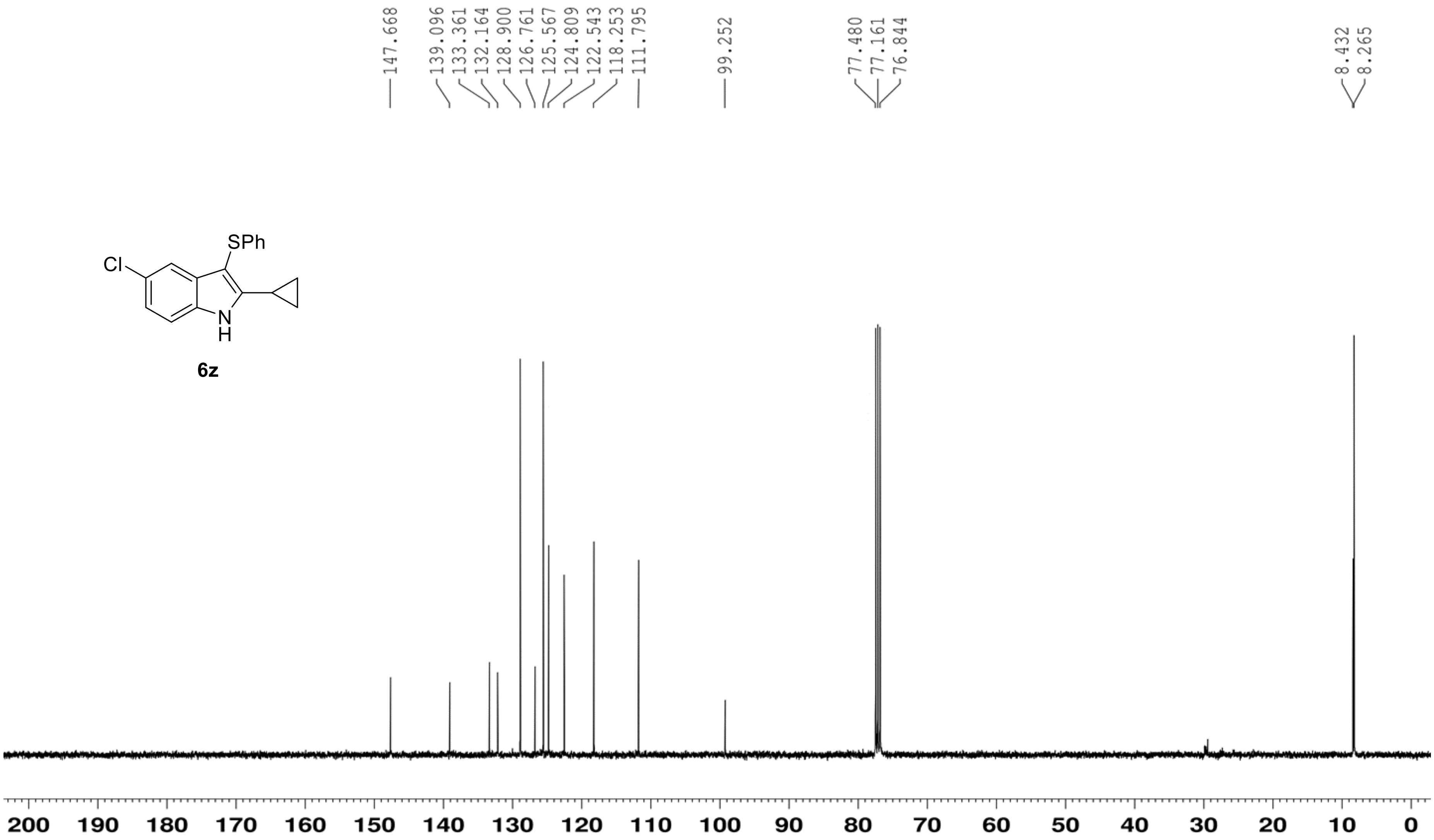

Supplementary Figure 142. ^{13}C NMR spectrum of **6z**.

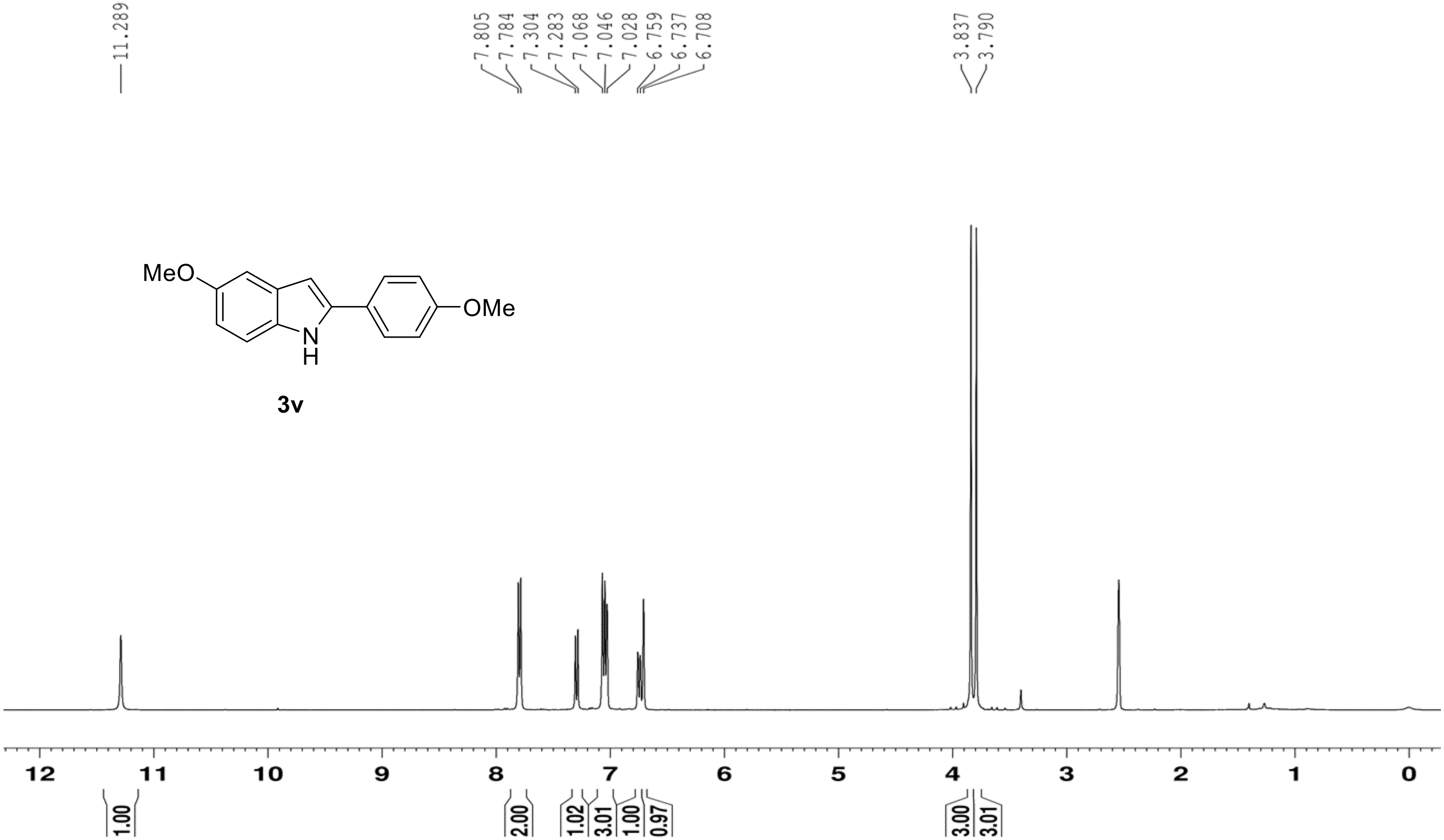

Supplementary Figure 143. ^{1}H NMR spectrum of **3v**.

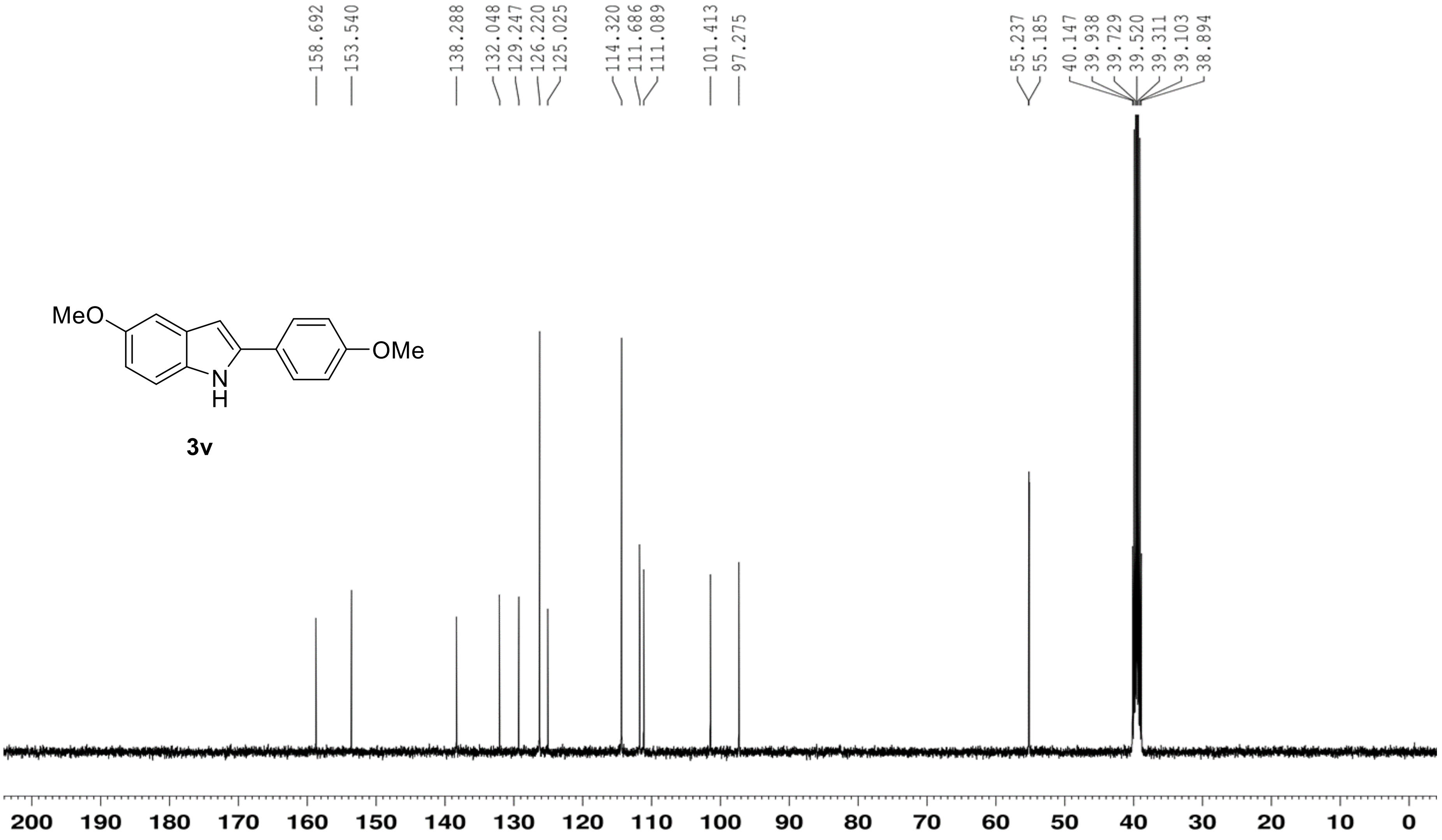

Supplementary Figure 144. ^{13}C NMR spectrum of **3v**.

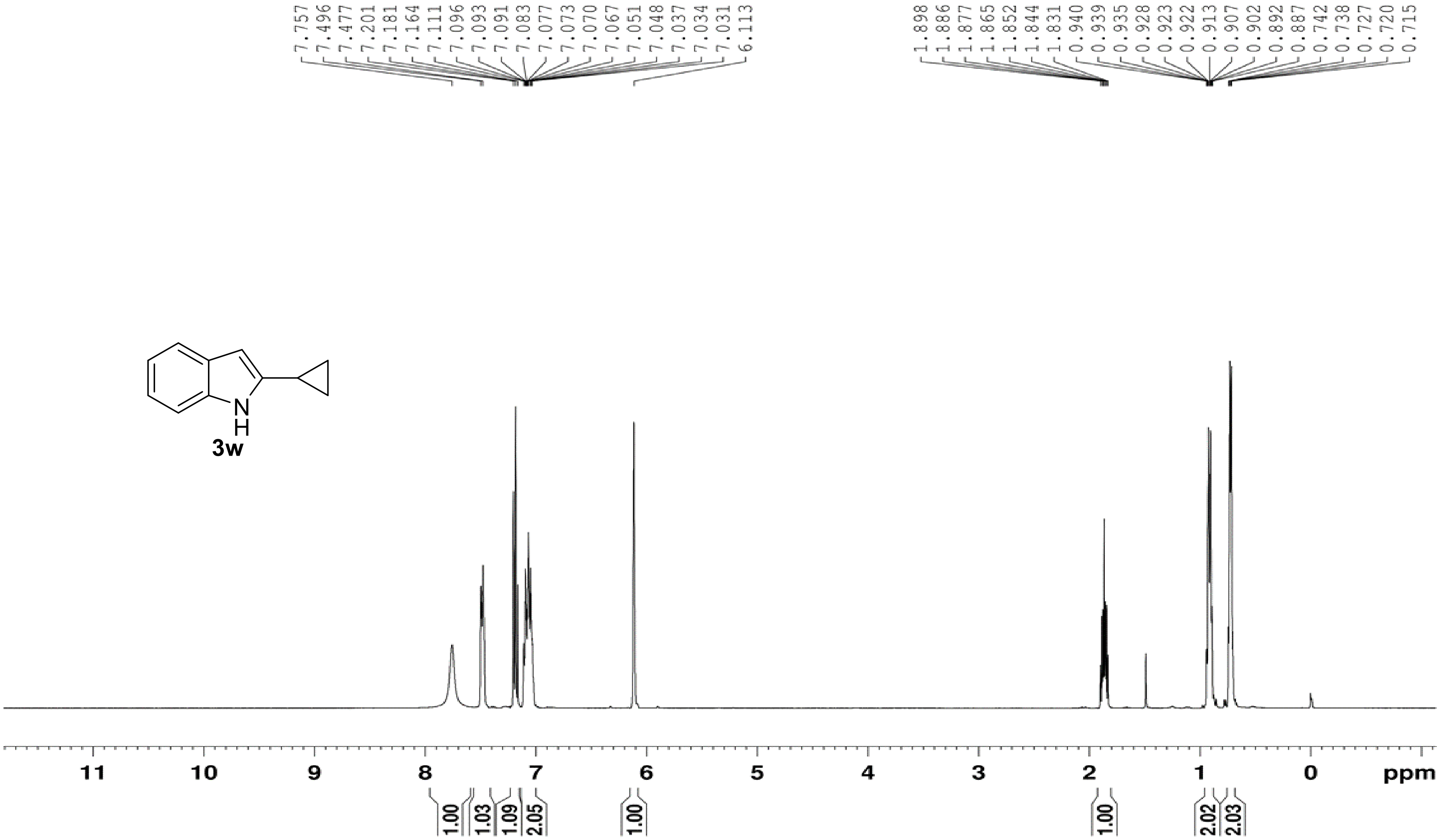

Supplementary Figure 145. ^{1}H NMR spectrum of **3w**.

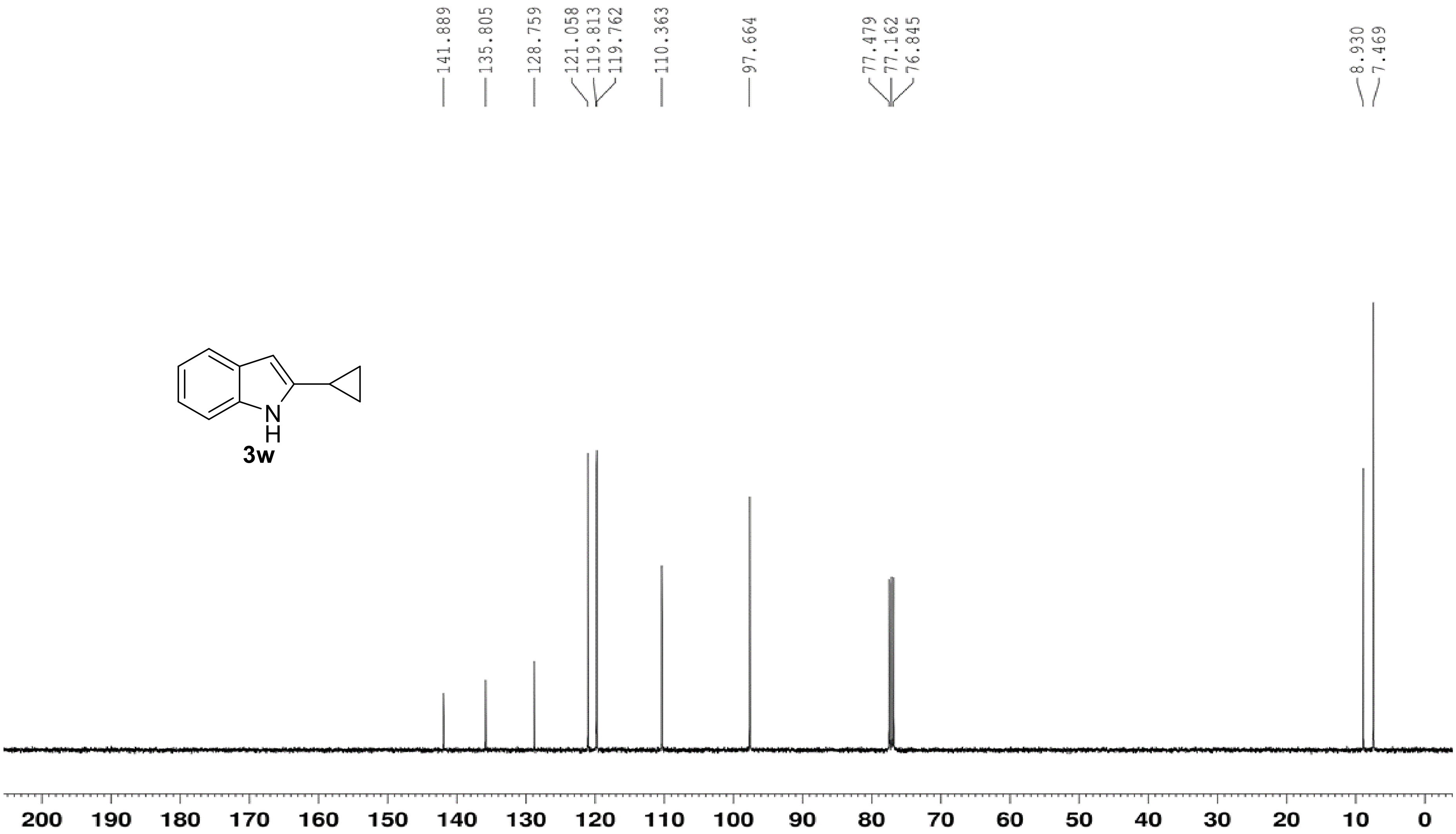

Supplementary Figure 146. ^{13}C NMR spectrum of **3w**.

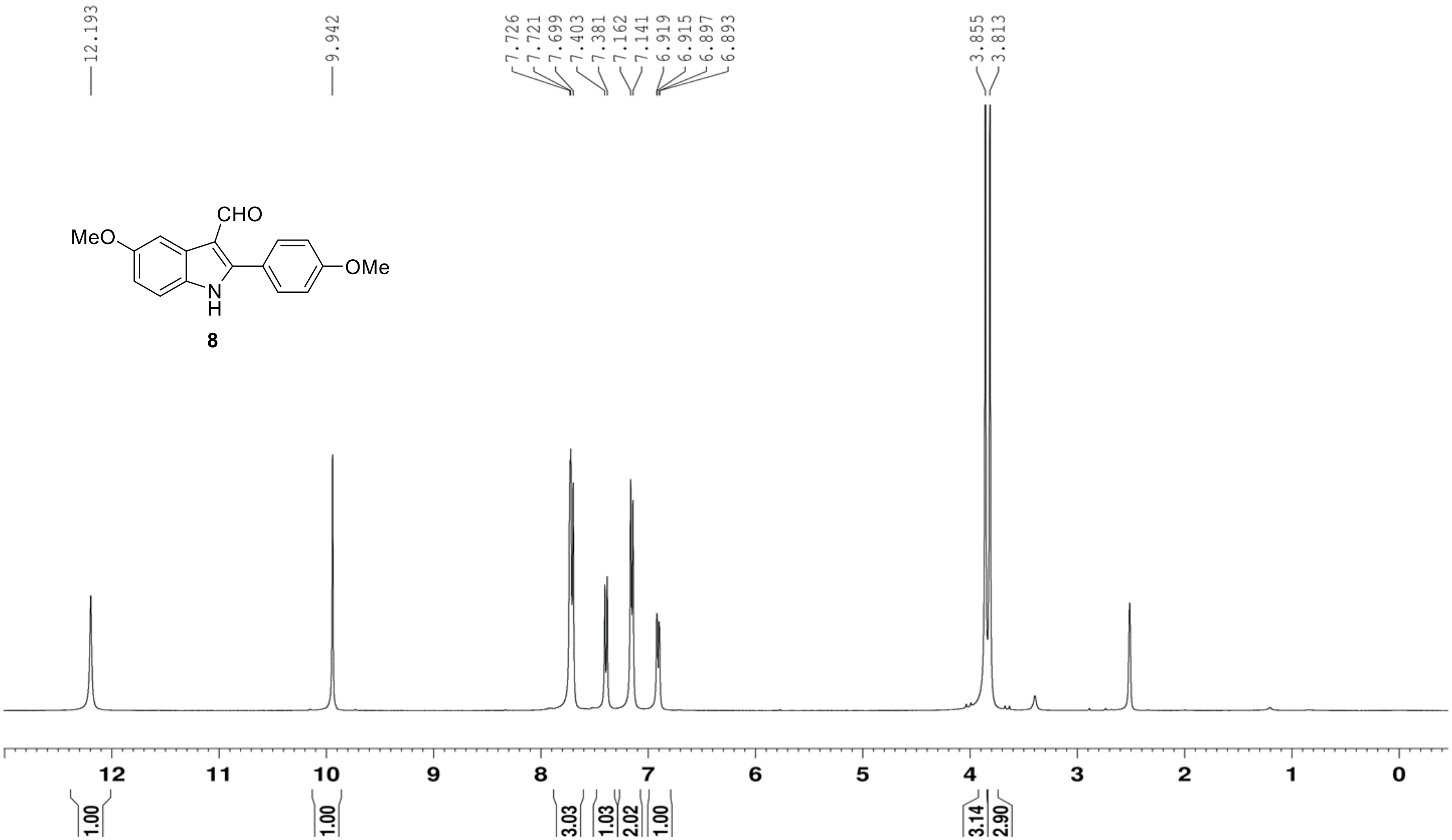

Supplementary Figure 147. ^{1}H NMR spectrum of **8**.

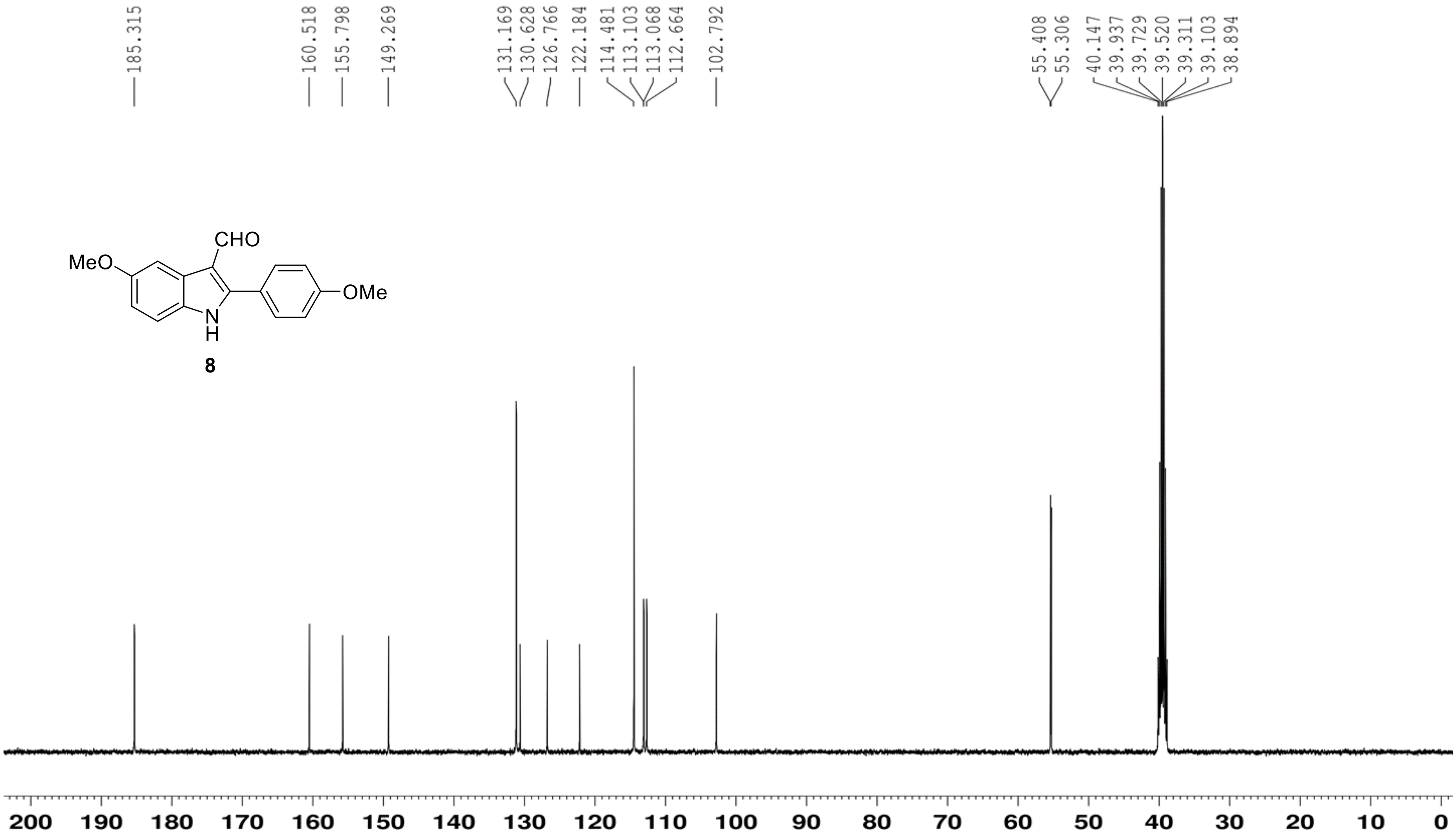

Supplementary Figure 148. ^{13}C NMR spectrum of **8**.

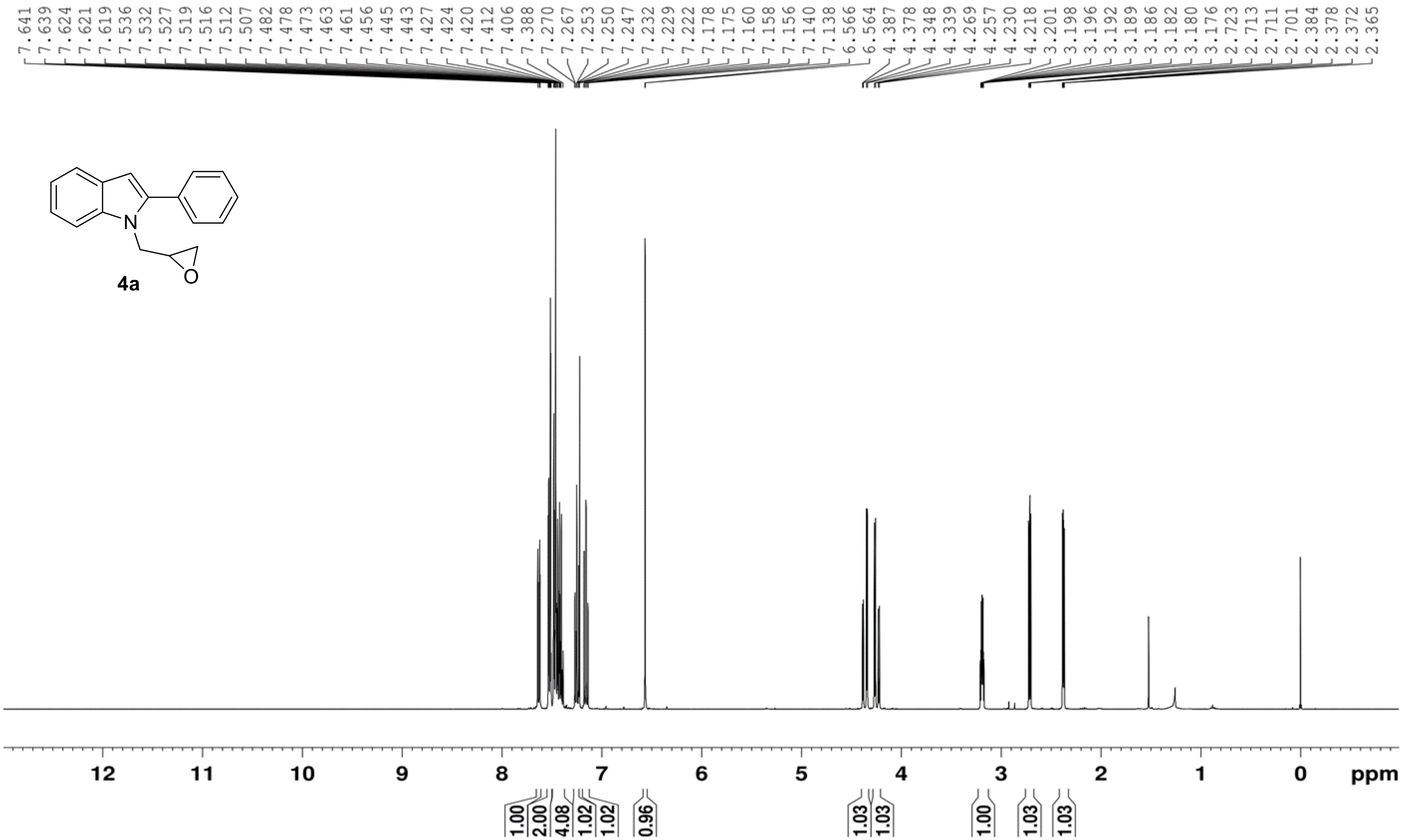

Supplementary Figure 149. ^{1}H NMR spectrum of **4a**.

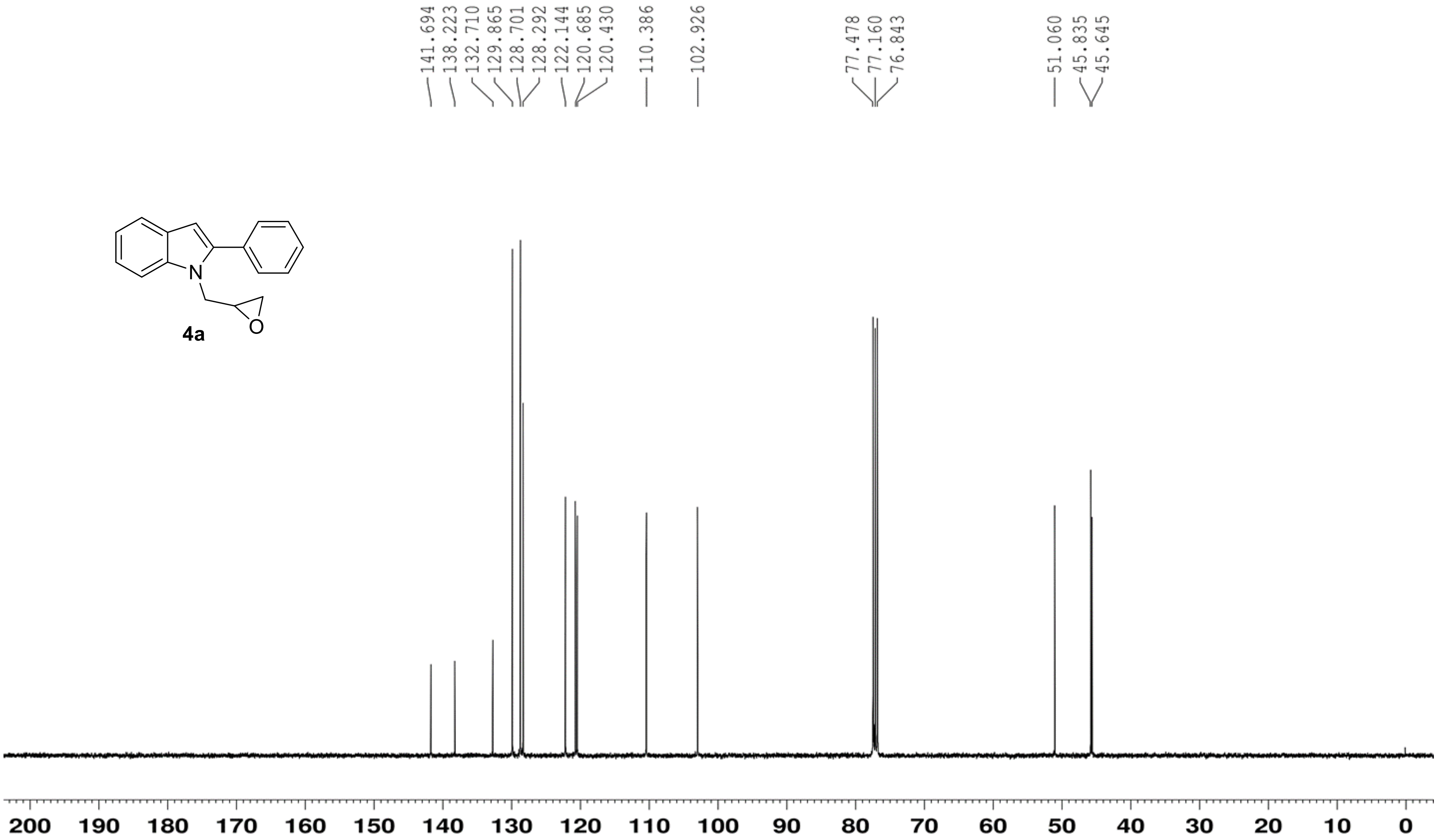

Supplementary Figure 150. ^{13}C NMR spectrum of **4a**.

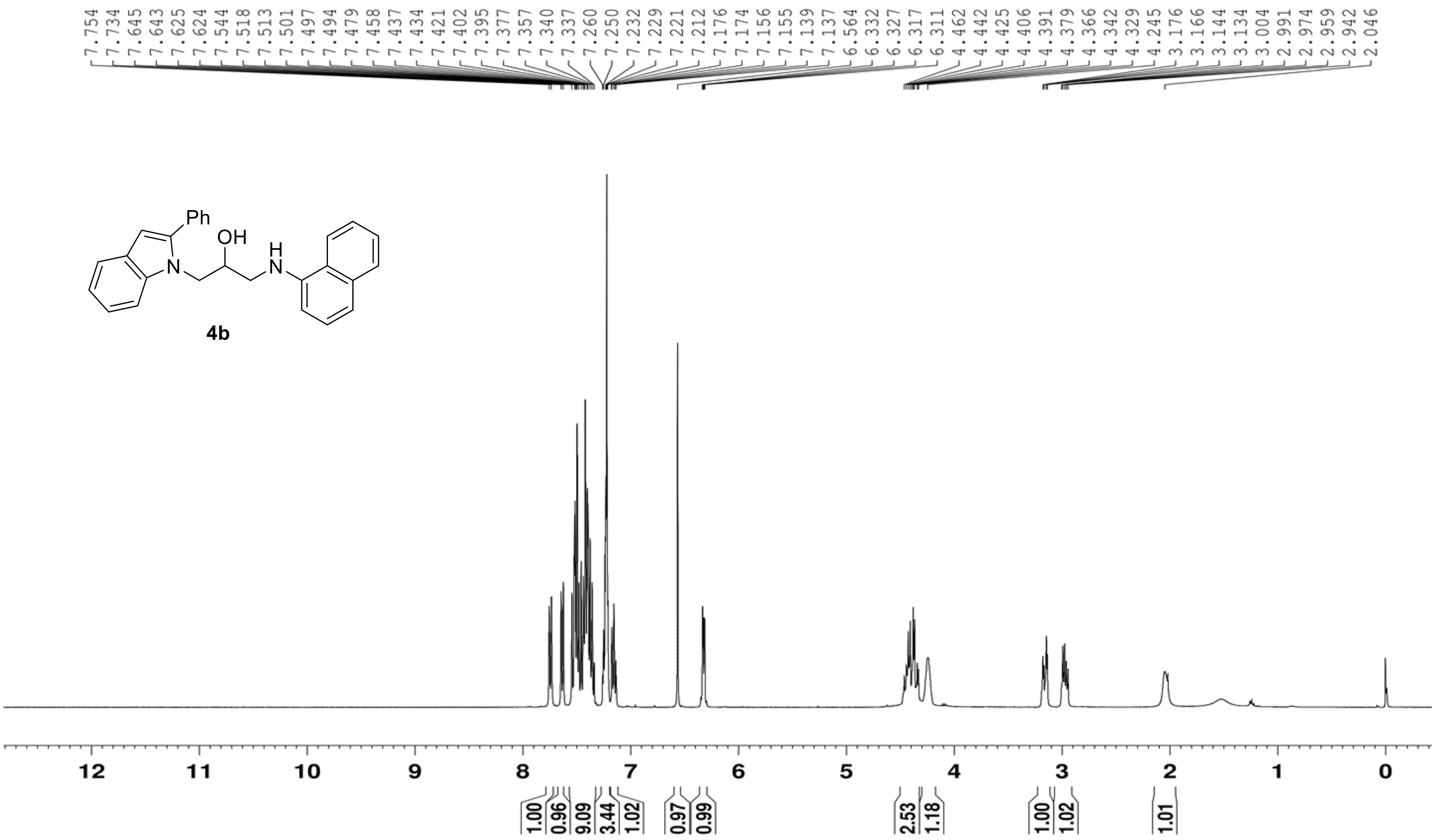

Supplementary Figure 151. ^{1}H NMR spectrum of **4b**.

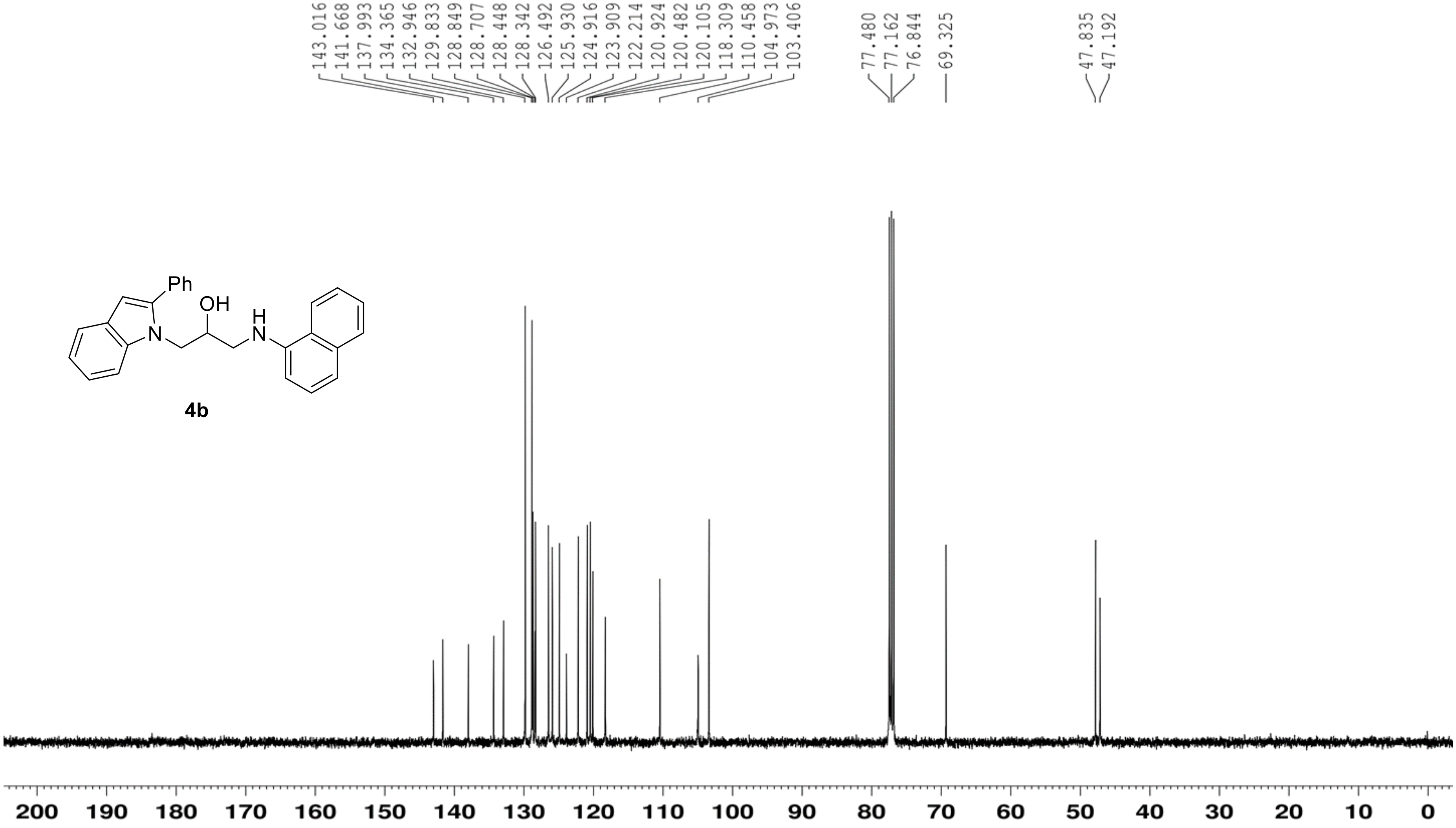

Supplementary Figure 152. ^{13}C NMR spectrum of **4b**.

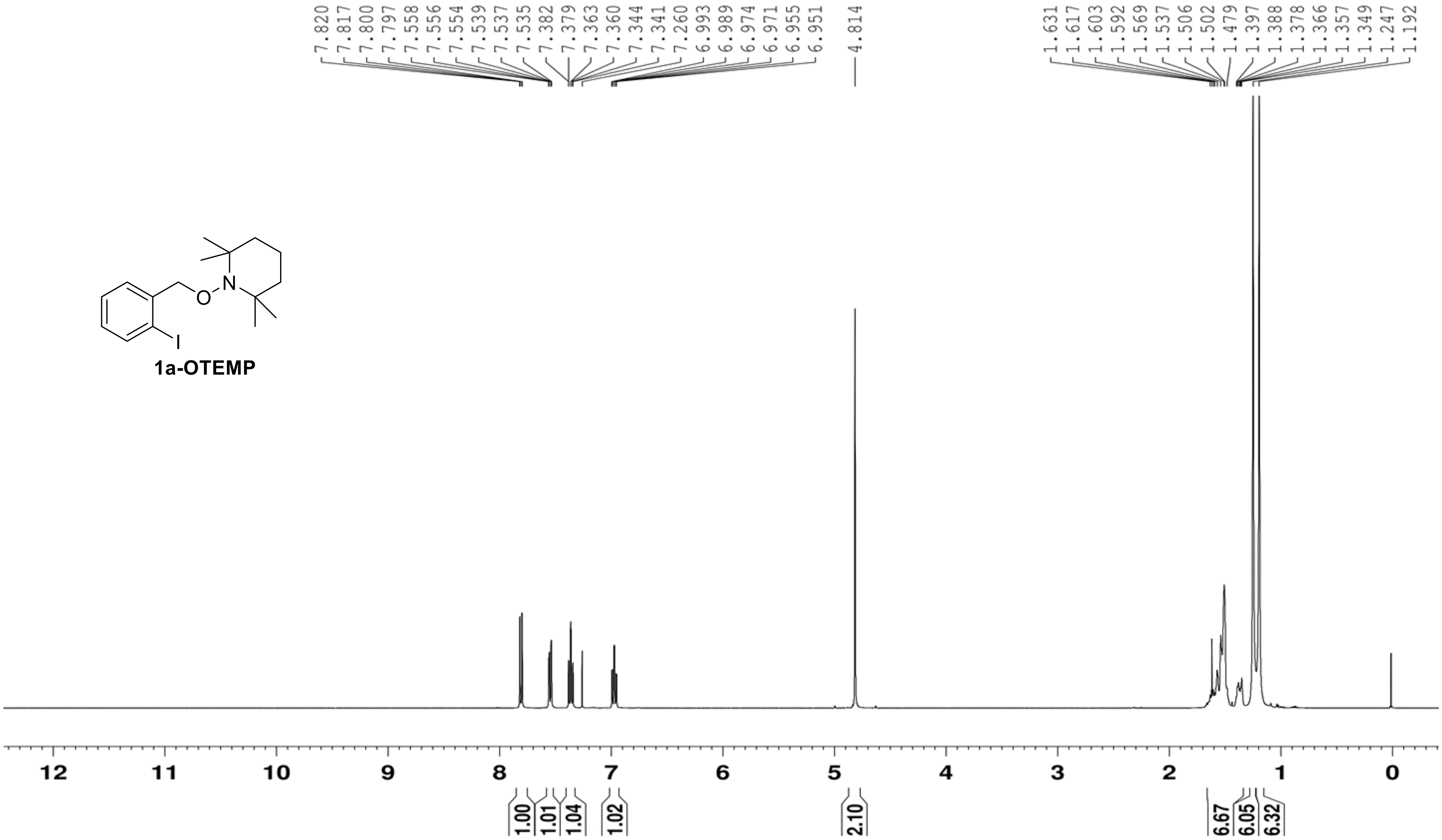

Supplementary Figure 153. ^{1}H NMR spectrum of **1a-OTEMP**.

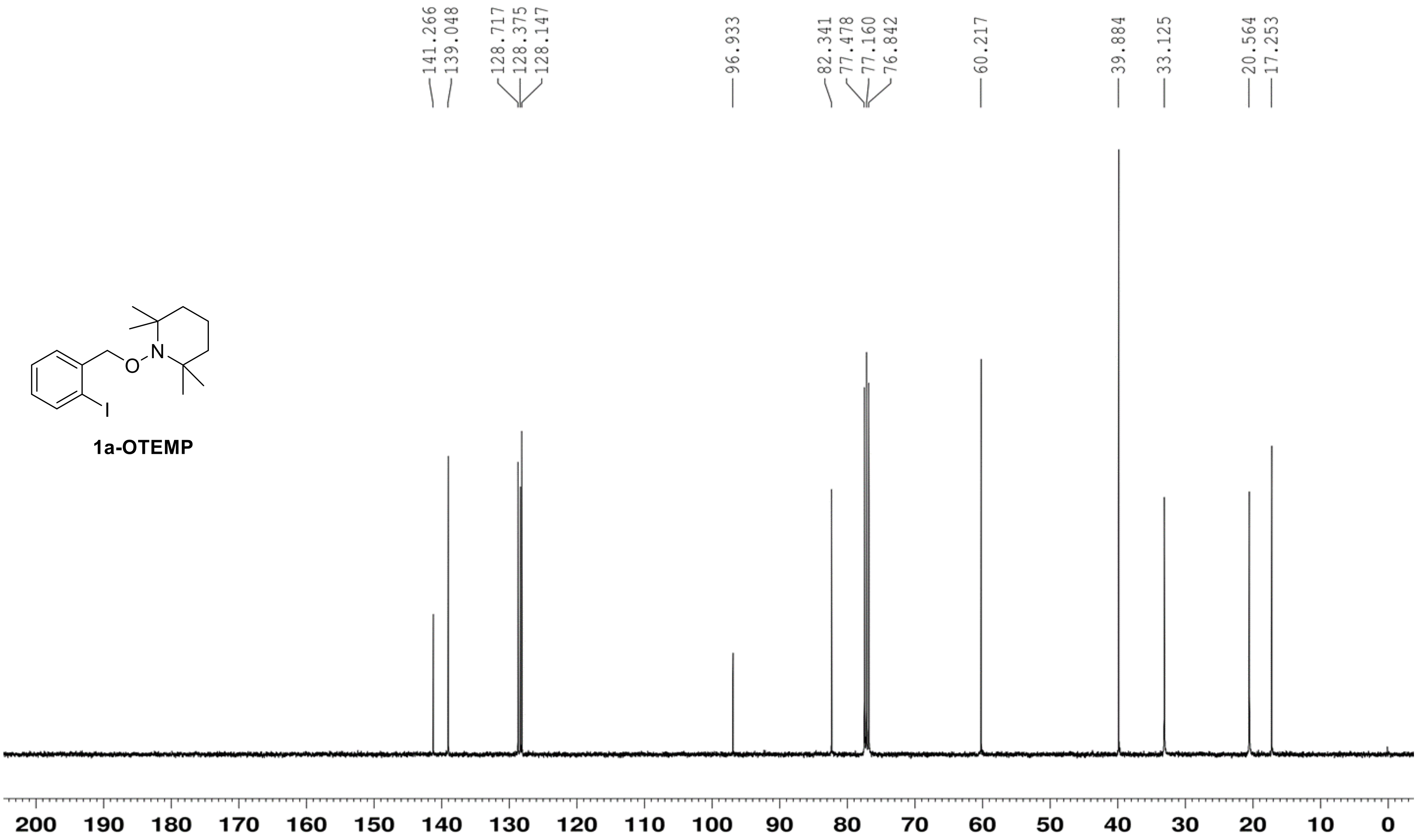

Supplementary Figure 154. ^{13}C NMR spectrum of **1a-OTEMP**.

Supplementary References

1. Zielinska, A. & Skulski, L. Eco-friendly Oxidative Iodination of Various Arenes with Sodium Percarbonate as the Oxidant. *Molecules* **10**, 1307–1317 (2005).
2. Barbero, N., SanMartin, R. & Domínguez, E. Divergent synthesis of isoindolo[2,1-a]indole and indolo[1,2-a]indole through copper-catalysed C- and N-arylations. *Tetrahedron Lett*. **50**, 2129–2131 (2009).
3. Campeau, L.-C., Thansandote, P. & Fagnou, K. High-Yielding Intramolecular Direct Arylation Reactions with Aryl Chlorides. *Org. Lett.* **7**, 1857–1860 (2005).
4. Mineno, T., Ueno, T., Urano, Y., Kojima, H. & Nagano, T. Creation of Superior Carboxyfluorescein Dyes by Blocking Donor-Excited Photoinduced Electron Transfer. *Org. Lett.* **8**, 5963–5966 (2006).
5. Ruiz, J., Ardeo, A., Ignacio, R., Sotomayor, N. & Lete E. An efficient entry to pyrrolo[1,2-b]isoquinolines and related systems through Parham cyclisation. *Tetrahedron* **61**, 3311–3324 (2005).
6. Sun, C.-L., Gu, Y.–F., Huang, W.-P. & Shi, Z.-J. Neocuproine–KOtBu promoted intramolecular cross coupling to approach fused rings. *Chem. Commun.* **47**, 9813–9815 (2011).
7. Pan, X. & Curran, D. P. Neutral Sulfur Nucleophiles: Synthesis of Thioethers and Thioesters by Substitution Reactions of N-Heterocyclic Carbene Boryl Sulfides and Thioamides. *Org. Lett.* **16**, 2728–2731 (2014).
8. Song, J., Li, Y., Sun, W., Yi, C., Wu, H., Wang, H., Ding, K., Xiao, K. & Liu, C. Efficient palladium-catalyzed C(sp2)–H activation towards the synthesis of fluorenes. *New J. Chem*. **40**, 9030–9033 (2016).
9. Mattiello, S., Rooney, M., Sanzone, A., Brazzo, P., Sassi, M. & Beverina, L. Suzuki–Miyaura Micellar Cross-Coupling in Water, at Room Temperature, and under Aerobic Atmosphere. *Org. Lett.* **19**, 654–657 (2017).
10. Xia, X.-D., Xuan, J., Wang, Q., Lu, L.-Q., Chen, J.-R., & Xiao, W.-J. Synthesis of 2-Substituted Indoles through Visible Light-Induced Photocatalytic Cyclizations of Styryl Azides. *Adv. Synth. Catal.* **356**, 2807–2812 (2014).
11. Liu, C., Ding, L., Guo, G., Liu, W., & Yang, F.-L. Palladium-catalyzed direct arylation of indoles with arylsulfonyl hydrazides. *Org. Biomol. Chem.* **14**, 2824–2827 (2016).
12. Yu, X., Park, E.-J., Kondratyuk, T. P., Pezzuto, J. M., & Sun, D. Synthesis of 2-arylindole derivatives and evaluation as nitric oxide synthase and NFκB inhibitors. *Org. Biomol. Chem.* **10**, 8835–8847 (2012).
13. Somei, M., & Yamada, K. Rearrangement Reaction of 1-Ethoxy- and 1-Hydroxy-2-phenylindole. *Heterocycles*, **84**, 785 (2012).
14. Gallou, F., Yee, N., Qiu, F., Senanayake, C., Linz, G., Schnaubelt, J., & Soyka, R. A Practical Synthesis of 2-Aryl-Indole-6-carboxylic Acids. *Synlett*, 0883–0885 (2004).
15. Yu, S., Qi, L., Hu, K., Gong, J., Cheng, T., Wang, Q., Chen, J. & Wu, H. The Development of a Palladium-Catalyzed Tandem Addition/Cyclization for the Construction of Indole Skeletons. *J. Org. Chem.* **82**, 3631–3638 (2017).
16. Gore, S., Baskaran, S., & König, B. Fischer Indole Synthesis in Low Melting Mixtures. *Org. Lett.* **14**, 4568–4571 (2012).
17. Sharma, H. A., Todd Hovey, M., & Scheidt, K. A. Azaindole synthesis through dual activation catalysis with

N-heterocyclic carbenes. *Chem. Commun.* **52**, 9283–9286 (2016).

18. Perea-Buceta, J. E., Wirtanen, T., Laukkanen, O.-V., Mäkelä, M. K., Nieger, M., Melchionna, M., Huittinen, N. Lopez-Sanchez, J. A. & Helaja, J. Cycloisomerization of 2-Alkynylanilines to Indoles Catalyzed by Carbon-Supported Gold Nanoparticles and Subsequent Homocoupling to 3,3′ -Biindoles. *Angew. Chem., Int. Ed.* **52**, 11835–11839 (2013).
19. He, L. & Li, X. W. CS and CN bond formation via Mn-promoted oxidative cascade reaction: Synthesis of C3-sulfenated indoles. *Tetrahedron* **73**, 6138-6145 (**2017**).
20. Li, J., Li, C., Yang, S., An, Y., Wu, W., & Jiang, H. Assembly of 3-Sulfenylbenzofurans and 3-Sulfenylindoles by Palladium-Catalyzed Cascade Annulation/Arylthiolation Reaction. *J. Org. Chem.* **81**, 2875–2887 (2016).
21. Tambe, S. D., Rohokale, R. S., & Kshirsagar, U. A. Visible-Light-Mediated Eosin Y Photoredox-Catalyzed Vicinal Thioamination of Alkynes: Radical Cascade Annulation Strategy for 2-Substituted-3-sulfenylindoles. *Eur. J. Org. Chem.* **2018**, 2117–2121 (2018).
22. Geary, L. M., & Hultin, P. G. Modular Construction of 2-Substituted Benzo[b]furans from 1,2-Dichlorovinyl Ethers. *Org. Lett.* **11**, 5478–5481 (2009).
23. Fu, H., Qiao, R., Jiang, Y., Zhao, Y., & Jiang, H. Palladium-Free Copper-Catalyzed Sonogashira Cross-Coupling at Room Temperature. *Synthesis,* **2008**, 2417–2426 (2008).
