# Supplementary material for: Copper-catalyzed oxidative benzylic C-H cyclization via iminyl radical from intermolecular anion-radical redox relay
Source: Nat Commun. 2019 Feb 22;10:908. doi: 10.1038/s41467-019-08849-z (PMC6385223; doi:10.1038/s41467-019-08849-z)
Supplement: Supplementary file 2 — Peer Review File [file 41467_2019_8849_MOESM2_ESM.pdf]

Reviewers' comments:

Reviewer #1 (Remarks to the Author):

The manuscript by Kang and coworkers reports on the indole synthesis ortho-iodotoluene and related compounds and nitriles in the presence of KOtBu and CuSO4 catalyst. The reaction is typically achieved using 2 mol% CuSO4 and excess (4 equiv) KOtBu in octane at 60-100 °C. The reaction is applicable to the synthesis of 2-arylindoles (Fig 3), 2-aryl-3-substituted indoles with phenyl, indolyl, phenoxy, or sulfenyl group on the 3-position (Fig 5), and 2-alkyl-3-sulfenyl indoles (Fig 6). Gram-scale syntheses of pharmaceutically relevant compounds are also presented (Fig 4 and 7). The reaction is considered to proceed through deprotonation of benzylic C-H with KOtBu, oxidation of the resulting carbanion with Cu(II) to a benzylic radical, addition of the radical to the nitrile, and intramolecular cyclization of the resulting iminyl radical, where Cu(I) is reoxidized to Cu(II). Overall, the reported reactions may serve as useful methods for the synthesis of some classes of indole derivatives. That said, there are some concerns about suitability of this manuscript for publication in Nature Communications, as described below.

1) The reaction is not totally atom-economical, as it requires a large excess of nitrile (5 equiv) with respect to the iodotoluene derivative. This would be a serious problem if one were to use a precious nitrile for the indole synthesis. By the way, there is not much point in indicating the leaving group as "X" in Fig 3, 5, and 6, because for most cases iodide substrates are used with only a few exceptions.

2) Many of the indole products presented do not seem particularly challenging to synthesized. For example, 2-arylindoles can of course be synthesized by the classical Fischer indole synthesis. Speaking of more recent methods, they can be accessed by C2-arylation of indoles, or dehydrogenative cyclization of N-aryl imines prepared by aniline-acetophenone condensation (ref 38). The latter method may be more economical and versatile when it comes to the synthesis of 2-arylindoles bearing substituents on the benzene moiety of the indole core. This is because substituted ortho-iodotoluenes may not be as broadly available as substituted anilines. Also, 2-aryl-3-sulfenylindoles, another highlighted class of indoles in this work, can be efficiently synthesized by 3-sulfenylation of 2-arylindoles (e.g., ref 74).

3) Relating to the above argument, the demonstrations of the "utility" of the present reaction in Fig 4 and 7 may not appear very impressive. The synthesis of BACE1 inhibitor in Fig 4 is based on routine transformation of 2-phenylindole, the synthesis of which is already well established and is possible by many different means including more atom-economical ones. For Fig 7, the reviewer simply does not understand why the compound 8 had to be synthesized by way of desulfenylation of the sulfenylated indole 6f rather than directly from the 2-arylindole 3r, which can be synthesized by the present method (Fig 3) as well as by other means.

The reviewer considers the present reaction involves mechanistically interesting aspects such as anion-to-radical conversion and formation of iminyl radical through radical addition to nitrile. However, the reviewer is afraid that the practicality and the scope of the reaction may not strongly appeal to the audience of Nature Communications.

Reviewer #2 (Remarks to the Author):

Efficient synthesis of indole derivatives is an interesting and important research topic in synthetic organic chemistry. The authors describe radical cyclization reactions of ortho-halotoluenes and nitriles in the presence of catalytic amounts of a Cu(II) salt and 1 eq. of KOtBu. The reactions proceeded to afford the desired indole derivatives in moderate to high yields. The authors insist that this is the first example of a catalytic anion-radical redox relay without any oxidant. The authors also show good

substrate scope. I think that this reaction is interesting and valuable for the synthesis of indole derivatives. However, while the authors investigate utility of this reaction, mechanistic consideration about the catalysis seems to be poor. For example, information of the active copper species is not provided in this manuscript. In Table 1, some Cu(I) species also promoted the reaction. I wonder what is the real active Cu species. In the reaction system, CuSO4 can react with KOtBu to form Cu(OtBu)2, and electron rich Cu(II) species is sometimes not so stable. The authors should provide information about the active Cu species. In the substrate scope, availability of other ortho-alkyl-halobenzenes such as ortho-ethyl-iodobenzene and ortho-alkylphenols protected and activated by Tf group is interesting. The authors should show possibility of those substrates.
After addressing the above issues, this article may be accepted for publication in Nat. Commun.

Reviewer #3 (Remarks to the Author):

The manuscript by Kang and coworkers reports on a copper-catalysed indole formation from toluenes and nitriles comprising a sequential C-C and C-N bond instalment. The authors propose a radical relay mechanism, which based on their control experiments from Figures 2B-D appears reasonable. In principle, one would anticipate that the radical relay occurs at the stage of the iminyl radical. Its reduction by cuprous salt would generate an iminyl anion, which would undergo copper-promoted C-N coupling following extensive work, as for example by Buchwald. The authors exclude such a possibility pointing to the outcome of their control experiment from Figure 2D. I agree with this conclusion, but maybe it would provide a better understanding if they discussed this important mechanistic context in more detail. This negative outcome on Ullmann coupling is surprising, but may be owed to the absence of any diamine ligands in the catalyst.
The major problem with this work is the clear limitation it presents due to the required strong basic conditions (usually, 4 equivalents of KOtBu). This limits the toluene component to the category of non-base-labile substituents and the nitrile component to benzonitriles. Such a limitation is severe from the synthetic standpoint. Nevertheless, the authors have provided a long list of successful examples in Figures 3, 5 and 6. Some preliminary application (Figures 4 and 7) is also provided. With respect to the latter Figure 7, it appears surprising that this synthesis is carried out with a sulfide placeholder at the benzylic position. Based on the successful example of 3m, why is it not possible to use the corresponding 3-methyl, 4-iodoanisole instead? This would reduce the synthesis of 8 to two steps.
In summary, this manuscript reports a potentially useful synthetic procedure for indole synthesis. However, it requires major revision as the authors need to explore and address the issue of base labile functional groups prior to publication.
The title should be shortened by removal of the last three words.
As to another problem, the manuscript contains a large number of typos and grammar mistakes that need to be corrected.
Some examples on page 3 include:
…uneasy to be achieved… should be: …difficult to generate…
The yields seems… should be: The yields seem…
Figure 6: Reaction conditions (not Reaction Condition)
…reagent 8 is synthesized though 3 steps… should be: …reagent 8 is synthesized through 3 steps…
..and as alow as… should be: … and as slow as…
…imynyl radials… shoud be: ….iminyl radicals…

Reviewer #1

The manuscript by Kang and coworkers reports on the indole synthesis ortho-iodotoluene and related compounds and nitriles in the presence of KOtBu and $CuSO_4$ catalyst. The reaction is typically achieved using 2 mol% $CuSO_4$ and excess (4 equiv) KOtBu in octane at 60-100 °C. The reaction is applicable to the synthesis of 2-arylindoles (Fig 3), 2-aryl-3-substituted indoles with phenyl, indolyl, phenoxy, or sulfenyl group on the 3-position (Fig 5), and 2-alkyl-3-sulfenyl indoles (Fig 6). Gram-scale syntheses of pharmaceutically relevant compounds are also presented (Fig 4 and 7). The reaction is considered to proceed through deprotonation of benzylic C-H with KOtBu, oxidation of the resulting carbanion with Cu(II) to a benzylic radical, addition of the radical to the nitrile, and intramolecular cyclization of the resulting iminyl radical, where Cu(I) is reoxidized to Cu(II). ***Overall, the reported reactions may serve as useful methods for the synthesis of some classes of indole derivatives. That said, there are some concerns about suitability of this manuscript for publication in Nature Communications,*** as described below.

1) The reaction is not totally atom-economical, as it requires a large excess of nitrile (5 equiv) with respect to the iodotoluene derivative. This would be a serious problem if one were to use a precious nitrile for the indole synthesis.

[Reply]: Thanks for reminding. The excess nitrile starting materials can be recovered quantitatively. For example, in the reaction below, 4.1 out of 5 equiv of nitrile has been recovered when 85% yield of product was obtained. That is to say, almost quantitative unreacted nitrile could be recovered. Moreover, the amount of nitrile can be further lowered down to 1.5 equiv for the reaction of **5** and **3**. As shown below, the reaction with 1.5 equiv of nitrile affords corresponding indole **6d** in 85%. We finally choose 2.5 equiv of base and nitrile for the optimized conditions.

This issue has been mentioned in the manuscript in Tables 1-2 and marked in yellow.

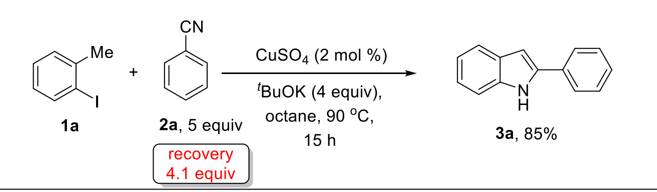

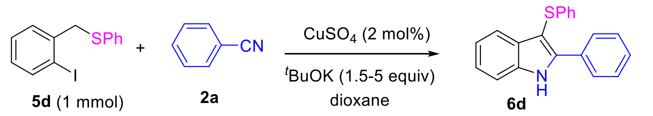

PhCN (equiv)	^{t}BuOK (equiv)	t (h)	temp.(°C)	6d (%)
5	4	11	60	93
2.5	2.5	24	90	92
2	2	36	90	75
2	3	24	90	91
1.5	3	24	90	85

C3-substituted indoles can also be synthesized by this method. Reaction conditions were further optimized on the basis of Table 1 (Table 2). Despite of the fact that even 1.5 equiv of nitrile affords 85% of 6d (entry 5), the conditions listed in entry 2 were chosen for the balance of yields and amounts of base and nitriles. Indoles

Table 2. Reaction conditions for the synthesis of 6.

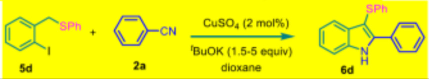

Entry[a]	PhCN (equiv)	^{t}BuOK (equiv)	t (h)	6d (%)
1[b]	5	4	11	93
2	2.5	2.5	24	92
3	2	2	36	75
4	2	3	24	91
5	1.5	3	24	85

[a] Conditions: **5d** (1 mmol), **2a** (1.5-5 mmol), $CuSO_4$ (2 mol %), ^{t}BuOK (2-5 mmol), dioxane (0.75 mL), argon. Yields were determined by ^{1}H NMR. [b] At 60 °C.

By the way, there is not much point in indicating the leaving group as "X" in Fig 3, 5, and 6, because for most cases iodide substrates are used with only a few exceptions.

[Reply]: Thanks for suggestion. We have included 14 examples for Br- and Cl-based substrates into Fig. 3 and 13 examples for Br- and Cl-based substrates in Figure 5 in the revsed version. In Fig. 6, X has been changed to I. The copied of the revised Figures 3-6 are sown below:

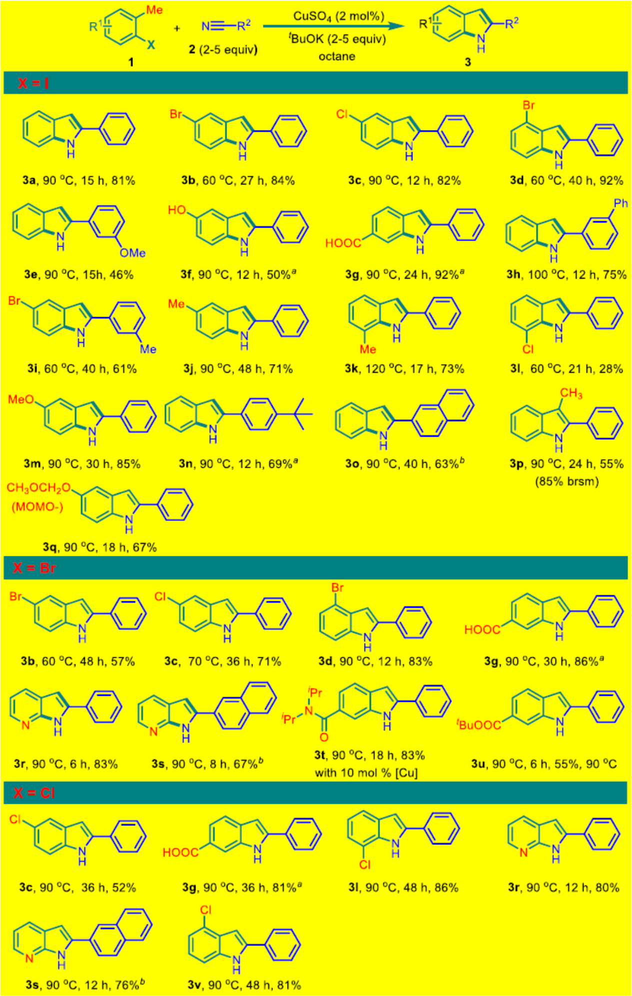

Fig. 3. Scope of methyl (het)arenes and nitriles. Reaction conditions: Conditions: $CuSO_4$ (2 mol%), **1** (1 mmol), **2** (5 mmol), ^tBuOK (5 mmol), octane (0.75 mL). Extra 1 mmol ^tBuOK was used for **3f** and **3g**. [a] **2** (1.5 mL) was used as solvent. [b] Octane (3 mL). Brsm yield refers to the yield based on recovered starting material.

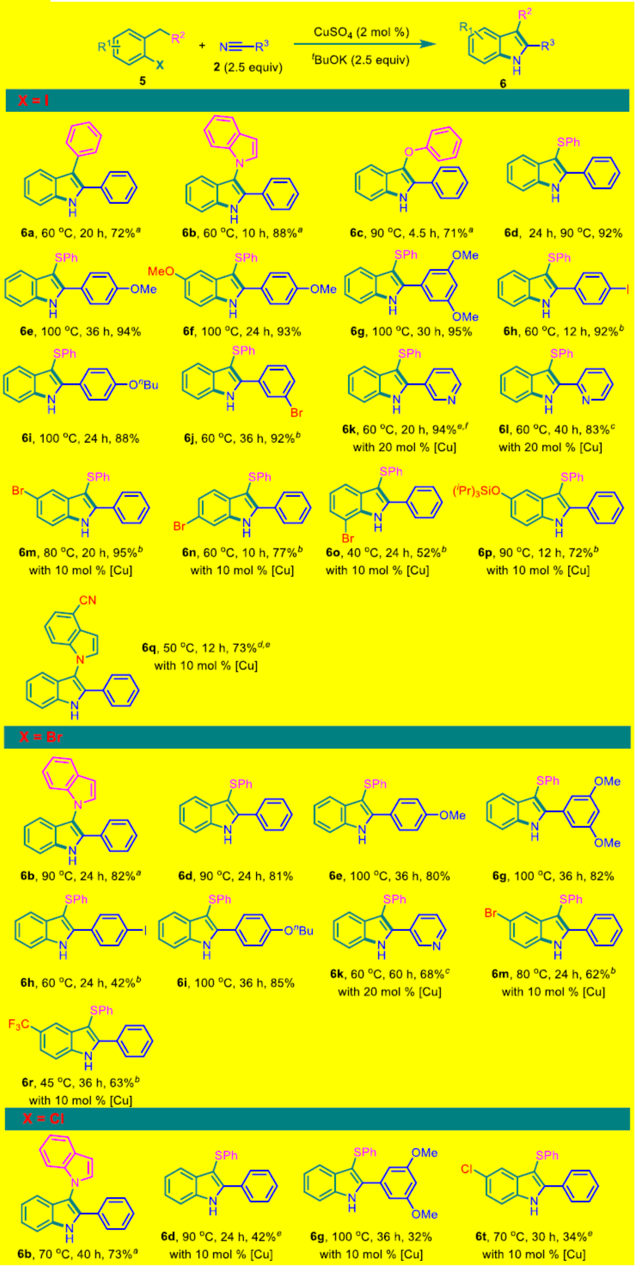

Fig. 5. Scope of C3-functionalized indoles. Reaction conditions: **5** (1 mmol), **2** (2.5 mmol), $CuSO_4$ (2 mol %), ^tBuOK (2.5 mmol), dioxane (0.75 mL). [a] **2** (5 mmol), ^tBuOK (4 mmol), octane (0.75 mL) instead of dioxane. [b] Dioxane (1.5 mL). [c] Dioxane (20 mL). [d] ^tBuOK (4 mmol). [e] **2** (1.5 mL) was used as solvent. [f] **2** (15 mL) was used as solvent.

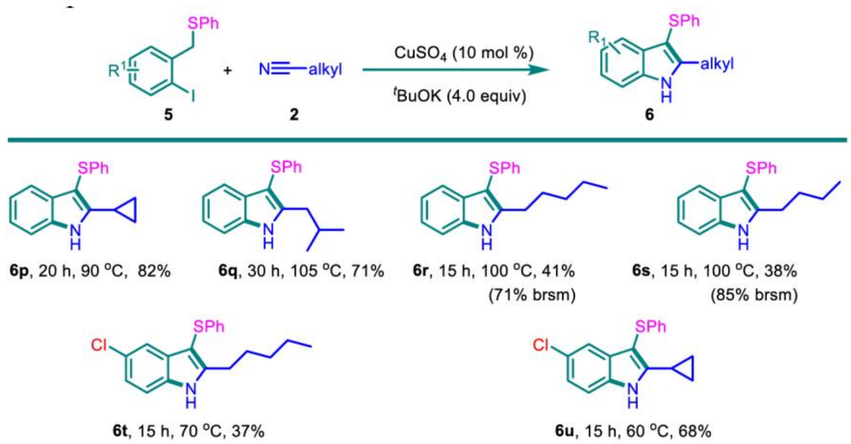

Fig. 6. Scope of C2-alkyl indoles. Reaction Condition: **5** (1 mmol), **2** (2 mL), $CuSO_4$ (10 mol %), ^{t}BuOK (4 mmol), no extra solvent. Brsm yield refers to the yield based on recovered starting material.

2) Many of the indole products presented do not seem particularly challenging to synthesized. For example, 2-arylindoles can of course be synthesized by the classical Fischer indole synthesis. Speaking of more recent methods, they can be accessed by C2-arylation of indoles, or dehydrogenative cyclization of N-aryl imines prepared by aniline-acetophenone condensation (ref 38).

The latter method may be more economical and versatile when it comes to the synthesis of 2-arylindoles bearing substituents on the benzene moiety of the indole core. This is because substituted ortho-iodotoluenes may not be as broadly available as substituted anilines.

Also, 2-aryl-3-sulfenylindoles, another highlighted class of indoles in this work, can be efficiently synthesized by 3-sulfenylation of 2-arylindoles (e.g., ref 74).

[Reply]: Thanks for the comment.

In fact, speaking about the methods for the synthesis of indoles, there are numerous well-established methods. Even so, there are still numerous reports on the new synthetic methods for indoles. The reason is that no method can match all needs of organic synthesis, even with the typical Fischer indoles synthesis. One big challenge is the construction of C4-C7 substituents on the indole backbone. The cross-coupling methods have proven to be a powerful method for the modification of indoles, but the indole backbone has to be pre-constructed. Besides, not all the substituted indoles can be furnished by the C2-funcationalization. For example, C2-n-alkylindoles are not accessible from the method reported in ref. 38 (see the copy below), while current method could provide both C2-alkyl (2 steps) and C2-aryl indoles. Another example is that C4-substituted indoles cannot be achieved by this C2-arylation method due the high steric hindrance, only C7- regioisomer could be obtained (see the copy below). In addition, the utilizing 10 mol% palladium catalyst has already flawed its application in organic synthesis. As a conclusion, chemists need versatile methods to solve complex synthetic problems other than just count on one or two limited methods.

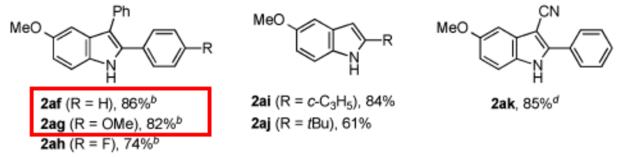

[a]NR'$_2$ is a morpholino group. [b]Cu(OAc)$_2$ was used as the oxidant (conditions in Table 1, entry 8). [c]Regioisomeric product was obtained in 7% yield. [d]Starting material was in the form of enamine.

yield, which was accompanied by a ketone byproduct (33%) arising from benzylic oxidation.[18] However, by using Cu(OAc)$_2$ as the oxidant, the yield of **2af** was improved to 86% with a complete suppression of benzylic oxidation. Similarly, 2,3-diarylindoles **2ag** and **2ah** were obtained in good yields. These examples demonstrate the utility of the present cyclization for regiocontrolled synthesis of 2,3-diarylindoles, which is difficult with the Larock- and Fagnou-type annulation reactions using diarylalkynes.[2,7] 2-Cyclopropyl- and 2-*tert*-butylindoles **2ai** and **2aj** were also obtained in good yields from the corresponding imines, while attempts to synthesize 2-*n*-alkylindoles have not been successful. In addition to these examples, the present

Table 2. Indole Synthesis from *N*-Aryl Imines

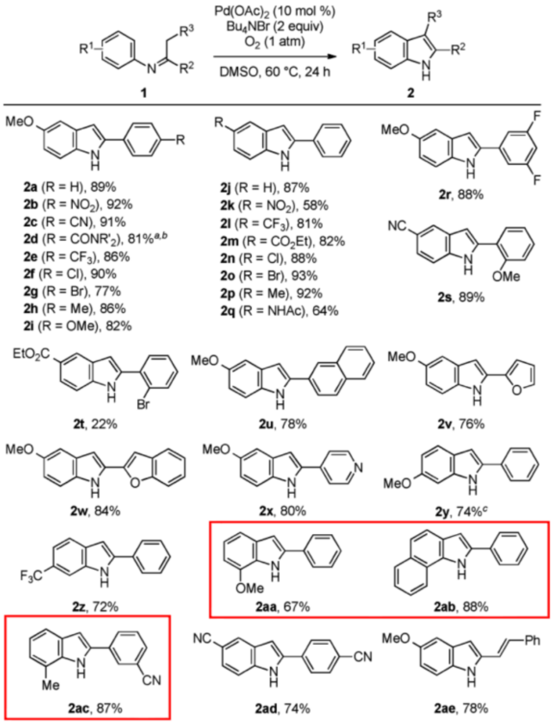

Speaking about economic issue, to be honest, the using of 10 mol% of palladium catalyst can hardly make a synthetic method "more economical". However, our method does not need noble metal or ligands. We use naturally abundant salt $CuSO_4$ generally in a very low amount of 2 mol%. The price of palladium and copper salts can make a remarkable proof. Considering the low catalyst loading as well as the low cost of such base metal catalyst (2 mol% **$CuSO_4$ [>99.99%, 63.4 USD/10g]** vs 10 mol% **$Pd(OAc)_2$ [>99.9%, 268 USD/5g]**), therefor it is of sufficient sustainability.

With respect of 2-aryl-3-sulfenylindoles, the method in ref. 74 is a typical C3-functionalization of indoles. This type of reactions must be based on the exiting indole backbones. Forgeting about the limited scope for C2, C4-C7 substitutions, similar problems will meet as what mentioned above on the C2-fuctionalization of indoles. In our work, a large variety of C2, C4-C7 substituted indoles can be smoothly obtained even in large scale, without the involvement of noble metal catalysts or the reagent with disagreeable odour.

3) Relating to the above argument, the demonstrations of the "utility" of the present reaction in Fig 4 and 7 may not appear very impressive. The synthesis of BACE1 inhibitor in Fig 4 is based on routine transformation of 2-phenylindole, the synthesis of which is already well established and is possible by many different means including more atom-economical ones. For Fig 7, the reviewer simply does not understand why the compound 8 had to be synthesized by way of desulfenylation of the sulfenylated indole 6f rather than directly from the 2-arylindole 3r, which can be synthesized by the present method (Fig 3) as well as by other means.

[Reply]: Thanks for the comment!

In the original manuscript, we have demostrated that the direct synthesis of key intermediate indole 3w (originally 3r) from 2-iodo-toluene needs two steps.

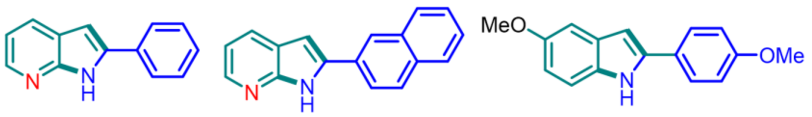

3p, 6 h, 83% X = Br, 90 °C | **3q**, 8 h, 67%[b] X = Br, 90 °C | **3r**, 15 h, 86%[c] X = I, 100 °C

Fig. 3. Scope of methyl (het)arenes and nitriles. Reaction conditions: Conditions: **1** (1 mmol), **2** (5 mmol) $CuSO_4$ (2 mol %), ^{t}BuOK (4 mmol), octane (0.75 mL). Extra 1 mmol ^{t}BuOK was used for **3f** and **3g**. [a] 2 (1.5 mL) was used as solvent. [b] Octane (3 mL). [c] **3r** was obtained from **6f** and PhS was removed by treating with 2-mercaptobenzoic acid and CF_3CO_2H at RT for 12 h.

To make this question more clear, we have performed the comparison experiments as shown below, indoles **3a** and **3b** can both be obtained from **5** or **1**, whereas **3w** and **3x** could not. This is why route 2 was applied in the synthesis of **8**. This issue has been clarified by the insertion of Fig. 7 (see the copy below).

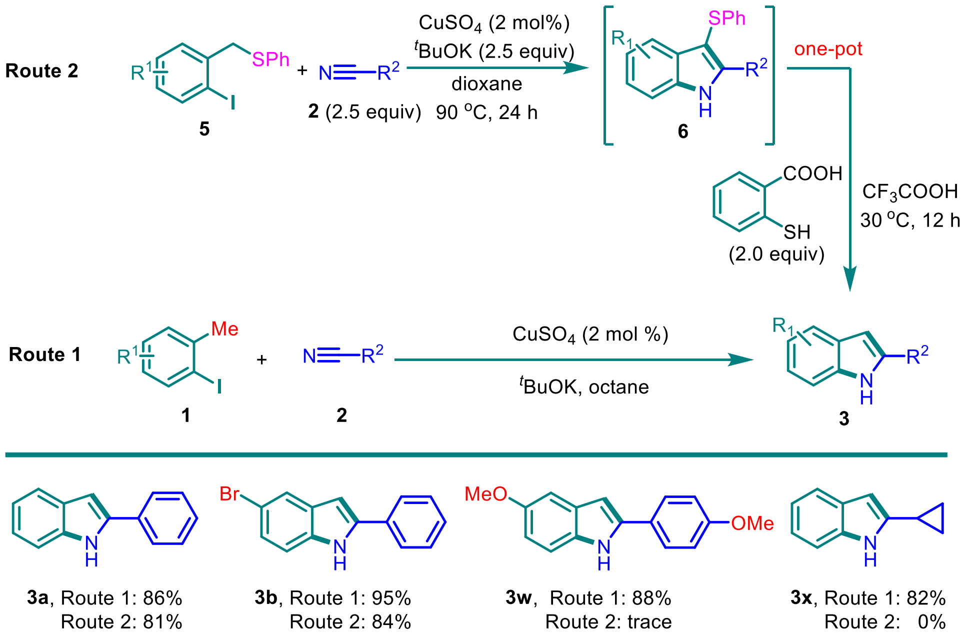

C3-phenylthio can be easily removed in the presence of 2-me
captobenzoic acid and CF_3CO_2H.[75] Thus PhS-group can be used a
a leaving group in organic synthesis. Thio-groups are better stab
lizer for adjacent carbon radicals. As a result, some unavailable i
doles from **1** and **2** can be achieved via **5**. In Fig. 7, a one-pot c
clization-deprotection synthesis of **3** either from **5** or **1** is demo
strated. Both indoles **3a** and **3b** were obtained in high yields v
both routes, whereas indoles **3w** and **3x** could only be afforded b
route 2 in high yields. Compound **3w** is a key intermediate for
potential anti-breast cancer medicine.

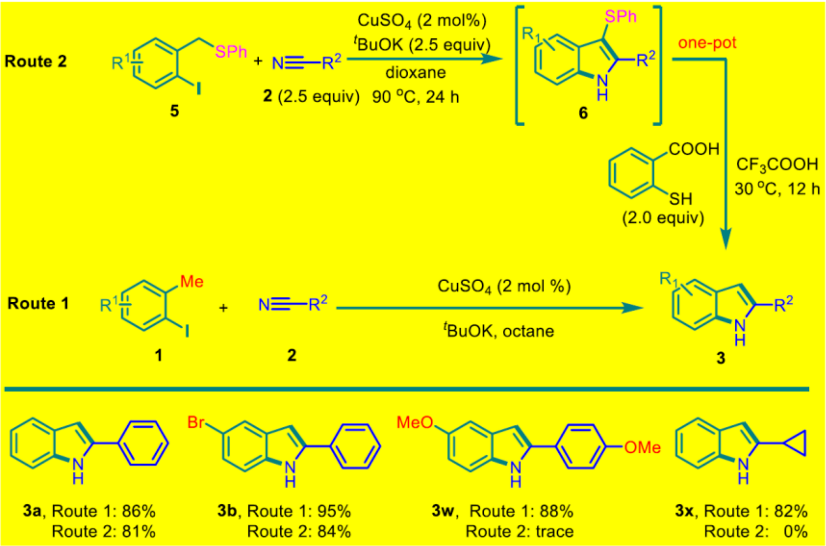

Fig. 7. Comparison the Synthesis of 3 from 1 and 5.

The reviewer considers the present reaction involves mechanistically interesting aspects such as anion-to-radical conversion and formation of iminyl radical through radical addition to nitrile. However, the reviewer is afraid that the practicality and the scope of the reaction may not strongly appeal to the audience of Nature Communications.

[Reply]: Thanks for the comment!

We believe that after revison this method has demostrared that it is an obvious ***practical*** synthetic method with ***wide scope***. From easily available starting materials and cheap naturally abundant copper salt, almost all C2-C7 substituted indoles without *N*-protection groups can be synthesized. Both C2-aryl and C2-alkyl indoles, especially C2-*n*-alkylindoles which is hard to install can be smoothly synthesized by this method. Our method can tolerate a wide spectrum of functional groups such as CO_2H, ArOH, amides, ester, halogen, etc. The large synthesis of pharmaceuticals in upto 12 grams has proven its ***practicality*** *and* ***scope*** in organic synthesis. Compared to existing indoles synthesis, this method must be a novel and practical stunning new member. In addition, the mechanistic insight has also proven its novelty in organic reactions. Anyway, any comments would be helpful for the authors to improve the manuscript. We thank the critical comments from this reviewer, which forth us to try our best to do even better.

Reviewer #2

Efficient synthesis of indole derivatives is an interesting and important research topic in synthetic organic chemistry. The authors describe radical cyclization reactions of ortho-halotoluenes and nitriles in the presence of catalytic amounts of a Cu(II) salt and 1 eq. of KOtBu. The reactions proceeded to afford the desired indole derivatives in moderate to high yields. The authors insist that this is the first example of a catalytic anion-radical redox relay without any oxidant. The authors also show good substrate scope. I think that this reaction is interesting and valuable for the synthesis of indole derivatives. However, while the authors investigate utility of this reaction, mechanistic consideration about the catalysis seems to be poor. For example, information of the active copper species is not provided in this manuscript. In Table 1, some Cu(I) species also promoted the reaction. I wonder what is the real active Cu species. In the reaction system, CuSO4 can react with KOtBu to form Cu(OtBu)2, and electron rich Cu(II) species is sometimes not so stable. The authors should provide information about the active Cu species. In the substrate scope, availability of other ortho-alkyl-halobenzenes such as ortho-ethyl-iodobenzene and ortho-alkylphenols protected and activated by Tf group is interesting. The authors should show possibility of those substrates.

After addressing the above issues, this article may be accepted for publication in Nat. Commun.

[Reply]: Thanks for the comment!

To dig out the experimental evidence for the catalytical active species of copper catalyst in this reaction, we first compared the reactivety of Cu(II) and Cu(I) in this reaction. An identical kinetic has been established (see Fig. R1). Either Cu(II) or Cu(I) does catalyze the reaction because the reaction without copper catalyst is much slower. To confirm the active Cu-species, the reaction mixture was directly dired for XPS test. Reactions with either Cu(II) or Cu(I) demonstrates same XPS spectrum. In each XPS experiment, Cu(I) and Cu(0) are determined. Plus the kinetic study, Cu(I) must be involved in the reaction. Just as this reviewer pointed out, if $Cu(II)(OtBu)_2$ formed, it must be unstable. Actually, the direct detection or preparation of $Cu(II)(OtBu)_2$ was unsuccesful. In the reaction model in Fig. 2A in the manuscript, a Cu-catalyzed reaction cycle has been presented. However, an Ullmann-type tandem cyclization bas been ruled out on the basis of control reaction in Fig. 2D. Thus what could be confirmed is that this reaction should definitely involve Cu(I), which might be generated via the reduction of Cu(II) during the catalysis. Cu(0) is generated from the further reduction of Cu(I). This issue has been mentioned in the manuscript and marked in yellow (see the copy bellow).

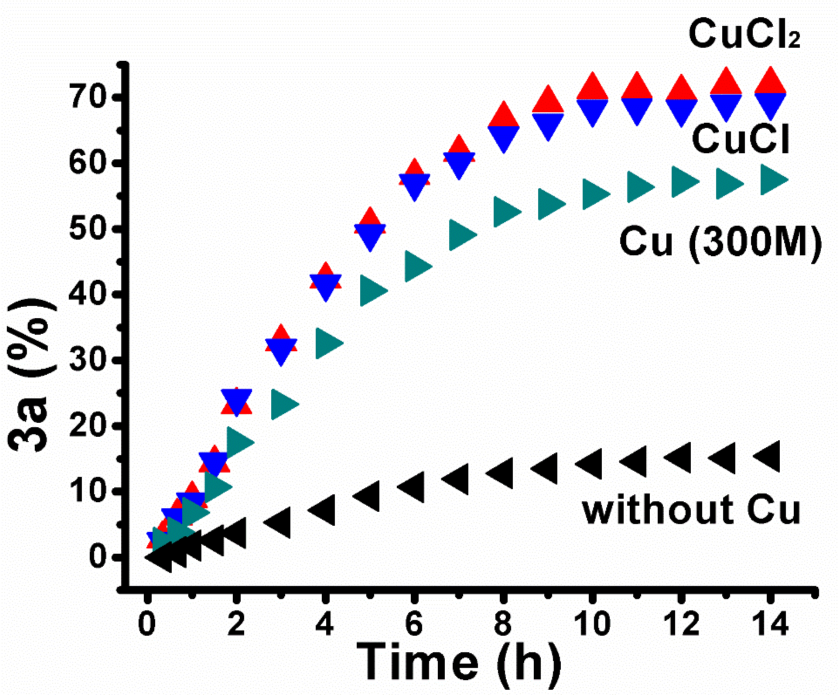

Fig. R1. Kinetic study for the reaction with $CuCl_2$, CuCl and wothout copper catalyst.

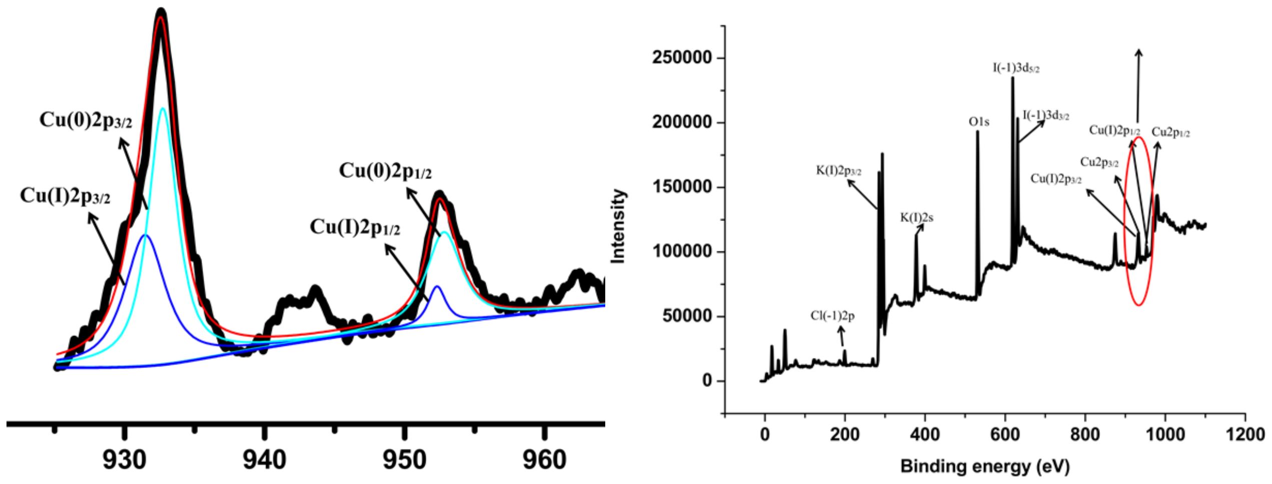

Fig. R2. XPS experiment for the reaction with $CuCl_2$

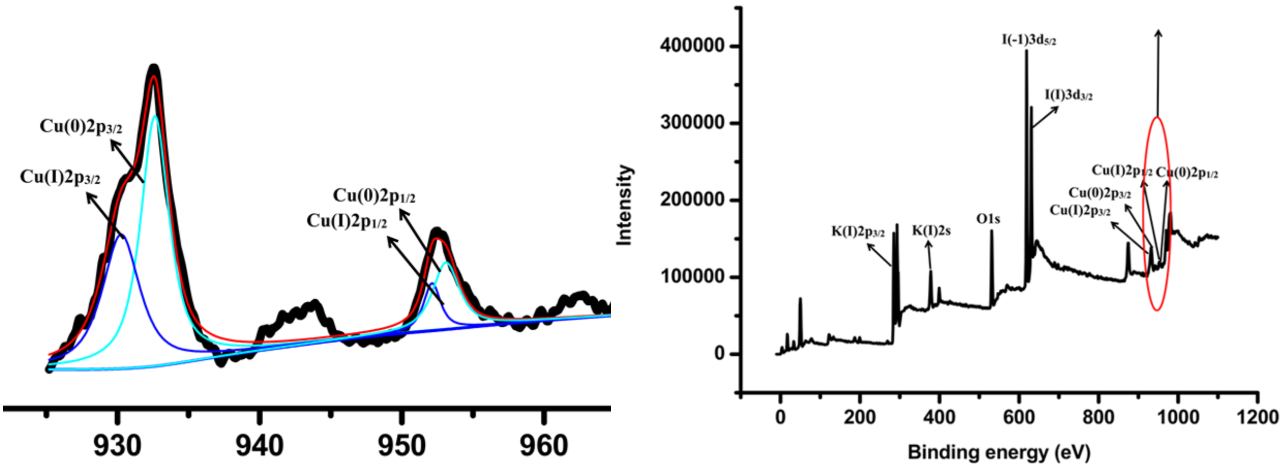

Fig. R3. XPS experiment for the reaction with CuCl

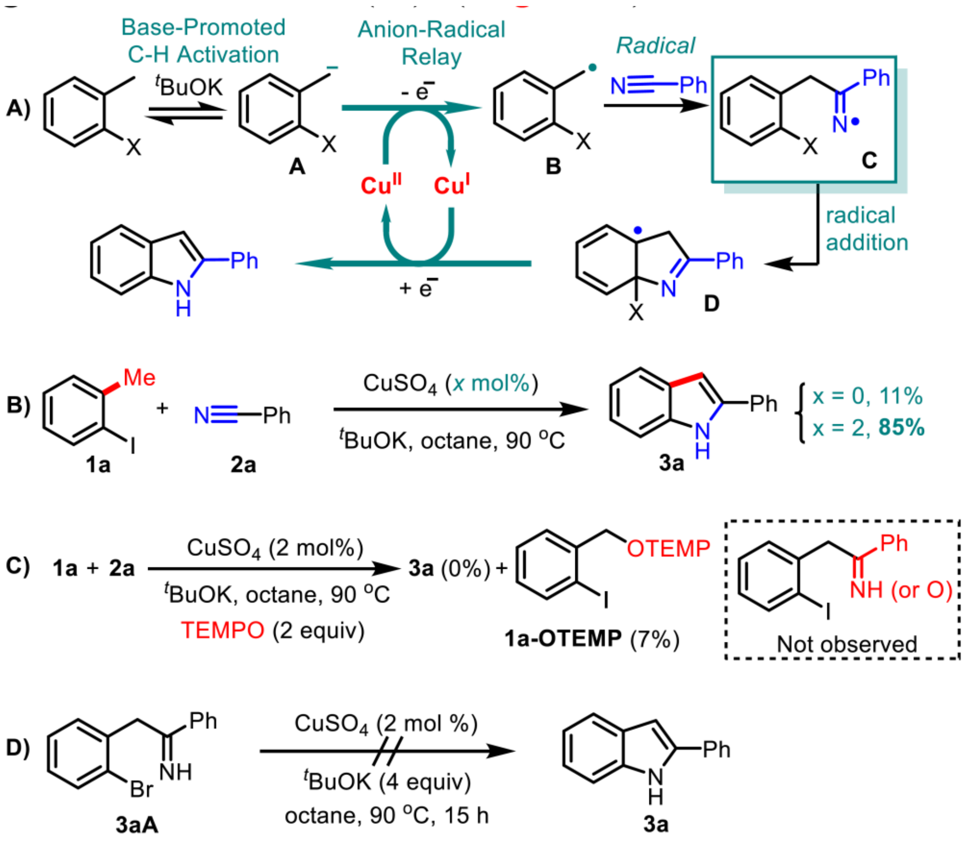

Fig. 2. Iminyl radical from anion-radical redox relay.

The reaction mechanism has been investigated with experimental evidence. In the reaction model in Fig. 2A, a Cu-catalyzed reaction cycle has been presented. In the kinetic study, $CuCl_2$ and CuCl give the identical results with same induction phases and Cu(0) gives lower conversion in a slower reaction rate but much higher than that in the absence of copper (Fig. 9A), indicating Cu(II) and Cu(I) should directly involve the reaction whereas Cu(0) might be slowly converted to higher oxidation states to directly participate the catalytic cycle. The XPS experiments show that Cu(II) has been reduced to Cu(I) and Cu(0) therefore Cu-salts have been involved in the reaction. However, an Ullmann-type tandem cyclization bas been ruled out on the basis of control reaction in Fig. 2D. Therefore the reaction could possibly pass through Fig. 1A via an iminyl radical addition to aryl rings.

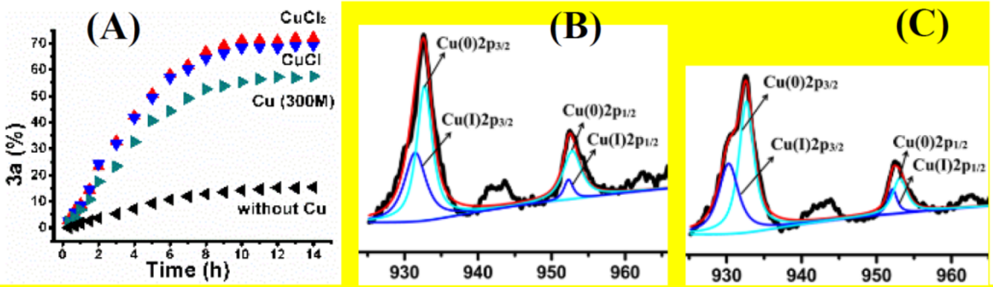

Fig. 9. Kinetic study and XPS experiments. (A) Kinetic study of reaction from **1a** and **2a** to **3a** with $CuCl_2$ and CuCl. XPS experiments of the reactions with $CuCl_2$ (B) and CuCl (C).

Other 2-iodo alkyl benzene such as 2-iodo ethyl benzene can slso transform to indole product in 55% yield. And most unreacted starting marterial was recovered.

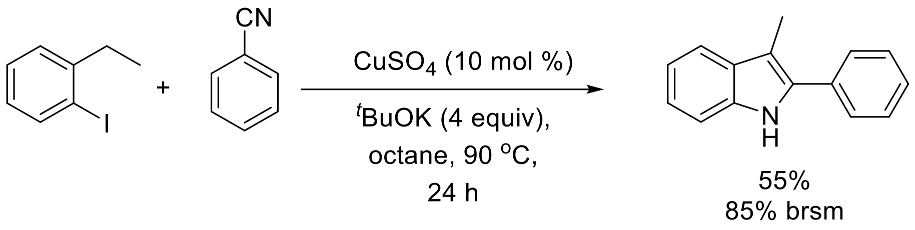

With respect to the Tf-protected substrates, Tf was removed in all reactions. Therefore Tf is not a suitable protection group and OTf is not a reactive leaving group in this reaction. For other tolerable protecting groups such as halogen, OH, COOH, amide, ester, silyl protecting group, MOM, CF_3, nitrile, pyridine, etc, the results are listed below:

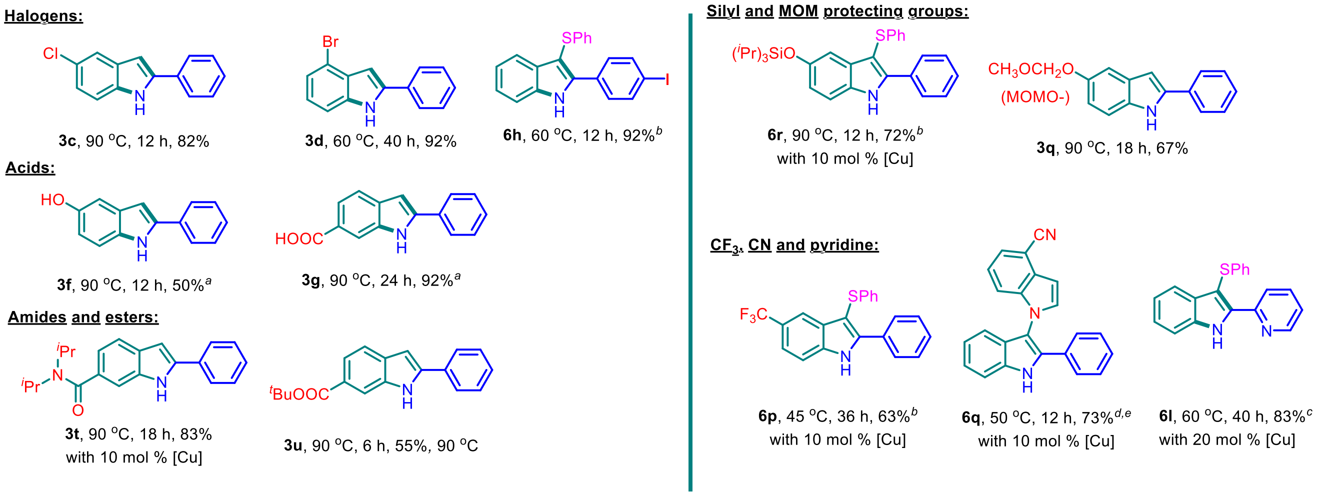

Reviewer #3

The manuscript by Kang and coworkers reports on a copper-catalysed indole formation from toluenes and nitriles comprising a sequential C-C and C-N bond instalment. The authors propose a radical relay mechanism, which based on their control experiments from Figures 2B-D appears reasonable. In principle, one would anticipate that the radical relay occurs at the stage of the iminyl radical. Its reduction by cuprous salt would generate an iminyl anion, which would undergo copper-promoted C-N coupling following extensive work, as for example by Buchwald. The authors exclude such a possibility pointing to the outcome of their control experiment from Figure 2D. I agree with this conclusion, but maybe it would provide a better understanding if they discussed this important mechanistic context in more detail. This negative outcome on Ullmann coupling is surprising, but may be owed to the absence of any diamine ligands in the catalyst.

The major problem with this work is the clear limitation it presents due to the required strong basic conditions (usually, 4 equivalents of KOtBu). This limits the toluene component to the category of non-base-labile substituents and the nitrile component to benzonitriles. Such a limitation is severe from the synthetic standpoint. Nevertheless, the authors have provided a long list of successful examples in Figures 3, 5 and 6. Some preliminary application (Figures 4 and 7) is also provided. With respect to the latter Figure 7, it appears surprising that this synthesis is carried out with a sulfide placeholder at the benzylic position. Based on the successful example of 3m, why is it not possible to use the corresponding 3-methyl, 4-iodoanisole instead? This would reduce the synthesis of 8 to two steps.

In summary, this manuscript reports a potentially useful synthetic procedure for indole synthesis. However, it requires major revision as the authors need to explore and address the issue of base labile functional groups prior to publication.

[Reply]: Thanks for the comment!

The excess nitrile starting materials can be recovered quantitatively. For example, in the reaction below, 4.1 out of 5 equiv of nitrile has been recovered when 85% yield of product was obtained. That is to say, almost quantitative unreacted nitrile could be recovered. Morever, the amount of nitrile can be further lowered down to 1.5 equiv for the reaction of **5** and **3**. As shown below, the reaction with 1.5 equiv of nitrile affords corresponding indole **6d** in 85%. We finally choose 2.5 equiv of base and nitrile for the optimized conditions.

This issue has been mentioned in the manuscript inTables 1-2 and marked in yellow.

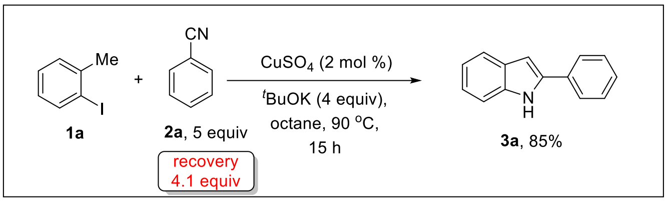

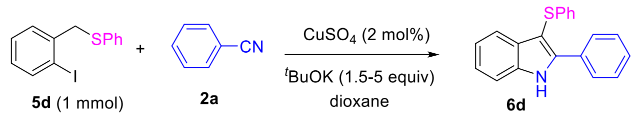

PhCN (equiv)	^{t}BuOK (equiv)	t (h)	temp.(°C)	6d (%)
5	4	11	60	93
2.5	2.5	24	90	92
2	2	36	90	75
2	3	24	90	91
1.5	3	24	90	85

C3-substituted indoles can also be synthesized by this method. Reaction conditions were further optimized on the basis of Table 1 (Table 2). Despite of the fact that even 1.5 equiv of nitrile affords 85% of 6d (entry 5), the conditions listed in entry 2 were chosen for the balance of yields and amounts of base and nitriles. Indoles

Table 2. Reaction conditions for the synthesis of 6.

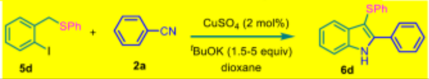

Entry[a]	PhCN (equiv)	^{t}BuOK (equiv)	t (h)	6d (%)
1[b]	5	4	11	93
2	2.5	2.5	24	92
3	2	2	36	75
4	2	3	24	91
5	1.5	3	24	85

[a] Conditions: **5d** (1 mmol), **2a** (1.5-5 mmol), $CuSO_4$ (2 mol %), ^{t}BuOK (2-5 mmol), dioxane (0.75 mL), argon. Yields were determined by ^{1}H NMR. [b] At 60 °C.

Other tolerable protecting groups such as halogen, OH, COOH, amide, ester, silyl protecting group, MOM, CF_3, nitrile, pyridine, etc, the results are listed below:

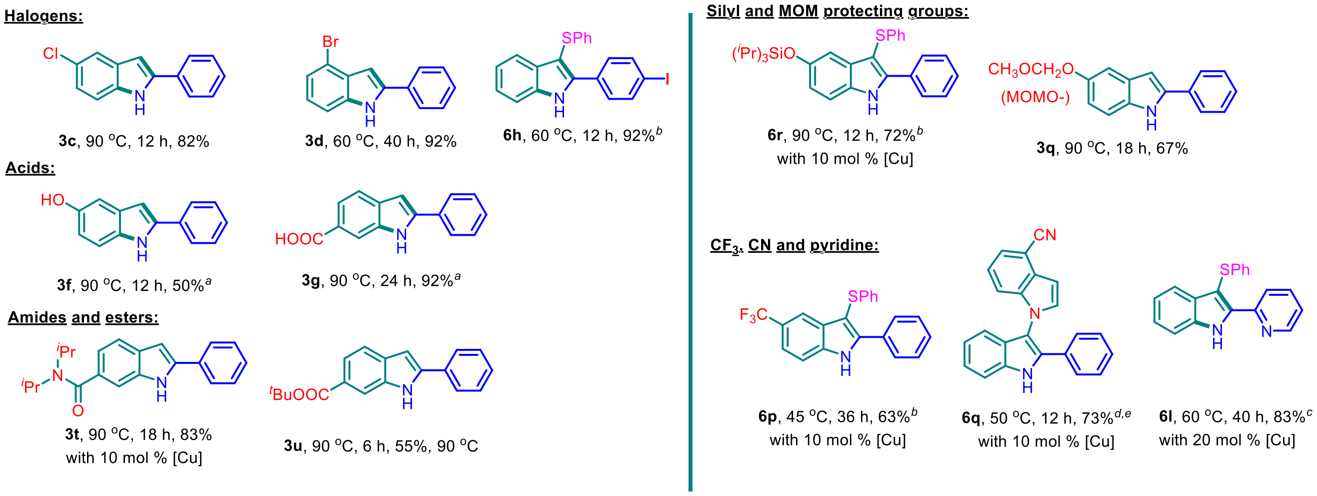

In the original manuscript, we have demostrated that the direct synthesis of key intermediate indole **3w** (originally **3r**) from 2-iodo-toluene needs two steps.

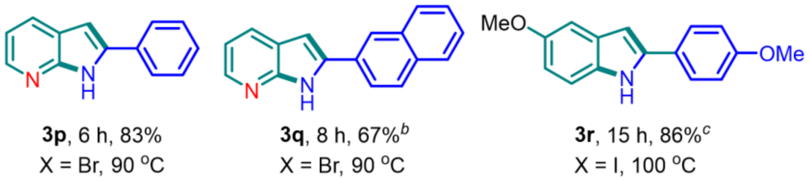

Fig. 3. Scope of methyl (het)arenes and nitriles. Reaction conditions: Conditions: **1** (1 mmol), **2** (5 mmol) $CuSO_4$ (2 mol %), ^{t}BuOK (4 mmol), octane (0.75 mL). Extra 1 mmol ^{t}BuOK was used for **3f** and **3g**. [a] 2 (1.5 mL) was used as solvent. [b] Octane (3 mL). [c] **3r** was obtained from **6f** and PhS was removed by treating with 2-mercaptobenzoic acid and CF_3CO_2H at RT for 12 h.

To make this question more clear, we have performed the comparison experiments as shown below, indoles **3a** and **3b** can both be obtained from **5** or **1**, whereas **3w** and **3x** could not. This is why route 2

was applied in the synthesis of **8**. This issue has been clarified by the insertion of Fig. 7 (see the copy below).

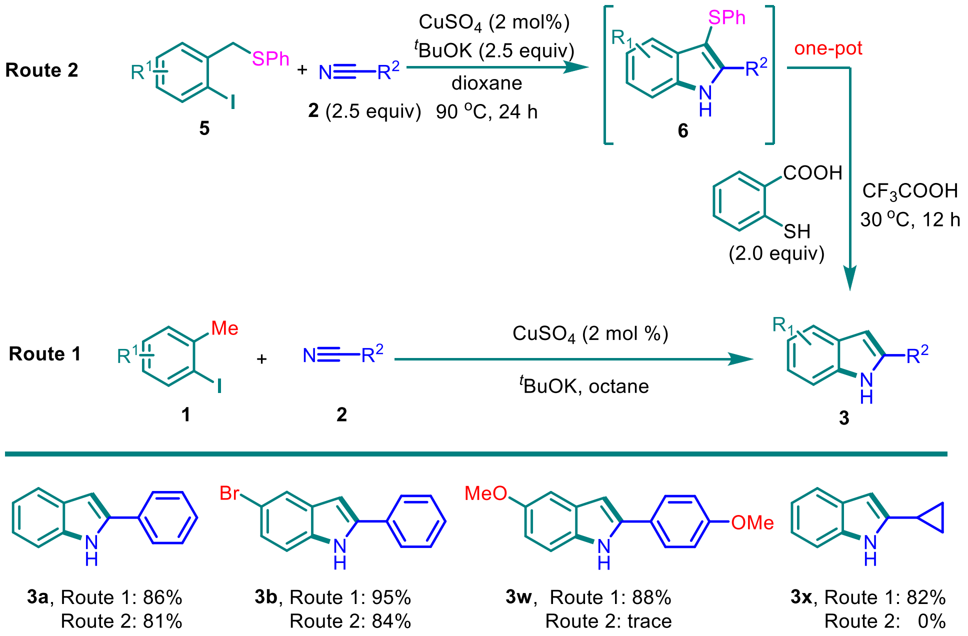

C3-phenylthio can be easily removed in the presence of 2-me captobenzoic acid and CF_3CO_2H.[75] Thus PhS-group can be used a leaving group in organic synthesis. Thio-groups are better stab lizer for adjacent carbon radicals. As a result, some unavailable i doles from **1** and **2** can be achieved via **5**. In Fig. 7, a one-pot c clization-deprotection synthesis of **3** either from **5** or **1** is demo strated. Both indoles **3a** and **3b** were obtained in high yields v both routes, whereas indoles **3w** and **3x** could only be afforded b route 2 in high yields. Compound **3w** is a key intermediate for potential anti-breast cancer medicine.

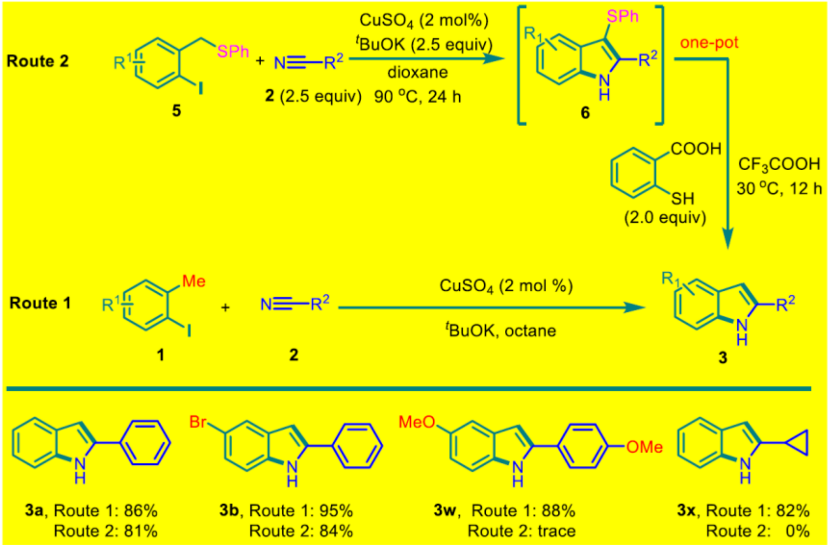

Fig. 7. Comparison the Synthesis of 3 from 1 and 5.

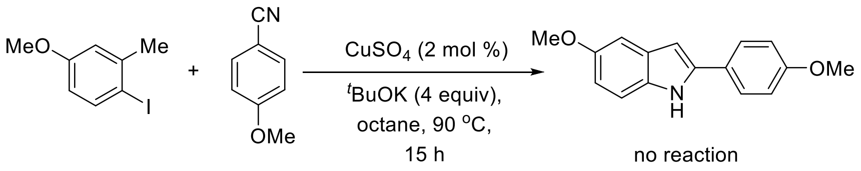

The title should be shortened by removal of the last three words.

[Reply]: Thanks for suggesting. The title has been changed as requested.

As to another problem, the manuscript contains a large number of typos and grammar mistakes that need to be corrected.

Some examples on page 3 include:

…uneasy to be achieved… should be: …difficult to generate…

The yields seems… should be: The yields seem…

Figure 6: Reaction conditions (not Reaction Condition)

…reagent 8 is synthesized though 3 steps… should be: …reagent 8 is synthesized through 3 steps…

..and as alow as… should be: … and as slow as…

…imynyl radials… shoud be: ….iminyl radicals…

[Reply]: Thanks for remiding. All the typos and other related errors have been checked and corrected as careful as we can.

The authors appreceate all reviewers for their helpful comments and suggestions. Without your comments we could hard do better job.

Reviewers' comments:

Reviewer #1 (Remarks to the Author):

The authors have made substantial effort to address comments and requests from myself and other reviewers, and solved some of the critical issues. They have demonstrated that the reaction can be performed using a lower amount of nitrile substrate and tBuOK, and also showed additional examples of chloro- and bromotoluene substrates. While my impression that the syntheses in Fig 4 and 8 are routine has not changed, the doubt about Fig 8 has been resolved. The authors have also performed additional experiments to gain insight into the mechanism and the catalytically active species involved. Overall, the quality of the manuscript has been improved, and its publication in Nature Communication may be acceptable.

1. Regarding Fig 7, the reviewer wonders why direct synthesis of 3w and 3x has failed. The authors should discuss the possible reason for the failure of each case to make the limitation of the present reaction clearer.

2. The XPS spectra in Fig 9B and 9C should be more carefully presented and discussed in the manuscript. The spectra lack the labels on x-axis and y-axis, and it is not clear under which conditions these spectra were measured (e.g., after how many hours). Also, what about the spectral behavior of the optimized reaction system using CuSO4 rather than CuCl and CuCl2?

3. The reaction using 2-iodoethylbenzene should be included in Fig 5.

4. There remain quite a few typos and grammatical errors. Shown below are errors found in the last page.
• Cu(II) and Cu(I) should directly involve the reaction
• as slow as only 2 mol%
• Upon completion of astarting materials
• directly prified

Reviewer #2 (Remarks to the Author):

The authors reported radical cyclization reactions of ortho-halotoluenes and nitriles in the presence of a catalytic amount of Cu(II) salt and KOtBu. Based on the reviewer’s comments, they revised their manuscript in several points. They conducted some experiments about Cu species and confirmed that Cu(I) and Cu(0) existed in the real reaction system, while they could not confirm Cu(II). From those results, I wonder that really the Cu(II)-C(I) redox cycle works in the current system. I think that it is difficult to rule out other possibilities, ex. Cu(I)-Cu(0) cycle, that Cu is just an initiator, etc. The authors should discuss and conduct further experiments if needed whether their mechanistic hypothesis is correct or not in the real system. The authors insist that this redox cycle is the first example of a catalytic anion-radical redox relay without any oxidant. Therefore, this discussion and further experiments, regeneration of Cu(II), is very important as a concept. I think that this article cannot be accepted without addressing this point.

Reviewer #3 (Remarks to the Author):

The manuscript by Kang and co-workers is a revised version of an earlier submission. The work has greatly benefited from the revision and all of my earlier concerns were fully addressed. Especially, my earlier problem with the synthesis strategy of 3w has been clarified. Overall, this work is a very useful

addition to indole synthesis, particularly taking into account the large-scale reactions. I therefore support publication of this work in its present form.

Reviewer #1

The authors have made substantial effort to address comments and requests from myself and other reviewers, and solved some of the critical issues. They have demonstrated that the reaction can be performed using a lower amount of nitrile substrate and tBuOK, and also showed additional examples of chloro- and bromotoluene substrates. While my impression that the syntheses in Fig 4 and 8 are routine has not changed, the doubt about Fig 8 has been resolved. The authors have also performed additional experiments to gain insight into the mechanism and the catalytically active species involved. Overall, the quality of the manuscript has been improved, and its publication in Nature Communication may be acceptable.

1. Regarding Fig 7, the reviewer wonders why direct synthesis of **3w** and **3x** has failed. The authors should discuss the possible reason for the failure of each case to make the limitation of the present reaction clearer.

[Reply]: Thanks for suggesting. The radical addition to nitrile is a nucleophilic process, thus the electron rich nitriles are normally less reactive. If the stability of radical intermediates are better, it gives more chance. Thio-groups are better stabilizer for adjacent carbon radicals. As a result, thio-substrate **5** is much better than **1** and some unavailable indoles from **1** and **2** can be achieved via **5**. This issue has been mentioned in the text and a copy is shown below.

C3-phenylthio can be easily removed in the presence of 2-mercaptobenzoic acid and CF_3CO_2H.[75] Thus PhS-group can be used as a leaving group in organic synthesis. In Fig. 7, a one-pot cyclization-deprotection synthesis of **3** either from **5** or **1** is demonstrated. Both indoles **3a** and **3b** were obtained in high yields via both routes, whereas indoles **3v** and **3w** could only be afforded by route **1** in high yields. The radical addition to nitrile is a nucleophilic process, thus the electron rich nitriles are normally less reactive. If the stability of radical intermediates are better, it gives more chance. Thio-groups are better stabilizer for adjacent carbon radicals. As a result, thio-substrate **5** is much better than **1** and some unavailable indoles from **1** and **2** can be achieved via **5**. Compound **3v** is a key intermediate for a potential anti-breast cancer medicine.

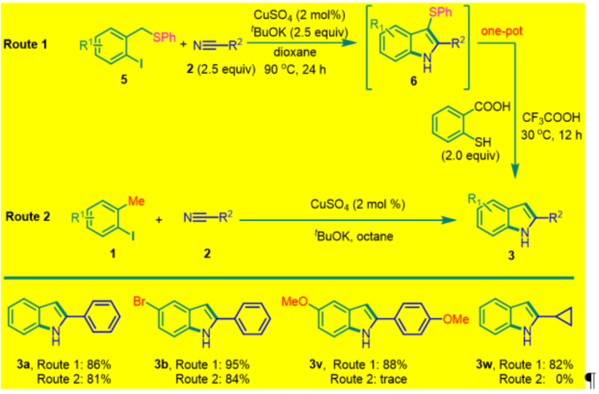

Fig. 7. Comparison the Synthesis of 3 from 1 and 5.

2. The XPS spectra in Fig 9B and 9C should be more carefully presented and discussed in the manuscript. The spectra lack the labels on x-axis and y-axis, and it is not clear under which conditions these spectra were measured (e.g., after how many hours). Also, what about the spectral behavior of the optimized reaction system using CuSO4 rather than CuCl and CuCl2?

[Reply]: Thanks for reminding. The issue mentioned above have been solved in the revised manuscript. The labels on x-axis and y-axis have been added. The measurement was done after 8 h reaction. The detailed conditions have been added as reference 77. In previous version of manuscript, CuCl and $CuCl_2$ were used instead of $CuSO_4$ because we wanted to compare the behavior of Cu(II) and Cu(I) as the catalysts whereas Cu(I)-sulfate is not available. In this version of manuscript, according to the suggestion from this Reviewer, we

performed the XPS measurement using $CuSO_4$ as catalyst. To our pleasure, in the reaction catalysed by $CuSO_4$, only Cu(II) and Cu(I) were observed whereas Cu(0) was not. Later we realized that Cu(0) was generated in the working up process in the previous experiments with CuCl or $CuCl_2$. Thus current evidence of XPS experiments for copper oxidation states proves the Cu(II)/Cu(I)-cycle is sufficient. The procedure for XPS experiments: A Schlenk tube charged with ^{t}BuOK (4 mmol, 449 mg) and $CuSO_4$ (0.1 mmol, 16 mg) was dried under high vacuum for 15 min. Octane (0.75 mL), **1** (1 mmol), and **2a** (5 mmol) were added under argon and stirred at 90 °C for 8 h. The mixture was concentrated under vacuum and the solid was measured by XPS tests. XPS Instrument type: Thermo ESCALAB 250Xi; X-ray excitation source: monochromatic Al Ka (hv =1486.6 eV), power 150W, X-ray beam 500µm; Energy analyzer fixed transmission energy: 30 eV.

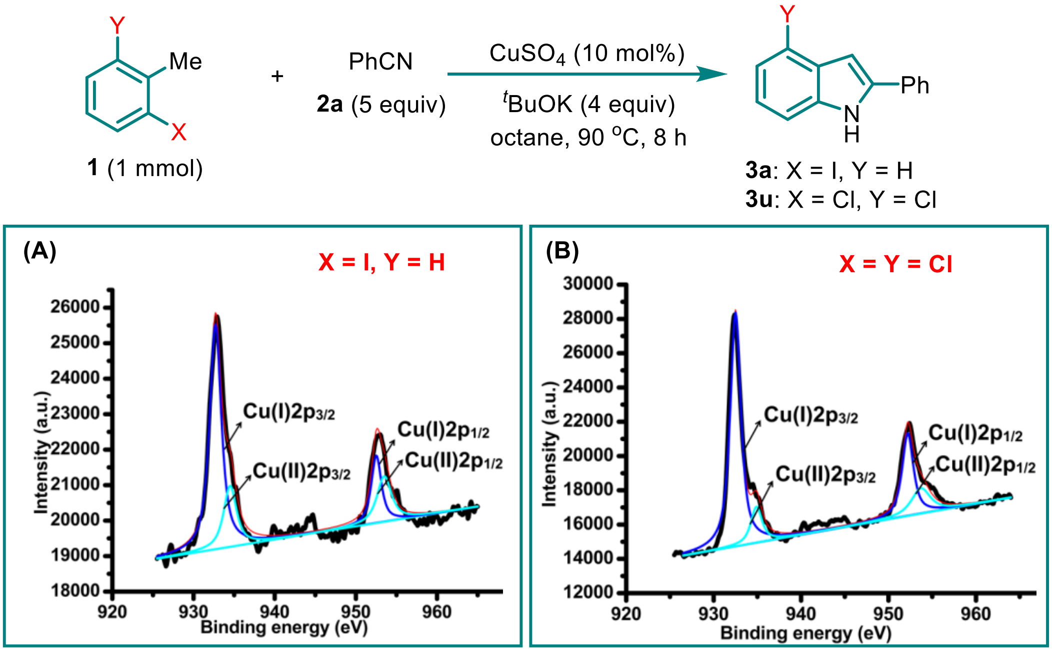

To further confirm the amount of Cu(II), the iodometric titration experiment was done for the reaction after 8 hours. By above titration, 65% Cu(II) was determined in the reaction of **1u** to **3u**. The experiment was perform in following steps: The iodometric determination of Cu(II) in the reaction mixture: A Schlenk tube charged with ^{t}BuOK (4 mmol, 449 mg) and $CuSO_4$ (0.1 mmol, 16 mg) was dried under high vacuum for 15 min. Octane (0.75 mL), **1u** (1 mmol) and **2a** (5 mmol) were added under argon and stirred at 90 °C. The reaction was stirred for 8 h and quenched by CH_2Cl_2 and extracted with H_2O. The aqueous phase were combined. When the pH was adjusted to 7-8, KI (8 mmol) and 5 ml of 0.5 wt% starch solution were sequentailly added. Sodium thiosulfate standard titration solution [c($Na_2S_2O_3$) = 0.05 mol/L] was used to titrate until the solution blue disappeared. Cu(II) was determined as 0.065 mmol (65% based on 10 mol% $CuSO_4$).

This issue has been addressed in the manuscript and the copy is shown below. The procedure for the measurements were described in ref. 77-78.

The reaction mechanism has been investigated with experimental evidence. In the reaction model in Fig. 2A, a Cu-catalyzed reaction cycle has been presented. The XPS experiments for the reaction using $CuSO_4$ as catalyst demonstrated both Cu(II) and Cu(I),[77] indicating that Cu-salts have been involved in the reaction. The iodometric determination of Cu(II) in the reaction mixture was performed using a chloro-substrate to avoid the interruption of KI generated from the reaction. By above titration, 65% Cu(II) was determined.[78] Therefore the reaction could possibly pass through Fig. 1A via an iminyl radical addition to aryl rings.

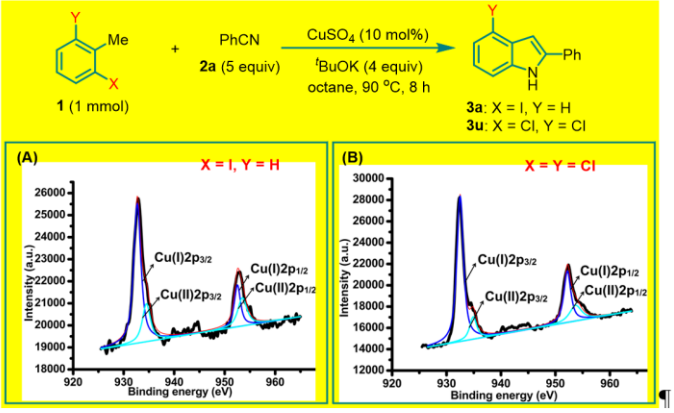

Fig. 9. Detection of Cu(II) and Cu(I) by XPS experiments with $CuSO_4$. (A) To **3a** when X = I and (B) to **3v** when X = Cl. For detailed conditions, see ref. 77. XPS Instrument type: Thermo ESCALAB 250Xi; X-ray excitation source: monochromatic Al Ka (hv =1486.6 eV), power 150W, X-ray beam 500µm; Energy analyzer fixed transmission energy: 30 eV.

Ref. 77-78:

77. The procedure for XPS experiments: A Schlenk tube charged with ^{t}BuOK (4 mmol, 449 mg) and $CuSO_4$ (0.1 mmol, 16 mg) was dried under high vacuum for 15 min. Octane (0.75 mL), **1** (1 mmol), and **2a** (5 mmol) were added under argon and stirred at 90 °C for 8 h. The mixture was concentrated under vacuum and the solid was measured by XPS tests.

78. The iodometric determination of Cu(II) in the reaction mixture: A Schlenk tube charged with ^{t}BuOK (4 mmol, 449 mg) and $CuSO_4$ (0.1 mmol, 16 mg) was dried under high vacuum for 15 min. Octane (0.75 mL), **1u** (1 mmol) and **2a** (5 mmol) were added under argon and stirred at 90 °C. The reaction was stirred for 8 h and quenched by CH_2Cl_2 and extracted with H_2O. The aqueous phase were combined. When the pH was adjusted to 7-8, KI (8 mmol) and 5 ml of 0.5 wt% starch solution were sequentailly added. Sodium thiosulfate standard titration solution [c($Na_2S_2O_3$) = 0.05 mol/L] was used to titrate until the solution blue disappeared. Cu(II) was determined as 0.065 mmol (65% based on 10 mol% $CuSO_4$).

3. The reaction using 2-iodoethylbenzene should be included in Fig 5.

[Reply]: Thanks for reminding. This example has been moved from Fig. 3 to Fig. 5 as **6r**.

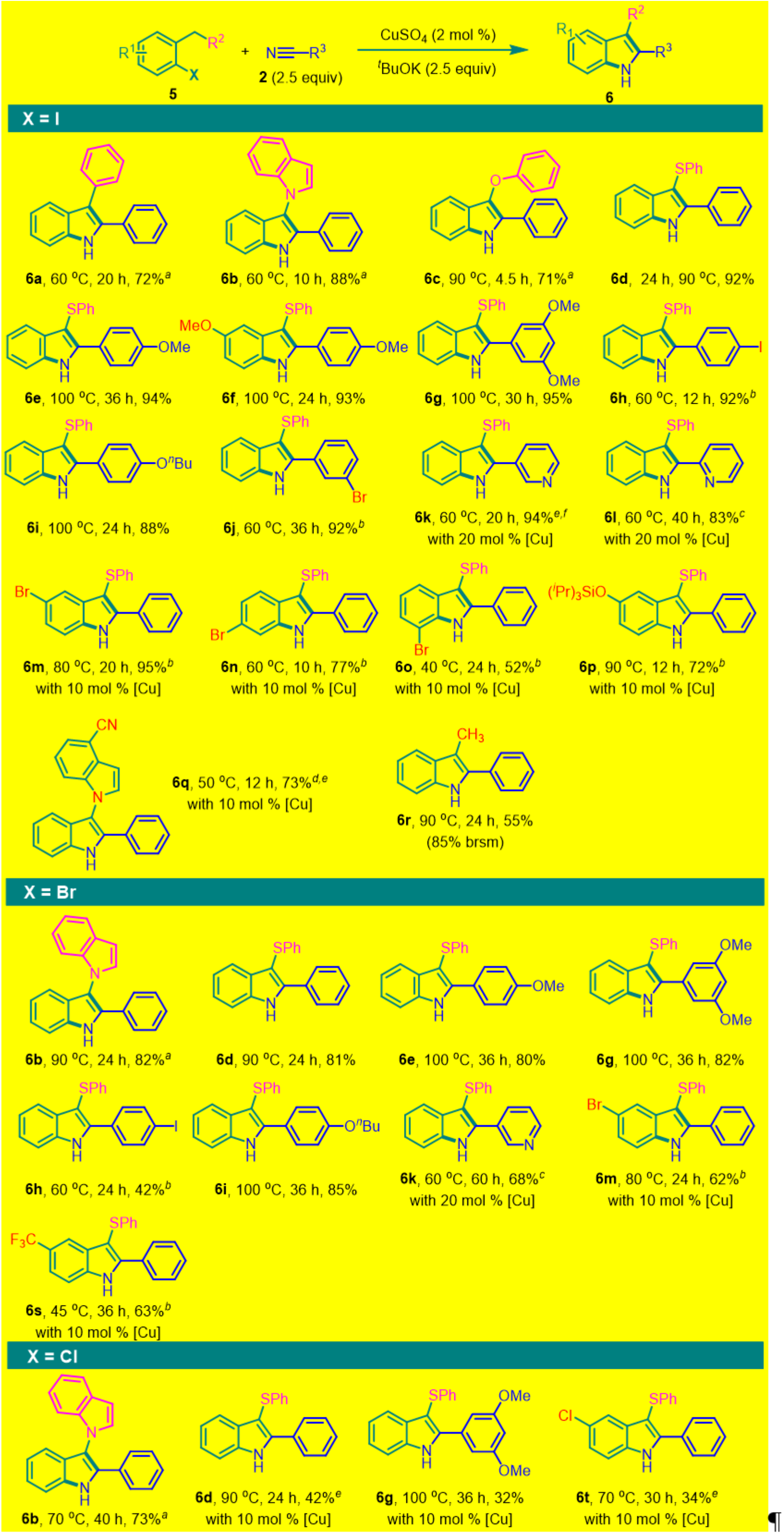

Fig. 5. Scope of C3-functionalized indoles. Reaction conditions: **5** (1 mmol), **2** (2.5 mmol), $CuSO_4$ (2 mol %), ^{t}BuOK (2.5 mmol), dioxane (0.75 mL). [a] **2** (5 mmol), ^{t}BuOK (4 mmol), octane (0.75 mL) instead of dioxane. [b] Dioxane (1.5 mL). [c] Dioxane (20 mL). [d] ^{t}BuOK (4 mmol). [e] **2** (1.5 mL) was used as solvent. [f] **2** (15 mL) was used as solvent.

4. There remain quite a few typos and grammatical errors. Shown below are errors found in the last page.

• Cu(II) and Cu(I) should directly involve the reaction

• as slow as only 2 mol%

• Upon completion of astarting materials

• directly prified

[Reply]: Thanks for reminding. All the typos have been corrected.

Reviewer #2 (Remarks to the Author):

The authors reported radical cyclization reactions of ortho-halotoluenes and nitriles in the presence of a catalytic amount of Cu(II) salt and KOtBu. Based on the reviewer's comments, they revised their manuscript in several points. They conducted some experiments about Cu species and confirmed that Cu(I) and Cu(0) existed in the real reaction system, while they could not confirm Cu(II). From those results, I wonder that really the Cu(II)-C(I) redox cycle works in the current system. I think that it is difficult to rule out other possibilities, ex. Cu(I)-Cu(0) cycle, that Cu is just an initiator, etc. The authors should discuss and conduct further experiments if needed whether their mechanistic hypothesis is correct or not in the real system. The authors insist that this redox cycle is the first example of a catalytic anion-radical redox relay without any oxidant. Therefore, this discussion and further experiments, regeneration of Cu(II), is very important as a concept. I think that this article cannot be accepted without addressing this point.

[Reply]: Thanks for the comments. The issue mentioned above have been solved in the revised manuscript. In previous version of manuscript, CuCl and $CuCl_2$ were used instead of $CuSO_4$ because we wanted to compare the behavior of Cu(II) and Cu(I) as the catalysts whereas Cu(I)-sulfate is not available. In this version of manuscript, according to the suggestion of Reviewers, we performed the XPS measurement using $CuSO_4$ as catalyst. To our pleasure, in the reaction catalysed by $CuSO_4$, only Cu(II) and Cu(I) were observed whereas Cu(0) was not. Later we realized that Cu(0) was generated in the working up process in the previous experiments with CuCl or $CuCl_2$. Thus current evidence of XPS experiments for copper oxidation states proves the Cu(II)/Cu(I)-cycle is sufficient. The procedure for XPS experiments: A Schlenk tube charged with ^{t}BuOK (4 mmol, 449 mg) and $CuSO_4$ (0.1 mmol, 16 mg) was dried under high vacuum for 15 min. Octane (0.75 mL), **1** (1 mmol), and **2a** (5 mmol) were added under argon and stirred at 90 °C for 8 h. The mixture was concentrated under vacuum and the solid was measured by XPS tests. XPS Instrument type: Thermo ESCALAB 250Xi; X-ray excitation source: monochromatic Al Ka (hv =1486.6 eV), power 150W, X-ray beam 500µm; Energy analyzer fixed transmission energy: 30 eV.

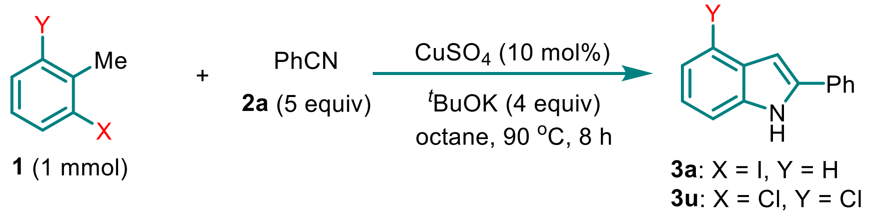

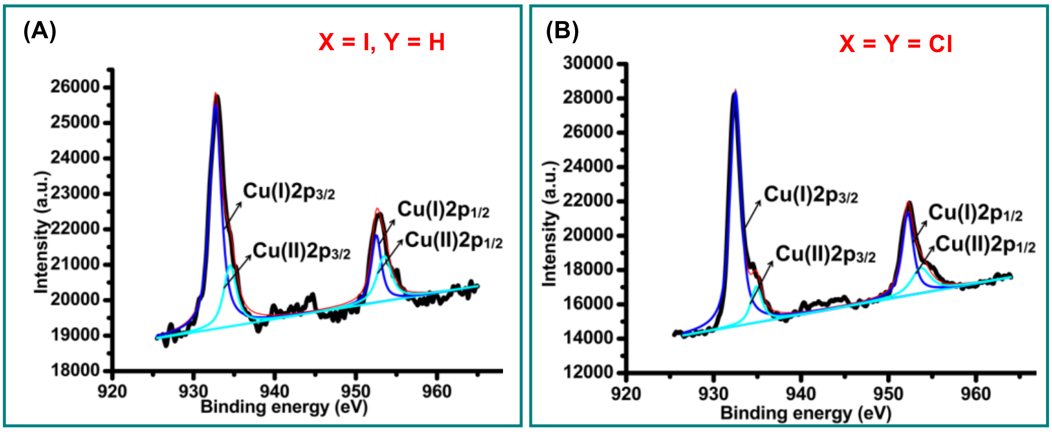

To further confirm the amount of Cu(II), the iodometric titration experiment was done for the reaction after 8 hours. By above titration, 65% Cu(II) was determined in the reaction of **1u** to **3u**. The experiment was perform in following steps: The iodometric determination of Cu(II) in the reaction mixture: A Schlenk tube charged with ^{t}BuOK (4 mmol, 449 mg) and $CuSO_4$ (0.1 mmol, 16 mg) was dried under high vacuum for 15 min. Octane (0.75 mL), **1u** (1 mmol) and **2a** (5 mmol) were added under argon and stirred at 90 °C. The reaction was stirred for 8 h and quenched by CH_2Cl_2 and extracted with H_2O. The aqueous phase were combined. When the pH was adjusted to 7-8, KI (8 mmol) and 5 ml of 0.5 wt% starch solution were sequentailly added. Sodium thiosulfate standard titration solution [c($Na_2S_2O_3$) = 0.05 mol/L] was used to titrate until the solution blue disappeared. Cu(II) was determined as 0.065 mmol (65% based on 10 mol% $CuSO_4$).

This issue has been addressed in the manuscript and the copy is shown below. The procedure for the measurements were described in ref. 77-78.

The reaction mechanism has been investigated with experimental evidence. In the reaction model in Fig. 2A, a Cu-catalyzed reaction cycle has been presented. The XPS experiments for the reaction using $CuSO_4$ as catalyst demonstrated both Cu(II) and Cu(I),[77] indicating that Cu-salts have been involved in the reaction. The iodometric determination of Cu(II) in the reaction mixture was performed using a chloro-substrate to avoid the interruption of KI generated from the reaction. By above titration, 65% Cu(II) was determined.[78] Therefore the reaction could possibly pass through Fig. 1A via an iminyl radical addition to aryl rings.

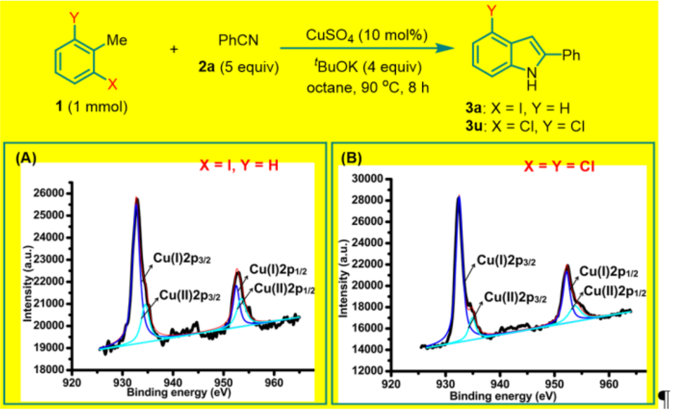

Fig. 9. Detection of Cu(II) and Cu(I) by XPS experiments with $CuSO_4$. (A) To **3a** when X = I and (B) to **3v** when X = Cl. For detailed conditions, see ref. 77. XPS Instrument type: Thermo ESCALAB 250Xi; X-ray excitation source: monochromatic Al Ka (hv =1486.6 eV), power 150W, X-ray beam 500μm; Energy analyzer fixed transmission energy: 30 eV.

Ref. 77-78:

77. The procedure for XPS experiments: A Schlenk tube charged with ^{t}BuOK (4 mmol, 449 mg) and $CuSO_4$ (0.1 mmol, 16 mg) was dried under high vacuum for 15 min. Octane (0.75 mL), **1** (1 mmol), and **2a** (5 mmol) were added under argon and stirred at 90 °C for 8 h. The mixture was concentrated under vacuum and the solid was measured by XPS tests.

78. The iodometric determination of Cu(II) in the reaction mixture: A Schlenk tube charged with ^{t}BuOK (4 mmol, 449 mg) and $CuSO_4$ (0.1 mmol, 16 mg) was dried under high vacuum for 15 min. Octane (0.75 mL), **1u** (1 mmol) and **2a** (5 mmol) were added under argon and stirred at 90 °C. The reaction was stirred for 8 h and quenched by CH_2Cl_2 and extracted with H_2O. The aqueous phase were combined. When the pH was adjusted to 7-8, KI (8 mmol) and 5 ml of 0.5 wt% starch solution were sequentailly added. Sodium thiosulfate standard titration solution [c($Na_2S_2O_3$) = 0.05 mol/L] was used to titrate until the solution blue disappeared. Cu(II) was determined as 0.065 mmol (65% based on 10 mol% $CuSO_4$).

Reviewer #3 (Remarks to the Author):

The manuscript by Kang and co-workers is a revised version of an earlier submission. The work has greatly benefited from the revision and all of my earlier concerns were fully addressed. Especially, my earlier problem with the synthesis strategy of **3w** has been clarified. Overall, this work is a very useful addition to indole synthesis, particularly taking into account the large-scale reactions. I therefore support publication of this work in its present form.

[Reply]: Thanks for the comments. The authors appreciate this reviewer again for the helpful comments and suggestions. Without your comments we could hardly improve the manuscript.

The authors appreciate all reviewers again for their helpful comments and suggestions. Without your comments we could hardly do better job.

REVIEWERS' COMMENTS:

Reviewer #1 (Remarks to the Author):

The authors have addressed the points raised for the previous version. The manuscript appears suitable for publication in Nature Communications.

Reviewer #2 (Remarks to the Author):

The authors reported radical cyclization reactions of ortho-halotoluenes and nitriles in the presence of catalytic amounts of Cu(II) salt and KOtBu. They investigated the points of my concern and confirmed Cu(II) in the current reaction system. This observation might support existence of the Cu(II)-Cu(I) catalytic cycle. I think this article can be accepted on Nat. Commun.
In page 4, "Discussion" should be "Conclusion."

REVIEWERS' COMMENTS:

Reviewer #1 (Remarks to the Author):

The authors have addressed the points raised for the previous version. The manuscript appears suitable for publication in Nature Communications.

Reviewer #2 (Remarks to the Author):

The authors reported radical cyclization reactions of ortho-halotoluenes and nitriles in the presence of catalytic amounts of Cu(II) salt and KOtBu. They investigated the points of my concern and confirmed Cu(II) in the current reaction system. This observation might support existence of the Cu(II)-Cu(I) catalytic cycle. I think this article can be accepted on Nat. Commun.

In page 4, “Discussion” should be “Conclusion.”

[Reply]: Done.
